# Generalizing Vinyl Halide Cross‐Coupling Reactions with Photoredox and Photoredox/Nickel Dual Catalysis

**DOI:** 10.1002/anie.202510715

**Published:** 2025-08-29

**Authors:** Kousik Das, Nayan Saha, Zhuofan Li, Indrajit Ghosh, Burkhard König

**Affiliations:** ^1^ Fakultät für Chemie und Pharmazie Universität Regensburg 93053 Regensburg Germany; ^2^ Nanotechnology Centre, Centre for Energy and Environmental Technologies VSB – Technical University of Ostrava Ostrava‐Poruba 708 00 Czech Republic

**Keywords:** Cross‐coupling, General method, Nickel, Photoredox, Vinyl halide

## Abstract

Reliable, broadly applicable cross‐coupling conditions that deliver the desired products with minimal optimization are essential in pharmaceutical research, where efficient synthesis accelerates lead discovery and late‐stage diversification. Although advances like high‐throughput additive screening and commercial catalyst/ligand libraries improve prediction in specific systems, a general strategy for vinyl halide cross‐coupling across diverse bond‐forming reactions remains elusive. Herein, we report a general and highly predictable method for vinyl halide cross‐coupling under photoredox conditions, employing two complementary catalytic systems. In the first, the organic photocatalyst 4CzIPN enables efficient coupling with nucleophiles such as thiols, selenols, sulfinate salts, activated alkenes, phosphorus (III), and boron compounds, affording C(sp^2^)─S, ─Se, ─C, ─P, and ─B bonds in high yields. In the second, a dual nickelphotoredox catalytic system facilitates coupling with less reactive nucleophiles, including phosphorus (V), nitrogen, and oxygen. This approach enables seven distinct bond‐forming reactions, offering broad electrophile and nucleophile scope, high functional group tolerance, and applicability to the late‐stage functionalization of complex biomolecules. The simple and consistent conditions enable one‐pot, two‐step bifunctional transformations by sequentially activating distinct chemical bonds involving nucleophiles and electrophiles.

## Graphical abstract

A general strategy has been developed for predictable and robust vinyl halide cross‐coupling reactions. This approach demonstrates broad applicability across seven distinct bond‐forming transformations, featuring wide electrophile and nucleophile scope, high tolerance for functional groups, and suitability for late‐stage functionalization of complex biomolecules. It also enables efficient one‐pot, two‐step bifunctional transformations.

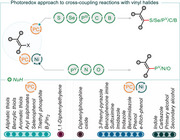

## Introduction

Over the past five decades, cross‐coupling reactions have become integral to modern organic synthesis, enabling the efficient formation of carbon─carbon (C─C) and carbon─heteroatom (C─X) bonds.^[^ ^1^, ^2^, ^3^, ^4^, ^5^ ^]^ Despite significant advances—particularly in palladium, copper, and nickel catalysis—achieving high efficiency remains challenging due to the need to fine‐tune multiple parameters, including pre‐catalysts, ligands, bases, solvents, temperature, and additives.^[^ ^6^ ^]^ The electronic and structural diversity of coupling partners often necessitates case‐by‐case optimization to achieve desirable yields and selectivity. To address this challenge, there is a growing demand in drug discovery for broadly applicable, robust, and predictable cross‐coupling protocols that require minimal optimization for the effective diversification of potential drug candidates. Recent developments—such as commercial pre‐catalyst/ligand libraries, high‐throughput additive screening, and machine learning‐based condition prediction—have accelerated optimization for specific transformations, particularly C(sp^2^)─C and C(sp^2^)─N bond formation involving aryl halides.^[^ ^7^, ^8^ ^]^ However, generalizable methods for vinyl halide cross‐coupling with a broad range of commercially available, inexpensive, and structurally diverse nucleophiles (e.g., thiols, selenols, sulfinate salts, alkenes, phosphorus(III) and (V) species, boron‐based reagents, nitrogen nucleophiles, and alcohols) to form C(vinyl)─S, ─Se, ─C, ─B, ─P, ─N, and ─O bonds remain underdeveloped. Expanding these methodologies would significantly enhance the versatility and applicability of cross‐coupling reactions involving vinyl halides.

**Figure 1:**
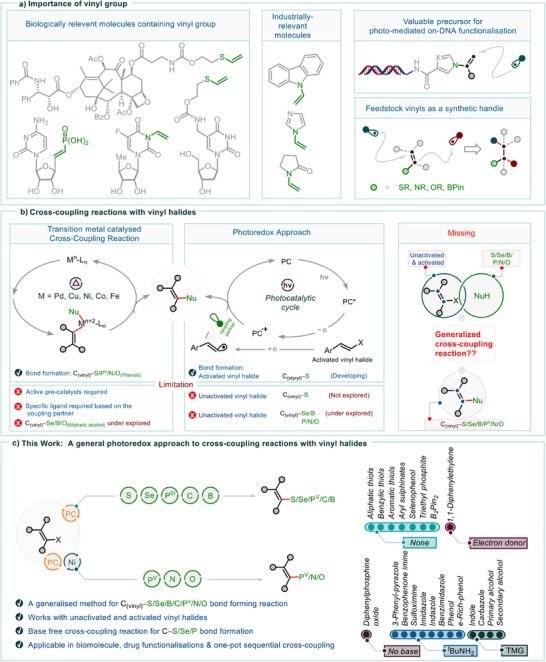
A general overview of the significance of the vinyl moiety and conventional approaches to cross‐coupling reactions. A general photoredox strategy for cross‐coupling reactions with vinyl halides, highlighting the classification of commonly used nucleophiles based on reaction conditions under visible‐light‐driven redox catalysis.

This limitation is particularly striking given the prevalence of vinyl groups in natural products, bioactive molecules,^[^ ^9^, ^10^, ^11^, ^12^ ^]^ and advanced materials.^[^ ^13^, ^14^, ^15^, ^16^, ^17^ ^]^ Vinyl derivatives are valuable synthetic intermediates^[^ ^18^ ^]^ and participate in a wide range of photochemical transformations,^[^ ^19^, ^20^, ^21^ ^]^ including DNA functionalization^[^ ^22^ ^]^ (Figure 1a). Accordingly, the development of modular and reliable cross‐coupling strategies involving vinyl halides could significantly advance the incorporation of vinyl groups in both early‐stage lead discovery and late‐stage functionalization.

Classical vinyl halide cross‐couplings rely on precise metal selection and specialized ligand design depending on the substrate and require harsh or highly specific conditions that are often limited to specific bond‐forming reactions.^[^ ^23^, ^24^, ^25^, ^26^, ^27^ ^]^ For instance, palladium‐catalyzed C─S bond formation with vinyl bromides is feasible using tris(dibenzylideneacetone)dipalladium(0) (Pd_2_(dba)_3_) with 1,1′‐bis(diphenylphosphino)ferrocene (dppf) as a ligand and lithium bis(trimethylsilyl)amide as a strong base at high temperature. In contrast, less reactive vinyl chlorides require alternative ligands such as CyPF*t*‐Bu, further underscoring the need for customized conditions.^[^ ^23^ ^]^ Similar challenges arise in C─N and C─O cross‐coupling reactions, which often require strong bases (e.g., sodium *tert*‐butoxide) and elevated temperatures, resulting in narrow substrate scope and limited generalizability. For example, α‐bromostyrenes couple efficiently with sulfoximines, yet β‐bromostyrenes fail to react, revealing inherent limitations in C─N bond formation.^[^ ^24^ ^]^ Likewise, while Pd‐catalyzed C─O coupling is well‐established for phenols, no examples have been reported for aliphatic alcohols reacting with unactivated vinyl bromides.^[^ ^28^ ^]^ Beyond substrate limitations, these methodologies frequently rely on specialized ligands that are not always readily available and can be costly. Additionally, the need for large ligand quantities complicates product purification. While efforts to develop more sustainable, earth‐abundant metal catalysts, such as copper, have shown promise, these reactions often require stoichiometric or excess amounts of metal and ligand, reducing their practical applicability and scalability.^[^ ^29^ ^]^ Bimetallic catalytic systems, such as Ni(ligand)_2_ or Co(ligand)_2_, frequently require co‐catalysts like CuI and high temperature to facilitate reactions with challenging nucleophiles such as phenols.^[^ ^25^, ^30^ ^]^ Nonetheless, these conditions necessitate extensive optimization to minimize side reactions, including vinyl halide reduction, alkyne formation,^[^ ^31^ ^]^ and dimerization.^[^ ^25^ ^]^ Recent advances in photoredox catalysis have facilitated selective cross‐coupling reactions, such as C─S bond formation, using either photocatalysts^[^ ^32^ ^]^ or electron donor–acceptor (EDA) conditions^[^ ^33^ ^]^ (Figure 1b). However, these methods remain inherently substrate‐specific, often requiring activated (styryl)bromides as vinyl halide substrates, which restricts their generality and broader applicability in late‐stage biomolecule functionalization and drug optimization. Furthermore, photoredox‐mediated cross‐coupling for the formation of C(sp^2^)─Se, C(sp^2^)─B, C(sp^2^)─P, C(sp^2^)─N, and C(sp^2^)─O bonds remains insufficiently explored, particularly when unactivated vinyl halides are employed (Figure 1b). Therefore, the establishment of a general and robust reaction protocol capable of engaging a diverse array of nucleophiles (e.g., thiols, selenols, sulfinate salts, alkenes, phosphorus(III) and (V) species, boron‐based reagents, nitrogen nucleophiles, and alcohols) and electrophiles, including both unactivated and activated vinyl halides, to form C(vinyl)─S, ─Se, ─C, ─B, ─P, ─N, and ─O bonds, would effectively address current limitations. Such advancement could facilitate streamlined synthetic access to functionalized advanced materials and bioactive molecules, while significantly broadening the methodology's versatility and reliability under well‐defined reaction conditions.

Here, we report a broadly applicable strategy for cross‐coupling reactions with vinyl halides under photoredox conditions. By leveraging the inherent reactivity of vinyl halides and nucleophiles as coupling partners, we establish two distinct reaction pathways. In the first approach, the photocatalyst 4CzIPN efficiently couples vinyl halides with nucleophiles such as thiols, selenols, sulfinate salts, activated alkenes, phosphorus(III), and boron compounds, yielding C(sp^2^)─S, C(sp^2^)─Se, C(sp^2^)─C, C(sp^2^)─P, and C(sp^2^)─B bonds in good to excellent yields. In a complementary approach, a Ni/photoredox system enables coupling with less reactive nucleophiles, including phosphorus(V), nitrogen, and oxygen, facilitating the formation of C(sp^2^)─P(V), C(sp^2^)─N, and C(sp^2^)─O bonds. This strategy enables the efficient formation of seven distinct C(sp^2^)─X bond types (C(sp^2^)─S, ─Se, ─N, ─P, ─B, ─O, and ─C), accommodating a broad range of nucleophiles and electrophiles, thereby enhancing the reaction's applicability and generality. Furthermore, it provides easy access to functionalized biomolecules bearing a vinyl moiety and facilitates the construction of complex molecular architectures incorporating a double bond via one‐pot, two‐step bifunctional transformations. This methodology enables the sequential activation of distinct chemical bonds, engaging both nucleophilic and electrophilic species, thereby allowing for the precise formation of multiple bonds within a single synthetic sequence. Its efficiency and reliability provide a versatile strategy for constructing complex molecules with minimal synthetic operations.

## Results and Discussion

**Figure 2:**
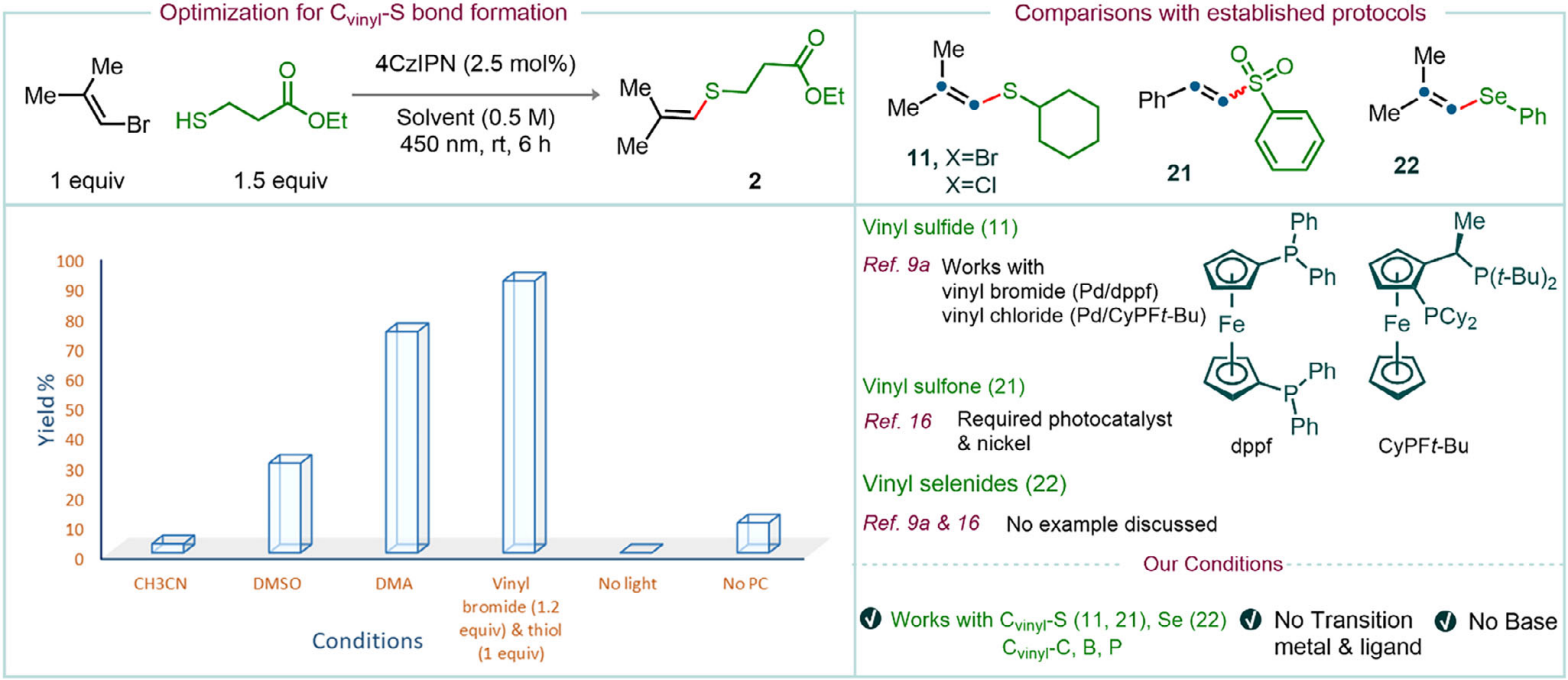
Optimization of reaction conditions for C_vinyl_─S bond‐forming reactions. The yields of the desired products were monitored by GC and GC‐MS analysis. A brief comparison of our protocol with previously reported methods is provided for representative nucleophiles, including an aliphatic thiol, phenyl sulfinate, and selenophenol.

We began our synthetic investigations using 1‐bromo‐2‐methyl‐1‐propene as a test electrophile in C(sp^2^)─S cross‐coupling reactions, with ethyl 3‐mercaptopropanoate as the coupling partner and 4CzIPN as the photocatalyst, in CH_3_CN (0.5 M) at room temperature under 450 nm light irradiation. To our delight, the desired cross‐coupled product **2** was obtained, albeit in only 3% yield (Figure 2, column 1). Changing the solvent to DMSO led to an improved yield of 30%, while the use of DMA proved most effective, affording product **2** in 74% yield (Figure 2, columns 2 and 3). Further optimization of the reaction conditions revealed that using a slight excess of vinyl bromide further improved the yield, delivering the desired product in 91% yield (Figure 2, column 4). Control experiments (Figure 2, columns 5 and 6) confirmed the essential roles of both light and 4CzIPN as the photocatalyst in this transformation (see Section 3.1 in the Supporting Information for further details). The reaction conditions are simple, requiring only mixing of the reagents (i.e., the reaction partners) and photocatalyst under air, followed by photoirradiation with a low‐power blue LED under nitrogen to achieve effective cross‐coupling. Additionally, unlike traditional cross‐coupling reactions, this transformation does not require any base or additives.

With the optimized reaction conditions in hand (see Section 3.1 in the Supporting Information for further details), we next explored the reaction scope by examining a variety of thiols as coupling partners with vinyl halides for C(sp^2^)─S bond formation. A broad range of thiols—including primary (products **1**–**7**), secondary (products **10**–**12**), tertiary (products **13** and **14**), benzylic (products **8** and **9**), and thiophenol derivatives (products **15**–**19**)—yielded the desired products in moderate to good isolated yields. Notably, steric hindrance, whether at the α‐position (cf. example **10**) or at the ortho positions (as in thiophenols, examples **17** and **18**), was well tolerated, affording the desired products in good to excellent yields. Heteroaryl thiols were also no exception and served as effective reaction partners; for example, the use of 2‐mercaptobenzothiazole furnished product **20** in 76% isolated yield.

**Figure 3:**
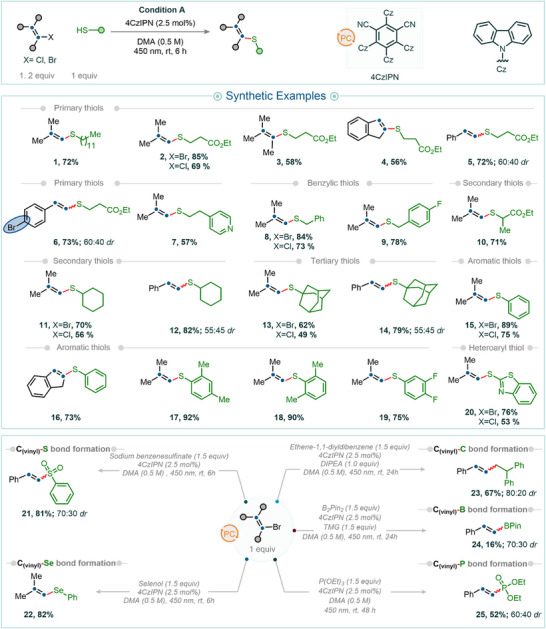
Synthetic examples of C(sp^2^)─S/Se/C/B/P bond‐forming reactions using vinyl halides. All reactions were carried out on a 0.2 mmol scale. Isolated yields are reported unless noted otherwise.

Under similar reaction conditions, aryl sulfinates—which previously required photoredox Ni/ligand dual catalytic systems^[^ ^34^ ^]^—also proved to be effective coupling partners. For example, the reaction of 2‐bromovinylbenzene with sodium phenylsulfinate afforded product **21** in 81% isolated yield. The cross‐coupling reactions demonstrated broad substrate compatibility, accommodating a diverse array of electrophiles. Various vinyl bromides—including 1‐bromo‐2‐methylprop‐1‐ene, 2‐bromo‐3‐methylbut‐2‐ene, 2‐bromo‐1*H*‐indene, (2‐bromovinyl)benzene, and 1‐bromo‐4‐(2‐bromovinyl)benzene—were efficiently converted to the desired products (cf. examples **2**, **3**, **4**, **5**, and **6**, respectively) in good to high isolated yields. Importantly, relatively challenging vinyl chlorides also proved effective as electrophiles. For example, the use of 1‐chloro‐2‐methylprop‐1‐ene in cross‐coupling reactions with thiols such as ethyl 3‐mercaptopropanoate, phenylmethanethiol, cyclohexanethiol, adamantane‐1‐thiol, thiophenol, and 2‐mercaptobenzothiazole furnished the desired products in moderate to good yields (cf. examples **2**, **8**, **11**, **13**, **15**, and **20**) (Figure 3).

Like sulfur nucleophiles, selenium‐based coupling partners also exhibited high reactivity under photochemical conditions with vinyl halides. For example, selenophenol furnished the desired product **22** in 82% yield under standard photocatalytic conditions. The scope of cross‐coupling reactions can also be readily extended to the formation of C(sp^2^)─C and C(sp^2^)─B bonds, requiring only the presence of a suitable base. For instance, the cross‐coupling of vinyl bromide with 1,1‐diphenylethylene proceeded efficiently in the presence of DIPEA (product **23**), an inexpensive and commercially available base. Similarly, the use of 1,1,3,3‐tetramethylguanidine (TMG) as a base enabled the coupling of B_2_Pin_2_, affording the corresponding C(sp^2^)─B bond product **24**. In addition, trialkyl phosphites (phosphorus in the +III oxidation state) served as competent nucleophiles, delivering the C(sp^2^)─P bond product **25** in good yield.

**Figure 4:**
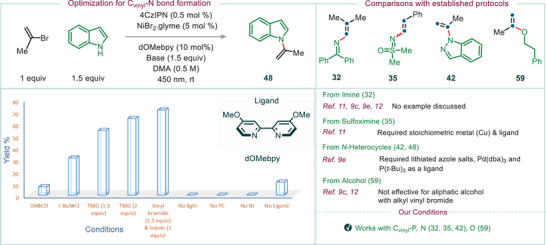
Reaction optimization for C(vinyl)─N bond formation, exemplified by the cross‐coupling of indole under dual photoredox/nickel catalysis. For detailed optimization studies involving other nucleophiles and the general experimental procedure, see Sections 3–5 of the Supporting Information. A brief comparison of our protocol with previously reported methods is provided for representative nucleophiles, including imines, sulfoximines, indazole, and an aliphatic alcohol.

The cross‐coupling reactions under photoredox–nickel dual catalysis, as depicted in Figure 1c, were found to be highly effective when applied to coupling partners that exhibit low reactivity with vinyl halides under photoredox‐only conditions. For instance, phosphorus nucleophiles in the +V oxidation state did not efficiently couple with vinyl halides in the presence of 4CzIPN alone (see Section 3.2 in the Supporting Information for further details) but required the addition of nickel for successful coupling. Notably, these reactions proceed without the need for a base, consistent with our previous findings,^[^ ^5^ ^]^ where base‐free conditions were effective. As illustrated in Figure 1c, cross‐coupling reactions involving nitrogen and oxygen nucleophiles similarly required the presence of nickel. However, in contrast to phosphorus nucleophiles, these reactions required the presence of a base to neutralize the acid generated during the process and to prevent protonation of the nucleophiles. In this context, although the formation of the desired cross‐coupled products was observed to varying extents with conventional soluble nitrogenous bases, a general trend emerged: nitrogen nucleophiles capable of coordinating to Ni(II)—such as imines, sulfoximines, and five‐membered heterocycles like indazoles, pyrazoles, and benzimidazoles (see Section 4 in the Supporting Information for changes in visual appearance)—underwent efficient cross‐coupling when *tert*‐butylamine was used as the base. In contrast, reactions involving relatively weak nucleophiles with low coordination propensity toward Ni(II) species—such as indoles–exhibited markedly enhanced efficiency when a stronger base, such as 1,1,3,3‐tetramethylguanidine (TMG), was employed. For example, in the cross‐coupling of indole with 2‐bromoprop‐1‐ene as a model electrophile, the desired product was obtained in low yield with DABCO (Figure 4, column 1). The yield increased substantially to 31% with *tert*‐butylamine and was further improved to 65% when TMG was used (Figure 4, columns 2 and 3). Under the optimized conditions—i.e., 2 equiv of TMG and 1.5 equiv of vinyl bromide—the desired product was obtained in 71% yield (Figure 4, column 5). Control experiments (Figure 4, columns 6–9) confirmed the essential roles of light, 4CzIPN, nickel, and the ligand in this transformation (see Section 3.3 in the Supporting Information for further details).

**Figure 5:**
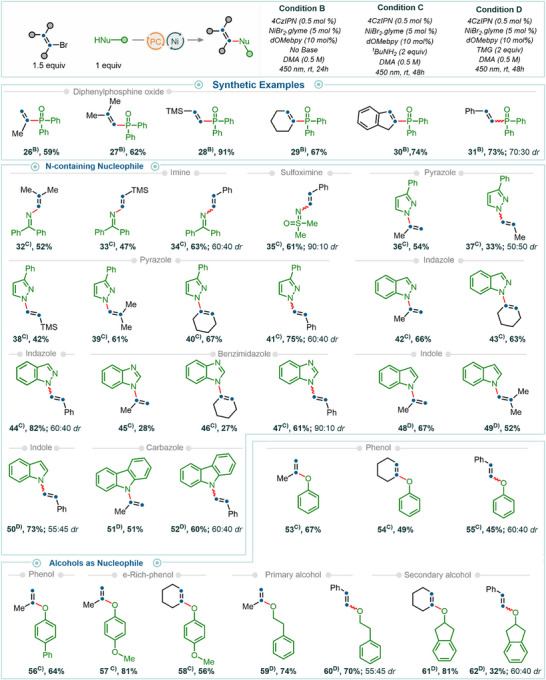
Synthetic examples of C(sp^2^)─P/N/O bond‐forming reactions using vinyl halides under nickel dual photoredox catalytic cross‐coupling reaction conditions on a 0.2 mmol scale. Isolated yields are reported unless noted otherwise. ^B)^Using condition B; ^C)^Using condition C; ^D)^Using condition D_._

Building on this insight, we next explored the generality of the method by evaluating a variety of nitrogen‐ and oxygen‐centered nucleophiles in cross‐coupling reactions. Pleasingly, various classes of nucleophiles were readily cross‐coupled with vinyl halides, affording the desired products in good to excellent isolated yields. For instance, when benzophenone imine was employed as a nucleophile, synthetically valuable vinyl imines (cf. examples **32**–**34**, Figure 5) were obtained in 47%–63% isolated yields. Notably, to the best of our knowledge, this represents one of the first examples of effective cross‐coupling between benzophenone imines and vinyl halides under photoredox conditions for the synthesis of vinyl imines (see Figure 4 for a comparison of our protocol with previously reported methods).

More importantly, sulfoximines—recently recognized as promising bioisosteres for sulfones and sulfonamides in drug discovery^[^ ^35^ ^]^ —also proved to be competent coupling partners. *N*‐vinylated sulfoximines are not only considered enamide analogues but have also been shown to exhibit potential biological activity. Although cross‐coupling reactions with sulfoximines have been previously reported,^[^ ^29^ ^]^ they typically require stoichiometric amounts of metal reagents (e.g., copper) and ligands. In contrast, our protocol enables efficient cross‐coupling under mild photoredox conditions, delivering, for example, product **35** in 61% isolated yield. Furthermore, functionalized five‐membered heterocycles—including substituted pyrazoles (**36**–**41**), indazoles (**42**–**44**), and biologically relevant benzimidazoles (**45**–**47**)—were also efficiently coupled under our conditions. Traditionally, such transformations require Pd‐catalyzed cross‐coupling reactions involving lithiated azole salts and P(*t*‐Bu)_3_ as a ligand at elevated temperature^[^ ^27^ ^]^ (see Figure 4 for a comparison of our protocol with previously reported methods). These substrates furnished the corresponding C─N cross‐coupled products in moderate to good isolated yields.

Finally, for less nucleophilic substrates such as indole—which exhibit relatively low coordination to Ni(II)—the use of TMG led to the formation of the desired products (**48**–**50**) in 52%–73% isolated yields when electrophiles 2‐bromoprop‐1‐ene (**48**), 1‐bromo‐2‐methylprop‐1‐ene (**49**), and (2‐bromovinyl)benzene (**50**) were used, respectively. As expected, carbazole displayed similar reactivity to indole, and its coupling in the presence of TMG afforded products **51** and **52** when 2‐bromoprop‐1‐ene and (2‐bromovinyl)benzene were used as electrophiles, respectively, in good isolated yields.

Comparable reactivity trends were observed with oxygen nucleophiles. It is worth noting that transition metal‐catalyzed C(sp^2^, vinyl)─O cross‐coupling reactions for phenol derivatives typically require bimetallic catalytic systems, such as Ni(ligand)_2_ or Co(ligand)_2_, along with co‐catalysts like CuI for effective coupling.^[^ ^25^, ^30^ ^]^ And to the best of our knowledge, cross‐coupling reactions of aliphatic alcohols with aliphatic vinyl bromides are not explored (see Figure 4). Notably, both classes of O‐nucleophiles—aromatic and aliphatic alcohols—can be cross‐coupled under our reaction conditions (see Figure 5). More reactive oxygen nucleophiles, such as phenols, were efficiently cross‐coupled in the presence of *tert*‐butylamine (products **53**–**58**). For less reactive oxygen nucleophiles, such as aliphatic alcohols, the use of TMG was essential to achieve good yields. A range of vinyl bromides—including 1‐bromocyclohex‐1‐ene—proved compatible under the reaction conditions, delivering the desired cross‐coupled products with primary and even with secondary alcohols (**59**–**62**) in moderate to good isolated yields.

**Figure 6:**
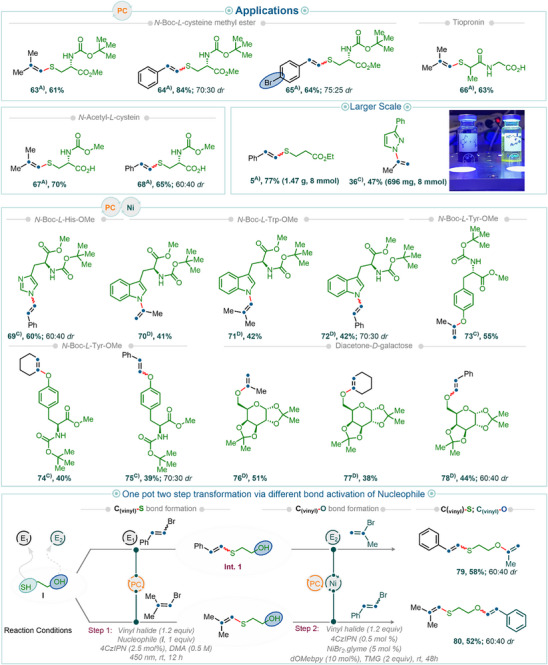
Synthetic examples of functionalization of biomolecules and one‐pot two‐step synthetic transformations using a nucleophile possessing two different functional groups on a 0.2 mmol scale. Isolated yields are reported unless noted otherwise. ^A)^Using condition A (vinyl bromide (1.2 equiv), thiol (1 equiv), 4CzIPN (2.5 mol%) DMA (0.5 M) 450 nm, rt, 6 h); ^C)^Using condition C; ^D)^Using condition D_._

The simplicity of the reaction conditions—requiring only mixing of the starting materials under air and conducting the reactions under an inert atmosphere—enabled straightforward execution of the cross‐coupling reactions on a gram scale (Figure 6). For instance, the reaction between (2‐bromovinyl)benzene and ethyl 3‐mercaptopropanoate afforded the desired C─S bond‐forming product **5** on a gram scale, even with low photocatalyst loadings. The gram‐scale reactions were also effective for other bond‐forming reactions. For example, the coupling of 2‐bromoprop‐1‐ene and 3‐phenylpyrazole yielded product **36** in 47% yield on a gram scale.

**Figure 7:**
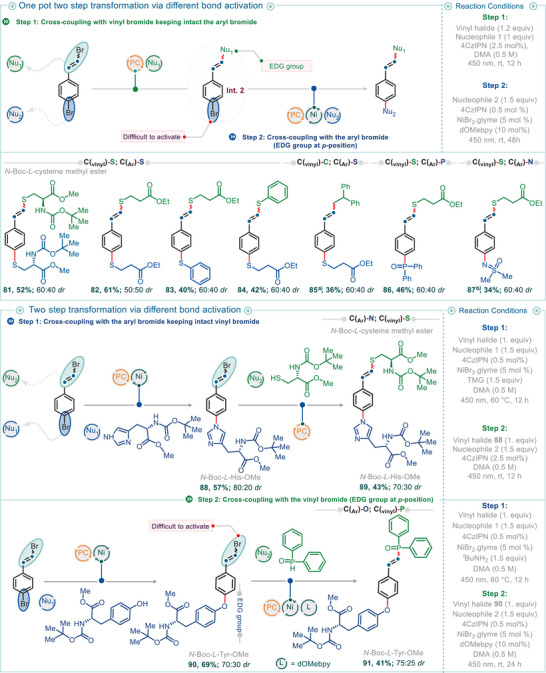
Synthetic examples of two‐step synthetic transformations involving distinct bond activation using an electrophile. All reactions were carried out on a 0.2 mmol scale. Isolated yields are reported unless noted otherwise. ^a)^Required additive DIPEA (1.5 equiv in the step 1), ^b)^Using *t*‐BuNH2 (1.5 equiv) as a base in step 2.

The inherent straightforwardness and robustness of the vinyl bromide‐based cross‐coupling protocol make it particularly suitable for the functionalization of biomolecules. For example, when *N*‐Boc‐l‐cysteine methyl ester was used as a protected amino acid nucleophile, functionalization at the sulfur center with a vinyl moiety yielded the corresponding products (**63**–**65**) in good to excellent isolated yields. Moreover, when 1‐bromo‐4‐(2‐bromovinyl) benzene was used as the electrophile, selective functionalization of the C_vinyl_─Br bond was achieved (**65**), leaving the C_aryl_─Br bond intact for further functionalization (see Figure 7 for one‐pot bi‐functionalization reactions involving sequential C_vinyl_─Br and C_aryl_─Br functionalization). Notably, the cross‐coupling protocol is so robust that *S*‐vinylated products were obtained even with *N*‐acetyl‐l‐cysteine (**67** and **68**), which possesses a free carboxylic acid, and with tiopronin (**66**), which contains both a free carboxylic acid and significant steric bulk adjacent to the sulfur center.

Functionalization of biomolecules was effective not only with sulfur‐containing compounds but also with nitrogen‐ and oxygen‐containing nucleophiles, including phenols and alcohols. For instance, the use of *N*‐Boc‐l‐His‐OMe and *N*‐Boc‐l‐Trp‐OMe as nucleophiles resulted in *N*‐vinylated imidazole (**69**) and indole derivatives (**70**–**72**) in good yields. Similarly, oxygen‐based nucleophiles such as *N*‐Boc‐l‐Tyr‐OMe and diacetone‐d‐galactose furnished phenolic and aliphatic vinyl ethers (**73**–**78**) in moderate to good isolated yields. Within the investigated electrophile scope, a variety of vinyl halides—including 2‐bromoprop‐1‐ene, 1‐bromo‐2‐methylprop‐1‐ene, 1‐bromocyclohex‐1‐ene, and (2‐bromovinyl) benzene—were compatible, efficiently introducing diverse vinyl moieties into the products (cf. **70**, **71**, **74**, and **78**) in moderate to good yields.

The simplicity and versatility of the vinyl halide cross‐coupling method became more apparent when we turned our attention to bi‐functionalization reactions, enabling the formation of two distinct chemical bonds for the rapid construction of molecular complexity (Figure 6). For example, using 2‐mercaptoethanol—which contains both ─SH and ─OH groups in a 1,4‐heteroatom arrangement—in a one‐pot, two‐step sequence, the reaction of (2‐bromovinyl)benzene under photoredox conditions yielded intermediate **Int. 1** (Figure 6). Subsequent addition of 2‐bromoprop‐1‐ene to the same mixture under dual photoredox–nickel conditions afforded the desired bifunctionalized product, (2‐(prop‐1‐en‐2‐yloxy) ethyl) (styryl)sulfane (**79**), in 58% yield. The use of different vinyl bromides in this bifunctionalization sequence was also successful in providing the desired products. For example, the use of 1‐bromo‐2‐methylprop‐1‐ene and (2‐bromovinyl) benzene, respectively, yielded product **80** in 52% isolated yield. Leveraging the inherent efficiency and reliability of vinyl bromide activation, we next questioned whether this methodology could be effectively combined with other bond activation modes—specifically, aryl bromides (C_aryl_─Br). We recognized that while vinyl bromides can be activated under photocatalytic conditions using only a photocatalyst (PC, in this case, 4CzIPN) and nucleophiles such as thiols, the activation of C_aryl_─Br bonds typically requires a dual catalytic system involving both a photocatalyst and nickel.^[^ ^5^ ^]^ Encouragingly, these two distinct bond activation modes could be seamlessly integrated, enabling one‐pot bifunctional transformations of electrophiles bearing both vinyl and aryl bromides. This allowed for the installation of either one or two distinct functional groups at separate sites within a single molecule, all in a one‐pot, two‐step sequence. For instance, 1‐bromo‐4‐(2‐bromovinyl) benzene, which contains both C_vinyl_─Br and C_aryl_─Br bonds, underwent selective activation of the vinyl bromide moiety in the presence of ethyl 3‐mercaptopropanoate and a photocatalyst (PC only), affording intermediate **Int. 2** (ethyl 3‐((4‐bromostyryl)thio)propanoate; see Figure 7). Subsequent addition of ethyl 3‐mercaptopropanoate under dual catalytic conditions (PC + Ni) led to the formation of bi‐functionalized product **82** in very good isolated yield via a two‐step transformation. As anticipated, two different thiols—for example, ethyl 3‐mercaptopropanoate (an aliphatic thiol) and thiophenol—could also be used sequentially, resulting in the installation of two distinct functional groups at the vinyl and aryl sites (cf. product **83**). Notably, the simplicity of the reaction conditions and the broad scope of C(sp^2^)─S bond‐forming reactions allowed for a straightforward reversal of the thiol addition sequence, leading to an inversion of functional group installation at the vinyl and aryl sites (cf. products **83** and **84**). Moreover, these sequential reactions were not limited to the formation of identical bonds; different types of bonds could also be forged in sequence by employing distinct nucleophiles. For example, starting from 1‐bromo‐4‐(2‐bromovinyl)benzene and ethyl 3‐mercaptopropanoate, initial activation of the vinyl bromide furnished intermediate **Int. 2**, as previously described. Upon addition of diphenylphosphine oxide or iminodimethyl‐*λ*⁶‐sulfanone under dual photoredox/nickel catalytic conditions for aryl bromide activation in the second step, sequential formation of C_vinyl_─S and C_aryl_─P (product **86**, 46% yield) or C_vinyl_─S and C_aryl_─N (product **87**, 34% yield) bonds was achieved. Interestingly, alternative coupling partners could also be employed in the first step. For instance, the stepwise use of 1,1‐diphenylethylene in the presence of DIPEA and ethyl 3‐mercaptopropanoate resulted in the formation of compound **85** via a one‐pot C_vinyl_─C and C_aryl_─S bond‐forming sequence.

Finally, we explored whether the order of these two bond activations—that are, C_vinyl_─Br and C_aryl_─Br—could be reversed. Our studies revealed that while activation of the C_vinyl_─Br bond under dual photoredox/nickel catalysis requires the presence of a ligand, activation of the C_aryl_─Br bond can proceed without one.^[^ ^5^ ^]^ This subtle but crucial distinction in reaction requirements enabled selective activation of the aryl bromide while preserving the vinyl bromide for subsequent functionalization. This strategy proved highly effective—even with structurally complex and diverse biomolecules as coupling partners—allowing for two distinct bond‐forming events in a controlled, sequential manner. For example, the reaction of 1‐bromo‐4‐(2‐bromovinyl)benzene with *N*‐Boc‐l‐His‐OMe and *N*‐Boc‐l‐Tyr‐OMe under dual catalytic conditions (PC + Ni) led to aryl‐functionalized intermediates **88** and **90**. Subsequent addition of *N*‐acetyl‐l‐cysteine methyl ester under photocatalytic‐only conditions (PC) or diphenylphosphine oxide under dual photoredox/nickel conditions and in the presence of ligand activated the vinyl bromide, affording bifunctionalized products **89** and **91**. These transformations correspond to C_aryl_─N/C_vinyl_─S and C_aryl_─O/C_vinyl_─P bond‐forming sequences, respectively, with isolated yields of 43% and 41%.

### Mechanistic Studies

Although proposing a comprehensive mechanism that accounts for seven different bond‐forming reactions—using a diverse range of vinyl halides and coupling/nucleophile partners under two distinct regimes (i.e., photoredox and dual photoredox/nickel catalysis)—is inherently challenging, the observed reactivity trends are generally consistent with those reported in the photoredox catalysis literature. For example, S, Se, P(III), B, and C‐based coupling partners readily react with aryl or vinyl halides under the photoredox reaction conditions. In contrast, N and O nucleophiles typically do not undergo direct coupling under photoredox conditions and usually require a transition metal to facilitate bond formation. This trend is also observed in our system.

**Figure 8:**
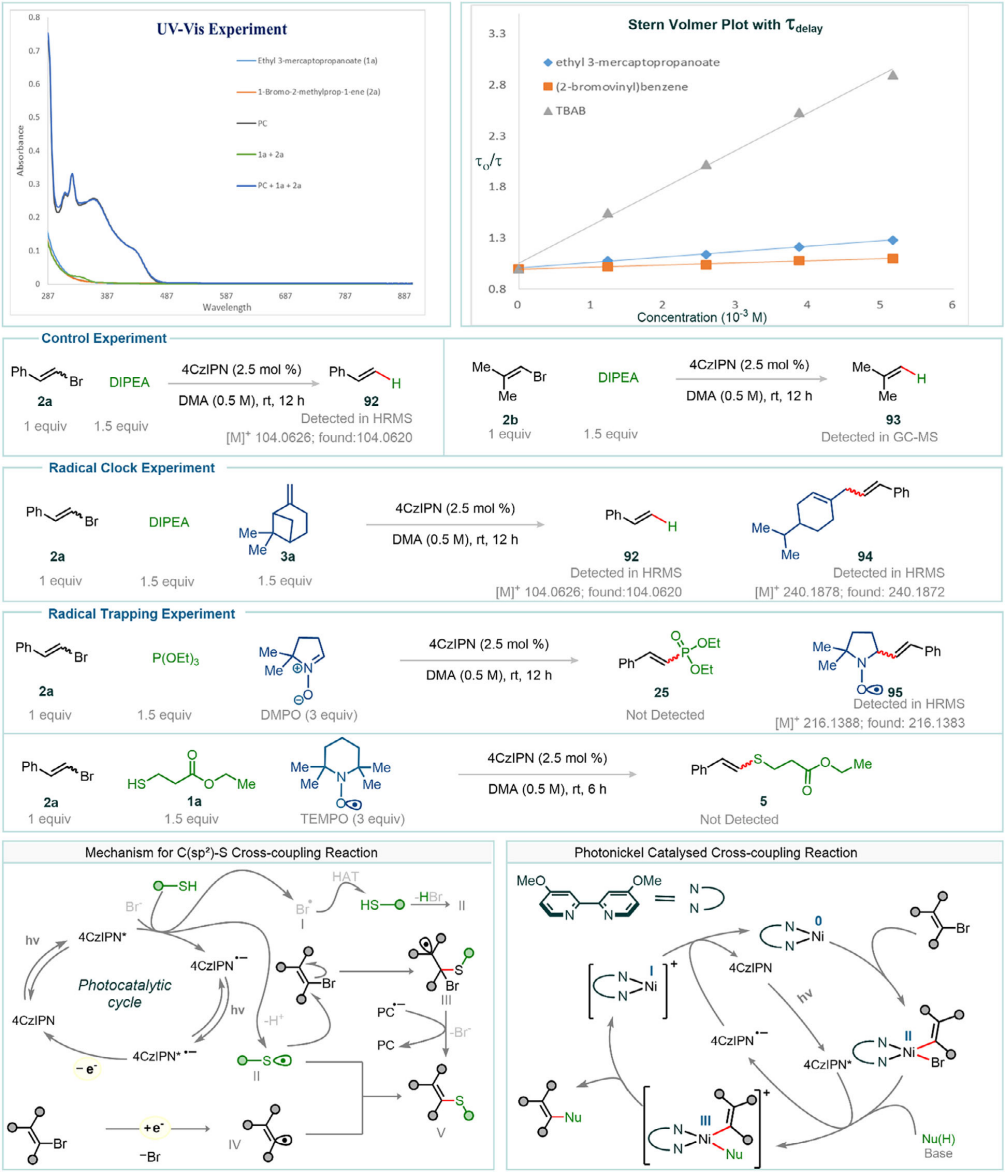
Spectroscopic investigations and a proposed mechanism for C(sp^2^)─S bond‐forming reactions, along with a simplified mechanistic model for the photonickel dual catalytic system, are presented. UV–vis experiments along with time‐resolved quenching studies of 4CzIPN with vinyl bromide, thiol, and tetrabutylammonium bromide (TBAB) are also shown. For further details, see Sections 6 and 7 in the Supporting Information.

To elucidate the mechanism of the purely photoredox‐mediated pathway, a series of experimental investigations were conducted. Initial insights were gained through photoluminescence (both steady‐state and time‐resolved) quenching studies, which probed the interaction between the excited‐state photocatalyst and various reaction components. The organic photocatalyst 4CzIPN, which absorbs visible light, has excited‐state redox potentials of *E*_1/2_(PC*/PC^•–^) = +1.4 V versus SCE and *E*_1/2_(PC^•+^/PC*) = −1.2 V versus SCE.^[^ ^36^ ^]^ Upon addition of 2‐bromovinylbenzene, only a very slight change in the steady‐state photoluminescence and excited‐state lifetime of 4CzIPN was observed. The quenching effect was more pronounced with ethyl 3‐mercaptopropanoate but significantly increased in the presence of tetrabutylammonium bromide (TBAB), a representative soluble bromide salt (see below and Figure 8 for the Stern–Volmer plot). A clear linear Stern–Volmer relationship was observed in the latter case, indicating effective quenching. To determine whether the reduced photocatalyst in the ground state is capable of directly reducing the vinyl bromide, cyclic voltammetry (CV) experiments were carried out (see Section 6.1 in the Supporting Information). The reduction potentials of vinyl bromides were found to be more negative^[^ ^37^ ^]^ than that of the reduced ground‐state 4CzIPN, suggesting that a direct single‐electron transfer from the reduced photocatalyst to the vinyl halide is unlikely. Taking these observations into account, we propose the following plausible mechanism for the thiol coupling reactions (for a more detailed mechanistic discussion, see section 7.1 in the Supporting Information). The photochemical transformation is initiated by the interaction of the thiol with the excited‐state photocatalyst, generating a thiyl radical(II) via oxidation followed by a proton loss. This thiyl radical(II) then adds to the vinyl bromide, forming intermediate III, which can be reduced by the ground‐state reduced photocatalyst. Debromination then furnishes the desired product. As the reaction proceeds, bromide anions accumulate in the reaction medium, potentially altering the mechanistic pathway. In this scenario, bromide ions may undergo oxidation to generate bromine radicals (Br•, cf., effective quenching of excited‐state luminescence of 4CzIPN in the presence of TBAB),^[^ ^38^ ^]^ which can abstract a hydrogen atom via hydrogen atom transfer (HAT), thereby regenerating the thiyl radical(II). The reaction then proceeds as before. It is to be noted here that recent studies have demonstrated that the excited‐state radical anion of 4CzIPN is a highly potent reductant.^[^ ^39^, ^40^, ^41^, ^42^, ^43^, ^44^, ^45^, ^46^ ^]^ The formation of this species in the reaction mixture under photoredox catalytic conditions can facilitate the reduction of organic halides with very high (negative) reduction potentials. For instance, when compounds **2a** and **2b** were photoirradiated in the presence of 4CzIPN and *N,N*‐diisopropylethylamine (DIPEA), the corresponding reduction products (**92** and **93**) were detected by HRMS and GC‐MS analysis (see control experiments in Figure 8). Additionally, radical trapping experiments led to the detection of species **95** via HRMS. Taking these experimental results and recent literature into account, a vinyl radical pathway for the formation of the desired product may also be operative, with its contribution varying depending on the reaction partners and reaction conditions.

In the dual photoredox/nickel cross‐coupling protocol, two catalytic cycles can be envisioned: a Ni(0)/Ni(II)/Ni(III)^[^ ^47^, ^48^, ^49^ ^]^ pathway and a Ni(I)/Ni(III)^[^ ^50^, ^51^, ^52^, ^53^ ^]^ pathway. In the former, the ligated Ni(II) species is reduced under photoredox conditions to generate a Ni(0) complex. This Ni(0) species undergoes oxidative addition with the vinyl halide to form a Ni(II) intermediate. Subsequent single‐electron oxidation by the excited state of the photocatalyst converts this Ni(II) intermediate into a Ni(III) species. Ligand exchange (in this case, Br^−^ is being replaced by the nucleophile), followed by reductive elimination from the Ni(III) species, yields the cross‐coupled product and forms a Ni(I) species. This Ni(I) species can be further reduced by the reduced state of photocatalyst to regenerate Ni(0), thereby completing the catalytic cycle, while simultaneously regenerating the ground‐state photocatalyst. Notably, depending on the nucleophile, a nucleophile‐ligated nickel species—coordinated either with the nucleophile itself or its corresponding anion—may form prior to oxidative addition. This effect is supported by a noticeable color change observed upon the addition of TMG to the reaction mixture (see Section 4 in the Supporting Information), suggesting a potential role for TMG in facilitating oxidative addition.

In the Ni(I)/Ni(III) pathway, Ni(II) is initially reduced to Ni(I) by the photocatalyst. The resulting Ni(I) species undergoes oxidative addition with the vinyl halide to form a Ni(III) intermediate, which then undergoes reductive elimination to yield the final product and regenerate the Ni(I) species. This cycle can be self‐sustaining, and its efficiency may depend on the specific combination of nucleophile and electrophile used. It is important to note that Ni(0)/Ni(II)/Ni(III) cycles typically require stabilizing ligands^[^ ^47^, ^48^, ^49^ ^]^ (e.g., bipyridine) to form and maintain the Ni(0) species, whereas Ni(I)/Ni(III) pathways can operate in both the presence and absence of such ligands.^[^ ^5^, ^50^, ^51^, ^52^, ^53^ ^]^ In our system, photochemical reactions proceed under both conditions (cf. columns 5 and 9 in Figure 4); though, the presence of bipyridine ligands significantly enhances product yields. We therefore believe that both catalytic pathways may be operative under our reaction conditions. The extent to which each pathway contributes likely depends on the specific substrate combination and the conditions used for a given cross‐coupling reaction (see Section 7.2 in the Supporting Information for further details).

## Conclusion

In conclusion, we report a general and versatile method for cross‐coupling reactions using vinyl halides to form C(sp^2^)─C(sp^3^) bonds and six different C(sp^2^)─(het)atom bonds under remarkably simple—and, more importantly, predictable—reaction conditions using visible light. This approach effectively accommodates a broad range of nucleophiles—including thiols, selenols, sulfinate salts, activated alkenes, phosphorus(III) and (V) species, boron‐based reagents, *N*‐nucleophiles, and alcohols—as well as both unactivated and activated vinyl halides, enabling applications such as biomolecule functionalization and one‐pot, two‐step transformations involving both electrophilic and nucleophilic partners. We anticipate that this methodology will not only simplify cross‐coupling reactions, enabling the efficient synthesis of a diverse array of products and expanding the applicability of vinyl halide chemistry, but also provide synthetic chemists with a reliable tool for constructing complex molecules. Furthermore, it holds significant potential for integration into predictive machine‐learning models.

## Conflict of Interests

The authors declare no conflict of interest.

## Supporting information



Supporting Information

## Data Availability

The data that support the findings of this study are available in the Supporting Information of this article.
