# Supplementary material for: Generalizing Vinyl Halide Cross‐Coupling Reactions with Photoredox and Photoredox/Nickel Dual Catalysis
Source: Angew Chem Int Ed Engl. 2025 Aug 29;64(42):e202510715. doi: 10.1002/anie.202510715 (PMC12518699; doi:10.1002/anie.202510715)

# Supporting Information for

# Generalizing vinyl halide cross-coupling reactions with photoredox and photoredox/nickel dual catalysis

Kousik Das[1], Nayan Saha[1], Zhuofan Li[1], Indrajit Ghosh[1,2,*], and Burkhard König[1,*]
[1]Fakultät für Chemie und Pharmazie, Universität Regensburg, 93053 Regensburg, Germany
[2]Current affiliation: Nanotechnology Centre, Centre for Energy and Environmental Technologies, VŠB–Technical University of Ostrava, 708 00 Ostrava-Poruba, Czech Republic
The correspondence may be addressed to: indrajit1.ghosh@ur.de (I.G.) and burkhard.koenig@ur.de (B.K)

## Table of Contents

1. General Information S2
2. Photographs and details of the photochemical reaction set-ups S2
3. Optimization of the Reaction Conditions S4
4. Naked-eye visual change in color/appearance upon addition of nucleophiles. to a solution of $NiBr_2$·glyme in dimethylacetamide S8
5. General Procedures and Unsuccessful Substrates S9
6. Spectroscopic investigations and Control Experiments S15
7. Possible mechanism/s for the formation of the desired product S22
8. Characterization data of the final product S25
9. References S65
10. Spectra for new compounds S66

## 1. General information

**Reagents and solvents:**

Commercially available chemicals were purchased at the highest commercial quality and used without further purification unless noted otherwise. 1,2,3,5-Tetrakis(carbazol-9-yl)-4,6-dicyanobenzene (4CzIPN) was synthesized according to a reported procedure[1]. All reactions were carried out in dry *N*,*N*-dimethylacetamide (DMA). DMA was dried with 3 Å molecular sieves.

**Gas chromatography**:

Gas chromatography with a flame-ionization detector (GC-FID) and gas chromatography coupled to low-resolution mass spectrometry (GC-MS) were performed using a capillary column (length: 30 m; diam.: 0.25 mm; film: 0.25 $\mu$M) using He as a carrier gas. GC-MS was performed on a 5975 MSD single quadrupole detector. Standard heating procedure: The initial temperature was set to 40 °C and was held for 3 minutes. Then, the temperature was increased to 280 °C at a rate of 15 °C/min and was held for 5 minutes. Lastly, the temperature was increased to 300 °C at a rate of 25 °C/min.

**TLC**:

Thin-layer chromatography (TLC) was performed on silica gel coated alumina plates (Macherey-Nagel, TLC sheets ALUGRAM Xtra SIL G UV254). Detection of the spots was accomplished utilizing UV light (254 nm).

**Flash column chromatography:**

Flash chromatography was performed either on an automated Biotage® IsoleraTM Spektra or Biotage® Selekt system using a prepacked Biotage® Sfär Silica HC Duo 20 µm 10 g column, if not stated otherwise.

**X-Ray** analysis was performed by the crystallography department of the University of Regensburg. The structures were solved by Birgit Hischa (University of Regensburg) and Sabine Stempfhuber (University of Regensburg).

**NMR:**

The NMR spectra were recorded at room temperature using a Bruker Avance 400 (400 MHz for $^{1}$H, 101 MHz for $^{13}$C, 376 MHz for $^{19}$F) NMR spectrometer. All chemical shifts are reported in $\delta$-scale as parts per million [ppm] (multiplicity, coupling constant J, number of protons) relative to the solvent residual peaks as the internal standard. Coupling constants J are given in Hertz [Hz]. Abbreviations used for signal multiplicity: $^{1}$H-NMR: br = broad, s = singlet, d = doublet, t = triplet, q = quartet, dd = doublet of doublets, dt = doublet of triplets, and m = multiplet.

**HRMS:**

High-resolution mass spectra (HRMS) were obtained from the central analytic mass spectrometry facilities of the Faculty of Chemistry and Pharmacy of the University of Regensburg. The measurements were carried out on either a JEOL AccuTOF GCX or Agilent Q-TOF 6540 UHD.

## 2. Photographs and details of the photochemical reaction set-ups

The photochemical reactions were performed using 455 (±15) nm LEDs (OSRAM Oslon SSL 80 LDCQ7P-1U3U (blue, $\lambda_{max}$ = 455 (±15) nm, $I_{max}$ = 1000 mA, 1.12 W) were used.

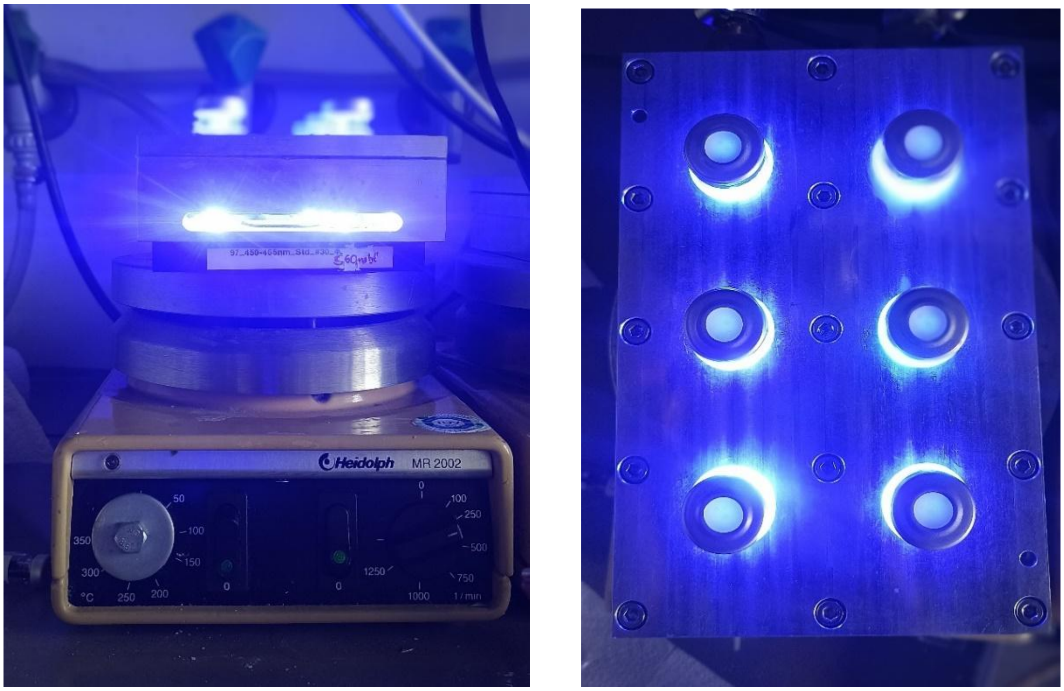


**Figure S1.** Side and top view of the photochemical reaction setup. The reaction vials (5 mL crimp cap vials) were illuminated from the bottom side with blue LEDs (λ = 455 (± 15) nm). The reaction temperature was maintained at either 25.0 °C or 60.0 °C by a custom-made thermostated aluminium cooling block.

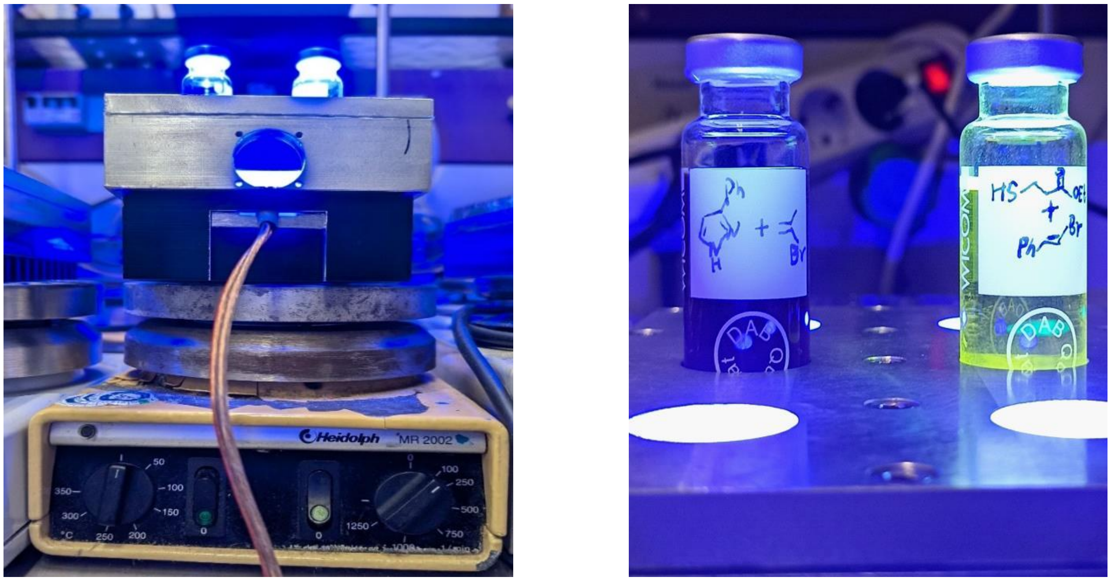


**Figure S2.** Side and top view of the photochemical larger reaction setup. The reaction vials (20 mL crimp cap vials) were illuminated from the bottom side with blue LEDs (λ = 455 (± 15) nm, HP, 1294 mW). The reaction temperature was maintained at either 25.0 °C or 60.0 °C by a custom-made thermostated aluminium cooling block

## 3. Optimization of the reaction conditions and control reactions

### 3.1 Optimization of reaction conditions for C-S bond formation

- **Screening of solvents**

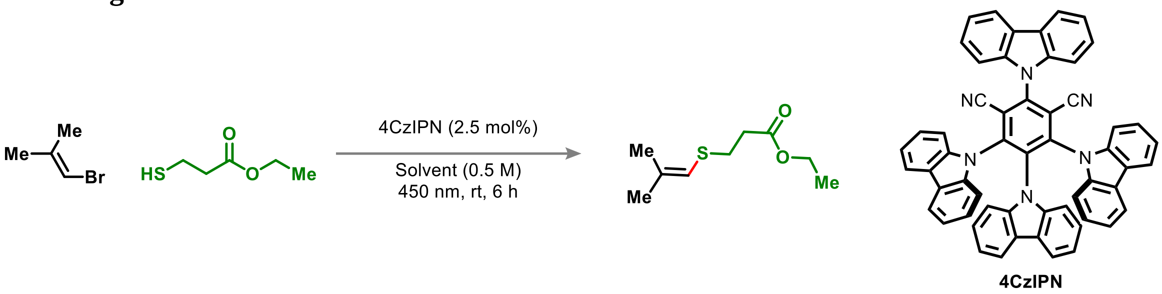


A 5 mL crimp top vial was charged with 1-bromo-2-methylprop-1-ene (21.0 µL, 0.20 mmol, 1.0 equiv), ethyl 3-mercaptopropanoate (38.0 µL, 0.30 mmol, 1.5 equiv), and 4CzIPN (4.0 mg, 2.5 mol%). Then the vial was crimped. The vial was degassed and refilled with nitrogen using the Schlenk-line technique (three times). Solvent (0.4 mL) was added to the mixture and stirred at room temperature for 3 h under the irradiation of a single blue LED ($\lambda_{Max}$ = 455 ± 15 nm). 1,3-Dimethoxybenzene (0.2 mmol) was added as an internal standard., and yields are calculated by GC-FID analysis.

| Entry | Solvent | Product |
|---|---|---|
| 1 | $CH_3CN$ | 3% |
| 2 | DMF | 65% |
| 3 | DMSO | 30% |
| **4** | **DMA** | **74%** |

- **Screening of stoichiometry of the reagents**

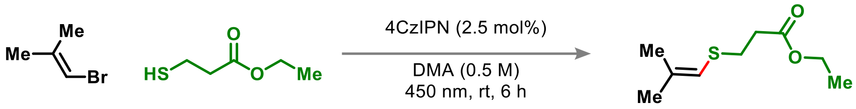


**xx equiv** **yy equiv**

A 5 mL crimp top vial was charged with 1-bromo-2-methylprop-1-ene (x.x equiv), ethyl 3-mercaptopropanoate (y.y equiv), 4CzIPN (4.0 mg, 2.5 mol%). Then the vial was crimped. The vial was degassed and refilled with nitrogen using the Schlenk-line technique (three times). Solvent dry DMA (0.4 mL) was added to the mixture. The vial was degassed and refilled with nitrogen using the Schlenk-line technique (three times) and stirred at room temperature for 3 h under the irradiation of a single blue LED (455 (± 15) nm). 1,3-Dimethoxybenzene (0.2 mmol) was added as an internal standard., and yields are calculated by GC-FID analysis.

| Entry | Vinyl bromide | Thiol | Product |
|---|---|---|---|
| **1** | **1** | **1.5** | 74% |
| **2** | **1.2** | **1** | **91%** |
| **3** | **1.5** | **1** | 90% |

- **Control Experiments**

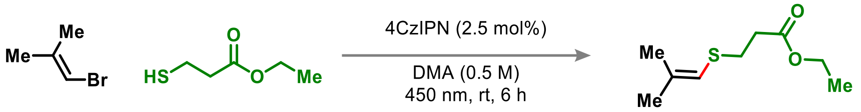


A 5 mL crimp top vial was charged with 1-bromo-2-methylprop-1-ene (25.0 µL, 0.24 mmol, 1.2 equiv), ethyl 3-mercaptopropanoate (26.0 µL, 0.20 mmol, 1.0 equiv), 4CzIPN (2.5 mol%). Then the vial was crimped. Solvent dry DMA (0.4 mL) was added to the mixture. The vial was degassed and refilled with nitrogen using the Schlenk-line technique (three times). and stirred at room temperature for 3 h under the irradiation of a single blue LED (455 (± 15) nm). 1,3-Dimethoxybenzene (0.2 mmol) was added as an internal standard., and yields are calculated by GC-FID analysis.

| Entry | Reaction component omitted | Product |
|---|---|---|
| 1 | Photocatalyst | 10% |
| 2 | Using 365 or 395 nm light source without PC | 10-12% |
| 3 | Light | ND |
| 4 | Without degassing | 25% |

### 3.2 Optimization of reaction for C-P bond forming reaction

- **Control Experiments**

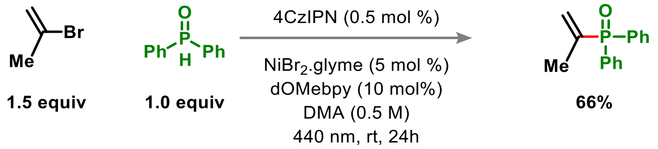


A 5 mL crimp top vial was charged with 2-bromoprop-1-ene (28 µL, 0.30 mmol, 1.5 equiv), diphenylphosphine oxide (40.0 mg, 0.20 mmol, 1.0 equiv), 4CzIPN (0.8 mg, 0.5 mol%), $NiBr_2$.glyme (3.1 mg, 5 mol%), 4,4'-Dimethoxy-2,2'-bipyridine (4.3 mg, 10 mol%). Then the vial was crimped. Solvent dry DMA (0.4 mL) was added to the mixture. The vial was degassed and refilled with nitrogen using the Schlenk-line technique (three times). and stirred at room temperature for 24 h under the irradiation of a single blue LED (455 (± 15) nm). 1,3-Dimethoxybenzene (0.2 mmol) was added as an internal standard., and yields are calculated by GC-FID analysis.

| Entry | Reaction component omitted | Product |
|---|---|---|
| 1 | Photocatalyst | 10% |
| 2 | $NiBr_2$·glyme | ND |
| 3 | dOMebpy | 33% |
| 4 | Light | ND |
| 5 | Without degassing | 37% |

### 3.3 Optimization of reaction for C-N/C-O bond forming reaction

- **Solvent Screening**

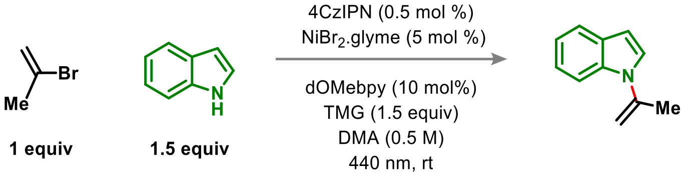


A 5 mL crimp top vial was charged with 2-bromoprop-1-ene (18.0 µL, 0.20 mmol, 1.0 equiv), the respective indole (35 mg, 0.30 mmol, 1.5 equiv), TMG (35 mg, 0.3 mmol, 1.5 equiv), 4CzIPN (0.8 mg, 0.5 mol%), $NiBr_2$·glyme (3.1 mg, 5.0 mol%), dOMebpy (4.3 mg, 10 mol%). Then the vial was crimped. Solvent dry DMA (0.4 mL) was added to the mixture. The vial was degassed and refilled with nitrogen using the Schlenk-line technique (three times). and stirred at room temperature for 24 h under the irradiation of a single blue LED (455 (± 15) nm). 1,3-Dimethoxybenzene (0.2 mmol) was added as an internal standard., and yields are calculated by GC-FID analysis.

| Entry | Solvent | Product |
|---|---|---|
| 1 | $CH_3CN$ | 50% |
| 2 | DMF | 45% |
| 3 | DMSO | 52% |
| **4** | **DMA** | **54%** |

- **Base Screening**

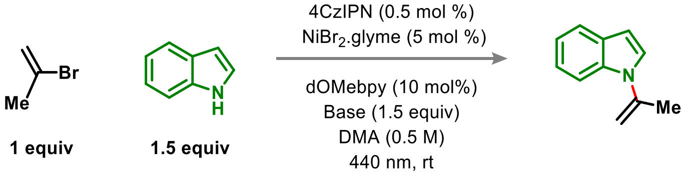


A 5 mL crimp top vial was charged with 2-bromoprop-1-ene (18.0 µL, 0.20 mmol, 1.0 equiv), the respective indole (35 mg, 0.30 mmol, 1.5 equiv), Base (0.3 mmol, 1.5 equiv), 4CzIPN (0.8 mg, 0.5 mol%), $NiBr_2$·glyme (3.1 mg, 5.0 mol%), dOMebpy (4.3 mg, 10 mol%). Then the vial was crimped. Solvent dry DMA (0.4 mL) was added to the mixture. The vial was degassed and refilled with nitrogen using the Schlenk-line technique (three times). and stirred at room temperature for 24 h under the irradiation of a single blue LED (455 (± 15) nm). 1,3-Dimethoxybenzene (0.2 mmol) was added as an internal standard., and yields are calculated by GC-FID analysis.

| Entry | Base | Product |
|---|---|---|
| 1 | None | ND |
| 2 | DABCO | 7% |
| 3 | TMG (1.5 equiv at a time) | 54% |
| 4 | TMG (Two times, 1.0 equiv each time) | 65 % |
| 5 | $^t$BuNH$_2$ | 31% |
| 6 | $^t$BuNH$_2$ ( 1.5 equiv) & $NEt_3$ (0.5 equiv) | 25% |

***NOTE:*** Added TMG (1.0 equiv) during the set upping the reaction and after 24h/48h (depending on vinyl bromide) adding another 1.0 equiv of TMG and continueing the reaction for another 24/48h (depending on vinyl bromide) gives the best yield.

- **Screening of stoichiometry of the reagents**

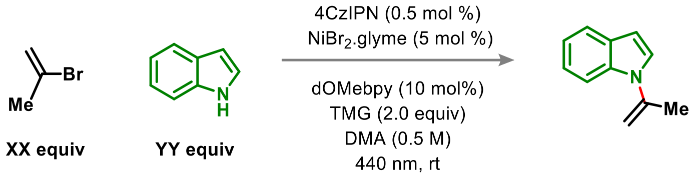


A 5 mL crimp top vial was charged with 2-bromoprop-1-ene (x.x equiv), the respective indole (y.y equiv), TMG (24 mg, 0.2 mmol, 1.0 equiv), 4CzIPN (0.8 mg, 0.5 mol%), $NiBr_2$·glyme (3.1 mg, 5 mol%), dOMebpy (4.3 mg, 10 mol%). Then the vial was crimped. Solvent dry DMA (0.4 mL) was added to the mixture .The vial was degassed and refilled with nitrogen using the Schlenk-line technique (three times). and stirred at room temperature under the irradiation of a single blue LED (455 (± 15) nm). After 24h, TMG (24 mg, 0.2 mmol, 1.0 equiv) was added and continued the reaction for another 24h. 1,3-Dimethoxybenzene (0.2 mmol) was added as an internal standard., and yields are calculated by GC-FID analysis.

| Entry | Vinyl bromide | Indole | Product |
|---|---|---|---|
| **1** | **1** | **1.5** | 65% |
| **2** | **1.5** | **1** | **71%** |
| **3** | **2.0** | **1** | 75% |
| **4** | **2.5** | **1** | 78% |
| **5** | **3.0** | **1** | 79% |

- **Control Experiments**

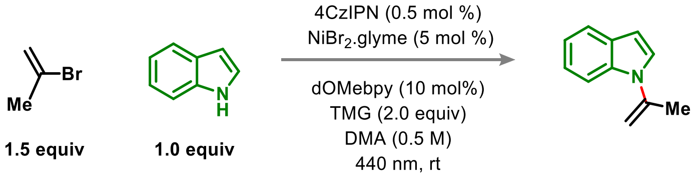


A 5 mL crimp top vial was charged with 2-bromoprop-1-ene (28 µL , 0.30 mmol 1.5 equiv), the respective indole (24 mg, 0.20 mmol, 1.0 equiv), TMG (24 mg, 0.20 mmol, 1.0 equiv), 4CzIPN (0.8 mg, 0.5 mol%), $NiBr_2$·glyme (3.1 mg, 5.0 mol%), dOMebpy (4.3 mg, 10 mol%). Then the vial was crimped. Solvent dry DMA (0.4 mL) was added to the mixture .The vial was degassed and refilled with nitrogen using the Schlenk-line technique (three times) and stirred at room temperature under the irradiation of a single blue LED (455 (± 15) nm). After 24h, TMG (24 mg, 0.2 mmol, 1.0 equiv) was added and continued the reaction for another 24h. 1,3-Dimethoxybenzene (0.2 mmol) was added as an internal standard., and yields are calculated by GC-FID analysis.

| Entry | Reaction component omitted | Product |
|---|---|---|
| 1 | Photocatalyst | ND |

| | | |
|---|---|---|
| 2 | $NiBr_2$·glyme | ND |
| 3 | dOMebpy | 11 % |
| 4 | Base | ND |
| 5 | Light | ND |
| 6 | Without degassing | 35% |

## 4. Naked-eye visual change in color/appearance upon addition of nucleophiles to a solution of $NiBr_2$·glyme in dimethylacetamide.

- **Class of nucleophiles where $^{t}BuNH_2$ gives the best yield**

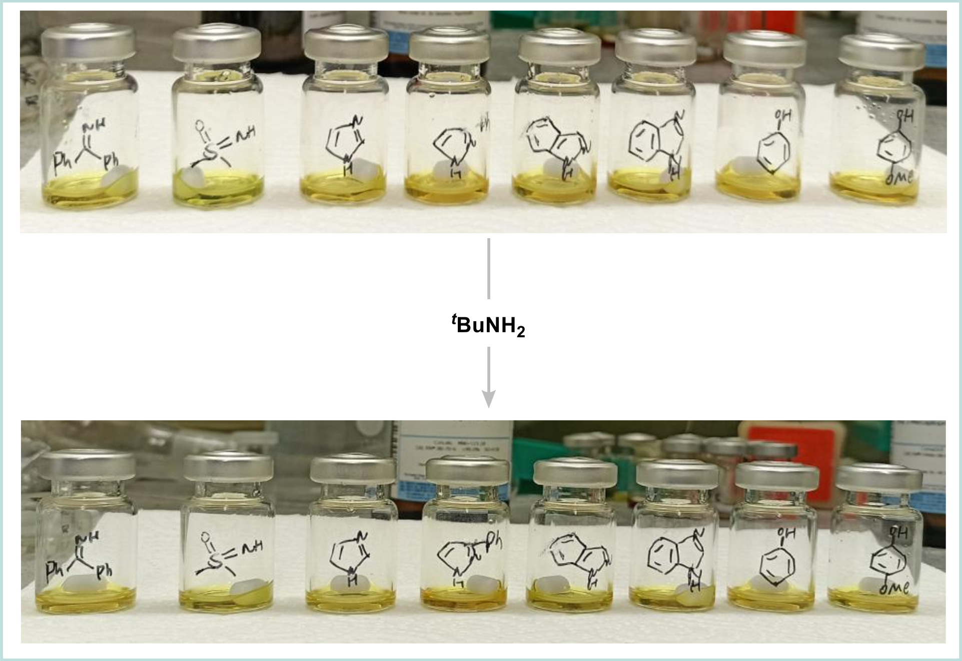


**Figure S3.** Changes in the visible appearance of a DMA solution of $NiBr_2$·glyme upon addition of various nucleophiles that require *tert*-butylamine as a base for effective formation of the desired product, in this case - from left to right - benzophenone imine, sulfoximine, imidazole, 3-phenyl-pyrazole, indazole, benzimidazole, phenol, and *e-rich*-phenol.

**Class of nucleophiles where TMG gives the best yield:**

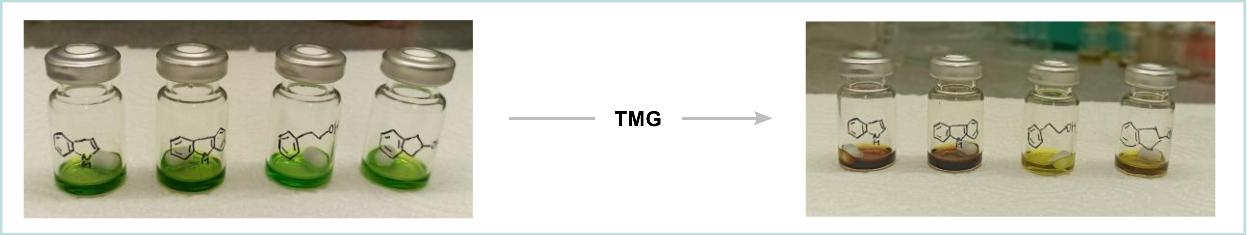


**Figure S4.** Changes in the visible appearance of a DMA solution of $NiBr_2$·glyme upon addition of various nucleophiles that require a strong base such as 1,1,3,3-tetramethylguanidine (TMG) for effective formation of the desired product, in this case - from left to right - indole, carbazole, primary aliphatic alcohol, and secondary aliphatic alcohol.

## 5. General Procedure for photochemical reactions

### 5.1. General Procedure I

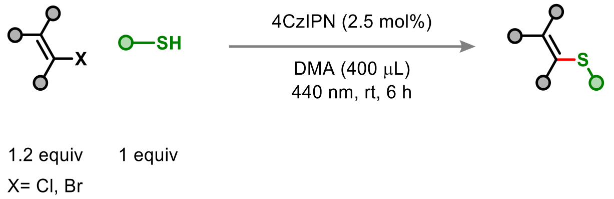


A 5 mL crimp-top vial was filled with vinyl halide (0.24 mmol, 1.2 equiv), the corresponding thiol (0.20 mmol, 1.0 equiv), 4CzIPN (2.5 mol%). The vial was then crimped. Solvent dry DMA (0.4 mL) was added to the mixture. The vial was degassed and refilled with nitrogen using the Schlenk line technique (three times). and stirred for 6 h at room temperature under irradiation of a single blue LED ($\lambda_{Max}$ = 455 ± 15 nm). The reaction mixture was quenched with $H_2O$ (15 mL). The resulting reaction mixture was extracted with EtOAc (2 × 15 mL). The combined organic phases were dried on $Na_2SO_4$. The solvent was removed by vacuum. Finally, the crude product  was purified by flash column chromatography using petrol ether/ethyl acetate or other suitable solvent mixtures (PE/DCM, DCM/MeOH, EtOAc/MeOH, wherever applicable) as eluents on silica gel.

### 5.2. General Procedure II

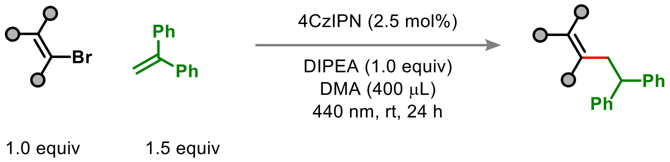


A 5 mL crimp-top vial was filled with vinyl halide (0.20 mmol, 1.0 equiv), the corresponding ethene-1,1-diyldibenzene (54 mg, 0.30 mmol, 1.5 equiv), DIPEA (39 mg, 0.30 mmol, 1.5 equiv), 4CzIPN (4.0 mg, 2.5 mol%). The vial was then crimped. Solvent dry DMA (0.4 mL) was added to the mixture. The vial was degassed and refilled with nitrogen using the Schlenk line technique (three times). and stirred for 24 h at room temperature under irradiation of a single blue LED ($\lambda_{Max}$ = 455 ± 15 nm). The reaction mixture was quenched with $H_2O$ (15 mL). The resulting reaction mixture was extracted with EtOAc (2 × 15 mL). The combined organic phases were dried on $Na_2SO_4$. The solvent was removed by vacuum. Finally, the crude product was purified by flash column chromatography using petrol ether/ethyl acetate or other suitable solvent mixtures (PE/DCM, DCM/MeOH, EtOAc/MeOH, wherever applicable) as eluents on silica gel.

## Other carbon nucleophiles as a trapping of vinyl radical:

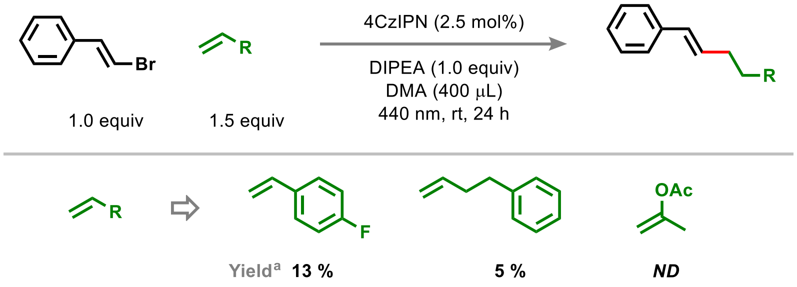


[a]Yields are calculated by GC-FID analysis

### 5.3. General Procedure III

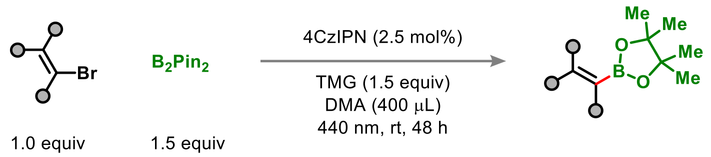


A 5 mL crimp-top vial was filled with vinyl halide (0.20 mmol, 1.0 equiv), $B_2Pin_2$ (76 mg, 0.30 mmol, 1.5 equiv), TMG (36 mg, 0.30 mmol, 1.5 equiv) and 4CzIPN (4.0 mg, 2.5 mol%). The vial was then crimped. Solvent dry DMA (0.4 mL) was added to the mixture. The vial was degassed and refilled with nitrogen using the Schlenk line technique (three times). and stirred for 48 h at room temperature under irradiation of a single blue LED ($\lambda_{Max}$ = 455 ± 15 nm). The reaction mixture was quenched with $H_2O$ (15 mL). The resulting reaction mixture was extracted with EtOAc (2 × 15 mL). The combined organic phases were dried on $Na_2SO_4$. The solvent was removed by vacuum. Finally, the crude product was purified by flash column chromatography using petrol ether/ethyl acetate or other suitable solvent mixtures (PE/DCM, DCM/MeOH, EtOAc/MeOH, wherever applicable) as eluents on silica gel.

### 5.4. General Procedure IV

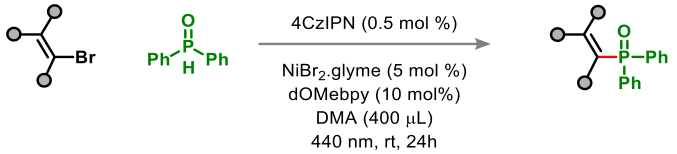


A 5 mL crimp-top vial was filled with vinyl halide (0.30 mmol, 1.5 equiv), the corresponding diphenylphosphine oxide (0.20 mmol, 1.0 equiv), 4CzIPN (0.5 mol%), $NiBr_2$-glyme (5 mol%), dOMebpy (10 mol%). The vial was then crimped. Solvent dry DMA (0.4 mL) was added to the mixture. The vial was degassed and refilled with nitrogen using the Schlenk line technique (three times). and stirred for 24 h at room temperature under irradiation of a single blue LED (455 (± 15) nm). The reaction mixture was quenched with $H_2O$ (15 mL). The resulting mixture was extracted with EtOAc (2 × 15 mL). The combined organic phases were dried over $Na_2SO_4$. The solvent was removed by vacuum. Finally, the crude product was purified by flash column chromatography using petrol ether/ethyl acetate or other suitable solvent mixtures (PE/DCM, DCM/MeOH, EtOAc/MeOH, wherever applicable) as eluents on silica gel.

### 5.5. General Procedure V

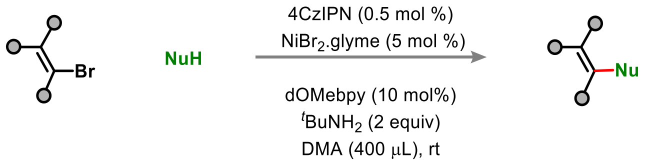


A 5 mL crimp-top vial was charged with vinyl halide (0.30 mmol, 1.5 equiv), the corresponding nucleophile (0.2 mmol, 1.0 equiv), 4CzIPN (0.5 mol%), $NiBr_2$-glyme (5 mol%), dOMebpy (10 mol%). The vial was then crimped. DMA (0.4 mL) was added to the vial. The vial was degassed and refilled with nitrogen using the Schlenk line technique (three times). ${}^tBuNH_2$ (0.4 mmol, 2.0 equiv) was added to the mixture and stirred at room temperature under irradiation of a single blue LED (455 (± 15) nm). After 48h/96h (depending on the reactivity of the vinyl bromide) the reaction mixture was quenched with $H_2O$ (15 mL). The resulting mixture was extracted with EtOAc (2 × 15 mL). The combined organic phases were dried over $Na_2SO_4$. The solvent was removed by vacuum. Finally, the crude product was purified by flash column chromatography using petrol ether/ethyl acetate or other suitable solvent mixtures (PE/DCM, DCM/MeOH, EtOAc/MeOH, wherever applicable) as eluents on silica gel.

## 5.6. General Procedure VI

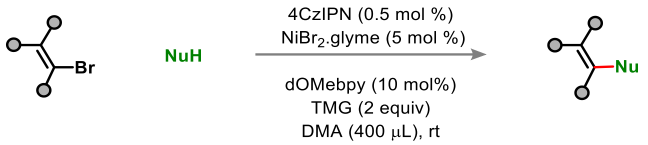


A 5 mL crimp-top vial was filled with vinyl halide (0.30 mmol, 1.5 equiv), the corresponding nucleophile (0.20 mmol, 1.0 equiv), TMG (0.20 mmol, 1.0 equiv), 4CzIPN (0.5 mol%), $NiBr_2$-glyme (5 mol%), dOMebpy (10 mol%). The vial was then crimped. DMA (0.4 mL) was added to the mixture. The vial was degassed and refilled with nitrogen using the Schlenk line technique (three times) and stirred at room temperature under irradiation of a single blue LED (455 (± 15) nm). After 24h/48h (depending on the reactivity of the vinyl bromide) TMG (0.2 mmol, 1.0 equiv) was added and the reaction continued for a further 24h/48h. The reaction mixture was quenched with $H_2O$ (15 mL). The resulting mixture was extracted with EtOAc (2 × 15 mL). The combined organic phases were dried over $Na_2SO_4$. The solvent was removed by vacuum. Finally, the crude product was purified by flash column chromatography using petrol ether/ethyl acetate or other suitable solvent mixtures (PE/DCM, DCM/MeOH, EtOAc/MeOH, wherever applicable) as eluents on silica gel.

## 5.7. General Procedure VII

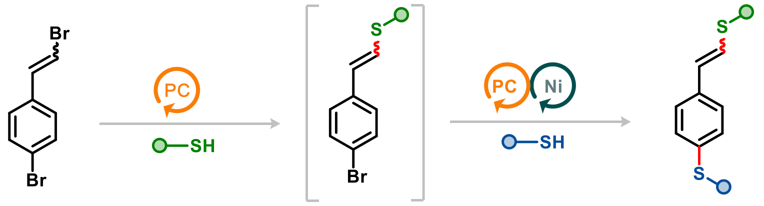


A 5 mL crimp-top vial was filled with vinyl halide (0.24 mmol, 1.2 equiv), the corresponding thiol (0.20 mmol, 1.0 equiv), and 4CzIPN (2.5 mol%). The vial was then crimped. Solvent dry DMA (0.4 mL) was added to the mixture. The vial was degassed and refilled with nitrogen using the Schlenk line technique (three times) and stirred at room temperature under irradiation of a single blue LED (455 (± 15) nm).
In the second step, the corresponding thiol (0.30 mmol, 1.5 eq.) and 0.1 mL of a catalyst stock solution containing 4CzIPN (0.8 mg, 0.5 mol%.) and $NiBr_2$-glyme (3.2 mg,5.0 mol%.) dissolved in DMA were added. The vial was degassed and refilled with nitrogen using the Schlenk line technique (three times). The mixture was stirred for 12 h at room temperature under irradiation of a single blue LED (455 (± 15) nm). The reaction mixture was quenched with $H_2O$ (15 mL). The resulting mixture was extracted with EtOAc (2 × 15 mL). The combined organic phases were dried over $Na_2SO_4$. The solvent was removed by vacuum. Finally, the crude product was purified by flash column chromatography using petrol ether/ethyl acetate or other suitable solvent mixtures (PE/DCM, DCM/MeOH, EtOAc/MeOH, wherever applicable) as eluents on silica gel.

## 5.8. General Procedure VIII

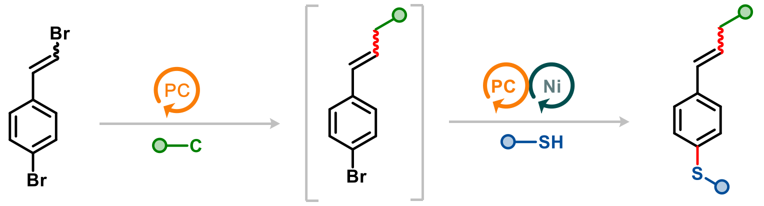


A 5 mL crimp-top vial was filled with vinyl halide (0.20 mmol, 1.0 equiv), the corresponding ethene-1,1-diyldibenzene (54 mg, 0.30 mmol, 1.5 equiv), DIPEA (39 mg, 0.30 mmol, 1.5 equiv) and 4CzIPN (2.5 mol%). The vial was then crimped. Solvent dry DMA (0.4 mL) was added to the mixture. The vial was degassed and refilled with nitrogen using the Schlenk line technique (three times) and stirred at room temperature under irradiation of a single blue LED (455 (± 15) nm).

In the second step, the corresponding thiol (0.30 mmol, 1.5 eq.) and 0.1 mL of a catalyst stock solution containing 4CzIPN (0.8 mg, 0.5 mol%.) and $NiBr_2$-glyme (3.2 mg,5.0 mol%.) dissolved in DMA were added. The vial was degassed and refilled with nitrogen using the Schlenk line technique (three times). The mixture was stirred for 12 h at room temperature under irradiation of a single blue LED (455 (± 15) nm). The reaction mixture was quenched with $H_2O$ (15 mL). The resulting mixture was extracted with EtOAc (2 × 15 mL). The combined organic phases were dried over $Na_2SO_4$. The solvent was removed by vacuum. Finally, the crude product was purified by flash column chromatography using petrol ether/ethyl acetate or other suitable solvent mixtures (PE/DCM, DCM/MeOH, EtOAc/MeOH, wherever applicable) as eluents on silica gel.

## 5.9. General Procedure IX

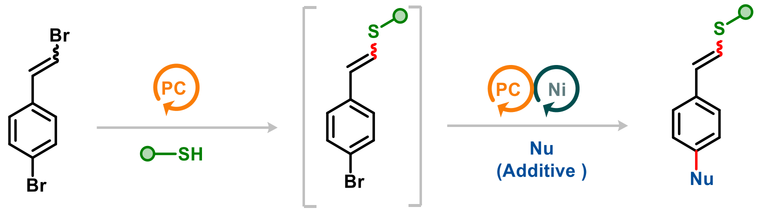


A 5 mL crimp-top vial was filled with vinyl halide (0.24 mmol, 1.2 equiv), the corresponding thiol (0.20 mmol, 1.0 equiv) and 4CzIPN (2.5 mol%). The vial was then crimped. Solvent (0.4 mL) was added to the mixture. The vial was degassed and refilled with nitrogen using the Schlenk line technique (three times) and stirred for 12 h at room temperature under irradiation of a single blue LED (455 (± 15) nm).
In the second step, the respective nucleophile (0.30 mmol, 1.5 equiv), $^{t}BuNH_2$ (0.3 mmol, 1.5 equiv), dOMebpy (10 mol%) and 0.1 mL of a catalyst stock solution containing 4CzIPN (0.8 mg, 0.5 mol%) and $NiBr_2$-glyme (3.2 mg,5.0 mol%) dissolved in DMA were added. The vial was degassed and refilled with nitrogen using the Schlenk-line technique (three times). The mixture was stirred for 24 h at room temperature under the irradiation of a single blue LED (455 (± 15) nm). The reaction mixture was quenched with $H_2O$ (15 mL). The resulting mixture was extracted with EtOAc (2 × 15 mL). The combined organic phases were dried over $Na_2SO_4$. The solvent was removed by vacuum. Finally, the crude product was purified by flash column chromatography using petrol ether/ethyl acetate or other suitable solvent mixtures (PE/DCM, DCM/MeOH, EtOAc/MeOH, wherever applicable) as eluents on silica gel.
***Note:*** For diphenylphosphine oxide, we don't need to add any base.
For C–N cross-coupling is performed in the second step, near quantitative conversion of thiol needs to be achieved. Running the first step longer as reported has not been associated with noticeable catalyst deactivation. However, running the first step for shorter reaction times can lead to catalyst inhibition. This is possibly due to the formation of inactive thiolate complexes in the presence of bases. For the same reason, the initial addition of too much thiol can be problematic as well.

## 5.10. General Procedure X

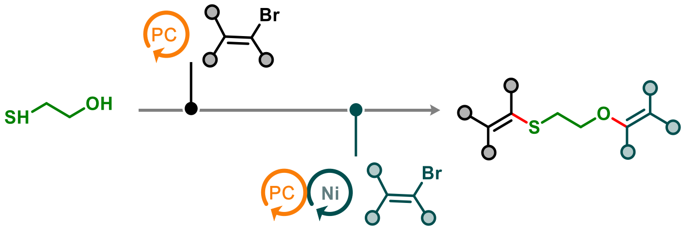


A 5 mL crimp-top vial was filled with vinyl halide (0.24 mmol, 1.2 equiv), the corresponding 2-mercaptoethan-1-ol (0.20 mmol, 1.0 equiv) and 4CzIPN (2.5 mol%). The vial was then crimped. Solvent (0.4 mL) was added to the mixture. The vial was degassed and refilled with nitrogen using the Schlenk line

technique (three times) and stirred for 12 h at room temperature under irradiation of a single blue LED (455 (± 15) nm).
In the second step, the respective vinyl halide (0.30 mmol, 1.5 equiv), TMG (0.2 mmol, 1.0 equiv), dOMebpy (10 mol%) and 0.1 mL of a catalyst stock solution containing 4CzIPN (0.8 mg, 0.5 mol%) and $NiBr_2$-glyme (3.2 mg,5.0 mol%) dissolved in DMA were added. The vial was degassed and refilled with nitrogen using the Schlenk-line technique (three times). The mixture was stirred at room temperature under the irradiation of a single blue LED (455 (± 15) nm). After 24h/48h (depending on the reactivity of the vinyl bromide) TMG (0.2 mmol, 1.0 equiv) was added and the reaction continued for a further 24h/48h. The reaction mixture was quenched with $H_2O$ (15 mL). The resulting mixture was extracted with EtOAc (2 × 15 mL). The combined organic phases were dried over $Na_2SO_4$. The solvent was removed by vacuum. Finally, the crude product was purified by flash column chromatography using petrol ether/ethyl acetate or other suitable solvent mixtures (PE/DCM, DCM/MeOH, EtOAc/MeOH, wherever applicable) as eluents on silica gel.

## 5.11. Procedure for larger scale synthesis:

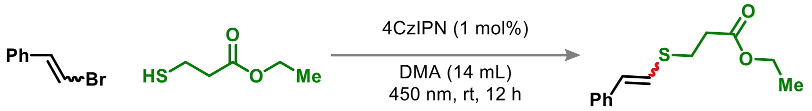


A 20 mL crimp top vial was charged with (2-bromovinyl)benzene (1.26 mL, 9.60 mmol, 1.20 equiv), ethyl 3-mercaptopropanoate (1.01 mL, 8.00 mmol, 1.00 equiv) and 4CzIPN (64 mg, 1 mol%). Then the vial was crimped. Solvent dry DMA (14.0 mL) was added to the mixture. The vial was degassed and refilled with nitrogen using the Schlenk-line technique (three times). and stirred at room temperature for 12h under the irradiation of a single blue LED (455 (± 15) nm, HP, 1294 mW). The reaction mixture was quenched with $H_2O$ (100 mL). The resulting reaction mixture was extracted with EtOAc (2 × 80 mL). The combined organic phases were dried on $Na_2SO_4$. The solvent was removed by vacuum. Finally, the crude product was purified by flash column chromatography using petrol ether/ethyl acetate or other suitable solvent mixtures (PE/EtOAc) as eluents on silica gel to afford the product **5** as colourless oil (1.47 g, 77%).

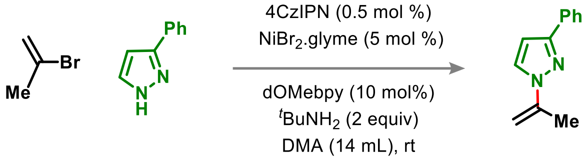


A 20 mL crimp-top vial was charged with 2-bromoprop-1-ene (1.10 mL, 12.0 mmol, 1.50 equiv), the corresponding 3-phenyl-1*H*-pyrazole (1.15 g, 8.0 mmol, 1.0 equiv), 4CzIPN (32 mg, 0.5 mol%), $NiBr_2$-glyme (128 mg, 5.00 mol%), dOMebpy (172 mg, 10.0 mol%). The vial was then crimped. DMA (14 mL) was added to the vial. The vial was degassed and refilled with nitrogen using the Schlenk line technique (three times). $^t$BuNH$_2$ (0.4 mmol, 2.0 equiv) was added to the mixture and stirred at room temperature under irradiation of a single blue LED (455 (± 15) nm, HP, 1294 mW)). After 48h The reaction mixture was quenched with $H_2O$ (100 mL). The resulting reaction mixture was extracted with EtOAc (2 × 80 mL). The combined organic phases were dried on $Na_2SO_4$. The solvent was removed by vacuum. Finally, the crude product was purified by flash column chromatography using petrol ether/ethyl acetate or other suitable solvent mixtures (PE/EtOAc) as eluents on silica gel to afford the product **36** as colourless oil (696 mg, 47%).

## 5.12 Unsuccessful Substrate:

The following substrates were found to be unsuccessful under our current reaction conditions and remain inaccessible.

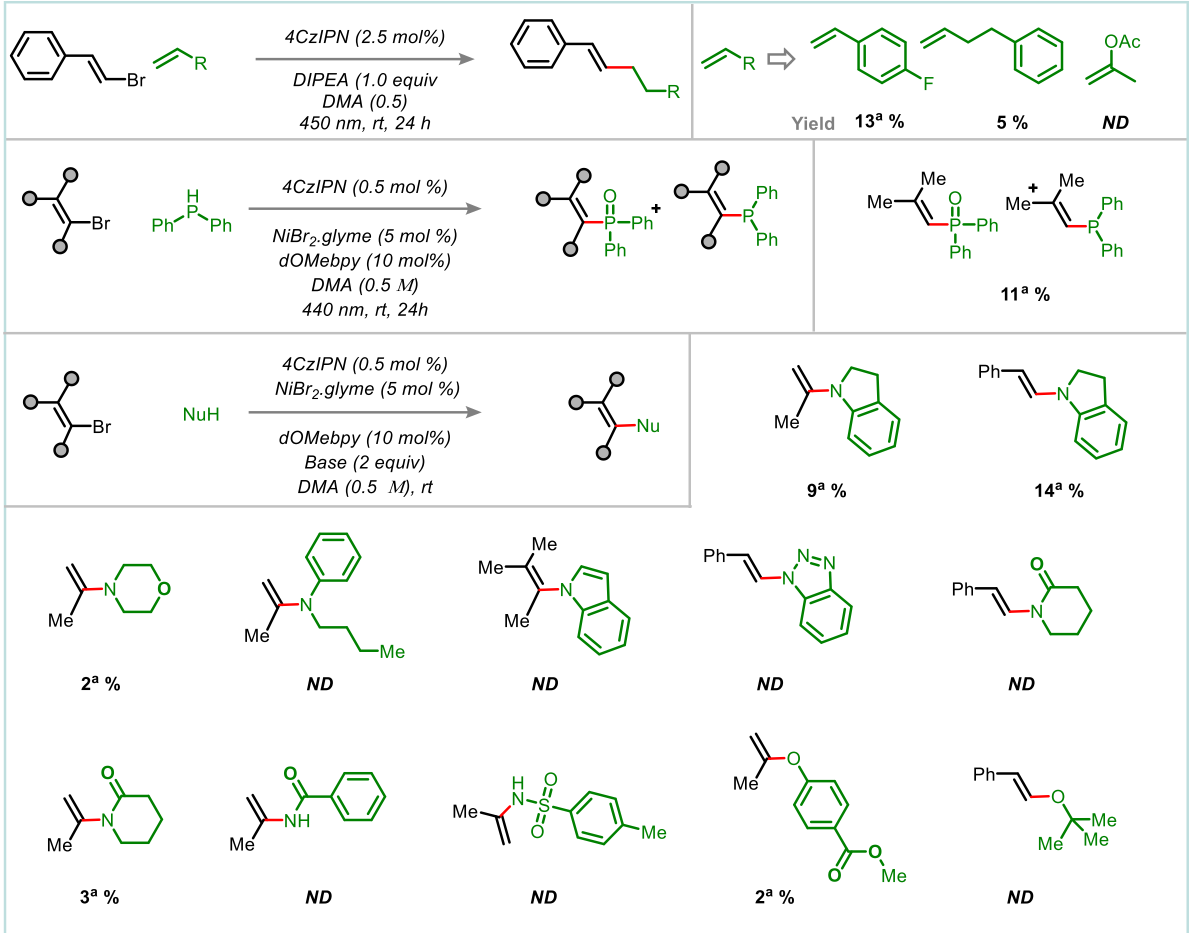


a Yields are calculated by GC-FID analysis using 1,3-dimethoxybenzene as an internal standard

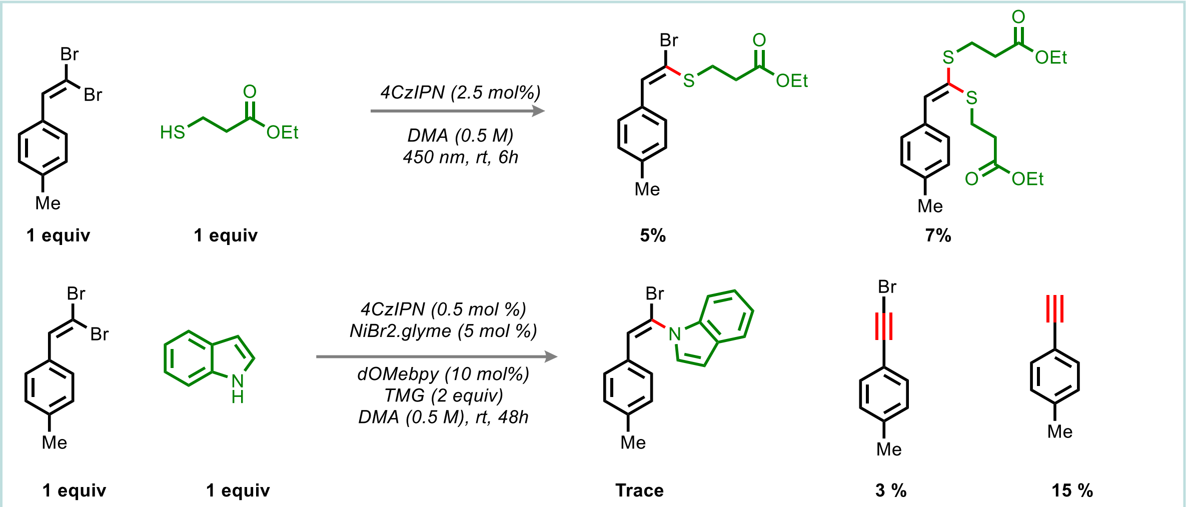


Yields are calculated by GC-FID analysis using 1,3-dimethoxybenzene as an internal standard

## 6. Results from spectroscopic investigations and Control Experiments:

### 6.1. Cyclic voltammetry (CV) measurements

CVs of vinyl bromides were measured by using tetrabutylammonium tetrafluoroborate (0.15 M) as a supporting electrolyte and ferrocene as an internal standard in $CH_3CN$ solvent under argon atmosphere in glassy carbon (GC) electrode with 50 mV/Sec scan rate.

Cyclic Voltammetry of 2-bromovinylbenzene (with ferrocene as a reference)

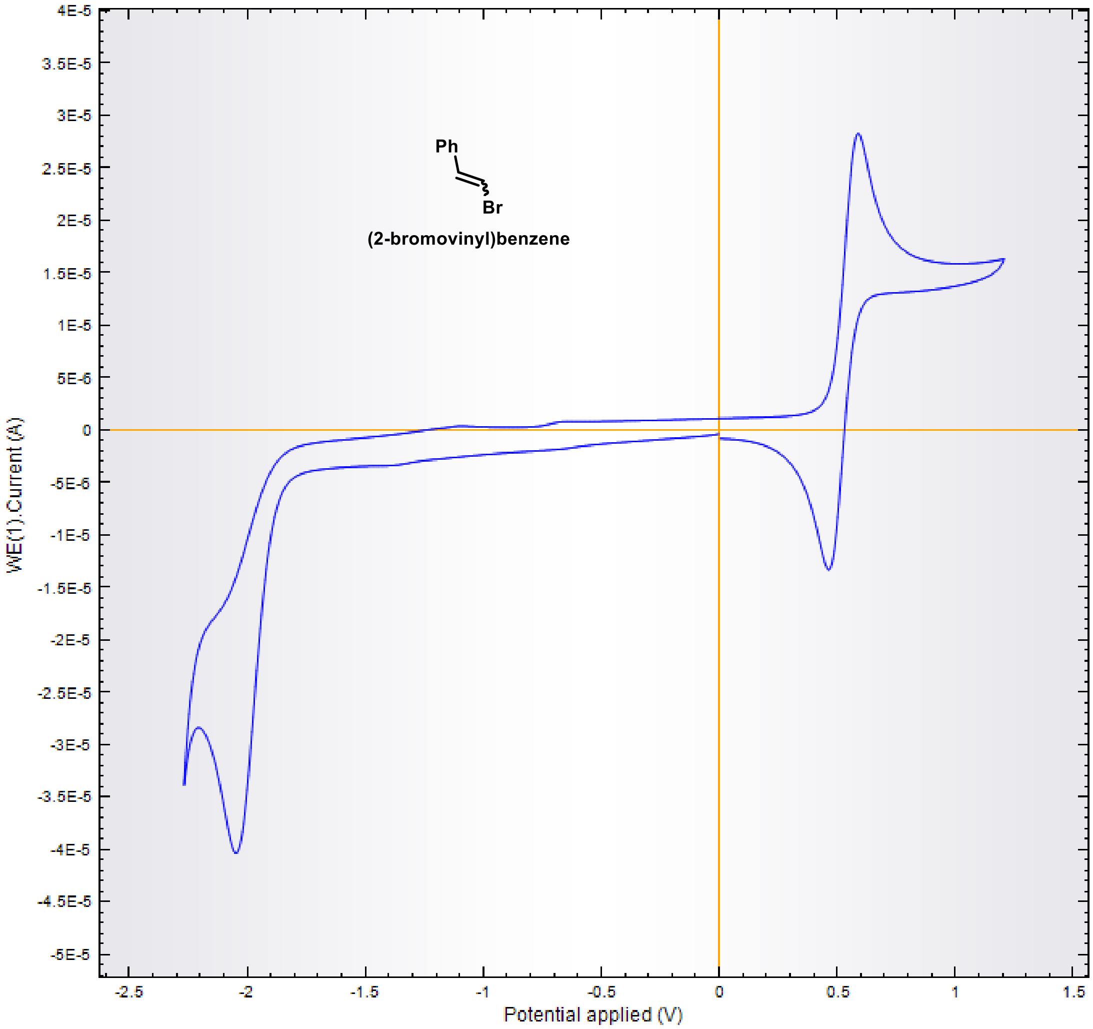


**-2.18 V vs SCE**

Cyclic Voltammetry of 2-bromo-propene (with ferrocene as a reference)

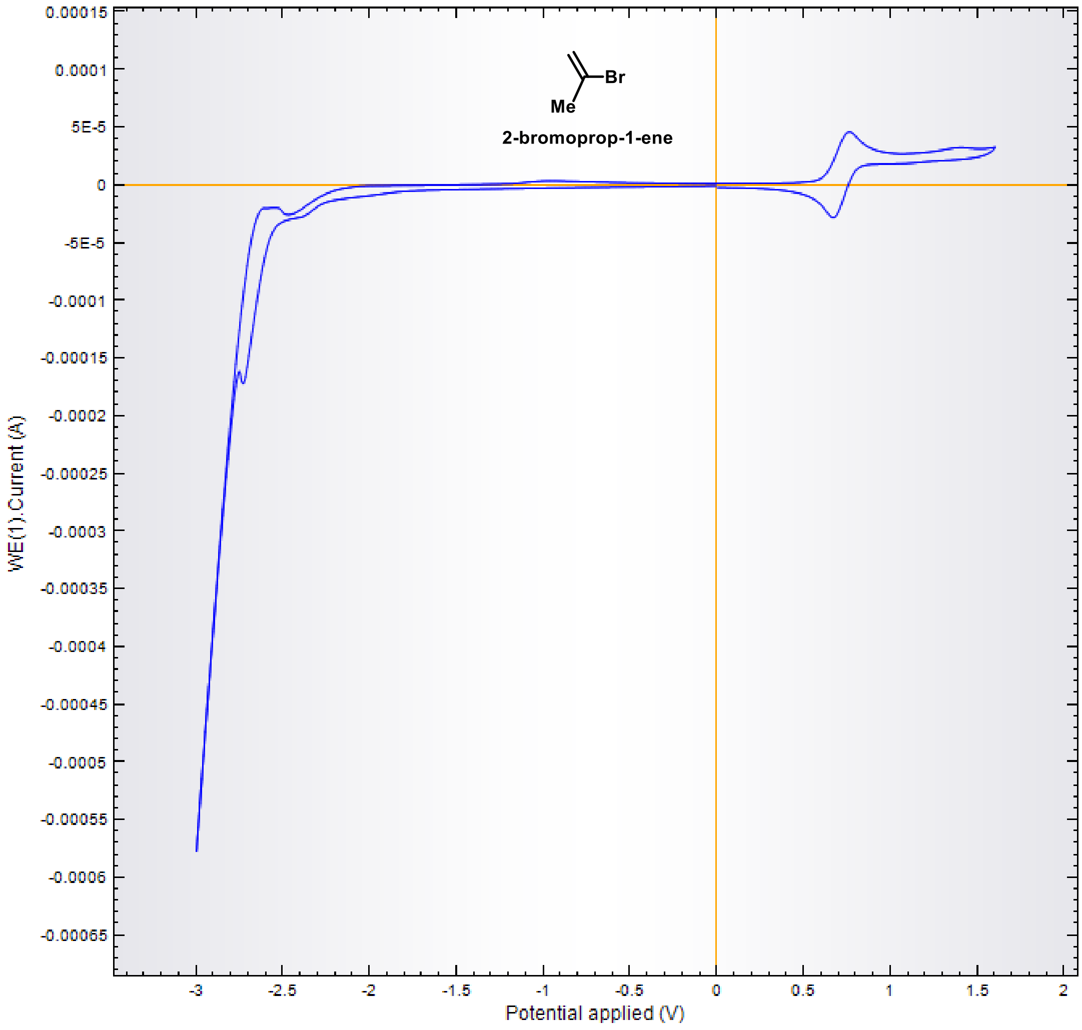


**-2.56 V vs SCE**

Cyclic Voltammetry of 1-bromo 2-methyl prop 1-ene (with ferrocene as a reference)

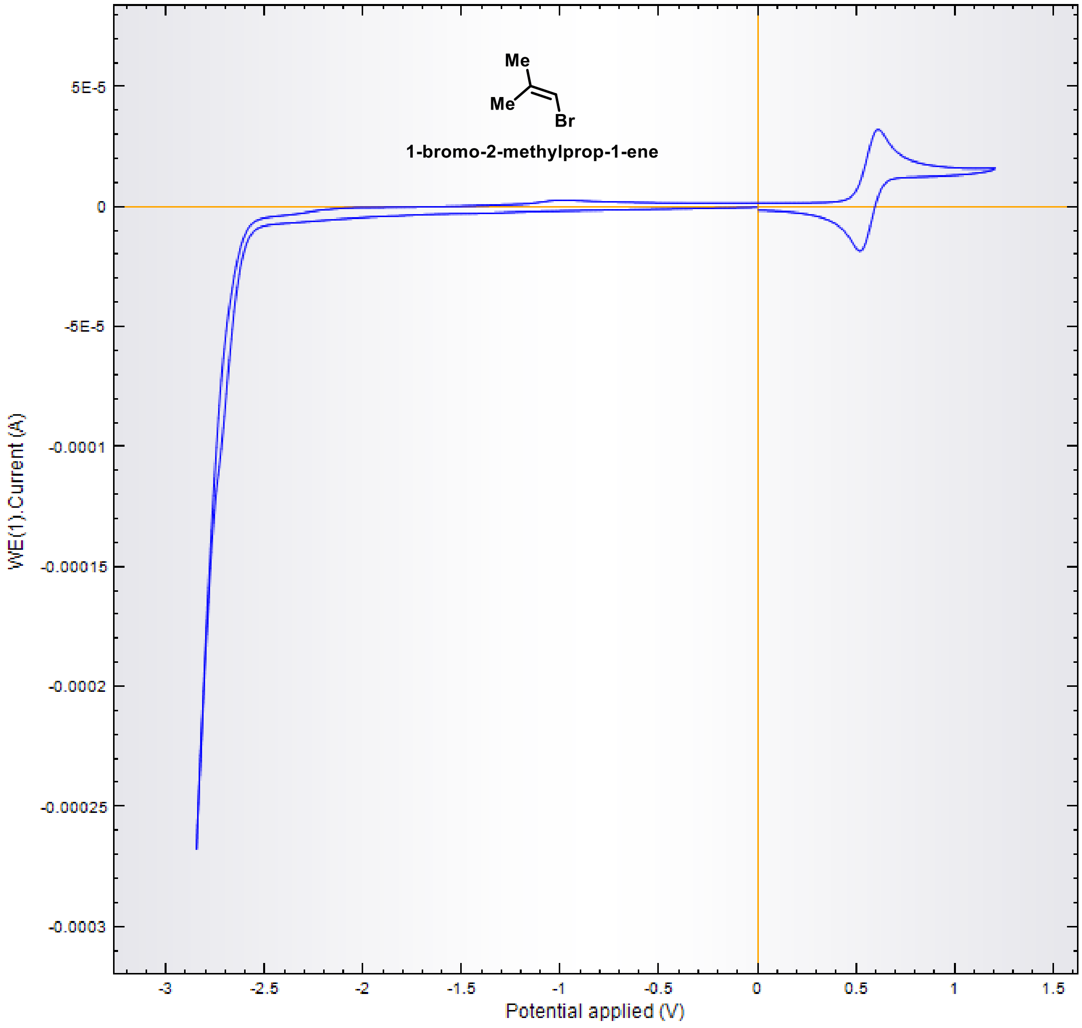


**>-2.60 V vs SCE**

Cyclic Voltammetry of 2-bromo-3-methyl-2-butene (with ferrocene as a reference)

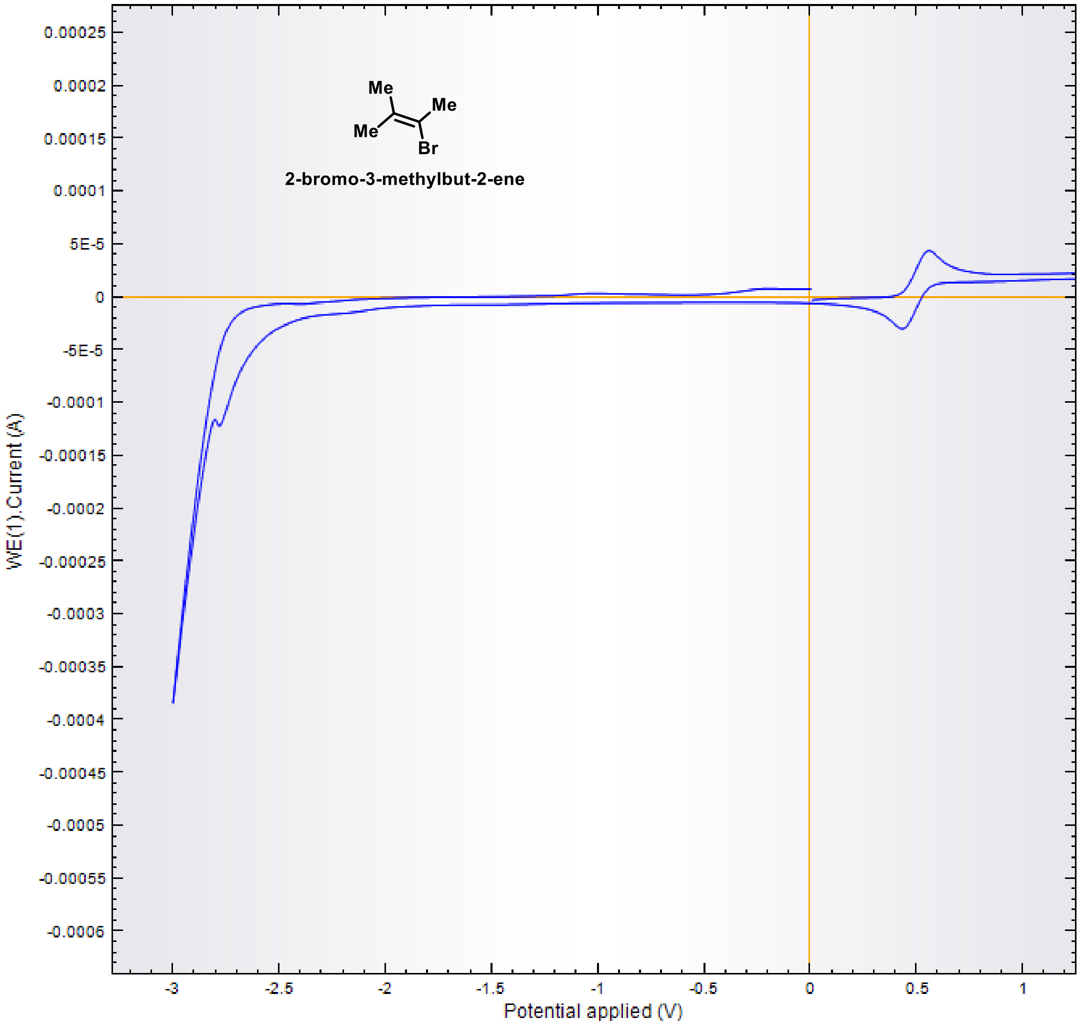


**-2.68 V vs SCE**

### 6.2. UV-Vis experiments:

The measurements were carried out by dissolving the corresponding thiol (1a), vinyl bromide (2a) in DMA at [1 mM] and 4CzIPN (PC) in DMA at [50 µM] concentration. No shift is observed for the mixture of 1a + 2a in 1:1 (v/v), which supports the requirement of photocatalyst.

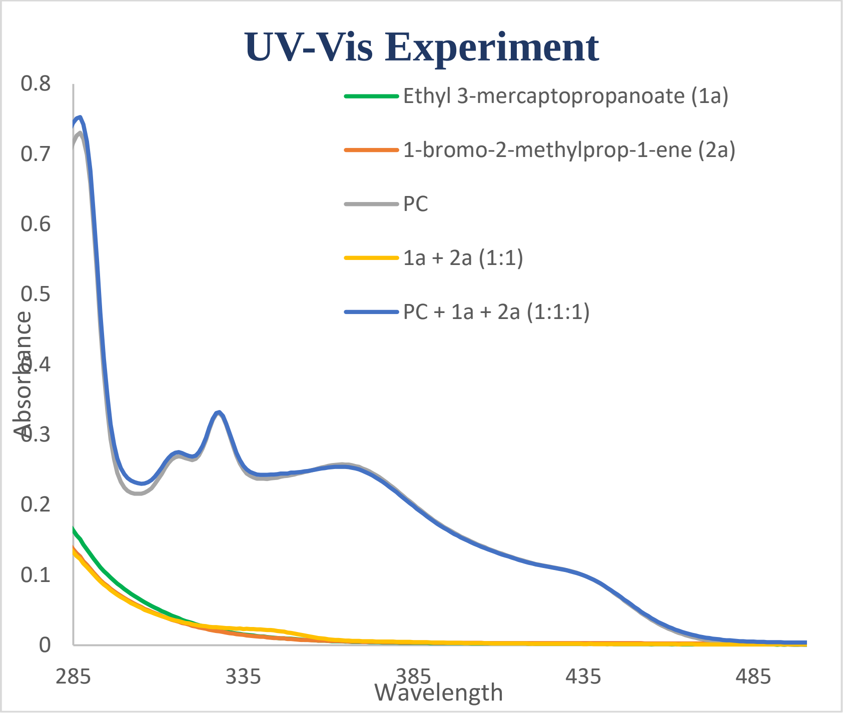

**6.3.Stern Volmer Analysis:**

Photoluminescence (both steady-state and time-resolved) quenching experiment was performed by dissolving the 4CzIPN (PC) in DMA at [1 µM] concentration and corresponding (2-bromovinyl)benzene (vinyl bromide) , ethyl 3-mercaptopropanoate (thiol), tetrabutylammonium bromide (**TBAB**) in DMA at [0.1 M]. The photoluminescence and the lifetime spectral data were collected by increasing amount of substates added in order to study the changing of the quencher concentration vs the $I_o/I$ and $\tau_o/\tau$.

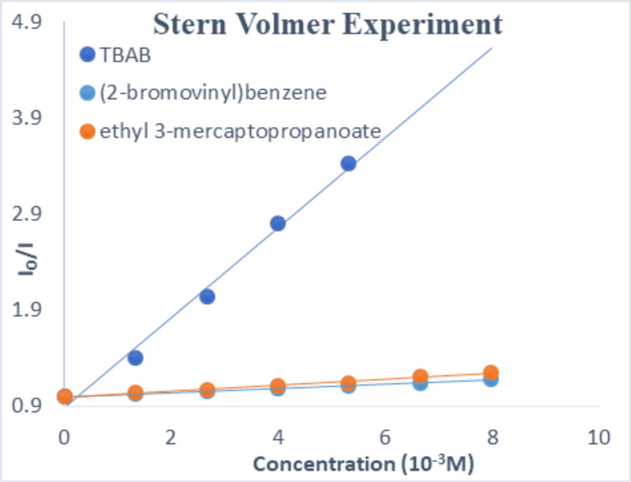


**Figure S5.** Luminescence quenching studies of 4CzIPN with vinyl bromide, thiol, and tetrabutylammonium bromide (TBAB).

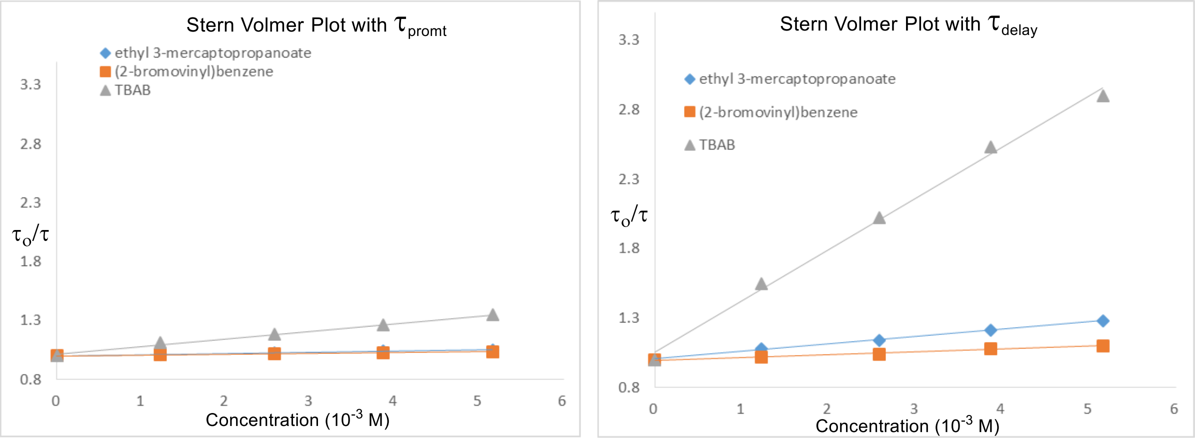


**Figure S6.** Time-resolved quenching studies of 4CzIPN with vinyl bromide, thiol, and tetrabutylammonium bromide (TBAB).

## 6.4. Control Experiments:

The experiment was carried out according to General Procedure **II** using vinyl bromide (1.0 equiv), DIPEA (1.5 equiv), and 4CzIPN (2.5 mol %) in DMA as the solvent, and the mixture was stirred for 12 hours. The reduction of vinyl bromide and the formation of products **92** and **93** were confirmed by HRMS as well as GC-MS.

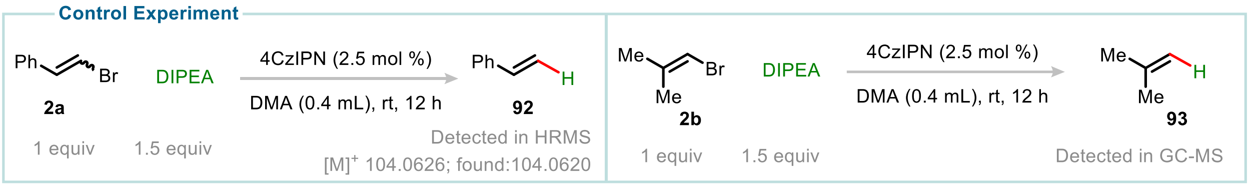


### Radical Clock Experiment:

According to General Procedure **II**, vinyl bromide (**2a**, 1.0 equiv), DIPEA (1.5 equiv), β-pinene (**3a**, 1.5 equiv), and 4CzIPN (2.5 mol %) were combined in DMA as the solvent. After stirring for 12 hours, the reduction product **92**, derived from the corresponding vinyl bromide, as well as the ring-opening product **94**, were confirmed by HRMS.

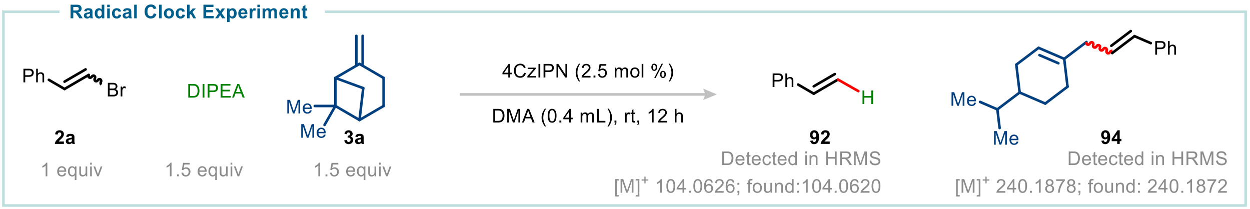


### Radical Trapping Experiment:

The reaction was conducted using vinyl bromide (**2a**, 1.0 equiv) and either triethyl phosphite or a thiol (**3a**, 1.5 equiv), in the presence of DMPO (5,5-dimethyl-1-pyrroline-N-oxide) or TEMPO (3.0 equiv), respectively, along with 4CzIPN (2.5 mol %) in DMA as the solvent. The reaction was completely quenched, and the DMPO-adduct product **95** was confirmed and characterized by HRMS.

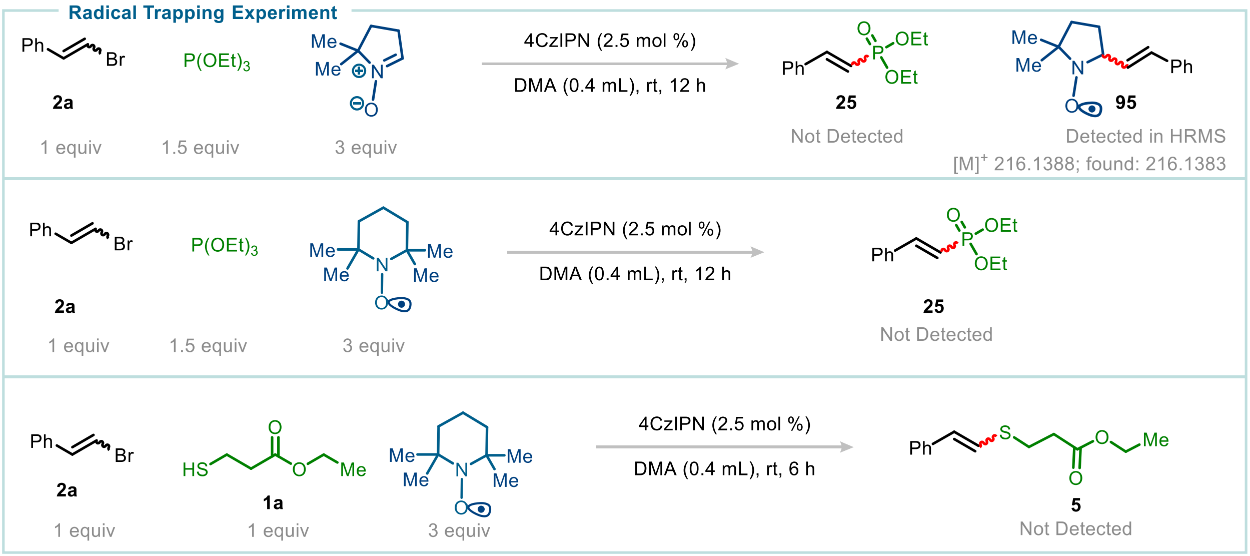

## 7. Possible mechanism/s for the formation of the desired product

### 7.1. Photoredox catalysis for the coupling reactions in the presence of only photocatalyst

Although a comprehensive mechanistic understanding of these transformations remains to be fully elucidated, experimental evidence strongly suggests a photochemical nature for the reported reaction (cf., control reactions in Section 3). In cases involving only a photocatalyst (i.e., without a metal catalyst, as in $C(sp^2)$–S transformations), multiple mechanistic pathways are conceivable. These pathways are influenced by factors such as the nature of the electrophiles, including their redox potentials, $C(sp^2)$–Br/Cl bond dissociation energies, and bond dissociation kinetics. Additionally, forming different bonds with various nucleophiles, each with distinct redox properties, introduces subtle but significant variations under photochemical reaction conditions. Below, we discuss a mechanistic model for $C(sp^2)$–S bond formation using (2-bromovinyl)benzene and ethyl 3-mercaptopropanoate as representative vinyl bromide electrophiles and thiols, respectively.

Initial insights were gained through photoluminescence (both steady-state and time-resolved) quenching studies, which probed the interaction between the excited-state photocatalyst and various reaction components. The organic photocatalyst 4CzIPN, which absorbs visible light, has excited-state redox potentials of $E_{1/2}(PC^*/PC^{\bullet-}) = +1.4$ V vs. SCE and $E_{1/2}(PC^{\bullet+}/PC^*) = -1.2$ V vs. SCE[2]. Upon addition of 2-bromovinylbenzene, only a very slight change in the steady-state photoluminescence and excited–state lifetime of 4CzIPN was observed. This quenching effect was more pronounced with ethyl 3-mercaptopropanoate but significantly increased in the presence of tetrabutylammonium bromide (TBAB), a representative soluble bromide salt (see section 6.3 Stern Volmer Analysis). A clear linear Stern–Volmer relationship was observed in the latter case, indicating effective quenching.

To determine whether the reduced photocatalyst in the ground state is capable of directly reducing the vinyl bromide, cyclic voltammetry (CV) experiments were carried out (see Section 6.1 CV measurement). The reduction potentials of vinyl bromides were found to be more negative[3] than that of the reduced ground-state 4CzIPN, suggesting that a direct single-electron transfer from the reduced photocatalyst to the vinyl halide is unlikely. Taking these observations into account, we propose the following plausible mechanism for the thiol coupling reactions.

1. In the first scenario, The photochemical transformation is initiated by the interaction of the thiol with the excited-state photocatalyst, generating a thiyl radical (II) via oxidation followed by proton loss. This thiyl radical (II) then adds to the vinyl bromide, forming intermediate III, which can be reduced by the ground-state reduced photocatalyst. Debromination then furnishes the desired product. As the reaction proceeds, bromide anions accumulate in the reaction medium, potentially altering the mechanistic pathway. In this scenario, bromide ions may undergo oxidation to generate bromine radicals (Br•)[4], which can abstract a hydrogen atom via hydrogen atom transfer (HAT), thereby regenerating the thiyl radical (II). The reaction then proceeds as before.

2. In an alternative scenario: We propose that the vinyl radical (IV) is generated in our reaction condition from the corresponding vinyl bromide, which can combine with the thiol radical (II) to produce the desired product (V). It is also worth noting that recent studies have demonstrated that the excited-state radical anion of 4CzIPN is a highly potent reductant[5,6]. The formation of this species in the reaction mixture under photoredox catalytic conditions can facilitate the reduction of organic halides with very high (negative) reduction potentials. For instance, when compounds **2a** and **2b** were photoirradiated in the presence of 4CzIPN and *N*,*N*-diisopropylethylamine (DIPEA), the respective reduction products (**92** and **93**) were detected by HRMS and GC-MS analysis (see section 6.4, control experiments). Additionally, radical trapping experiments led to the detection of species **95** via HRMS, supporting the involvement of vinyl radical in the formation of the desired product.

The reaction may proceed through one or a combination of these pathways, depending on the specific combination of electrophile (vinyl bromide) and nucleophile (thiol). The faster quenching of the

photocatalyst by the bromide anion compared to thiol and vinyl bromide suggests that mechanisms involving bromide radicals may play a significant role, although all two pathways could operate simultaneously under varying conditions or during the progression of the reaction.
In the cross-coupling process with other coupling partners—such as $C(sp^2)–C(sp^3)$ bond formation, which is also effective for $C(sp^2)–B$ bond formation using $B_2Pin_2$ and $C(sp^2)–P$ bond formation using triethyl phosphite—the mechanistic pathways are most likely to be the generation of vinyl bromide (IV) under photochemical condition. This intermediate is then intercepted by nucleophiles, followed by oxidation to furnish the desired product.
At this stage, it is challenging to pinpoint a single mechanism responsible for the formation of the desired product. Multiple mechanisms are likely involved, with their relative contributions changing throughout the reaction (e.g., the involvement of $Br^–$ at a later stage in $C(sp^2)$-$C(sp^3)$/B/P bond-forming reactions). Additionally, the dominant mechanism may vary depending on the specific combination of electrophiles and nucleophiles, as their redox potentials and quenching abilities can influence the photoredox process.
We continue to investigate these mechanisms in greater detail to deepen our understanding of the process, which may guide the development of new chemical reactions involving vinyl bromides.

*Note:* The results of the luminescence and lifetime quenching experiments reveal only a slight decrease upon addition of 5 mM (2-bromovinyl)benzene) in both the emission intensity and excited-state lifetime. The reason for this relatively small change is not entirely clear at this stage. One possible explanation could be the minor incorporation of air during the successive addition of the quencher during measurements. Notably, we rule out the possibility of direct energy transfer to the vinyl bromide, as such a process would likely lead to a more substantial decrease in lifetime—particularly in the delayed fluorescence component—as observed in analogous systems[7]. That said, the full photochemical reaction mixture includes other components (e.g., the thiol and, subsequently, $Br^–$ in the case of photoredox-catalyzed $C(sp^2)–S$ bond-forming reactions), which interact more effectively with the excited state of 4CzIPN and contribute significantly to the overall quenching behaviour.

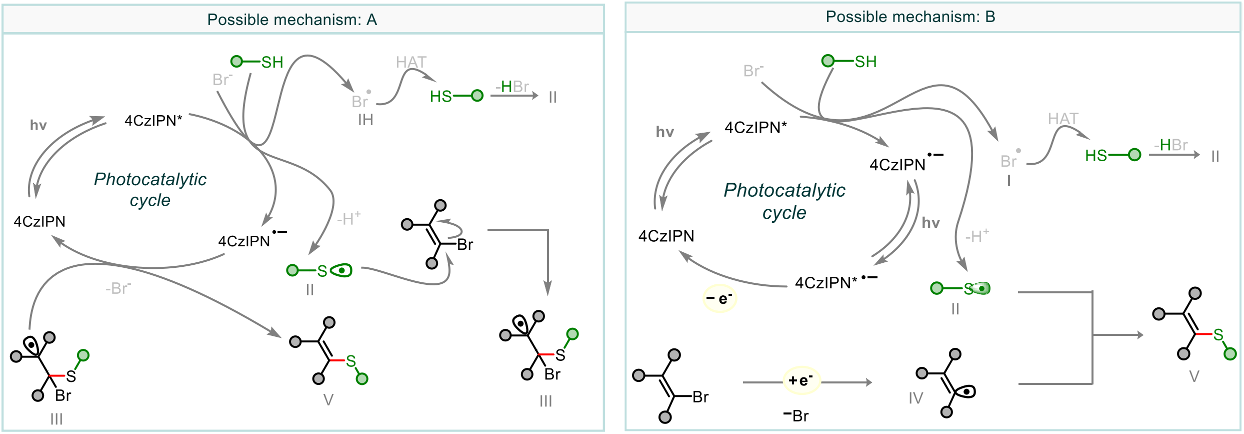


## 7.2. Photoredox Nickel dual catalytic system

Similar to the photoredox process, two mechanistic pathways can be envisioned for nickel-mediated cross-coupling reactions under photoredox conditions: a Ni(0)/Ni(II)/Ni(III) pathway and a Ni(I)/Ni(III) pathway. In the former, the ligated Ni(II) species is reduced under photoredox conditions to generate a Ni(0) complex. This Ni(0) species undergoes oxidative addition with the vinyl halide to form a Ni(II) intermediate. Subsequent single-electron oxidation by the excited state of photocatalyst, converts this Ni(II) intermediate into a Ni(III) species. Ligand exchange (in this case, $Br^–$ is being replaced by the nucleophile), followed by reductive elimination from the Ni(III) species, yields the cross-coupled product and forms a Ni(I) species. This Ni(I) species can be further reduced by the reduced state of photocatalyst to regenerate Ni(0), thereby completing the catalytic cycle, while simultaneously regenerating the ground-state photocatalyst. Notably,

depending on the nucleophile, a nucleophile-ligated nickel species —coordinated either with the nucleophile itself or its corresponding anion—may form prior to oxidative addition. This effect is supported by a noticeable color change observed upon the addition of TMG to the reaction mixture (see Section 4 in the Supporting Information), suggesting a potential role for TMG in facilitating oxidative addition.

In the Ni(I)/Ni(III) pathway, Ni(II) is initially reduced to Ni(I) by the photocatalyst. The resulting Ni(I) species undergoes oxidative addition with the vinyl halide to form a Ni(III) intermediate, which then undergoes reductive elimination to yield the final product and regenerate the Ni(I) species. This cycle can be self-sustaining, and its efficiency may depend on the specific combination of nucleophile and electrophile used.

It is important to note that Ni(0)/Ni(II)/Ni(III) cycles typically require stabilizing ligands[8] (e.g., bipyridine) to form and maintain the Ni(0) species, whereas Ni(I)/Ni(III) pathways can operate in both the presence and absence of such ligands.[9] In our system, photochemical reactions proceed under both conditions (*cf.*see section 3 in the supporting information); however, the presence of bipyridine ligands significantly enhances product yields. We therefore believe that both catalytic pathways may be operative under our reaction conditions. The extent to which each pathway contributes likely depends on the specific substrate combination and the conditions used for a given cross-coupling reaction

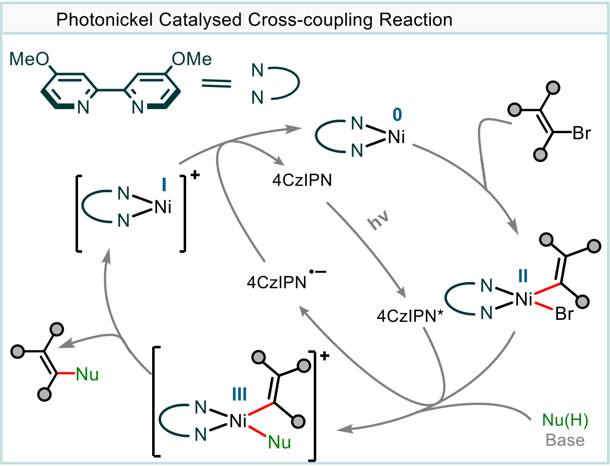


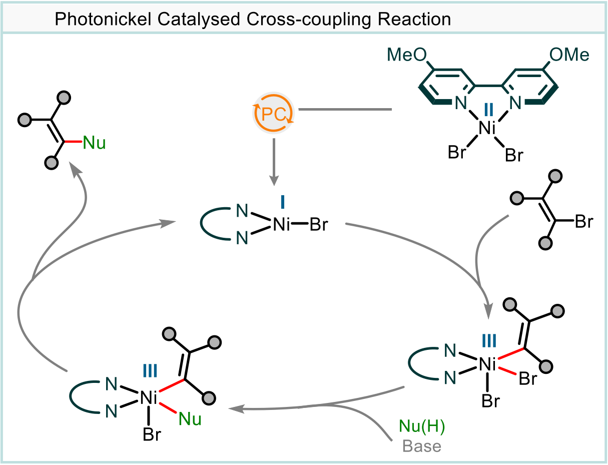

## 8. Characterization data of the final product

**Dodecyl(2-methylprop-1-en-1-yl)sulfane (1)**

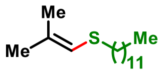


**1**

Following general procedure **I**, 1-bromo-2-methylprop-1-ene (24 µL, 0.24 mmol, 1.2 equiv), dodecane-1-thiol (48 µL, 0.20 mmol, 1.0 equiv), and 4CzIPN (4.0 mg, 2.5 mol%), were stirred for 6 h. After the workup, the organic phases were concentrated under vacuum. The crude product was purified by flash chromatography using silica, EtOAc:Hexane to afford the product **1** as colourless oil (74 mg, 72%).

- **$^{1}$H NMR (400 MHz, $CDCl_3$):** δ 5.60 (t, *J* = 1.3 Hz, 1H), 2.64 – 2.48 (m, 2H), 1.75 (dd, *J* = 12.4, 1.2 Hz, 6H), 1.58 (q, *J* = 7.6 Hz, 2H), 1.32 – 1.16 (m, 18H), 0.97 – 0.79 (m, 3H).
- **$^{13}$C NMR (101 MHz, $CDCl_3$):** δ 133.8, 118.2, 39.2, 34.0, 31.9, 30.3, 29.7, 29.6, 29.4, 29.3, 29.2, 28.7, 25.2, 22.7, 19.6, 14.1.
- **HRMS (ESI):** calcd. for $C_{16}H_{32}S^+$ [$M^+$] 256.2225; found = 256.2222.

**Ethyl 3-((2-methylprop-1-en-1-yl)thio)propanoate (2)**

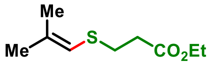


**2**

Following general procedure **I**, 1-bromo-2-methylprop-1-ene (24 µL, 0.24 mmol, 1.2 equiv), ethyl 3-mercaptopropanoate (26 µL, 0.20 mmol, 1.0 equiv), and 4CzIPN (4.0 mg, 2.5 mol%), were stirred for 6 h. After the workup, the organic phases were concentrated under vacuum. The crude product was purified by flash chromatography using silica, EtOAc:Hexane to afford the product **2** as colourless oil (64 mg, 85%).

- **$^{1}$H NMR (400 MHz, $CDCl_3$):** δ 5.58 (p, *J* = 1.3 Hz, 1H), 4.12 (q, *J* = 7.2 Hz, 2H), 2.83 (t, *J* = 7.4 Hz, 2H), 2.57 (t, *J* = 7.4 Hz, 2H), 1.73 (dd, *J* = 14.7, 1.3 Hz, 6H), 1.24 (t, *J* = 7.2 Hz, 3H).
- **$^{13}$C NMR (101 MHz, $CDCl_3$):** δ 171.9, 136.5, 116.9, 60.6, 35.4, 29.0, 25.2, 19.6, 14.2.
- **HRMS (ESI):** calcd. for $C_9H_{16}O_2S^+$ [$M^+$] 188.0871, found = 188.0869.

**Ethyl 3-((3-methylbut-2-en-2-yl)thio)propanoate (3)**

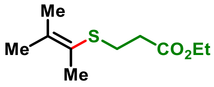


**3**

Following general procedure **I**, 2-bromo-3-methylbut-2-ene (24 µL, 0.24 mmol, 1.2 equiv), ethyl 3-mercaptopropanoate (26 µL, 0.20 mmol, 1.0 equiv), and 4CzIPN (4.0 mg, 2.5 mol%), were stirred for 6 h. After the workup, the organic phases were concentrated under vacuum. The crude product was purified by flash chromatography using silica, EtOAc:Hexane to afford the product **3** as yellow oil (44 mg, 58%).

- **$^{1}$H NMR (400 MHz, $CD_3CN$)** δ 4.08 (q, *J* = 7.1 Hz, 2H), 2.84 (t, *J* = 7.0 Hz, 2H), 2.46 (t, *J* = 7.0 Hz, 2H), 1.95 (p, *J* = 1.2 Hz, 3H), 1.88 (t, *J* = 1.5 Hz, 3H), 1.76 (d, *J* = 1.1 Hz, 3H), 1.20 (t, *J* = 7.1 Hz, 3H).
- **$^{13}$C NMR (101 MHz, $CD_3CN$)** δ 172.72, 137.04, 121.37, 61.17, 35.64, 27.51, 23.08, 21.33, 19.77, 14.53.
- **HRMS (ESI):** calcd. for $C_{10}H_{18}O_2S^+$ [$M^+$] 202.1028; found = 202.1026.

**Ethyl 3-((1*H*-inden-2-yl)thio)propanoate (4)**

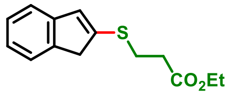


**4**

Following general procedure **I**, 2-bromo-1*H*-indene (47 mg, 0.24 mmol, 1.2 equiv), ethyl 3-mercaptopropanoate (26 µL, 0.20 mmol, 1.0 equiv), and 4CzIPN (4.0 mg, 2.5 mol%), were stirred for 6 h. After the workup, the organic phases were concentrated under vacuum. The crude product was purified by flash chromatography using silica, EtOAc:Hexane to afford the product **4** as yellow oil (56 mg, 56%).

- **$^1$H NMR (400 MHz, $CD_3CN$):** δ 7.36 (dq, *J* = 7.4, 0.9 Hz, 1H), 7.27 – 7.16 (m, 2H), 7.08 (ddd, *J* = 7.3, 6.9, 1.8 Hz, 1H), 6.61 (q, *J* = 1.2 Hz, 1H), 4.11 (q, *J* = 7.1 Hz, 2H), 3.53 – 3.47 (m, 2H), 3.18 (t, *J* = 7.1 Hz, 2H), 2.71 (t, *J* = 7.1 Hz, 2H), 1.22 (t, *J* = 7.1 Hz, 3H).
- **$^{13}$C NMR (101 MHz, $CD_3CN$)** δ 172.5, 145.9, 143.9, 143.5, 127.5, 126.1, 124.6, 124.2, 120.2, 61.4, 42.7, 34.7, 28.1, 14.5.
- **HRMS (ESI):** calcd. for $C_{14}H_{16}O_2S^+$ [$M^+$] 248.0871; found = 248.0859.

**Ethyl 3-(styrylthio)propanoate (5)**

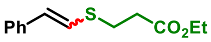


**5**

Following general procedure **I**, (2-bromovinyl)benzene (26 µL, 0.24 mmol, 1.2 equiv), ethyl 3-mercaptopropanoate (26 µL, 0.20 mmol, 1.0 equiv), and 4CzIPN (4.0 mg, 2.5 mol%), were stirred for 6 h. After the workup, the organic phases were concentrated under vacuum. The crude product was purified by flash chromatography using silica, EtOAc:Hexane to afford the product **5** as yellow oil (68 mg, 72%, 60:40 *dr*).

- **$^1$H NMR (400 MHz, $CD_3CN$)** δ 7.47 – 7.43 (m, 1H), 7.39 – 7.28 (m, 3H), 7.26 – 7.17 (m, 1H), 6.86 (d, *J* = 15.7 Hz, 0.5H), 6.57 – 6.46 (m, 1H), 6.36 (d, *J* = 10.9 Hz, 0.5H), 4.11 (qd, *J* = 7.1, 1.7 Hz, 2H), 3.05 (q, *J* = 7.2 Hz, 2H), 2.68 (td, *J* = 7.0, 4.5 Hz, 2H), 1.22 (td, *J* = 7.1, 3.7 Hz, 3H).
- **$^{13}$C NMR (101 MHz, $CD_3CN$):** δ 172.5, 172.4, 138.15, 138.12, 129.7, 129.5, 129.3, 128.3, 128.02, 128.0, 127.8, 126.5, 126.5, 125.9, 61.5, 61.4, 36.0, 35.4, 31.4, 28.3, 14.58, 14.56.
- **HRMS (ESI):** calcd. for $C_{13}H_{16}O_2S^+$ [$M^+$] 238.0871; found = 238.0864.

**Ethyl 3-((4-bromostyryl)thio)propanoate (6)**

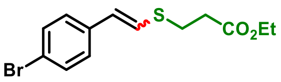


**6**

Following general procedure **I**, 1-bromo-4-(2-bromovinyl)benzene (52 mg, 0.24 mmol, 1.2 equiv), ethyl 3-mercaptopropanoate (26 µL, 0.20 mmol, 1.0 equiv), and 4CzIPN (4.0 mg, 2.5 mol%), were stirred for 6 h. After the workup, the organic phases were concentrated under vacuum. The crude product was purified by flash chromatography using silica, EtOAc:Hexane to afford the product **6** as yellow oil (92 mg, 73%, 60:40 *dr*).

- **$^1$H NMR (400 MHz, $CDCl_3$)** δ 7.45 (dd, *J* = 20.2, 8.6 Hz, 2H), 7.33 (d, *J* = 8.5 Hz, 1.5H), 7.16 (d, *J* = 8.5 Hz, 0.5H), 6.72 (d, *J* = 15.6 Hz, 0.5H), 6.51 – 6.37 (m, 1H), 6.29 (d, *J* = 10.9 Hz, 0.5H), 4.18 (q, *J* = 7.1 Hz, 2H), 3.11 – 3.02 (m, 2H), 2.71 (td, *J* = 7.3, 1.4 Hz, 2H), 1.28 (td, *J* = 7.1, 2.3 Hz, 3H).

- **$^{13}$C NMR (101 MHz, $CDCl_3$):** δ 171.5, 171.3, 135.7, 135.5, 132.3, 131.6, 131.2, 130.1, 127.5, 127.0, 126.5, 125.1, 125.0, 120.6, 120.4, 60.8, 35.3, 34.6, 30.6, 27.5, 14.1.
- **HRMS (ESI):** calcd. for $C_{13}H_{15}BrO_2S^+$ [$M^+$] 313.9976; found = 313.9968.

**4-(2-((2-methylprop-1-en-1-yl)thio)ethyl)pyridine (7)**

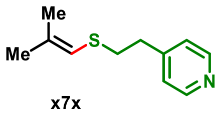


x7x

Following general procedure **I**, 1-bromo-2-methylprop-1-ene (25 µL, 0.24 mmol, 1.2 equiv), 4-(2-mercaptoethyl)pyridin-1-ium chloride (35 mg, 0.20 mmol, 1.0 equiv), and 4CzIPN (4.0 mg, 2.5 mol%), were stirred for 6 h. After the workup, the organic phases were concentrated under vacuum. The crude product was purified by flash chromatography using silica, EtOAc:Hexane to afford the product **7** as yellow oil (44 mg, 57%).

- **$^1$H NMR (400 MHz, $CDCl_3$)** δ 8.51 – 8.50 (m, 2H), 7.13 – 7.12 (m, 2H), 5.59 (t, 1H), 2.91 – 2.83 (m, 4H), 1.76 (dd, *J* = 14.5, 1.2 Hz, 6H).
- **$^{13}$C NMR (101 MHz, $CDCl_3$):** δ 149.8, 149.1, 136.2, 124.0, 117.0, 35.9, 34.2, 25.3, 19.7.
- **HRMS (ESI):** calcd. for $C_{11}H_{15}NS^+$ [$M^+$] 193.0925; found = 193.0925.

**Benzyl(2-methylprop-1-en-1-yl)sulfane (8)**

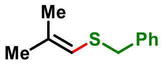


8

Following general procedure **I**, 1-bromo-2-methylprop-1-ene (25 µL, 0.24 mmol, 1.2 equiv), phenylmethanethiol (24 µL, 0.20 mmol, 1.0 equiv), and 4CzIPN (4.0 mg, 2.5 mol%), were stirred for 6 h. After the workup, the organic phases were concentrated under vacuum. The crude product was purified by flash chromatography using silica, EtOAc:Hexane to afford the product **8** as colourless oil (64 mg, 84%).

- **$^1$H NMR (400 MHz, $CDCl_3$):** δ 7.24 – 7.14 (m, 5H), 5.54 – 5.53 (m, 1H), 3.72 (s, 2H), 1.64 (dd, *J* = 12.8, 1.3 Hz, 6H).
- **$^{13}$C NMR (101 MHz, $CDCl_3$):** δ 138.5, 135.5, 128.8, , 128.49, 127.0, 116.9, 38.3, 25.3, 19.7.
- **HRMS (ESI):** calcd. for $C_{11}H_{14}S^+$ [$M^+$] 178.0816; found = 178.0810.

**(4-fluorobenzyl)(2-methylprop-1-en-1-yl)sulfane (9)**

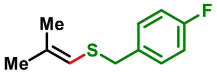


9

Following general procedure **I**, 1-bromo-2-methylprop-1-ene (25 µL, 0.24 mmol, 1.2 equiv), (4-fluorophenyl)methanethiol (21 µL, 0.20 mmol, 1.0 equiv), and 4CzIPN (4.0 mg, 2.5 mol%), were stirred for 6 h. After the workup, the organic phases were concentrated under vacuum. The crude product was purified by flash chromatography using silica, EtOAc:Hexane to afford the product **9** as yellow oil (60 mg, 75%).

- **$^{1}$H NMR (400 MHz, $CDCl_3$):** δ 7.21 – 7.18 (m, 3H), 6.94 – 6.89 (m, 2H), 5.51 (p, *J* = 1.3 Hz, 1H), 3.70 (s, 2H), 1.64 (dd, *J* = 17.0, 1.2 Hz, 6H).
- $^{13}$C NMR (101 MHz, $CDCl_3$) δ 162.0 (d, *J* = 245.3 Hz), 136.3, 134.4 (d, *J* = 3.3 Hz), 130.4 (d, *J* = 8.2 Hz), 116.7, 115.4 (d, *J* = 21.3 Hz), 37.71, 25.4, 19.8.
- **$^{19}$F NMR (376 MHz, $CDCl_3$)** δ -116.3.
- **HRMS (ESI):** calcd. for $C_{11}H_{13}FS^+$ [$M^+$] 196.0722, found = 196.0712.

**Ethyl 2-((2-methylprop-1-en-1-yl)thio)propanoate (10)**

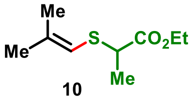


10

Following general procedure **I**, 1-bromo-2-methylprop-1-ene (25 µL, 0.24 mmol, 1.2 equiv), ethyl 2-mercaptopropanoate (25 µL, 0.20 mmol, 1.0 equiv), and 4CzIPN (4.0 mg, 2.5 mol%), were stirred for 6 h. After the workup, the organic phases were concentrated under vacuum. The crude product was purified by flash chromatography using silica, EtOAc:Hexane to afford the product **10** as colourless oil (54 mg, 71%).

- **$^{1}$H NMR (400 MHz, $CDCl_3$):** δ 5.75 (hept, *J* = 1.3 Hz, 1H), 4.16 (dd, *J* = 7.1, 5.3 Hz, 2H), 3.43 (q, *J* = 7.1 Hz, 1H), 1.76 (dd, *J* = 15.1, 1.3 Hz, 6H), 1.44 (d, *J* = 7.1 Hz, 3H), 1.25 (t, *J* = 7.1 Hz, 3H).
- **$^{13}$C NMR (101 MHz, $CDCl_3$):** δ 172.7, 138.4, 114.1, 61.1, 42.9, 25.4, 19.6, 17.1, 14.1.
- **HRMS (ESI):** calcd. for $C_9H_{16}O_2S^+$ [$M^+$] 188.0871; found = 188.0871.

**Cyclohexyl(2-methylprop-1-en-1-yl)sulfane (11)**

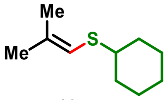


11

Following general procedure **I**, 1-bromo-2-methylprop-1-ene (25 µL, 0.24 mmol, 1.2 equiv), cyclohexanethiol (25 µL, 0.20 mmol, 1.0 equiv), and 4CzIPN (4.0 mg, 2.5 mol%), were stirred for 6 h. After the workup, the organic phases were concentrated under vacuum. The crude product was purified by flash chromatography using silica, EtOAc:Hexane to afford the product **11** as colourless oil (48 mg, 70%).

- **$^{1}$H NMR (400 MHz, $CDCl_3$):** δ 5.69 – 5.68 (m, 1H), 2.73 – 2.70 (m, 1H), 2.03 – 1.96 (m, 2H), 1.76 (dd, *J* = 15.1, 1.2 Hz, 6H), 1.63 – 1.56 (m, 2H), 1.35 – 1.25 (m, 6H).
- **$^{13}$C NMR (101 MHz, $CDCl_3$):** δ 134.7, 116.3, 50.0, 45.7, 33.8, 32.9, 26.1, 25.8, 25.3, 19.6.
- **HRMS (ESI):** calcd. for $C_{10}H_{18}S^+$ [$M^+$] 170.1129; found = 170.1119

**Cyclohexyl(styryl)sulfane (12)**

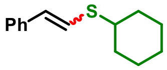


12

Following general procedure **I**, (2-bromovinyl)benzene (26 µL, 0.24 mmol, 1.2 equiv), cyclohexanethiol (25 µL, 0.20 mmol, 1.0 equiv), and 4CzIPN (4.0 mg, 2.5 mol%), were stirred for 6 h. After the workup, the

organic phases were concentrated under vacuum. The crude product was purified by flash chromatography using silica, EtOAc:Hexane to afford the product **12** as colourless oil (72 mg, 82%, 55:45 *dr*).

- **$^{1}$H NMR (400 MHz, CDCl$_3$):** δ 7.53 – 7.55 (m, 1H), 7.41 (t, *J* = 7.8 Hz, 1.5H), 7.36 – 7.35 (m, 1.5H), 7.29 – 7.24(m, 1H), 6.83 (d, *J* = 15.6 Hz, 0.5H), 6.64 (d, *J* = 15.6 Hz, 0.5H), 6.49 (d, *J* = 11.0 Hz, 0.5H), 6.39 (d, *J* = 11.0 Hz, 0.5H), 3.08 – 3.01 (m, 0.47H), 2.94 (ddt, *J* = 10.7, 7.6, 3.8 Hz, 0.76H), 2.15 – 2.10 (m, 2H), 1.89 – 1.84 (m, 2H), 1.72 – 1.67 (m, 1H), 1.57 – 1.32 (m, 7H).
- **$^{13}$C NMR (101 MHz, CDCl$_3$):** δ 137.1, 128.7, 128.6, 128.1, 126.8, 126.4, 126.0, 125.5, 125.0, 124.0, 47.7, 45.3, 33.6, 33.5, 26.1, 26.0, 25.6, 25.5.
- **HRMS (ESI):** calcd. for $C_{14}H_{18}S^+$ [M$^+$] 218.1129; found = 218.1129.

**((3*s*,5*s*,7*s*)-adamantan-1-yl)(2-methylprop-1-en-1-yl)sulfane (13)**

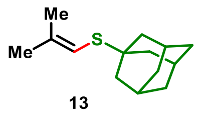


**13**

Following general procedure **I**, 1-bromo-2-methylprop-1-ene (25 µL, 0.24 mmol, 1.2 equiv), (3*s*,5*s*,7*s*)-adamantane-1-thiol (34 mg, 0.20 mmol, 1.0 equiv), and 4CzIPN (4.0 mg, 2.5 mol%), were stirred for 6 h. After the workup, the organic phases were concentrated under vacuum. The crude product was purified by flash chromatography using silica, EtOAc:Hexane to afford the product **13** as colourless oil (55 mg, 62%).

- **$^{1}$H NMR (400 MHz, CDCl$_3$):** δ 5.83 – 5.82 (m, 1H), 2.05 – 2.54 (m, 4H), 1.87 (d, *J* = 2.9 Hz, 6H), 1.81 (t, *J* = 2.2 Hz, 5H), 1.76 (d, *J* = 1.1 Hz, 2H), 1.69-1.66(m, 4H)
- **$^{13}$C NMR (101 MHz, CDCl$_3$):** δ 137.4, 112.1, 47.6, 47.4, 45.9, 43.5, 43.1, 36.3, 36.2, 30.14, 30.07, 29.8, 25.7, 19.7.
- **HRMS (ESI):** calcd. for $C_{14}H_{22}S^+$ [M$^+$] 222.1442; found = 222.1437.

**((3*s*,5*s*,7*s*)-adamantan-1-yl)(styryl)sulfane (14)**

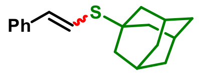


**14**

Following general procedure **I**, (2-bromovinyl)benzene (26 µL, 0.24 mmol, 1.2 equiv), (3*s*,5*s*,7*s*)-adamantane-1-thiol (34 mg, 0.20 mmol, 1.0 equiv), and 4CzIPN (4.0 mg, 2.5 mol%), were stirred for 6 h. After the workup, the organic phases were concentrated under vacuum. The crude product was purified by flash chromatography using silica, EtOAc:Hexane to afford the product **14** as colourless oil (86 mg, 79%, 55:45 *dr*).

- **$^{1}$H NMR (400 MHz, CDCl$_3$):** δ 7.40 (dd, *J* = 8.1, 1.4 Hz, 1H), 7.26 – 7.18 (m, 3H), 7.12 – 7.07 (m, 1H), 6.83 (d, *J* = 15.5 Hz, 0.5H), 6.61 (d, *J* = 15.5 Hz, 0.5H), 6.42 – 6.35 (m, 1H), 2.04 – 1.97 (m, 3.5H), 1.88 (dd, *J* = 7.8, 2.9 Hz, 6H), 1.74 (d, *J* = 3.0 Hz, 0.5H), 1.63 (t, *J* = 3.4 Hz, 5H).
- **$^{13}$C NMR (101 MHz, CDCl$_3$):** δ 137.2, 137.0, 131.7, 128.6, 128.5, 128.0, 127.0, 126.3, 125.7, 125.1, 121.2, 120.1, 46.5, 46.4, 43.6, 43.2, 43.0, 41.7, 36.2, 36.1, 36.1, 36.1, 30.0, 29.7, 29.7.
- **HRMS (ESI):** calcd. for $C_{18}H_{22}S^+$ [M$^+$]  270.1442; found = 270.1437.

**(2-methylprop-1-en-1-yl)(phenyl)sulfane (15)**

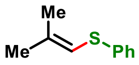


**15**

Following general procedure **I**, 1-bromo-2-methylprop-1-ene (25 µL, 0.24 mmol, 1.2 equiv), benzenethiol (21 µL, 0.20 mmol, 1.0 equiv), and 4CzIPN (4.0 mg, 2.5 mol%), were stirred for 6 h. After the workup, the organic phases were concentrated under vacuum. The crude product was purified by flash chromatography using silica, EtOAc:Hexane to afford the product **15** as colourless oil (58 mg, 89%).

- **$^1$H NMR (400 MHz, $CDCl_3$):** δ 7.22 – 7.15 (m, 4H), 7.08 – 7.04 (m, 1H), 5.84 – 5.83 (m, 1H), 1.80 (dd, *J* = 8.5, 1.3 Hz, 6H).
- **$^{13}$C NMR (101 MHz, $CDCl_3$):** δ 140.1, 137.4, 128.8, 127.7, , 125.5, 115.2, 25.4, 19.7.
- **HRMS (ESI):** calcd. for $C_{10}H_{12}S^+$ [$M^+$] 164.0660; found = 164.0650.

**(1*H*-inden-2-yl)(phenyl)sulfane (16)**

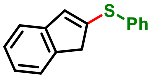


**16**

Following general procedure **I**, 2-bromo-1H-indene (47 mg, 0.24 mmol, 1.2 equiv), benzenethiol (21 µL, 0.20 mmol, 1.0 equiv), and 4CzIPN (4.0 mg, 2.5 mol%), were stirred for 6 h. After the workup, the organic phases were concentrated under vacuum. The crude product was purified by flash chromatography using silica, EtOAc:Hexane to afford the product **16** as colourless oil (65 mg, 73%).

- **$^1$H NMR (400 MHz, $CDCl_3$):** δ 7.40 – 7.37 (m, 2H), 7.26 – 7.18 (m, 4H), 7.12 – 7.09 (m, 2H), 7.00 (ddd, *J* = 7.4, 5.4, 3.3 Hz, 1H), 6.57 (d, *J* = 1.8 Hz, 1H), 3.35 (t, *J* = 1.2 Hz, 2H).
- **$^{13}$C NMR (101 MHz, $CDCl_3$):** δ 144.5, 143.2, 142.0, 133.9, 132.2, 130.7, 129.2, 127.7, 127.4, 124.3, 123.3, 119.9, 42.0.
- **HRMS (ESI):** calcd. for $C_{15}H_{12}S^+$ [$M^+$] 224.0660, found = 224.0653.

**(2,4-dimethylphenyl)(2-methylprop-1-en-1-yl)sulfane (17)**

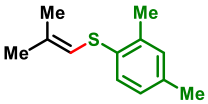


**17**

Following general procedure **I**, 1-bromo-2-methylprop-1-ene (25 µL, 0.24 mmol, 1.2 equiv), 2,4-dimethylbenzenethiol (27 µL, 0.20 mmol, 1.0 equiv), and 4CzIPN (4.0 mg, 2.5 mol%), were stirred for 6 h. After the workup, the organic phases were concentrated under vacuum. The crude product was purified by flash chromatography using silica, EtOAc:Hexane to afford the product **17** as colourless oil (71 mg, 92%).

- **$^1$H NMR (400 MHz, $CDCl_3$):** δ 7.18 (d, *J* = 7.9 Hz, 1H), 7.01 – 6.96 (m, 2H), 5.82-5.81 (m, 1H), 2.36 (s, 3H), 2.31 (s, 3H), 1.90 (d, *J* = 1.3 Hz, 6H).
- **$^{13}$C NMR (101 MHz, $CDCl_3$):** δ 139.0, 136.9, 135.65, 132.74, 131.0, 128.5, 127.2, 116.0, 25.4, 20.9, 20.3, 19.7.

- **HRMS (ESI):** calcd. for $C_{12}H_{16}S^+$ $[M]^+$ 192.0973; found: 192.0971

**(2,6-dimethylphenyl)(2-methylprop-1-en-1-yl)sulfane (18)**

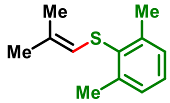


**18**

Following general procedure **I**, 1-bromo-2-methylprop-1-ene (25 µL, 0.24 mmol, 1.2 equiv), 2,6-dimethylbenzenethiol (27 µL, 0.20 mmol, 1.0 equiv), and 4CzIPN (4.0 mg, 2.5 mol%), were stirred for 6 h. After the workup, the organic phases were concentrated under vacuum. The crude product was purified by flash chromatography using silica, EtOAc:Hexane to afford the product **18** as colourless oil (69 mg, 90%).

- **$^1$H NMR (400 MHz, $CD_3CN$)** δ 7.14 – 7.06 (m, 3H), 5.41 (hept, *J* = 1.3 Hz, 1H), 2.43 (s, 6H), 1.83 (d, *J* = 1.2 Hz, 3H), 1.74 (d, *J* = 1.4 Hz, 3H).
- **$^{13}$C NMR (101 MHz, $CD_3CN$)** δ 143.26, 135.22, 134.16, 129.31, 129.13, 118.98, 24.95, 22.15, 19.40.
- **HRMS (ESI):** calcd. for $C_{12}H_{16}S^+$ $[M]^+$ 192.0967; found: 192.0969.

**(3,4-difluorophenyl)(2-methylprop-1-en-1-yl)sulfane (19)**

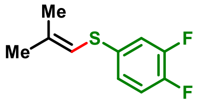


**19**

Following general procedure **I**, 1-bromo-2-methylprop-1-ene (25 µL, 0.24 mmol, 1.2 equiv), 3,4-difluorobenzenethiol (22 µL, 0.20 mmol, 1.0 equiv), and 4CzIPN (4.0 mg, 2.5 mol%), were stirred for 6 h. After the workup, the organic phases were concentrated under vacuum. The crude product was purified by flash chromatography using silica, EtOAc:Hexane to afford the product **19** as yellow oil (60 mg, 75%).

- **$^1$H NMR (400 MHz, $CDCl_3$):** δ 7.10 – 6.96 (m, 3H), 5.85 – 5.83 (m, 1H), 1.89 (dd, *J* = 15.7, 1.3 Hz, 6H).
- **$^{13}$C NMR (101 MHz, $CDCl_3$)** δ 150.8 (dd, *J* = 179.1, 13.0 Hz), 148.3 (dd, *J* = 175.9, 12.9 Hz), 142.3, 133.9 (dd, *J* = 5.8, 3.8 Hz), 123.6 (dd, *J* = 6.0, 3.8 Hz), 117.5 (d, *J* = 17.8 Hz), 116.6 (d, *J* = 19.0 Hz), 114.3, 25.4, 19.7.
- **$^{19}$F NMR (376 MHz, $CDCl_3$)** δ -137.33 (d, *J* = 21.0 Hz), -142.22 (d, *J* = 21.1 Hz).
- **HRMS (ESI):** calcd. for $C_{10}H_{10}F_2S^+$ $[M^+]$ 200.0471; found = 200.0468.

**2-((2-methylprop-1-en-1-yl)thio)benzo[*d*]thiazole (20)**

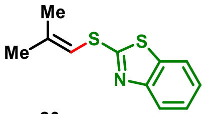


**20**

Following general procedure **I**, 1-bromo-2-methylprop-1-ene (25 µL, 0.24 mmol, 1.2 equiv), benzo[*d*]thiazole-2-thiol (34 mg, 0.20 mmol, 1.0 equiv), and 4CzIPN (4.0 mg, 2.5 mol%), were stirred for

6 h. After the workup, the organic phases were concentrated under vacuum. The crude product was purified by flash chromatography using silica, EtOAc:Hexane to afford the product **20** as colourless oil (117 mg, 76%).

- **$^1$H NMR (400 MHz, $CDCl_3$):** δ 7.81 – 7.78 (m, 1H), 7.67 (dd, *J* = 8.0, 1.2 Hz, 1H), 7.33 (ddd, *J* = 8.3, 7.3, 1.3 Hz, 1H), 7.22 – 7.18 (m, 1H), 6.21 – 6.20 (m, 1H), 1.89 (dd, *J* = 18.9, 1.3 Hz, 6H).
- **$^{13}$C NMR (101 MHz, $CDCl_3$):** δ 168.4, 154.0, 146.4, 135.2, 126.1, 124.1, 121.7, 120.9, 111.1, 25.6, 20.2.
- **HRMS (ESI):** calcd. for $C_{11}H_{11}NS_2^+$ [$M^+$] 221.0333, found = 221.0331.

**(2-(phenylsulfonyl)vinyl)benzene (21)**

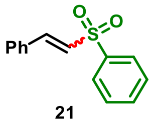


**21**

Following general procedure **I**, (2-bromovinyl)benzene (26 µL, 0.24 mmol, 1.2 equiv), sodium benzenesulfinate (33 mg, 0.20 mmol, 1.0 equiv), and 4CzIPN (4.0 mg, 2.5 mol%), were stirred for 24 h. After the workup, the organic phases were concentrated under vacuum. The crude product was purified by flash chromatography using silica, EtOAc:Hexane to afford the product **21** as light yellow oil (79 mg, 81%, 70:30 *dr*).

- **$^1$H NMR (400 MHz, $CD_3CN$)** δ 7.97 – 7.90 (m, 1H), 7.85 – 7.77 (m, 1H), 7.72 – 7.47 (m, 5.5H), 7.43 – 7.31 (m, 3H), 7.21 – 7.08 (m, 1H), 6.60 (d, *J* = 12.1 Hz, 0.5H).
- **$^{13}$C NMR (101 MHz, $CD_3CN$)** δ 143.3, 142.4, 142.1, 141.9, 134.5, 134.5, 133.7, 133.5, 132.1, 131.7, 130.9, 130.6, 130.5, 130.2, 129.9, 129.7, 128.9, 128.6, 128.3, 128.2.

The spectra data are in accordance with the literature report[10].

**(2-methylprop-1-en-1-yl)(phenyl)selane (22)**

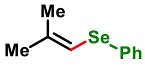


**22**

Following general procedure **I**, 1-bromo-2-methylprop-1-ene (25 µL, 0.24 mmol, 1.2 equiv), benzeneselenol (22 µL, 0.20 mmol, 1.0 equiv), and 4CzIPN (4.0 mg, 2.5 mol%), were stirred for 6 h. After the workup, the organic phases were concentrated under vacuum. The crude product was purified by flash chromatography using silica, EtOAc:Hexane to afford the product **22** as colourless oil (71 mg, 92%).

- **$^1$H NMR (400 MHz, $CDCl_3$):** δ 7.37 – 7.34 (m, 2H), 7.20 – 7.11 (m, 3H), 6.07 – 6.06 (m, 1H), 1.89 (dd, *J* = 21.7, 1.2 Hz, 6H).
- **$^{13}$C NMR (101 MHz, $CDCl_3$):** δ 140.7, 132.0, 130.8, 129.0, 126.3, 112.2, 25.8, 21.5.
- **HRMS (ESI):** calcd. for $C_{10}H_{12}Se^+$ [$M^+$] 212.0104, found = 212.0099.

**But-3-ene-1,1,4-triyltribenzene (23)**

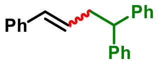


**23**

Following general procedure **II**, (2-bromovinyl)benzene (22 µL, 0.20 mmol, 1.0 equiv), ethene-1,1-diyldibenzene (54 mg, 0.30 mmol, 1.5 equiv), DIPEA (39 mg, 0.30 mmol, 1.5 equiv)and 4CzIPN (4.0 mg, 2.5 mol%), were stirred for 24 h. After the workup, the organic phases were concentrated under vacuum. The crude product was purified by flash chromatography using silica, EtOAc:Hexane to afford the product **23** as yellow oil (38 mg, 67%, 80:20 *dr*).

- **$^{1}$H NMR (400 MHz, $CD_3CN$)** δ 7.36 – 7.21 (m, 12H), 7.17 (ddt, *J* = 7.1, 5.2, 1.6 Hz, 3H), 6.41 (dt, *J* = 15.9, 1.5 Hz, 1H), 6.16 (dt, *J* = 15.9, 7.0 Hz, 1H), 4.13 (dt, *J* = 11.8, 8.0 Hz, 1H), 2.97 (ddd, *J* = 8.2, 7.0, 1.4 Hz, 2H).
- **$^{13}$C NMR (101 MHz, $CD_3CN$)** δ 145.88, 145.74, 138.53, 138.44, 132.26, 131.80, 130.69, 129.88, 129.66, 129.51, 129.44, 129.24, 128.79, 128.01, 127.75, 127.24, 127.18, 126.73, 52.34, 52.23, 39.42, 35.16.
- **HRMS (ESI):** calcd. for $C_{22}H_{20}^{+}$ [M]$^{+}$ 284.1559; found: 284.1563

**4,4,5,5-Tetramethyl-2-styryl-1,3,2-dioxaborolane (24)**

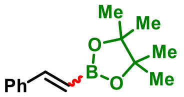


**24**

Following general procedure **III**, (2-bromovinyl)benzene (22 µL, 0.20 mmol, 1.0 equiv), $B_2Pin_2$ (76 mg, 0.30 mmol, 1.5 equiv), TMG (36 mg, 0.30 mmol, 1.5 equiv)and 4CzIPN (4.0 mg, 2.5 mol%), were stirred for 48 h. After the workup, the organic phases were concentrated under vacuum. The crude product was purified by flash chromatography using silica, EtOAc:Hexane to afford the product **24** as yellow oil (7 mg, 16%, 70:30 *dr*).

- **$^{1}$H NMR (400 MHz, $CD_3CN$)** δ 7.60 – 7.48 (m, 2H), 7.42 – 7.28 (m, 4H), 6.16 (d, *J* = 18.4 Hz, 1H), 1.26 (s, 12H).
- **$^{13}$C NMR (101 MHz, $CD_3CN$)** δ 150.1, 138.4, 130.1, 129.7, 128.0, 84.3, 25.1.

The spectra data are in accordance with the literature report[11].

**Diethyl styrylphosphonate (25)**

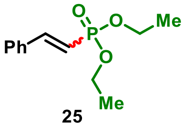


**25**

Following general procedure **I**, (2-bromovinyl)benzene (26 µL, 0.24 mmol, 1.0 equiv), triethyl phosphite (52 µL, 0.30 mmol, 1.5 equiv), and 4CzIPN (4.0 mg, 2.5 mol%), were stirred for 48 h. After the workup, the organic phases were concentrated under vacuum. The crude product was purified by flash chromatography using silica, EtOAc:Hexane to afford the product **25** as yellow oil (50 mg, 52%, 60:40 *dr*).

- **$^{1}$H NMR (400 MHz, $CDCl_3$) δ** 7.67 (dd, *J* = 7.8, 1.9 Hz, 1H), 7.57 – 7.43 (m, 1H), 7.39 – 7.14 (m, 4H), 6.25 (t, *J* = 17.6 Hz, 0.5H), 5.80 (dd, *J* = 15.6, 14.2 Hz, 0.5H), 4.18 – 4.06 (m, 3H), 4.04 – 3.93 (m, 1H), 1.37 – 1.30 (m, 4H), 1.18 (t, *J* = 7.1 Hz, 2H).
- **$^{13}$C NMR (101 MHz, $CDCl_3$) δ** 148.8 (d, *J* = 6.8 Hz), 148.4, 129.8 (d, *J* = 92.0 Hz), 129.6 (d, *J* = 1.7 Hz), 128.5 (d, *J* = 72.4 Hz), 127.7, 116.6 (d, *J* = 185.4 Hz), 113.9 (d, *J* = 191.4 Hz), 63.6 (d, *J* = 5.8 Hz), 61.86 (d, *J* = 5.6 Hz), 61.78 (d, *J* = 5.9 Hz), 16.4 (d, *J* = 6.4 Hz), 16.1 (d, *J* = 6.8 Hz).

- **$^{31}$P NMR (162 MHz, CDCl$_3$)** δ 20.2, 16.7

The spectra data are in accordance with the literature report[12].

**Diphenyl(prop-1-en-2-yl)phosphine oxide (26)**

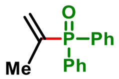

26

Following general procedure **IV**, 2-bromoprop-1-ene (28 µL, 0.30 mmol, 1.5 equiv), diphenylphosphine oxide (41 mg, 0.20 mmol, 1.0 equiv), NiBr$_2$.glyme (3.1 mg, 5 mol%), 4,4'-Dimethoxy-2,2'-bipyridine (4.33 mg, 10 mol%), and 4CzIPN (0.8 mg, 0.5 mol%), were stirred for 24 h. After the workup, the organic phases were concentrated under vacuum. The crude product was purified by flash chromatography using silica, EtOAc:Hexane to afford the product **26** as pale yellow solid (57 mg, 59%).

- **$^1$H NMR (400 MHz, CDCl$_3$)** δ 7.72 – 7.62 (m, 4H), 7.58 – 7.40 (m, 6H), 5.93 (dt, $J$ = 41.1, 1.6 Hz, 1H), 5.61 (dt, $J$ = 19.7, 1.2 Hz, 1H), 1.98 (d, $J$ = 12.2 Hz, 3H).
- **$^{13}$C NMR (101 MHz, CDCl$_3$)** δ 139.3 (d, $J$ = 93.1 Hz), 131.9 (d, $J$ = 2.9 Hz), 131.8 (d, $J$ = 9.8 Hz), 130.9 (d, $J$ = 8.6 Hz), 130.4 (d, $J$ = 9.6 Hz), 128.5 (d, $J$ = 11.8 Hz), 19.1 (d, $J$ = 11.7 Hz).
- **$^{31}$P NMR (162 MHz, CDCl$_3$)** δ 31.9.
- **HRMS (ESI):** calcd. for $C_{15}H_{15}OP^+$ $[M]^+$ 242.0861, found = 242.0854.

The spectra data are in accordance with the literature report[13].

## (2-methylprop-1-en-1-yl)diphenylphosphine oxide (27)

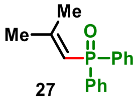

27

Following general procedure **IV**, 1-bromo-2-methylprop-1-ene (31 µL, 0.30 mmol, 1.5 equiv), diphenylphosphine oxide (41 mg, 0.20 mmol, 1.0 equiv), NiBr$_2$.glyme (3.1 mg, 5 mol%), 4,4'-Dimethoxy-2,2'-bipyridine (4.33 mg, 10 mol%), and 4CzIPN (0.8 mg, 0.5 mol%), were stirred for 24 h. After the workup, the organic phases were concentrated under vacuum. The crude product was purified by flash chromatography using silica, EtOAc:Hexane to afford the product **27** as white solid (64 mg, 62%).

- **$^1$H NMR (400 MHz, CDCl$_3$)** δ 7.71 (ddt, $J$ = 11.9, 6.6, 1.6 Hz, 4H), 7.49 – 7.38 (m, 6H), 5.87 (dt, $J$ = 25.5, 1.3 Hz, 1H), 2.05 (dd, $J$ = 2.6, 1.0 Hz, 3H), 1.98 (d, $J$ = 1.2 Hz, 3H).
- **$^{13}$C NMR (101 MHz, CDCl$_3$)** δ 160.43 (d, $J$ = 1.6 Hz), 134.88 (d, $J$ = 104.0 Hz), 131.25 (d, $J$ = 2.7 Hz), 130.82 (d, $J$ = 9.6 Hz), 128.42 (d, $J$ = 11.8 Hz), 116.94 (d, $J$ = 105.4 Hz), 28.69 (d, $J$ = 17.8 Hz), 21.87 (d, $J$ = 7.4 Hz).
- **$^{31}$P NMR (162 MHz, CDCl$_3$)** δ 21.4.
- **HRMS (ESI):** calcd. for $C_{16}H_{17}OP^+$ $[M]^+$ 256.1017; found = 256.1005.

**(*E*)-diphenyl(2-(trimethylsilyl)vinyl)phosphine oxide (28)**

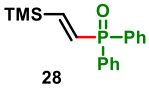

28

Following general procedure **IV**, (*E*)-(2-bromovinyl)trimethylsilane (36 mg, 0.30 mmol, 1.5 equiv), diphenylphosphine oxide (41 mg, 0.20 mmol, 1.0 equiv), NiBr$_2$.glyme (3.1 mg, 5 mol%), 4,4'-Dimethoxy-2,2'-bipyridine (4.33 mg, 10 mol%), and 4CzIPN (0.8 mg, 0.5 mol%), were stirred for 24 h. After the

workup, the organic phases were concentrated under vacuum. The crude product was purified by flash chromatography using silica, EtOAc:Hexane to afford the product **28** as pale yellow oil (54 mg, 91%).

- **$^1$H NMR (400 MHz, $CD_3CN$)** δ 7.73 – 7.65 (m, 4H), 7.57 – 7.45 (m, 6H), 7.25 (dd, *J* = 29.5, 20.4 Hz, 1H), 7.09 (dd, *J* = 32.2, 20.4 Hz, 1H), 0.14 (s, 9H).
- **$^{13}$C NMR (101 MHz, $CD_3CN$)** δ 155.11 (d, *J* = 5.1 Hz), 139.03 (d, *J* = 89.1 Hz), 134.63 (d, *J* = 101.4 Hz), 132.66 (d, *J* = 2.9 Hz), 131.89 (d, *J* = 9.6 Hz), 129.63 (d, *J* = 11.7 Hz), -1.85.
- **$^{31}$P NMR (162 MHz, $CD_3CN$)** δ 20.85.
- **HRMS (ESI):** calcd. for $C_{17}H_{22}OPSi^+$ $[M+H]^+$ 301.1178; found: 301.1180.

**Cyclohex-1-en-1-yldiphenylphosphine oxide (29)**

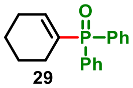


29

Following general procedure **IV**, 1-bromocyclohex-1-ene (35 µL, 0.30 mmol, 1.5 equiv), diphenylphosphine oxide (41 mg, 0.20 mmol, 1.0 equiv), $NiBr_2$.glyme (3.1 mg, 5 mol%), 4,4'-Dimethoxy-2,2'-bipyridine (4.33 mg, 10 mol%), and 4CzIPN (0.8 mg, 0.5 mol%), were stirred for 24 h. After the workup, the organic phases were concentrated under vacuum. The crude product was purified by flash chromatography using silica, EtOAc:Hexane to afford the product **29** as pale yellow oil (57 mg, 59%).

- **$^1$H NMR (400 MHz, $CDCl_3$)** δ 7.65 (ddd, *J* = 11.7, 8.2, 1.5 Hz, 4H), 7.55 – 7.35 (m, 6H), 6.43 – 6.29 (m, 1H), 2.24 – 2.07 (m, 4H), 1.68 – 1.55 (m, 4H).
- **$^{13}$C NMR (101 MHz, $CDCl_3$)** δ 143.24 (d, *J* = 8.5 Hz), 132.04 (d, *J* = 16.1 Hz), 131.77 (d, *J* = 9.7 Hz), 131.56 (d, *J* = 2.9 Hz), 131.04 (d, *J* = 18.5 Hz), 128.32 (d, *J* = 11.8 Hz), 26.28 (d, *J* = 14.4 Hz), 24.42 (d, *J* = 9.4 Hz), 22.01 (d, *J* = 8.3 Hz), 21.37 (d, *J* = 1.6 Hz).
- **$^{31}$P NMR (162 MHz, $CDCl_3$)** δ 31.1.
- **HRMS (ESI):** calcd. for $C_{18}H_{19}OP^+$ $[M]^+$ 282.1174, found : 282.1168.

The spectra data are in accordance with the literature report[13].

**(1*H*-inden-3-yl)diphenylphosphine oxide (30)**

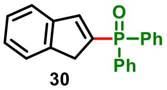


30

Following general procedure **IV**, 2-bromo-1*H*-indene (59 mg, 0.30 mmol, 1.5 equiv), diphenylphosphine oxide (41 mg, 0.20 mmol, 1.0 equiv), $NiBr_2$.glyme (3.1 mg, 5 mol%), 4,4'-Dimethoxy-2,2'-bipyridine (4.33 mg, 10 mol%), and 4CzIPN (0.8 mg, 0.5 mol%), were stirred for 24 h. After the workup, the organic phases were concentrated under vacuum. The crude product was purified by flash chromatography using silica, EtOAc:Hexane to afford the product **30** as pale yellow solid (94 mg, 74%).

- **$^1$H NMR (400 MHz, $CDCl_3$)** δ 7.81 – 7.72 (m, 4H), 7.60 – 7.42 (m, 8H), 7.35 – 7.25 (m, 3H), 3.71 (d, *J* = 2.0 Hz, 2H).
- **$^{13}$C NMR (101 MHz, $CDCl_3$)** δ 145.89 (d, *J* = 9.2 Hz), 145.62 (d, *J* = 11.5 Hz), 142.60 (d, *J* = 16.2 Hz), 139.02 (d, *J* = 109.7 Hz), 132.18 (d, *J* = 106.2 Hz), 131.85 (d, *J* = 2.8 Hz), 131.51 (d, *J* = 10.2 Hz), 128.46 (d, *J* = 12.2 Hz), 126.81 (d, *J* = 1.3 Hz), 124.89 (d, *J* = 435.7 Hz), 124.05 (d, *J* = 1.9 Hz), 40.19 (d, *J* = 11.7 Hz).
- **$^{31}$P NMR (162 MHz, $CDCl_3$) )** δ 24.6.

- **HRMS (ESI):** calcd. for $C_{21}H_{17}OP^+$ [M]$^+$ 316.1017; found :316.1005.

**Diphenyl(styryl)phosphine oxide (31)**

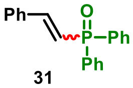


31

Following general procedure **IV**, (2-bromovinyl)benzene (33 µL, 0.30 mmol, 1.5 equiv), diphenylphosphine oxide (41 mg, 0.20 mmol, 1.0 equiv), $NiBr_2$.glyme (3.1 mg, 5 mol%), 4,4'-Dimethoxy-2,2'-bipyridine (4.33 mg, 10 mol%), and 4CzIPN (0.8 mg, 0.5 mol%), were stirred for 24 h. After the workup, the organic phases were concentrated under vacuum. The crude product was purified by flash chromatography using silica, EtOAc:Hexane to afford the product **31** as white solid (89 mg, 73%, 70:30 *dr*).

- **$^1$H NMR (400 MHz, $CDCl_3$)** δ 7.79 – 7.66 (m, 4.5H), 7.58 – 7.42 (m, 7H), 7.40 – 7.30 (m, 3.5H), 7.19 – 7.10 (m, 1H), 6.85 (dd, $J$ = 22.4, 17.4 Hz, 0.77H), 6.30 (dd, $J$ = 19.5, 14.1 Hz, 0.23H).
- **$^{13}$C NMR (101 MHz, $CDCl_3$)** δ 149.79 (d, $J$ = 1.7 Hz), 147.33 (d, $J$ = 3.7 Hz), 134.89 (d, $J$ = 17.8 Hz), 134.62 (d, $J$ = 7.3 Hz), 133.68 (d, $J$ = 105.4 Hz), 132.77 (d, $J$ = 105.8 Hz), 131.68 (d, $J$ = 2.7 Hz), 131.15 (d, $J$ = 10.0 Hz), 130.68 (d, $J$ = 9.8 Hz), 129.98, 129.91, 129.12, 128.43 (d, $J$ = 12.1 Hz), 128.21 (d, $J$ = 12.1 Hz), 128.10 (d, $J$ = 110.4 Hz), 127.74, 121.57 (d, $J$ = 98.2 Hz), 119.05 (d, $J$ = 104.4 Hz).
- **$^{31}$P NMR (162 MHz, $CDCl_3$)** δ 25.1, 20.6.
- **HRMS (ESI):** calcd. for $C_{20}H_{17}OP^+$ [M]$^+$ 304.1017, found :304.1015.

The spectra data are in accordance with the literature report[14].

***N*-(2-methylprop-1-en-1-yl)-1,1-diphenylmethanimine (32)**

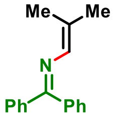


32

Following general procedure **V**, 1-bromo-2-methylprop-1-ene (31 µL, 0.30 mmol, 1.5 equiv), diphenylmethanimine (37 mg, 0.20 mmol, 1.0 equiv), $^t$BuNH$_2$ (44 µL, 0.4 mmol, 2.0 equiv), and 4CzIPN (0.8 mg, 0.5 mol%), $NiBr_2$·glyme (3.2 mg, 5.0 mol%), dOMebpy (4.3 mg, 10 mol%) were stirred for 96 h. After the workup, the organic phases were concentrated under vacuum. The crude product was purified by flash chromatography using silica, EtOAc:Hexane to afford the product **32** (24 mg, 51%)

- **$^1$H NMR (400 MHz, $CD_3CN$)** δ 7.68 – 7.63 (m, 2H), 7.51 – 7.42 (m, 3H), 7.38 – 7.31 (m, 3H), 7.17 – 7.12 (m, 2H), 6.49 (h, $J$ = 1.5 Hz, 1H), 2.10 (d, $J$ = 1.5 Hz, 3H), 1.68 (d, $J$ = 1.4 Hz, 3H).
- **$^{13}$C NMR (101 MHz, $CD_3CN$)** δ 162.4, 140.9, 137.6, 133.3, 130.7, 129.5, 129.38, 129.35, 129.09, 129.07, 23.2, 18.1.
- **HRMS (ESI):** calcd. for $C_{17}H_{17}N^+$ [M]$^+$ 235.1355; found: 235.1351

**(*E*)-1,1-diphenyl-N-(2-(trimethylsilyl)vinyl)methanimine (33)**

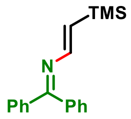

33

Following general procedure **V**, (*E*)-(2-bromovinyl)trimethylsilane (36 mg, 0.30 mmol, 1.5 equiv), diphenylmethanimine (37 mg, 0.20 mmol, 1.0 equiv), $^t$BuNH$_2$ (44 µL, 0.4 mmol, 2.0 equiv), and 4CzIPN (0.8 mg, 0.5 mol%), NiBr$_2$·glyme (3.2 mg, 5.0 mol%), dOMebpy (4.3 mg, 10 mol%) were stirred for 48 h. After the workup, the organic phases were concentrated under vacuum. The crude product was purified by flash chromatography using silica, EtOAc:Hexane to afford the product **33** (26 mg, 47%)

- **$^1$H NMR (400 MHz, CD$_3$CN)** δ 7.73 – 7.57 (m, 2H), 7.54 – 7.33 (m, 6H), 7.24 – 7.14 (m, 2H), 6.95 (d, *J* = 15.9 Hz, 1H), 6.02 (d, *J* = 15.9 Hz, 1H), 0.00 (s, 9H).
- **$^{13}$C NMR (101 MHz, CD$_3$CN)** δ 168.1, 149.7, 140.4, 137.2, 131.6, 129.9, 129.7, 129.6, 129.5, 129.4, 129.2, -1.1.
- **HRMS (ESI):** calcd. for $C_{18}H_{22}NSi^+$ [M+H]$^+$ 280.1522; found: 280.1520

**1,1-Diphenyl-N-styrylmethanimine (34)**

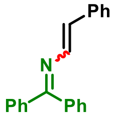

34

Following general procedure **V**, (2-bromovinyl)benzene (55.0 mg, 0.30 mmol, 1.5 equiv), diphenylmethanimine (37 mg, 0.20 mmol, 1.0 equiv), $^t$BuNH$_2$ (44 µL, 0.4 mmol, 2.0 equiv), and 4CzIPN (0.8 mg, 0.5 mol%), NiBr$_2$·glyme (3.2 mg, 5.0 mol%), dOMebpy (4.3 mg, 10 mol%) were stirred for 48 h. After the workup, the organic phases were concentrated under vacuum. The crude product was purified by flash chromatography using silica, EtOAc:Hexane to afford the product **34** (38 mg, 67%, 60:40 *dr*)

- **$^1$H NMR (400 MHz, CD$_3$CN)** δ 7.79 – 7.72 (m, 1.5H), 7.70 – 7.61 (m, 2.5H), 7.55 – 7.35 (m, 7H), 7.33 – 7.16 (m, 5.5H), 6.98 (d, *J* = 13.4 Hz, 0.5H).
- **$^{13}$C NMR (101 MHz, CD$_3$CN)** δ 167.83, 140.42, 138.54, 138.08, 137.54, 137.11, 133.46, 132.06, 131.45, 130.73, 129.97, 129.71, 129.68, 129.61, 129.58, 129.35, 129.24, 128.72, 127.55.
- **HRMS (ESI):** calcd. for $C_{21}H_{17}N^+$ [M]$^+$ 283.1355; found: 283.1345

**Dimethyl(styrylimino)-λ$^6$-sulfanone (35)**

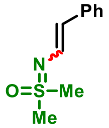

35

Following general procedure **V**, (2-bromovinyl)benzene (55 mg, 0.30 mmol, 1.5 equiv), iminodimethyl- λ$^6$-sulfanone (19 mg, 0.20 mmol, 1.0 equiv), $^t$BuNH$_2$ (44 µL, 0.4 mmol, 2.0 equiv), and 4CzIPN (0.8 mg, 0.5 mol%), NiBr$_2$·glyme (3.2 mg, 5.0 mol%), dOMebpy (4.3 mg, 10 mol%) were stirred for 48 h. After the

workup, the organic phases were concentrated under vacuum. The crude product was purified by flash chromatography using silica, EtOAc:Hexane to afford the product **35** (24 mg, 61%, 90:10 *dr*)

- **$^{1}$H NMR (400 MHz, $CD_3CN$)** δ 7.82 – 7.71 (m, 2H), 7.34 – 7.19 (m, 2H), 7.12 – 7.02 (m, 1H), 6.58 (d, *J* = 8.5 Hz, 1H), 5.42 (d, *J* = 8.5 Hz, 1H), 3.12 (d, *J* = 11.7 Hz, 6H).
- **$^{13}$C NMR (101 MHz, $CD_3CN$)** δ 139.62, 139.34, 132.05, 130.35, 129.41, 129.06, 128.79, 125.92, 125.84, 125.53, 115.97, 112.07, 42.55, 42.48.
- **HRMS (ESI):** calcd. for $C_{10}H_{14}NOS^{+}$ $[M+H]^{+}$ 196.0796; found: 196.0792

**3-phenyl-1-(prop-1-en-2-yl)-1*H*-pyrazole (36)**

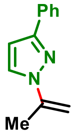


36

Following general procedure **V**, 2-bromoprop-1-ene (28 µL, 0.30 mmol, 1.5 equiv), 3-phenyl-1*H*-pyrazole (29 mg, 0.20 mmol, 1.0 equiv), $^{t}$BuNH$_2$ (44 µL, 0.4 mmol, 2.0 equiv), and 4CzIPN (0.8 mg, 0.5 mol%), $NiBr_2$·glyme (3.2 mg, 5.0 mol%), dOMebpy (4.3 mg, 10 mol%) were stirred for 48 h. After the workup, the organic phases were concentrated under vacuum. The crude product was purified by flash chromatography using silica, EtOAc:Hexane to afford the product **36** (23 mg, 63%)

- **$^{1}$H NMR (400 MHz, $CD_3CN$)** δ 8.01 – 7.78 (m, 2H), 7.53 – 7.37 (m, 2H), 7.34 – 7.09 (m, 1H), 6.75 (d, *J* = 2.6 Hz, 1H), 5.46 (s, 1H), 4.74 (q, *J* = 1.4 Hz, 1H), 2.30 (d, *J* = 1.3 Hz, 3H).
- **$^{13}$C NMR (101 MHz, $CD_3CN$)** δ 152.8, 142.2, 134.2, 129.7, 129.0, 126.5, 105.0, 99.8, 19.4.
- **HRMS (ESI):** calcd. for $C_{12}H_{12}N_2^{+}$ $[M]^{+}$ 184.0995; found: 184.0992

**3-Phenyl-1-(prop-1-en-1-yl)-1H-pyrazole (37)**

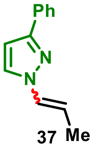


37

Following general procedure **V**, (*E*)-1-bromoprop-1-ene (26 µL, 0.30 mmol, 1.5 equiv), 3-phenyl-1*H*-pyrazole (29 mg, 0.20 mmol, 1.0 equiv), $^{t}$BuNH$_2$ (44 µL, 0.4 mmol, 2.0 equiv), and 4CzIPN (0.8 mg, 0.5 mol%), $NiBr_2$·glyme (3.2 mg, 5.0 mol%), dOMebpy (4.3 mg, 10 mol%) were stirred for 48 h. After the workup, the organic phases were concentrated under vacuum. The crude product was purified by flash chromatography using silica, EtOAc:Hexane to afford the product **37** (12 mg, 33%, 50:50 *dr*)

- **$^{1}$H NMR (400 MHz, $CD_3CN$)** δ 7.84 (td, *J* = 8.2, 1.3 Hz, 2H), 7.70 (dd, *J* = 12.6, 2.5 Hz, 1H), 7.41 (dddt, *J* = 9.7, 8.3, 3.5, 1.4 Hz, 2H), 7.36 – 7.29 (m, 1H), 6.94 (dq, *J* = 14.0, 1.7 Hz, 0.5H), 6.82 (dq, *J* = 9.4, 1.8 Hz, 0.5H), 6.71 (dd, *J* = 12.1, 2.5 Hz, 1H), 6.18 (dq, *J* = 13.9, 6.9 Hz, 0.5H), 5.37 (dq, *J* = 9.3, 7.3 Hz, 0.5H), 2.05 (dd, *J* = 7.3, 1.8 Hz, 1.5H), 1.82 (dd, *J* = 7.0, 1.8 Hz, 1.5H).
- **$^{13}$C NMR (101 MHz, $CD_3CN$)** δ 152.54, 152.45, 134.25, 134.21, 133.12, 130.41, 129.68, 128.87, 128.85, 128.80, 127.47, 126.45, 126.42, 115.49, 113.72, 104.59, 104.10, 14.84, 13.34.
- **HRMS (ESI):** calcd. for $C_{12}H_{12}N_2^{+}$ $[M]^{+}$ 184.0995; found: 184.0991

**(*E*)-3-phenyl-1-(2-(trimethylsilyl)vinyl)-1H-pyrazole (38)**

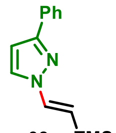


38

Following general procedure **V**, (*E*)-(2-bromovinyl)trimethylsilane (36 mg, 0.30 mmol, 1.5 equiv), 3-phenyl-1*H*-pyrazole (29 mg, 0.20 mmol, 1.0 equiv), ${}^{t}$BuNH$_2$ (44 µL, 0.4 mmol, 2.0 equiv), and 4CzIPN (0.8 mg, 0.5 mol%), NiBr$_2$·glyme (3.2 mg, 5.0 mol%), dOMebpy (4.3 mg, 10 mol%) were stirred for 48 h. After the workup, the organic phases were concentrated under vacuum. The crude product was purified by flash chromatography using silica, EtOAc:Hexane to afford the product **38** (20 mg, 42%)

- **$^{1}$H NMR (400 MHz, CD$_3$CN)** δ 7.88 – 7.83 (m, 2H), 7.78 (d, *J* = 2.5 Hz, 1H), 7.42 (tt, *J* = 6.9, 0.8 Hz, 2H), 7.37 – 7.29 (m, 1H), 7.12 (d, *J* = 16.9 Hz, 1H), 6.75 (d, *J* = 2.5 Hz, 1H), 6.16 (d, *J* = 16.9 Hz, 1H), 0.18 (s, 9H).
- **$^{13}$C NMR (101 MHz, CD$_3$CN)** δ 153.25, 137.70, 134.02, 130.68, 129.09, 126.54, 112.26, 105.39, -1.03.
- **HRMS (ESI):** calcd. for $C_{14}H_{19}N_2Si^+$ [M+H]$^+$ 243.1318; found: 243.1318

**1-(2-methylprop-1-en-1-yl)-3-phenyl-1H-pyrazole (39)**

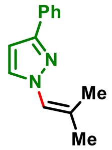


39

Following general procedure **V**, 1-bromo-2-methylprop-1-ene (31 µL, 0.30 mmol, 1.5 equiv), 3-phenyl-1*H*-pyrazole (29 mg, 0.20 mmol, 1.0 equiv), ${}^{t}$BuNH$_2$ (44 µL, 0.4 mmol, 2.0 equiv), and 4CzIPN (0.8 mg, 0.5 mol%), NiBr$_2$·glyme (3.2 mg, 5.0 mol%), dOMebpy (4.3 mg, 10 mol%) were stirred for 48 h. After the workup, the organic phases were concentrated under vacuum. The crude product was purified by flash chromatography using silica, EtOAc:Hexane to afford the product **39** (24 mg, 61%)

- **$^{1}$H NMR (400 MHz, CD$_3$CN)** δ 7.86 – 7.79 (m, 2H), 7.60 (d, *J* = 2.4 Hz, 1H), 7.44 – 7.37 (m, 2H), 7.34 – 7.28 (m, 1H), 6.74 – 6.66 (m, 2H), 1.94 (q, *J* = 1.8, 1.3 Hz, 3H), 1.86 (d, *J* = 1.5 Hz, 3H).
- **$^{13}$C NMR (101 MHz, CD$_3$CN)** δ 151.93, 134.45, 132.99, 129.65, 129.52, 128.67, 126.34, 123.25, 103.61, 23.24, 18.52.
- **HRMS (ESI):** calcd. for $C_{13}H_{14}N_2^+$ [M]$^+$ 198.11515; found: 198.11494

**1-(cyclohex-1-en-1-yl)-3-phenyl-1H-pyrazole (40)**

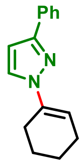


40

Following general procedure **V**, 1-bromocyclohex-1-ene (35 µL, 0.30 mmol, 1.5 equiv), 3-phenyl-1*H*-pyrazole (29 mg, 0.20 mmol, 1.0 equiv), $^{t}$BuNH$_2$ (44 µL, 0.4 mmol, 2.0 equiv), and 4CzIPN (0.8 mg, 0.5 mol%), NiBr$_2$·glyme (3.2 mg, 5.0 mol%), dOMebpy (4.3 mg, 10 mol%) were stirred for 96 h. After the workup, the organic phases were concentrated under vacuum. The crude product was purified by flash chromatography using silica, EtOAc:Hexane to afford the product **40** (33 mg, 67%)

- **$^{1}$H NMR (400 MHz, $CD_3CN$)** δ 7.89 – 7.82 (m, 2H), 7.80 – 7.65 (m, 1H), 7.45 – 7.37 (m, 2H), 7.34 – 7.28 (m, 1H), 6.69 (d, *J* = 2.5 Hz, 1H), 6.21 (tt, *J* = 4.1, 1.6 Hz, 1H), 2.58 (qd, *J* = 4.2, 1.9 Hz, 2H), 2.27 – 2.15 (m, 2H), 1.82 (p, *J* = 6.2 Hz, 2H), 1.68 – 1.56 (m, 2H).
- **$^{13}$C NMR (101 MHz, $CD_3CN$)** δ 152.0, 137.5, 134.6, 129.7, 128.74, 128.7, 126.4, 114.2, 104.2, 26.4, 24.7, 23.2, 22.7.
- **HRMS (ESI):** calcd. for $C_{15}H_{16}N_2^+$ [M]$^+$ 224.1308; found: 224.1311

**3-phenyl-1-styryl-1H-pyrazole (41)**

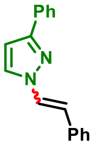

41

Following general procedure **V**, (2-bromovinyl)benzene (55 mg, 0.30 mmol, 1.5 equiv), 3-phenyl-1*H*-pyrazole (29 mg, 0.20 mmol, 1.0 equiv), $^{t}$BuNH$_2$ (44 µL, 0.4 mmol, 2.0 equiv), and 4CzIPN (0.8 mg, 0.5 mol%), NiBr$_2$·glyme (3.2 mg, 5.0 mol%), dOMebpy (4.3 mg, 10 mol%) were stirred for 48 h. After the workup, the organic phases were concentrated under vacuum. The crude product was purified by flash chromatography using silica, EtOAc:Hexane to afford the product **41** (37 mg, 75%, 60:40 *dr*)

- **$^{1}$H NMR (400 MHz, $CD_3CN$)** δ 7.98 – 7.89 (m, 0.5H), 7.84 – 7.76 (m, 2H), 7.67 (d, *J* = 14.4 Hz, 0.5H), 7.54 – 7.15 (m, 9H), 7.00 (d, *J* = 9.8 Hz, 0.5H), 6.80 (d, *J* = 2.5 Hz, 0.5H), 6.63 (d, *J* = 2.5 Hz, 0.5H), 6.31 (d, *J* = 9.8 Hz, 0.5H).
- **$^{13}$C NMR (101 MHz, $CD_3CN$)** δ 153.5, 152.7, 136.4, 135.6, 134.0, 133.9, 132.6, 131.8, 129.9, 129.8, 129.75, 129.7, 129.3, 129.2, 129.0, 128.8, 128.4, 127.6, 127.3, 127.1, 126.6, 126.5, 120.2, 117.2, 105.6, 104.9.
- **HRMS (ESI):** calcd. for $C_{17}H_{14}N_2^+$ [M]$^+$ 246.1151; found 246.1143

**1-(prop-1-en-2-yl)-1*H*-indazole (42)**

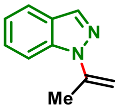

42

Following general procedure **V**, 2-bromoprop-1-ene (28 µL, 0.30 mmol, 1.5 equiv), 3-phenyl-1*H*-indazole (24 mg, 0.20 mmol, 1.0 equiv), $^{t}$BuNH$_2$ (44 µL, 0.4 mmol, 2.0 equiv), and 4CzIPN (0.8 mg, 0.5 mol%), NiBr$_2$·glyme (3.2 mg, 5.0 mol%), dOMebpy (4.3 mg, 10 mol%) were stirred for 48 h. After the workup, the organic phases were concentrated under vacuum. The crude product was purified by flash chromatography using silica, EtOAc:Hexane to afford the product **42** (21 mg, 66%)

- **$^{1}$H NMR (400 MHz, $CD_3CN$)** δ 8.10 (d, *J* = 0.9 Hz, 1H), 7.79 (ddt, *J* = 8.2, 2.1, 1.0 Hz, 2H), 7.44 (ddd, *J* = 8.4, 6.9, 1.2 Hz, 1H), 7.21 (ddd, *J* = 8.0, 6.9, 0.8 Hz, 1H), 5.26 (s, 1H), 5.08 (d, *J* = 1.5 Hz, 1H), 2.37 (d, *J* = 1.3 Hz, 3H).
- **$^{13}$C NMR (101 MHz, $CD_3CN$)** δ 143.4, 139.5, 135.3, 128.0, 126.3, 122.5, 122.2, 112.8, 102.9, 21.5.
- **HRMS (ESI):** calcd. for $C_{10}H_{10}N_2^+$ $[M]^+$ 158.0836; found 158.0838

**1-(cyclohex-1-en-1-yl)-1*H*-indazole (43)**

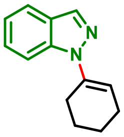

43

Following general procedure **V**, 1-bromocyclohex-1-ene (35 µL, 0.30 mmol, 1.5 equiv), 1*H*-indazole (24 mg, 0.20 mmol, 1.0 equiv), $^{t}$BuNH$_2$ (44 µL, 0.4 mmol, 2.0 equiv), and 4CzIPN (0.8 mg, 0.5 mol%), $NiBr_2$·glyme (3.2 mg, 5.0 mol%), dOMebpy (4.3 mg, 10 mol%) were stirred for 96 h. After the workup, the organic phases were concentrated under vacuum. The crude product was purified by flash chromatography using silica, EtOAc:Hexane to afford the product **43** (25 mg, 63%)

- **$^{1}$H NMR (400 MHz, $CD_3CN$)** δ 8.04 (d, *J* = 1.0 Hz, 1H), 7.75 (dt, *J* = 8.1, 1.1 Hz, 1H), 7.67 (dq, *J* = 8.6, 0.9 Hz, 1H), 7.38 (ddd, *J* = 8.3, 6.9, 1.1 Hz, 1H), 7.15 (ddd, *J* = 7.9, 6.9, 0.9 Hz, 1H), 6.01 (td, *J* = 4.0, 2.0 Hz, 1H), 2.60 (tddd, *J* = 6.6, 4.9, 3.2, 1.9 Hz, 2H), 2.36 – 2.23 (m, 2H), 1.89 – 1.81 (m, 2H), 1.76 – 1.68 (m, 2H).
- **$^{13}$C NMR (75 MHz, $CD_3CN$)** δ 139.5, 137.9, 134.5, 127.5, 125.6, 122.0, 121.9, 119.6, 112.1, 28.4, 25.0, 23.5, 22.8.
- **HRMS (ESI):** calcd. for $C_{13}H_{14}N_2^+$ $[M]^+$ 198.1151; found 198.1147

**1-styryl-1*H*-indazole (44)**

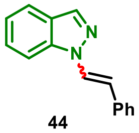

44

Following general procedure **V**, (2-bromovinyl)benzene (55 mg, 0.30 mmol, 1.5 equiv), 1*H*-indazole (24 mg, 0.20 mmol, 1.0 equiv), $^{t}$BuNH$_2$ (44 µL, 0.4 mmol, 2.0 equiv), and 4CzIPN (0.8 mg, 0.5 mol%), $NiBr_2$·glyme (3.2 mg, 5.0 mol%), dOMebpy (4.3 mg, 10 mol%) were stirred for 48 h. After the workup, the organic phases were concentrated under vacuum. The crude product was purified by flash chromatography using silica, EtOAc:Hexane to afford the product **44** (27 mg, 61%, 60:40 *dr*)

- **$^{1}$H NMR (400 MHz, $CD_3CN$)** δ 8.19 (s, 0.4H), 8.12 (d, *J* = 1.0 Hz, 0.6H), 8.00 (d, *J* = 14.2 Hz, 0.5H), 7.85 – 7.73 (m, 1.5H), 7.63 – 7.56 (m, 0.5H), 7.50 (ddd, *J* = 8.3, 6.9, 1.1 Hz, 0.5H), 7.38 (dd, *J* = 8.5, 7.0 Hz, 0.5H), 7.30 – 7.22 (m, 2H), 7.20 – 7.06 (m, 5H), 6.49 (d, *J* = 9.3 Hz, 0.5H).
- **$^{13}$C NMR (101 MHz, $CD_3CN$)** δ 139.9, 137.1, 136.3, 135.9, 129.9, 129.7, 128.6, 128.3, 128.0, 127.8, 127.0, 125.8, 125.3, 124.7, 123.0, 122.5, 122.3, 122.0, 115.6, 111.4, 110.7. (Five carbons are not resolved in 101 MHz NMR)
- **HRMS (ESI):** calcd. for $C_{15}H_{12}N_2^+$ $[M]^+$ 220.0995; found 220.0989

**1-(prop-1-en-2-yl)-1*H*-benzo[*d*]imidazole (45)**

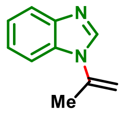

45

Following general procedure **V**, 2-bromoprop-1-ene (28 µL, 0.30 mmol, 1.5 equiv), 1*H*-benzo[*d*]imidazole (24 mg, 0.20 mmol, 1.0 equiv), $^t$BuNH$_2$ (44 µL, 0.4 mmol, 2.0 equiv), and 4CzIPN (0.8 mg, 0.5 mol%), NiBr$_2$·glyme (3.2 mg, 5.0 mol%), dOMebpy (4.3 mg, 10 mol%) were stirred for 48 h. After the workup, the organic phases were concentrated under vacuum. The crude product was purified by flash chromatography using silica, EtOAc:Hexane to afford the product **45** (9 mg, 28%)

- **$^1$H NMR (400 MHz, CD$_3$CN)** δ 8.11 (s, 1H), 7.78 – 7.58 (m, 2H), 7.44 – 7.13 (m, 2H), 5.28 (t, *J* = 0.7 Hz, 1H), 5.18 (q, *J* = 1.4 Hz, 1H), 2.33 (dd, *J* = 1.4, 0.7 Hz, 3H).
- **$^{13}$C NMR (101 MHz, CD$_3$CN)** δ 143.1, 140.0, 124.3, 123.4, 121.0, 113.0, 107.5, 21.6.
- **HRMS (ESI):** calcd. for $C_{10}H_{10}N_2^+$ [M]$^+$ 158.0838; found 158.0841

**1-(cyclohex-1-en-1-yl)-1*H*-benzo[*d*]imidazole (46)**

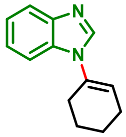

46

Following general procedure **V**, 1-bromocyclohex-1-ene (35 µL, 0.30 mmol, 1.5 equiv), 1*H*-benzo[*d*]imidazole (24 mg, 0.20 mmol, 1.0 equiv), $^t$BuNH$_2$ (44 µL, 0.4 mmol, 2.0 equiv), and 4CzIPN (0.8 mg, 0.5 mol%), NiBr$_2$·glyme (3.2 mg, 5.0 mol%), dOMebpy (4.3 mg, 10 mol%) were stirred for 96 h. After the workup, the organic phases were concentrated under vacuum. The crude product was purified by flash chromatography using silica, EtOAc:Hexane to afford the product **46** (11 mg, 27%)

- **$^1$H NMR (400 MHz, CD$_3$CN)** δ 7.99 (s, 1H), 7.75 – 7.63 (m, 1H), 7.60 – 7.47 (m, 1H), 7.35 – 7.19 (m, 2H), 6.01 (tt, *J* = 3.9, 1.7 Hz, 1H), 2.49 (ttd, *J* = 6.3, 2.6, 1.7 Hz, 2H), 2.28 (dddd, *J* = 8.9, 6.4, 4.0, 2.6 Hz, 2H), 1.91 – 1.83 (m, 2H), 1.77 – 1.67 (m, 2H).
- **$^{13}$C NMR (101 MHz, CD$_3$CN)** δ 145.0, 143.1, 134.6, 123.9, 123.6, 123.0, 120.8, 112.3, 29.1, 25.2, 23.4, 22.4.
- **HRMS (ESI):** calcd. for $C_{13}H_{14}N_2^+$ [M]$^+$ 198.1151; found 198.1152

**1-styryl-1*H*-benzo[*d*]imidazole (47)**

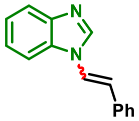

47

Following general procedure **V**, (2-bromovinyl)benzene (55 mg, 0.30 mmol, 1.5 equiv), 1*H*-benzo[*d*]imidazole (24 mg, 0.20 mmol, 1.0 equiv), $^t$BuNH$_2$ (44 µL, 0.4 mmol, 2.0 equiv), and 4CzIPN (0.8 mg, 0.5 mol%), NiBr$_2$·glyme (3.2 mg, 5.0 mol%), dOMebpy (4.3 mg, 10 mol%) were stirred for 48 h. After the workup, the organic phases were concentrated under vacuum. The crude product was purified by flash chromatography using silica, EtOAc:Hexane to afford the product **47** (36 mg, 82%, 90:10 *dr*).

- **$^{1}$H NMR (400 MHz, $CD_3CN$)** δ 7.80 (d, *J* = 11.5 Hz, 1H), 7.76 – 7.66 (m, 1H), 7.59 – 7.53 (m, 0.5H), 7.44 – 7.35 (m, 0.5H), 7.34 – 7.29 (m, 1H), 7.27 – 7.18 (m, 4H), 7.12 – 7.03 (m, 2H), 6.98 (d, *J* = 9.0 Hz, 1H), 6.68 (d, *J* = 9.0 Hz, 1H).
- **$^{13}$C NMR (101 MHz, $CD_3CN$)** δ 145.1, 144.4, 143.4, 142.5, 136.3, 135.2, 134.0, 129.9, 129.6, 129.4, 129.2, 128.7, 127.2, 126.8, 124.6, 124.3, 123.8, 123.5, 123.0, 122.0, 121.0, 120.8, 119.7, 112.0, 111.8.
- **HRMS (ESI):** calcd. for $C_{15}H_{12}N_2^+$ $[M]^+$ 220.0995; found 220.0990

**1-(Prop-1-en-2-yl)-1H-indole (48)**

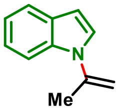

48

Following general procedure **VI**, 2-bromoprop-1-ene (28 µL, 0.30 mmol, 1.5 equiv), indole (24 mg, 0.20 mmol, 1.0 equiv), TMG (24 mg, 0.20 mmol, 1.0 equiv) and 4CzIPN (0.8 mg, 0.5 mol%), $NiBr_2$·glyme (3.2 mg, 5.0 mol%), dOMebpy (4.3 mg, 10 mol%) were stirred. After 24h one equivalent of TMG (24 mg, 0.20 mmol, 1.0 equiv) was added to the reaction mixture and stirred for another 24h. After the workup, the organic phases were concentrated under vacuum. The crude product was purified by flash chromatography using silica, EtOAc:Hexane to afford the product **48** (21 mg, 67%).

- **$^{1}$H NMR (400 MHz, $CD_3CN$)** δ 7.72 – 7.55 (m, 2H), 7.35 (d, *J* = 3.4 Hz, 1H), 7.20 (ddd, *J* = 8.4, 7.0, 1.3 Hz, 1H), 7.10 (ddd, *J* = 8.0, 7.0, 1.0 Hz, 1H), 6.56 (dd, *J* = 3.4, 0.9 Hz, 1H), 5.21 – 5.04 (m, 2H), 2.27 (d, *J* = 1.3 Hz, 3H).
- **$^{13}$C NMR (101 MHz, $CD_3CN$)** δ 142.05, 136.29, 130.50, 127.58, 123.11, 121.79, 121.10, 112.77, 105.83, 103.67, 22.10.
- **HRMS (ESI):** calcd. for $C_{11}H_{11}N^+$ $[M]^+$ 157.0886; found: 157.0884

**1-(2-Methylprop-1-en-1-yl)-1H-indole (49)**

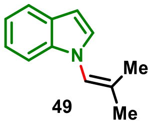

49

Following general procedure **VI**, 1-bromo-2-methylprop-1-ene (31µL, 0.30 mmol, 1.5 equiv), indole (24 mg, 0.20 mmol, 1.0 equiv), TMG (24 mg, 1.0 equiv) and 4CzIPN (0.8 mg, 0.5 mol%), $NiBr_2$·glyme (3.2 mg, 5.0 mol%), dOMebpy (4.3 mg, 10 mol%) were stirred. After 48h one equivalent of TMG (24 mg, 1.0 equiv) was added to the reaction mixture and stirred for another 48h. After the workup, the organic phases were concentrated under vacuum. The crude product was purified by flash chromatography using silica, EtOAc:Hexane to afford the product **49** (18 mg, 52%)

- **$^{1}$H NMR (400 MHz, $CD_3CN$)** δ 7.58 (dt, *J* = 7.8, 1.1 Hz, 1H), 7.27 (dq, *J* = 8.2, 1.0 Hz, 1H), 7.21 – 7.15 (m, 2H), 7.08 (ddd, *J* = 8.0, 6.9, 1.1 Hz, 1H), 6.65 (p, *J* = 1.5 Hz, 1H), 6.53 (dd, *J* = 3.2, 0.9 Hz, 1H), 1.92 (d, *J* = 1.5 Hz, 3H), 1.66 (d, *J* = 1.5 Hz, 3H).
- **$^{13}$C NMR (101 MHz, $CD_3CN$)** δ 137.39, 133.36, 129.36, 129.07, 122.70, 121.49, 120.69, 120.57, 111.17, 102.71, 22.46, 18.25.
- **HRMS (ESI):** calcd. for $C_{12}H_{13}N^+$ $[M]^+$ 171.1042; found: 171.1042

**1-styryl-1H-indole (50)**

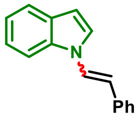


50

Following general procedure **VI**, (2-bromovinyl)benzene (55 mg, 0.30 mmol, 1.5 equiv), indole (24 mg, 0.20 mmol, 1.0 equiv), TMG (24 mg, 1.0 equiv) and 4CzIPN (0.8 mg, 0.5 mol%), $NiBr_2$·glyme (3.2 mg, 5.0 mol%), dOMebpy (4.3 mg, 10 mol%) were stirred. After 24h one equivalent of TMG (24 mg, 1.0 equiv) was added to the reaction mixture and stirred for another 24h. After the workup, the organic phases were concentrated under vacuum. The crude product was purified by flash chromatography using silica, EtOAc:Hexane to afford the product **50** (32 mg, 73%, 55:45 *dr*)

- **$^1$H NMR (400 MHz, $CDCl_3$)** δ 7.79 – 7.67 (m, 1.5H), 7.62 (dd, *J* = 8.3, 1.0 Hz, 0.5H), 7.56 (d, *J* = 3.4 Hz, 0.5H), 7.54 – 7.48 (m, 1H), 7.47 – 7.41 (m, 1.5H), 7.39 – 7.23 (m, 5H), 7.10 (d, *J* = 3.4 Hz, 0.5H), 7.01 (d, *J* = 9.2 Hz, 0.5H), 6.79 – 6.70 (m, 1H), 6.57 (dd, *J* = 3.4, 0.9 Hz, 0.5H), 6.34 (d, *J* = 9.2 Hz, 0.5H).
- **$^{13}$C NMR (101 MHz, $CDCl_3$)** δ 136.1, 135.8, 135.7, 134.9, 129.2, 128.8, 128.7, 128.5, 128.4, 127.5, 127.1, 126.9, 125.7, 123.7, 123.5, 123.3, 122.8, 122.4, 121.2, 120.9, 120.9, 120.7, 119.5, 114.0, 110.1, 109.6, 105.3, 103.9.
- **HRMS (ESI):** calcd. for $C_{16}H_{13}N^+$ $[M]^+$ 219.1042; found: 219.1038

**9-(Prop-1-en-2-yl)-9H-carbazole (51)**

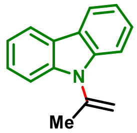


51

Following general procedure **VI**, 2-bromoprop-1-ene (28 µL, 0.30 mmol, 1.5 equiv), carbazole (34 mg, 0.20 mmol, 1.0 equiv), TMG (24 mg, 1.0 equiv) and 4CzIPN (0.8 mg, 0.5 mol%), $NiBr_2$·glyme (3.2 mg, 5.0 mol%), dOMebpy (4.3 mg, 10 mol%) were stirred. After 24h one equivalent of TMG (24 mg, 1.0 equiv) was added to the reaction mixture and stirred for another 24h. After the workup, the organic phases were concentrated under vacuum. The crude product was purified by flash chromatography using silica, EtOAc:Hexane to afford the product **51** (21 mg, 51%).

- **$^1$H NMR (400 MHz, $CD_3CN$)** δ 8.12 (dt, *J* = 7.8, 1.0 Hz, 2H), 7.53 – 7.40 (m, 4H), 7.24 (ddd, *J* = 8.0, 6.8, 1.4 Hz, 2H), 5.64 (q, *J* = 1.4 Hz, 1H), 5.33 (s, 1H), 2.20 (d, *J* = 1.1 Hz, 3H).
- **$^{13}$C NMR (101 MHz, $CD_3CN$)** δ 141.17, 140.87, 126.98, 123.98, 121.18, 120.55, 115.70, 111.13, 20.46.
- **HRMS (ESI):** calcd. for $C_{15}H_{13}N^+$ $[M]^+$ 207.1042; found: 207.1036

**9-Styryl-9H-carbazole (52)**

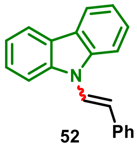


52

Following general procedure **VI**, (2-bromovinyl)benzene (55.0 mg, 0.30 mmol, 1.5 equiv), carbazole (34 mg, 0.20 mmol, 1.0 equiv), TMG (24 mg, 1.0 equiv) and 4CzIPN (0.8 mg, 0.5 mol%), $NiBr_2$·glyme (3.2 mg, 5.0 mol%), dOMebpy (4.3 mg, 10 mol%) were stirred. After 24h one equivalent of TMG (24 mg, 1.0

equiv) was added to the reaction mixture and stirred for another 24h. After the workup, the organic phases were concentrated under vacuum. The crude product was purified by flash chromatography using silica, EtOAc:Hexane to afford the product **52** (32 mg, 73%, 60:40 *dr*)

- **$^1$H NMR (400 MHz, $CD_3CN$)** δ 8.12 (ddt, *J* = 7.5, 5.4, 1.0 Hz, 2H), 7.92 – 7.80 (m, 2H), 7.65 – 7.58 (m, 1.5H), 7.50 (ddd, *J* = 8.4, 7.2, 1.3 Hz, 1.5H), 7.43 – 7.37 (m, 1.5H), 7.34 – 6.95 (m, 6H), 6.76 (d, *J* = 8.7 Hz, 0.5H).
- **$^{13}$C NMR (101 MHz, $CD_3CN$)** δ 140.37, 140.06, 137.40, 136.05, 129.75, 129.44, 129.22, 128.76, 128.06, 127.85, 127.40, 126.92, 126.88, 124.83, 124.60, 124.46, 123.01, 121.82, 121.21, 121.16, 120.03, 111.94, 111.79.
- **HRMS (ESI):** calcd. for $C_{20}H_{15}N^+$ $[M]^+$ 269.11990; found: 269.11991

**(prop-1-en-2-yloxy)benzene (53)**

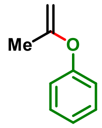


**53**

Following general procedure **V**, 2-bromoprop-1-ene (28 µL, 0.30 mmol, 1.5 equiv), phenol (19 mg, 0.20 mmol, 1.0 equiv), $^t$BuNH$_2$ (44 µL, 0.4 mmol, 2.0 equiv), and 4CzIPN (0.8 mg, 0.5 mol%), NiBr$_2$·glyme (3.2 mg, 5.0 mol%), dOMebpy (4.3 mg, 10 mol%) were stirred for 48 h. After the workup, the organic phases were concentrated under vacuum. The crude product was purified by flash chromatography using silica, EtOAc:Hexane to afford the product **53** (18 mg, 67%).

- **$^1$H NMR (400 MHz, $CD_3CN$)** δ 7.42 – 7.30 (m, 2H), 7.22 – 7.11 (m, 1H), 7.07 – 6.96 (m, 2H), 4.20 (p, *J* = 1.0 Hz, 1H), 3.89 (d, *J* = 1.5 Hz, 1H), 2.13 (s, 3H).
- **$^{13}$C NMR (75 MHz, $CD_3CN$)** δ 160.8, 156.4, 130.7, 125.0, 121.5, 90.4, 20.1.
- **HRMS (ESI):** calcd. for $C_9H_{10}O^+$ $[M]^+$ 134.0726; found: 134.0726

**(cyclohex-1-en-1-yloxy)benzene (54)**

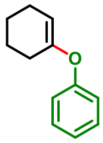


**54**

Following general procedure **V**, 1-bromocyclohex-1-ene (35 µL, 0.30 mmol, 1.5 equiv), phenol (19 mg, 0.20 mmol, 1.0 equiv), $^t$BuNH$_2$ (44 µL, 0.4 mmol, 2.0 equiv), and 4CzIPN (0.8 mg, 0.5 mol%), NiBr$_2$·glyme (3.2 mg, 5.0 mol%), dOMebpy (4.3 mg, 10 mol%) were stirred for 96 h. After the workup, the organic phases were concentrated under vacuum. The crude product was purified by flash chromatography using silica, EtOAc:Hexane to afford the product **54** (17 mg, 49%)

- **$^1$H NMR (400 MHz, $CD_3CN$)** δ 7.53 – 7.21 (m, 2H), 7.11 – 6.98 (m, 1H), 6.99 – 6.65 (m, 2H), 5.02 (tt, *J* = 4.0, 1.4 Hz, 1H), 2.12 – 2.09 (m, 2H), 2.06 (dtd, *J* = 8.5, 4.3, 2.1 Hz, 2H), 1.79 – 1.69 (m, 2H), 1.58 (ddt, *J* = 9.2, 6.0, 3.2 Hz, 2H).
- **$^{13}$C NMR (101 MHz, $CD_3CN$)** δ 157.5, 153.9, 130.6, 123.5, 119.3, 108.5, 27.2, 24.3, 23.6, 22.9.
- **HRMS (ESI):** calcd. for $C_{12}H_{14}O^+$ $[M]^+$ 174.1039; found: 174.1043

**(2-phenoxyvinyl)benzene (55)**

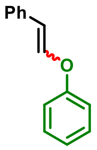

55

Following general procedure **V**, (2-bromovinyl)benzene (55 mg, 0.30 mmol, 1.5 equiv), phenol (19 mg, 0.20 mmol, 1.0 equiv),, $^{t}BuNH_2$ (44 µL, 0.4 mmol, 2.0 equiv), and 4CzIPN (0.8 mg, 0.5 mol%), $NiBr_2$·glyme (3.2 mg, 5.0 mol%), dOMebpy (4.3 mg, 10 mol%) were stirred for 48 h. After the workup, the organic phases were concentrated under vacuum. The crude product was purified by flash chromatography using silica, EtOAc:Hexane to afford the product **55** (17 mg, 45%, 60:40 *dr*)

- **$^{1}H$ NMR (400 MHz, $CD_3CN$)** δ 7.75 – 7.64 (m, 0.7H), 7.52 – 7.45 (m, 1.3H), 7.42 – 6.99 (m, 8H), 6.77 – 6.66 (m, 1H), 6.34 (d, *J* = 12.4 Hz, 0.3H), 5.68 (d, *J* = 6.9 Hz, 0.7H).
- **$^{13}C$ NMR (101 MHz, $CD_3CN$)** δ 158.1, 144.6, 142.8, 138.4, 133.7, 130.9, 130.3, 129.7, 129.6, 129.4, 128.6, 127.7, 127.4, 126.7, 124.5, 124.3, 117.7, 117.6, 114.1, 110.9.
- **HRMS (ESI):** calcd. for $C_{14}H_{12}O^+$ $[M]^+$ 196.0882; found: 196.0883

**4-(prop-1-en-2-yloxy)-1,1'-biphenyl (56)**

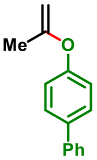

56

Following general procedure **V**, 2-bromoprop-1-ene (28 µL, 0.30 mmol, 1.5 equiv), [1,1'-biphenyl]-4-ol (34 mg, 0.20 mmol, 1.0 equiv), $^{t}BuNH_2$ (44 µL, 0.4 mmol, 2.0 equiv), and 4CzIPN (0.8 mg, 0.5 mol%), $NiBr_2$·glyme (3.2 mg, 5.0 mol%), dOMebpy (4.3 mg, 10 mol%) were stirred for 48 h. After the workup, the organic phases were concentrated under vacuum. The crude product was purified by flash chromatography using silica, EtOAc:Hexane to afford the product **56** (27 mg, 64%).

- **$^{1}H$ NMR (400 MHz, $CD_3CN$)** δ 7.67 – 7.55 (m, 4H), 7.47 – 7.39 (m, 2H), 7.37 – 7.23 (m, 1H), 7.17 – 6.99 (m, 2H), 4.26 (q, *J* = 1.1 Hz, 1H), 4.01 (d, *J* = 1.5 Hz, 1H), 1.98 (d, *J* = 1.0 Hz, 3H).
- **$^{13}C$ NMR (101 MHz, $CD_3CN$)** δ 160.6, 156.0, 141.2, 137.6, 129.9, 129.2, 128.2, 127.7, 121.7, 91.0, 20.1.
- **HRMS (ESI):** Not detected in HRMS (ESI, CI).

**1-methoxy-4-(prop-1-en-2-yloxy)benzene (57)**

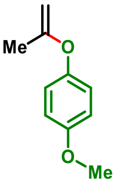

57

Following general procedure **V**, 2-bromoprop-1-ene (28 µL, 0.30 mmol, 1.5 equiv), 4-methoxyphenol (25 mg, 0.20 mmol, 1.0 equiv), $^{t}BuNH_2$ (44 µL, 0.4 mmol, 2.0 equiv), and 4CzIPN (0.8 mg, 0.5 mol%), $NiBr_2$·glyme (3.2 mg, 5.0 mol%), dOMebpy (4.3 mg, 10 mol%) were stirred for 48 h. After the workup, the

organic phases were concentrated under vacuum. The crude product was purified by flash chromatography using silica, EtOAc:Hexane to afford the product **57** (27 mg, 82%).

- **$^1$H NMR (400 MHz, $CD_3CN$)** δ 6.97 – 6.93 (m, 2H), 6.91 – 6.87 (m, 2H), 4.08 (dq, *J* = 1.8, 1.0 Hz, 1H), 3.75 (s, 3H), 3.74 (d, *J* = 1.5 Hz, 1H), 1.94 (d, *J* = 0.9 Hz, 3H).
- **$^{13}$C NMR (101 MHz, $CD_3CN$)** δ 161.8, 157.4, 149.6, 123.0, 115.6, 88.2, 56.2, 20.3.
- **HRMS (ESI):** calcd. for $C_{10}H_{12}O_2^+$ [M]$^+$ 164.0831; found: 164.0829

**1-(cyclohex-1-en-1-yloxy)-4-methoxybenzene (58)**

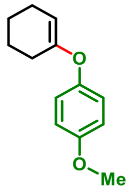


58

Following general procedure **V**, 1-bromocyclohex-1-ene (35 µL, 0.30 mmol, 1.5 equiv), 4-methoxyphenol (25 mg, 0.20 mmol, 1.0 equiv), $^t$BuNH$_2$ (44 µL, 0.4 mmol, 2.0 equiv), and 4CzIPN (0.8 mg, 0.5 mol%), NiBr$_2$·glyme (3.2 mg, 5.0 mol%), dOMebpy (4.3 mg, 10 mol%) were stirred for 96 h. After the workup, the organic phases were concentrated under vacuum. The crude product was purified by flash chromatography using silica, EtOAc:Hexane to afford the product **58** (23 mg, 56%).

- **$^1$H NMR (400 MHz, $CD_3CN$)** δ 6.95 – 6.74 (m, 4H), 4.78 (tt, *J* = 4.0, 1.4 Hz, 1H), 3.74 (s, 3H), 2.12 (dddd, *J* = 7.7, 6.2, 2.3, 1.2 Hz, 2H), 2.00 (tdt, *J* = 6.2, 4.3, 2.3 Hz, 2H), 1.71 (ddp, *J* = 9.3, 5.6, 3.1 Hz, 2H), 1.56 (ddt, *J* = 8.7, 6.1, 2.8 Hz, 2H).
- **$^{13}$C NMR (101 MHz, $CD_3CN$)** δ 156.6, 155.4, 150.4, 121.5, 115.5, 104.9, 56.2, 27.5, 24.2, 23.6, 23.1.
- **HRMS (ESI):** calcd. for $C_{13}H_{16}O_2^+$ [M]$^+$ 204.1144; found: 204.1146

**(2-(Prop-1-en-2-yloxy)ethyl)benzene (59)**

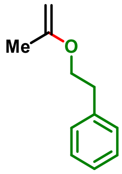


59

Following general procedure **VI**, 2-bromoprop-1-ene (28 µL, 0.30 mmol, 1.5 equiv), 2-phenylethan-1-ol (25 mg, 0.20 mmol, 1.0 equiv), TMG (24 mg, 1.0 equiv) and 4CzIPN (0.8 mg, 0.5 mol%), NiBr$_2$·glyme (3.2 mg, 5.0 mol%), dOMebpy (4.3 mg, 10 mol%) were stirred. After 24h one equivalent of TMG (24 mg, 1.0 equiv) was added to the reaction mixture and stirred for another 24h. After the workup, the organic phases were concentrated under vacuum. The crude product was purified by flash chromatography using silica, EtOAc:Hexane to afford the product **59** (24 mg, 74%).

- **$^1$H NMR (400 MHz, $CD_3CN$)** δ 7.35 – 7.20 (m, 5H), 3.91 – 3.82 (m, 4H), 2.94 (t, *J* = 6.8 Hz, 2H), 1.75 (s, 3H).
- **$^{13}$C NMR (101 MHz, $CD_3CN$)** δ 160.5, 140.0, 129.9, 129.3, 127.2, 82.2, 68.8, 35.9, 21.1.
- **HRMS (ESI):** Not detected in HRMS (ESI, CI).

**(2-Phenethoxyvinyl)benzene (60)**

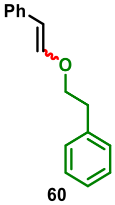

60

Following general procedure **VI**, (2-bromovinyl)benzene (55 mg, 0.30 mmol, 1.5 equiv), 2-phenylethan-1-ol (25 mg, 0.20 mmol, 1.0 equiv), TMG (24 mg, 1.0 equiv) and 4CzIPN (0.8 mg, 0.5 mol%), $NiBr_2$·glyme (3.2 mg, 5.0 mol%), dOMebpy (4.3 mg, 10 mol%) were stirred. After 24h one equivalent of TMG (24 mg, 1.0 equiv) was added to the reaction mixture and stirred for another 24h. After the workup, the organic phases were concentrated under vacuum. The crude product was purified by flash chromatography using silica, EtOAc:Hexane to afford the product **60** (32 mg, 72%, 55:45 *dr*).

- **$^{1}$H NMR (400 MHz, $CD_3CN$)** δ 7.55 – 7.42 (m, 1H), 7.36 – 7.28 (m, 4H), 7.26 – 7.17 (m, 4H), 7.14 – 7.04 (m, 1.5H), 6.29 (d, *J* = 7.0 Hz, 0.5H), 5.86 (d, *J* = 13.0 Hz, 0.5H), 5.20 (d, *J* = 7.0 Hz, 0.5H), 4.14 (t, *J* = 6.6 Hz, 1.2H), 4.06 (t, *J* = 6.8 Hz, 0.8H), 2.99 (q, *J* = 7.0 Hz, 2H).
- **$^{13}$C NMR (101 MHz, $CD_3CN$)** δ 149.08, 147.87, 139.62, 137.64, 137.14, 130.08, 129.97, 129.54, 129.39, 129.35, 129.04, 129.03, 127.34, 126.50, 126.48, 125.88, 106.87, 105.96, 75.03, 71.57, 36.89, 36.32.
- **HRMS (ESI):** calcd. for $C_{16}H_{16}O^+$ $[M]^+$ 224.1195; found: 224.1195

**2-(Cyclohex-1-en-1-yloxy)-2,3-dihydro-1H-indene (61)**

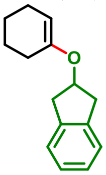

61

Following general procedure **VI**, 1-bromocyclohex-1-ene (35 µL, 0.30 mmol, 1.5 equiv), 2,3-dihydro-1*H*-inden-2-ol (27 mg, 0.20 mmol, 1.0 equiv), TMG (24 mg, 1.0 equiv) and 4CzIPN (0.8 mg, 0.5 mol%), $NiBr_2$·glyme (3.2 mg, 5.0 mol%), dOMebpy (4.3 mg, 10 mol%) were stirred. After 48h one equivalent of TMG (24 mg, 1.0 equiv) was added to the reaction mixture and stirred for another 48h. After the workup, the organic phases were concentrated under vacuum. The crude product was purified by flash chromatography using silica, EtOAc:Hexane to afford the product **61** (35 mg, 81%)

- **$^{1}$H NMR (400 MHz, $CD_3CN$)** δ 7.20 (ddd, *J* = 8.9, 5.2, 3.4 Hz, 2H), 7.16 – 7.11 (m, 2H), 4.85 (tt, *J* = 6.4, 2.5 Hz, 1H), 4.76 – 4.65 (m, 1H), 3.21 (td, *J* = 16.0, 15.2, 6.6 Hz, 2H), 3.03 – 2.86 (m, 2H), 2.07 (dddq, *J* = 10.2, 6.1, 4.1, 2.4 Hz, 2H), 1.92 – 1.87 (m, 2H), 1.67 – 1.58 (m, 2H), 1.53 (qd, *J* = 5.9, 2.5 Hz, 2H).
- **$^{13}$C NMR (101 MHz, $CD_3CN$)** δ 153.54, 142.38, 127.38, 125.57, 125.37, 96.14, 76.53, 40.30, 28.68, 24.30, 23.69, 23.50.
- **HRMS (ESI):** calcd. for $C_{15}H_{18}O^+$ $[M]^+$ 214.1352; found: 214.1354

**2-(Styryloxy)-2,3-dihydro-1H-indene (62)**

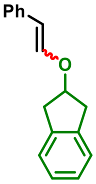


**62**

Following general procedure **VI**, (2-bromovinyl)benzene (55 mg, 0.30 mmol, 1.5 equiv),2,3-dihydro-1*H*-inden-2-ol (27 mg, 0.20 mmol, 1.0 equiv), TMG (24 mg, 1.0 equiv) and 4CzIPN (0.8 mg, 0.5 mol%), $NiBr_2$·glyme (3.2 mg, 5.0 mol%), dOMebpy (4.3 mg, 10 mol%) were stirred. After 24h one equivalent of TMG (24 mg, 1.0 equiv) was added to the reaction mixture and stirred for another 24h. After the workup, the organic phases were concentrated under vacuum. The crude product was purified by flash chromatography using silica, EtOAc:Hexane to afford the product **62** (15 mg, 32%, 60:40 *dr*)

- **$^1$H NMR (400 MHz, $CD_3CN$)** δ 7.52 – 7.38 (m, 1H), 7.25 (dd, *J* = 4.7, 2.9 Hz, 4.5H), 7.20 – 7.09 (m, 3H), 7.09 – 7.03 (m, 1H), 6.40 (d, *J* = 7.1 Hz, 0.5H), 5.86 (d, *J* = 12.9 Hz, 0.5H), 5.28 (d, *J* = 7.1 Hz, 0.5H), 4.94 – 4.76 (m, 1H), 3.29 (dt, *J* = 17.0, 5.3 Hz, 2H), 3.09 (ddd, *J* = 24.7, 16.8, 2.5 Hz, 2H).
- **$^{13}$C NMR (101 MHz, $CD_3CN$)** δ 147.72, 146.39, 141.89, 141.72, 137.65, 137.14, 129.55, 129.02, 128.91, 127.63, 127.58, 126.54, 126.52, 125.87, 125.64, 108.29, 106.92, 84.24, 81.69, 40.54, 40.30.
- **HRMS (ESI):** calcd. for $C_{17}H_{16}O^+$ $[M]^+$ 236.1173; found: 236.1180

**Methyl *N*-(tert-butoxycarbonyl)-*S*-(2-methylprop-1-en-1-yl)-*L*-cysteinate (63)**

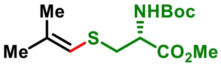


**63**

Following general procedure **I**, 1-bromo-2-methylprop-1-ene (25 µL, 0.24 mmol, 1.2 equiv), methyl (tert-butoxycarbonyl)-L-cysteinate (47 mg, 0.20 mmol, 1.0 equiv), and 4CzIPN (4.0 mg, 2.5 mol%), were stirred for 6 h. After the workup, the organic phases were concentrated under vacuum. The crude product was purified by flash chromatography using silica, EtOAc:Hexane to afford the product **63** as colourless oil (71 mg, 61%).

- $^1$H NMR **(400 MHz, $CDCl_3$)** δ 5.55 (p, *J* = 1.3 Hz, 1H), 5.41 – 5.21 (m, 1H), 4.53 (dt, *J* = 8.8, 4.7 Hz, 1H), 3.71 (s, 3H), 3.04 (d, *J* = 4.8 Hz, 2H), 1.78 – 1.68 (m, 6H), 1.44 (s, 9H).
- **$^{13}$C NMR (101 MHz, $CDCl_3$):** δ 171.2, 155.0, 137.0, 116.8, 80.0, 53.6, 52.4, 36.5, 28.3, 25.3, 19.6.
- **HRMS (ESI):** calcd. for $C_{13}H_{24}NO_4S^+$ $[M+H]^+$ = 290.1421, found = 290.1425.

**Methyl *N*-(tert-butoxycarbonyl)-*S*-styryl-*L*-cysteinate (64)**

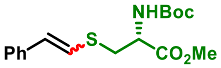


**64**

Following general procedure **I**, (2-bromovinyl)benzene (26 µL, 0.24 mmol, 1.2 equiv), methyl (tert-butoxycarbonyl)-L-cysteinate (47 mg, 0.20 mmol, 1.0 equiv), and 4CzIPN (4.0 mg, 2.5 mol%), were stirred for 6 h. After the workup, the organic phases were concentrated under vacuum. The crude product was

purified by flash chromatography using silica, EtOAc:Hexane to afford the product **64** as yellow oil (113 mg, 84%, 70:30 *dr*).

- **$^{1}$H NMR (400 MHz, $CDCl_3$)** δ 7.48 – 7.43 (m, 1H), 7.38 – 7.32 (m, 1H), 7.29 (d, *J* = 3.9 Hz, 2H), 7.24 – 7.16 (m, 1H), 6.69 – 6.55 (m, 1H), 6.44 (d, *J* = 10.8 Hz, 0.5H), 6.17 (d, *J* = 10.8 Hz, 0.5H), 5.48 (t, *J* = 10.2 Hz, 1H), 4.66 (dd, *J* = 9.2, 4.8 Hz, 1H), 3.80 – 3.68 (m, 3H), 3.30 – 3.21 (m, 1.5H), 3.02 – 2.89 (m, 0.5H), 1.48 – 1.39 (m, 9H).
- **$^{13}$C NMR (101 MHz, $CDCl_3$):** δ 171.0, 170.9, 155.0, 136.6, 136.5, 129.6, 128.7, 128.6, 128.6, 127.3, 126.9, 126.5, 126.4, 125.7, 124.0, 80.2, 54.9, 53.8, 53.6, 52.6, 38.3, 35.7, 28.3, 28.3, 28.1, 27.3.
- **HRMS (ESI):** calcd. for $C_{17}H_{24}NO_4S^+$ $[M+H]^+$ = 338.1421, found = 338.141

**Methyl S-(4-bromostyryl)-N-(tert-butoxycarbonyl)-L-cysteinate (65)**

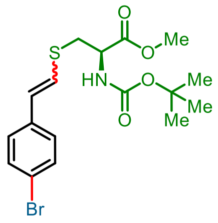


**65**

Following general procedure **I**, 1-bromo-4-(2-bromovinyl)benzene (63 mg, 0.24 mmol, 1.2 equiv), the nucleophile methyl (tert-butoxycarbonyl)-*L*-cysteinate (47 mg, 0.20 mmol, 1.0 equiv), and 4CzIPN (4.0 mg, 2.5 mol%) were stirred for 6h. After the workup, the organic phases were concentrated under vacuum. The crude product was purified by flash chromatography using silica, EtOAc:Hexane to afford the product **65** as yellow oil (54 mg, 64%, 75:25 *dr*).

- **$^{1}$H NMR (400 MHz, $CDCl_3$)** δ 7.48 – 7.32 (m, 2H), 7.17 – 7.06 (m, 2H), 6.63 (d, *J* = 15.6 Hz, 1H), 6.50 (d, *J* = 15.6 Hz, 1H), 5.38 (d, *J* = 8.0 Hz, 1H), 4.71 – 4.50 (m, 1H), 3.83 – 3.74 (m, 1H), 3.71 (s, 2H), 3.34 – 3.12 (m, 2H), 1.44 (d, *J* = 12.8 Hz, 9H).
- **$^{13}$C NMR (101 MHz, $CDCl_3$)** δ 170.9, 154.9, 135.5, 131.7, 127.9, 127.1, 125.2, 120.9, 80.3, 53.5, 52.7, 35.7, 28.3, 27.3.
- **HRMS (ESI):** calcd. for $C_{17}H_{22}BrNO_4SNa^+$ $[M+Na]^+$ = 438.0351, found = 438.0343

**(2-((2-methylprop-1-en-1-yl)thio)propanoyl)glycine (66)**

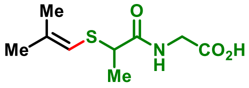


**66**

Following general procedure **I**, 1-bromo-2-methylprop-1-ene (25 µL, 0.24 mmol, 1.2 equiv), (2-mercaptopropanoyl)glycine (33 mg, 0.20 mmol, 1.0 equiv), and 4CzIPN (4.0 mg, 2.5 mol%), were stirred for 6 h. After the workup, the organic phases were concentrated under vacuum. The crude product was purified by flash chromatography using silica, EtOAc:Hexane to afford the product **66** as white solid (55 mg, 63%).

- **$^{1}$H NMR (400 MHz, DMSO)** δ 12.55 (s, 1H), 8.28 (t, *J* = 5.9 Hz, 1H), 5.85 (p, *J* = 1.3 Hz, 1H), 3.75 (d, *J* = 5.9 Hz, 2H), 3.55 (q, *J* = 7.0 Hz, 1H), 1.73 (d, *J* = 1.3 Hz, 3H), 1.64 (d, *J* = 1.1 Hz, 3H), 1.28 (d, *J* = 7.0 Hz, 3H).

- **$^{13}$C NMR (101 MHz, DMSO)** δ 171.73, 171.11, 134.50, 115.51, 42.94, 40.78, 24.95, 19.34, 17.92.
- **HRMS (ESI):** calcd. for $C_9H_{16}NO_3S^+$ $[M+H]^+$ = 218.0845, found = 218.0850.

***N*-acetyl-*S*-(2-methylprop-1-en-1-yl)-*L*-cysteine (67)**

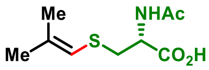


**67**

Following general procedure **I**, 1-bromo-2-methylprop-1-ene (25 µL, 0.24 mmol, 1.2 equiv), acetyl-*L*-cysteine (33 mg, 0.20 mmol, 1.0 equiv), and 4CzIPN (4.0 mg, 2.5 mol%), were stirred for 6 h. After the workup, the organic phases were concentrated under vacuum. The crude product was purified by flash chromatography using silica, EtOAc:Hexane to afford the product **67** as colourless oil (61 mg, 70%).

- $^{1}$H NMR **(400 MHz, $CDCl_3$)** δ 8.75 (s, 1H), 6.58 (d, *J* = 7.3 Hz, 1H), 5.59 (p, *J* = 1.3 Hz, 1H), 4.77 (dt, *J* = 7.3, 4.7 Hz, 1H), 3.23 – 3.06 (m, 2H), 2.02 (s, 3H), 1.71 (dd, *J* = 4.9, 1.3 Hz, 6H).
- $^{13}$C NMR **(101 MHz, $CDCl_3$)** δ 172.28, 170.75, 136.10, 117.19, 52.63, 35.62, 25.08, 22.82, 19.46.
- **HRMS (ESI):** calcd. for $C_9H_{16}NO_3S^+$ $[M+H]^+$ = 218.0845, found = 218.0848.

***N*-acetyl-*S*-styryl-*L*-cysteine (68)**

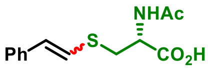


**68**

Following general procedure **I**, (2-bromovinyl)benzene (26 µL, 0.24 mmol, 1.2 equiv), acetyl-*L*-cysteine (33 mg, 0.20 mmol, 1.0 equiv), and 4CzIPN (4.0 mg, 2.5 mol%), were stirred for 6 h. After the workup, the organic phases were concentrated under vacuum. The crude product was purified by flash chromatography using silica, EtOAc:Hexane to afford the product **68** as yellow oil (69 mg, 65%, 60:40 *dr*).

- **$^{1}$H NMR (400 MHz, $CDCl_3$)** δ 9.89 (s, 1H), 7.41 – 7.32 (m, 1H), 7.28 – 7.07 (m, 4H), 6.66 (dd, *J* = 11.2, 7.1 Hz, 1H), 6.62 – 6.44 (m, 1H), 6.32 (d, *J* = 10.8 Hz, 0.5H), 6.11 (d, *J* = 10.8 Hz, 0.5H), 4.81 – 4.74 (m, 1H), 3.35 – 3.13 (m, 2H), 1.93 (d, *J* = 14.4 Hz, 3H).
- **$^{13}$C NMR (101 MHz, $CDCl_3$)** δ 172.2, 171.2, 136.47, 136.45, 128.81, 128.59, 128.56, 128.17, 127.14, 126.79, 126.70, 126.16, 125.58, 124.31, 52.68, 52.55, 38.11, 35.56, 22.75, 22.73.
- **HRMS (ESI):** calcd. For $C_{13}H_{16}NO_3S^+$ $[M+H]^+$ = 266.045, found = 266.0846.

**Methyl *N*$^{α}$-(*tert*-butoxycarbonyl)-*N*$^{τ}$-styryl-*L*-histidinate (69)**

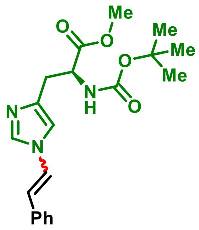


**69**

Following general procedure **VI**, (2-bromovinyl)benzene (55 mg, 0.30 mmol, 1.5 equiv), methyl (*tert*-butoxycarbonyl)-L-histidinate (54 mg, 0.20 mmol, 1.0 equiv), TMG (24 mg, 1.0 equiv) and 4CzIPN (0.8 mg, 0.5 mol%), $NiBr_2$·glyme (3.2 mg, 5.0 mol%), dOMebpy (4.3 mg, 10 mol%) were stirred. After 24h one equivalent of TMG (24 mg, 1.0 equiv) was added to the reaction mixture and stirred for another 24h. After

the workup, the organic phases were concentrated under vacuum. The crude product was purified by flash chromatography using silica, EtOAc:Hexane to afford the product **69** (45 mg, 61%, 60:40 *dr*)

- **$^1$H NMR (400 MHz, $CD_3CN$)** δ 7.72 (d, *J* = 1.4 Hz, 0.5H), 7.54 – 7.21 (m, 5H), 7.11 – 7.04 (m, 1H), 6.85 – 6.74 (m, 1H), 6.69 (s, 0.5H), 6.53 – 6.35 (m, 1H), 4.35 (dt, *J* = 8.2, 5.4 Hz, 0.5H), 4.05 (ddd, *J* = 8.9, 5.8, 1.6 Hz, 0.5H), 3.64 (s, 1H), 3.60 (s, 2H), 3.01 – 2.93 (m, 1H), 2.90 – 2.82 (m, 1H), 1.39 (d, *J* = 2.1 Hz, 9H).
- **$^{13}$C NMR (101 MHz, $CD_3CN$)** δ 173.3, 156.4, 139.8, 138.8, 138.0, 137.7, 135.1, 129.9, 129.7, 129.5, 129.1, 128.6, 127.2, 127.1, 126.1, 124.3, 124.2, 123.7, 117.2, 114.9, 80.0, 54.6, 52.6, 30.3, 28.5. (Seven carbon)
- **HRMS (ESI):** calcd. for $C_{20}H_{26}N_3O_4^+$ $[M+H]^+$ 372.1923; found: 372.1919

**Methyl *N*$^α$-(tert-butoxycarbonyl)-1-(prop-1-en-2-yl)-*L*-tryptophanate (70)**

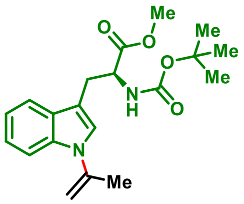


**70**

Following general procedure **VI**, 2-bromoprop-1-ene (28 µL, 0.30 mmol, 1.5 equiv), methyl (*tert*-butoxycarbonyl)-*L*-tryptophanate (58 mg, 0.20 mmol, 1.0 equiv), TMG (24 mg, 1.0 equiv) and 4CzIPN (0.8 mg, 0.5 mol%), $NiBr_2$·glyme (3.2 mg, 5.0 mol%), dOMebpy (4.3 mg, 10 mol%) were stirred. After 24h one equivalent of TMG (24 mg, 1.0 equiv) was added to the reaction mixture and stirred for another 24h. After the workup, the organic phases were concentrated under vacuum. The crude product was purified by flash chromatography using silica, EtOAc:Hexane to afford the product **70** (29 mg, 41%)

- **$^1$H NMR (400 MHz, $CD_3CN$)** δ 7.66 – 7.59 (m, 1H), 7.55 (dd, *J* = 8.1, 1.1 Hz, 1H), 7.24 – 7.18 (m, 2H), 7.12 (ddd, *J* = 8.0, 7.0, 1.0 Hz, 1H), 5.53 (d, *J* = 8.3 Hz, 1H), 5.11 (s, 1H), 5.07 (q, *J* = 1.3 Hz, 1H), 4.45 (q, *J* = 7.4 Hz, 1H), 3.65 (s, 3H), 3.23 (dd, *J* = 14.7, 5.5 Hz, 1H), 3.10 (dd, *J* = 14.7, 7.7 Hz, 1H), 2.25 (s, 3H), 1.35 (s, 9H).
- **$^{13}$C NMR (101 MHz, $CD_3CN$)** δ 173.6, 156.3, 141.8, 136.6, 130.0, 126.3, 123.4, 121.0, 119.8, 113.0, 112.2, 105.3, 80.0, 55.2, 52.7, 28.5, 28.2, 22.2.
- **HRMS (ESI):** calcd. for $C_{20}H_{26}N_2O_4Na^+$ $[M+Na]^+$ 381.1790; found: 381.1789

**Methyl *N*$^α$-(*tert*-butoxycarbonyl)-1-(2-methylprop-1-en-1-yl)-*L*-tryptophanate (71)**

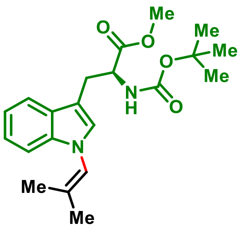


**71**

Following general procedure **VI**, 1-bromo-2-methylprop-1-ene (31 µL, 0.30 mmol, 1.5 equiv), methyl (*tert*-butoxycarbonyl)-*L*-tryptophanate (58 mg, 0.20 mmol, 1.0 equiv), TMG (24 mg, 1.0 equiv) and 4CzIPN (0.8 mg, 0.5 mol%), $NiBr_2$·glyme (3.2 mg, 5.0 mol%), dOMebpy (4.3 mg, 10 mol%) were stirred. After 48h one equivalent of TMG (24 mg, 1.0 equiv) was added to the reaction mixture and stirred for another 48h. After

the workup, the organic phases were concentrated under vacuum. The crude product was purified by flash chromatography using silica, EtOAc:Hexane to afford the product **71** (31 mg, 42%)

- **$^{1}$H NMR (400 MHz, $CD_3CN$)** δ 7.54 (d, *J* = 7.9 Hz, 1H), 7.29 – 7.22 (m, 1H), 7.18 (ddd, *J* = 8.2, 6.8, 1.2 Hz, 1H), 7.10 (ddd, *J* = 8.0, 6.8, 1.2 Hz, 1H), 7.03 (s, 1H), 6.61 (p, *J* = 1.5 Hz, 1H), 5.48 (d, *J* = 8.3 Hz, 1H), 4.43 (q, *J* = 7.2 Hz, 1H), 3.64 (s, 3H), 3.24 (dd, *J* = 14.7, 5.5 Hz, 1H), 3.11 (dd, *J* = 14.7, 7.5 Hz, 1H), 1.90 (d, *J* = 1.5 Hz, 3H), 1.67 (d, *J* = 1.5 Hz, 3H), 1.34 (s, 9H).
- **$^{13}$C NMR (101 MHz, $CD_3CN$)** δ 137.6, 132.8, 128.1, 123.0, 120.5, 120.4, 119.6, 111.3, 55.4, 52.7, 28.5, 28.2, 22.5, 18.4.
- **HRMS (ESI):** calcd. for $C_{21}H_{28}N_2O_4Na^+$ $[M+Na]^+$ 395.1947; found: 395.1942

**Methyl *N*$^{α}$-(*tert*-butoxycarbonyl)-1-styryl-*L*-tryptophanate (72)**

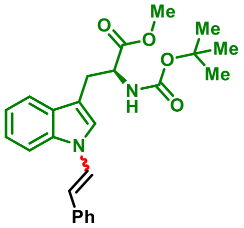


**72**

Following general procedure **VI**, (2-bromovinyl)benzene (55 mg, 0.30 mmol, 1.5 equiv), methyl (*tert*-butoxycarbonyl)-*L*-tryptophanate **xx** (58 mg, 0.20 mmol, 1.0 equiv), TMG (24 mg, 1.0 equiv) and 4CzIPN (0.8 mg, 0.5 mol%), $NiBr_2$·glyme (3.2 mg, 5.0 mol%), dOMebpy (4.3 mg, 10 mol%) were stirred. After 24h one equivalent of TMG (24 mg, 1.0 equiv) was added to the reaction mixture and stirred for another 24h. After the workup, the organic phases were concentrated under vacuum. The crude product was purified by flash chromatography using silica, EtOAc:Hexane to afford the product **72** (35 mg, 42%, 70:30 *dr*).

- **$^{1}$H NMR (400 MHz, $CD_3CN$)** δ 7.82 (d, *J* = 14.5 Hz, 0.5H), 7.68 (d, *J* = 8.3 Hz, 0.5H), 7.61 – 7.50 (m, 2H), 7.39 – 7.10 (m, 7H), 7.04 – 6.86 (m, 1H), 6.74 (d, *J* = 14.5 Hz, 0.4H), 6.34 (d, *J* = 9.2 Hz, 0.6H), 5.59 (d, *J* = 8.3 Hz, 0.4H), 5.40 (d, *J* = 8.2 Hz, 0.6H), 4.50 (q, *J* = 7.4 Hz, 0.4H), 4.38 (q, *J* = 7.2 Hz, 0.6H), 3.67 (s, 1H), 3.57 (s, 2H), 3.28 (dd, *J* = 14.7, 5.4 Hz, 0.5H), 3.14 (dt, *J* = 15.0, 5.8 Hz, 1H), 3.04 (dd, *J* = 14.7, 7.3 Hz, 0.5H), 1.35 (d, *J* = 8.1 Hz, 9H).
- **$^{13}$C NMR (101 MHz, $CD_3CN$)** δ 173.5, 173.4, 156.3, 137.4, 136.9, 136.0, 129.8, 129.6, 129.5, 129.1, 128.6, 127.7, 126.7, 126.6, 124.2, 123.9, 123.6, 123.5, 121.7, 121.4, 120.9, 120.1, 119.8, 115.1, 114.1, 113.3, 111.4, 111.1, 80.0, 55.1, 52.8, 52.7, 28.5, 28.0.
- **HRMS (ESI):** calcd. for $C_{25}H_{28}N_2O_4Na^+$ $[M+Na]^+$ 443.1947; found: 443.1944

**Methyl (*S*)-2-((*tert*-butoxycarbonyl)amino)-3-(4-(prop-1-en-2-yloxy)phenyl)propanoate (73)**

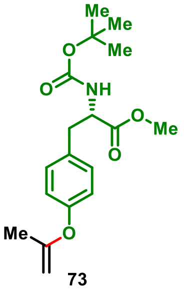


**73**

Following general procedure **V**, 2-bromoprop-1-en (28 µL, 0.30 mmol, 1.5 equiv), methyl (*tert*-butoxycarbonyl)-*L*-tyrosinate (60 mg, 0.20 mmol, 1.0 equiv), $^{t}BuNH_2$ (44 µL, 0.4 mmol, 2.0 equiv), and

4CzIPN (0.8 mg, 0.5 mol%), $NiBr_2$·glyme (3.2 mg, 5.0 mol%), dOMebpy (4.3 mg, 10 mol%) were stirred for 48 h. After the workup, the organic phases were concentrated under vacuum. The crude product was purified by flash chromatography using silica, EtOAc:Hexane to afford the product **73** (37 mg, 55%).

- **$^1$H NMR (400 MHz, $CD_3CN$)** δ 7.26 – 7.10 (m, 2H), 6.98 – 6.83 (m, 2H), 5.71 – 5.38 (m, 1H), 4.48 – 4.26 (m, 1H), 4.18 (t, *J* = 1.2 Hz, 1H), 3.86 (d, *J* = 1.4 Hz, 1H), 3.64 (s, 3H), 3.06 (dd, *J* = 13.9, 5.4 Hz, 1H), 2.87 (dd, *J* = 13.9, 8.7 Hz, 1H), 1.93 (d, *J* = 0.9 Hz, 3H), 1.33 (s, 9H).
- **$^{13}$C NMR (101 MHz, $CD_3CN$)** δ 173.4, 160.8, 156.3, 155.2, 133.8, 131.5, 121.4, 90.3, 80.0, 55.9, 52.7, 37.7, 28.5, 20.1.
- **HRMS (ESI):** calcd. for $C_{18}H_{25}NO_5Na^+$ $[M+Na]^+$ 358.1630; found: 358.1631

**Methyl (*S*)-2-((*tert*-butoxycarbonyl)amino)-3-(4-(cyclohex-1-en-1-yloxy)phenyl)propanoate (74)**

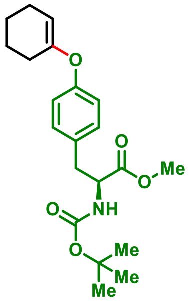


**74**

Following general procedure **V**, 1-bromocyclohex-1-ene (35 µL, 0.30 mmol, 1.5 equiv), methyl (*tert*-butoxycarbonyl)-*L*-tyrosinate (60 mg, 0.20 mmol, 1.0 equiv), $^t$BuNH$_2$ (44 µL, 0.4 mmol, 2.0 equiv), and 4CzIPN (0.8 mg, 0.5 mol%), $NiBr_2$·glyme (3.2 mg, 5.0 mol%), dOMebpy (4.3 mg, 10 mol%) were stirred for 96 h. After the workup, the organic phases were concentrated under vacuum. The crude product was purified by flash chromatography using silica, EtOAc:Hexane to afford the product **74** (30 mg, 40%).

- **$^1$H NMR (400 MHz, $CD_3CN$)** δ 7.22 – 7.08 (m, 2H), 6.93 – 6.80 (m, 2H), 5.49 (d, *J* = 8.5 Hz, 1H), 4.98 (tt, *J* = 4.0, 1.5 Hz, 1H), 4.46 – 4.14 (m, 1H), 3.65 (s, 3H), 3.04 (dd, *J* = 13.9, 5.4 Hz, 1H), 2.84 (dd, *J* = 13.9, 8.7 Hz, 1H), 2.14 – 2.08 (m, 2H), 2.05 (tdt, *J* = 6.2, 4.5, 2.3 Hz, 2H), 1.76 – 1.69 (m, 2H), 1.61 – 1.54 (m, 2H), 1.34 (s, 9H).
- **$^{13}$C NMR (101 MHz, $CD_3CN$)** δ 173.5, 156.3, 154.0, 132.2, 131.4, 119.3, 108.2, 80.0, 56.1, 52.7, 37.6, 28.5, 27.3, 24.3, 23.6, 22.9.
- **HRMS (ESI):** calcd. for $C_{21}H_{29}NO_5Na^+$ $[M+Na]^+$ 398.1943; found: 398.1944

**Methyl (*S*)-2-((*tert*-butoxycarbonyl)amino)-3-(4-(styryloxy)phenyl)propanoate (75)**

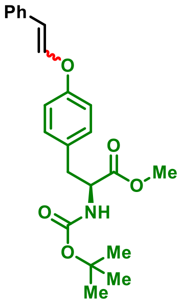


**75**

Following general procedure **V**, (2-bromovinyl)benzene (55 mg, 0.30 mmol, 1.5 equiv), methyl (*tert*-butoxycarbonyl)-*L*-tyrosinate (60 mg, 0.20 mmol, 1.0 equiv),, $^t$BuNH$_2$ (44 µL, 0.4 mmol, 2.0 equiv), and

4CzIPN (0.8 mg, 0.5 mol%), $NiBr_2$·glyme (3.2 mg, 5.0 mol%), dOMebpy (4.3 mg, 10 mol%) were stirred for 48 h. After the workup, the organic phases were concentrated under vacuum. The crude product was purified by flash chromatography using silica, EtOAc:Hexane to afford the product **75** (31 mg, 39%, 70:30 *dr*).

- **$^{1}$H NMR (400 MHz, $CD_3CN$)** δ 7.72 – 7.63 (m, 1H), 7.42 – 7.28 (m, 3H), 7.25 – 7.17 (m, 3H), 7.12 – 7.01 (m, 2.3H), 6.70 (d, *J* = 6.9 Hz, 0.7H), 6.32 (d, *J* = 12.5 Hz, 0.3H), 5.66 (d, *J* = 6.9 Hz, 0.7H), 5.56 – 5.42 (m, 1H), 4.44 – 4.20 (m, 1H), 3.66 (s, 3H), 3.08 (dd, *J* = 13.9, 5.2 Hz, 1H), 2.95 – 2.79 (m, 1H), 1.39 – 1.27 (m, 9H).
- **$^{13}$C NMR (101 MHz, $CD_3CN$)** δ 173.4, 156.9, 156.3, 144.6, 142.8, 136.0, 133.2, 132.9, 131.7, 131.6, 129.6, 129.5, 129.3, 127.6, 127.57, 126.64, 117.5, 117.4, 116.7, 114.0, 110.9, 80.0, 55.9, 52.7, 37.5, 28.6.
- **HRMS (ESI):** calcd. for $C_{23}H_{28}NO_5^+$ $[M+H]^+$ 420.1787; found: 420.1783

**(3a*R*,5*R*,5a*S*,8a*S*,8b*R*)-2,2,7,7-Tetramethyl-5-((prop-1-en-2-yloxy)methyl)tetrahydro-5*H*-bis([1,3]dioxolo)[4,5-b:4',5'-*d*]pyran (76)**

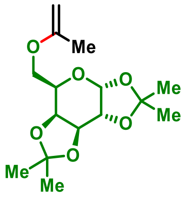


**76**

Following general procedure **VI**, 2-bromoprop-1-ene (28 µL, 0.30 mmol, 1.5 equiv), ((3a*R*,5*R*,5a*S*,8a*S*,8b*R*)-2,2,7,7-tetramethyltetrahydro-5*H*-bis([1,3]dioxolo)[4,5-b:4',5'-*d*]pyran-5-yl)methanol (53 mg, 0.20 mmol, 1.0 equiv), TMG (24 mg, 1.0 equiv) and 4CzIPN (0.8 mg, 0.5 mol%), $NiBr_2$·glyme (3.2 mg, 5.0 mol%), dOMebpy (4.3 mg, 10 mol%) were stirred. After 24h one equivalent of TMG (24 mg, 1.0 equiv) was added to the reaction mixture and stirred for another 24h. After the workup, the organic phases were concentrated under vacuum. The crude product was purified by flash chromatography using silica, EtOAc:Hexane to afford the product **76** (31 mg, 51%).

- **$^{1}$H NMR (400 MHz, $CD_3CN$)** δ 5.45 (dd, *J* = 10.0, 5.2 Hz, 1H), 4.60 (ddd, *J* = 14.9, 7.9, 2.5 Hz, 1H), 4.39 – 4.17 (m, 2H), 4.03 (ddd, *J* = 7.1, 5.0, 2.0 Hz, 1H), 3.93 – 3.86 (m, 1H), 3.84 – 3.75 (m, 1H), 3.70 (dd, *J* = 10.3, 7.1 Hz, 1H), 3.59 – 3.39 (m, 1H), 2.12 (s, 1H), 1.77 (d, *J* = 0.8 Hz, 2H), 1.47 (d, *J* = 2.1 Hz, 3H), 1.37 (d, *J* = 5.9 Hz, 3H), 1.30 (dd, *J* = 3.1, 1.7 Hz, 6H).
- **$^{13}$C NMR (101 MHz, $CD_3CN$)** δ 160.4, 109.9, 109.4, 97.2, 82.5, 72.0, 71.6, 71.4, 67.3, 67.0, 26.4, 26.3, 25.2, 24.7, 21.0.
- **HRMS (ESI):** calcd. for $C_{15}H_{25}O_6^+$ $[M+H]^+$ 301.1651; found: 301.1644

**(3a*R*,5*R*,5a*S*,8a*S*,8b*R*)-5-((cyclohex-1-en-1-yloxy)methyl)-2,2,7,7-tetramethyltetrahydro-5*H*-bis([1,3]dioxolo)[4,5-b:4',5'-*d*]pyran (77)**

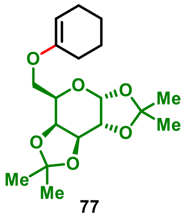


**77**

Following general procedure **VI**, 1-bromocyclohex-1-ene (35 μL, 0.30 mmol, 1.5 equiv), ((3a*R*,5*R*,5a*S*,8a*S*,8b*R*)-2,2,7,7-tetramethyltetrahydro-5*H*-bis([1,3]dioxolo)[4,5-b:4',5'-*d*]pyran-5-yl)methanol (53 mg, 0.20 mmol, 1.0 equiv), TMG (24 mg, 1.0 equiv) and 4CzIPN (0.8 mg, 0.5 mol%), $NiBr_2$·glyme (3.2 mg, 5.0 mol%), dOMebpy (4.3 mg, 10 mol%) were stirred. After 48h one equivalent of TMG (24 mg, 1.0 equiv) was added to the reaction mixture and stirred for another 48h. After the workup, the organic phases were concentrated under vacuum. The crude product was purified by flash chromatography using silica, EtOAc:Hexane to afford the product **77** (27 mg, 39%).

- **$^1$H NMR (400 MHz, $CD_3CN$)** δ 5.45 (d, *J* = 5.0 Hz, 1H), 4.65 (t, *J* = 3.8 Hz, 1H), 4.60 (dd, *J* = 7.9, 2.5 Hz, 1H), 4.33 (dd, *J* = 5.0, 2.5 Hz, 1H), 4.23 (dd, *J* = 7.9, 1.9 Hz, 1H), 4.00 (ddd, *J* = 7.1, 5.2, 2.0 Hz, 1H), 3.78 (dd, *J* = 10.3, 5.2 Hz, 1H), 3.66 (dd, *J* = 10.2, 7.0 Hz, 1H), 2.01 (dddt, *J* = 8.1, 6.4, 4.8, 2.1 Hz, 4H), 1.70 – 1.60 (m, 2H), 1.55 – 1.48 (m, 2H), 1.46 (s, 3H), 1.37 (s, 3H), 1.30 (d, *J* = 2.2 Hz, 6H).
- **$^{13}$C NMR (101 MHz, $CD_3CN$)** δ 155.0, 109.9, 109.3, 97.3, 95.3, 72.0, 71.6, 71.4, 67.0, 66.1, 28.5, 26.4, 26.3, 25.2, 24.7, 24.2, 23.7, 23.4.
- **HRMS (ESI):** calcd. for $C_{18}H_{29}O_6^+$ $[M+H]^+$ 341.1964; found: 341.1957

**(3a*R*,5*R*,5a*S*,8a*S*,8b*R*)-2,2,7,7-tetramethyl-5-((styryloxy)methyl)tetrahydro-5*H*-bis([1,3]dioxolo)[4,5-b:4',5'-*d*]pyran (78)**

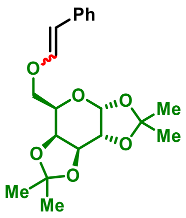


**78**

Following general procedure **VI**, (2-bromovinyl)benzene (55 mg, 0.30 mmol, 1.5 equiv), ((3a*R*,5*R*,5a*S*,8a*S*,8b*R*)-2,2,7,7-tetramethyltetrahydro-5*H*-bis([1,3]dioxolo)[4,5-b:4',5'-*d*]pyran-5-yl)methanol (53 mg, 0.20 mmol, 1.0 equiv), TMG (24 mg, 1.0 equiv) and 4CzIPN (0.8 mg, 0.5 mol%), $NiBr_2$·glyme (3.2 mg, 5.0 mol%), dOMebpy (4.3 mg, 10 mol%) were stirred. After 24h one equivalent of TMG (24 mg, 1.0 equiv) was added to the reaction mixture and stirred for another 24h. After the workup, the organic phases were concentrated under vacuum. The crude product was purified by flash chromatography using silica, EtOAc:Hexane to afford the product **78** (32 mg, 44%, 60:40 *dr*).

- **$^1$H NMR (400 MHz, $CD_3CN$)** δ 8.08 (s, 0.5H), 7.63 – 7.54 (m, 0.5H), 7.42 – 7.30 (m, 1H), 7.28 – 7.21 (m, 2.5H), 7.16 – 7.06 (m, 1H), 6.31 (d, *J* = 7.0 Hz, 0.5H), 5.88 (d, *J* = 13.0 Hz, 0.5H), 5.47 (ddd, *J* = 8.1, 4.9, 2.9 Hz, 1H), 5.24 (d, *J* = 7.0 Hz, 0.5H), 4.63 (dt, *J* = 7.9, 2.8 Hz, 1H), 4.35 (dt, *J* = 4.8, 2.4 Hz, 1H), 4.26 (dddd, *J* = 16.4, 8.5, 6.1, 4.2 Hz, 1H), 4.11 – 3.71 (m, 3H), 1.47 (d, *J* = 8.8 Hz, 3H), 1.42 – 1.37 (m, 3H), 1.31 (d, *J* = 6.3 Hz, 6H).
- **$^{13}$C NMR (101 MHz, $CD_3CN$)** δ 162.3, 149.0, 148.1, 137.5, 137.1, 129.6, 129.5, 129.14, 129.1, 126.9, 126.9, 126.61, 126.60, 125.9, 110.1, 110.1, 109.5, 109.4, 107.2, 106.2, 97.2, 97.1, 73.3, 71.9, 71.6, 71.4, 71.3, 70.2, 68.1, 67.5, 26.4, 26.3, 25.3, 25.2, 24.72, 24.7.
- **HRMS (ESI):** calcd. for $C_{20}H_{26}O_6Na^+$ $[M+Na]^+$ 385.1627; found: 385.1621

**(2-(Prop-1-en-2-yloxy)ethyl)(styryl)sulfane (79)**

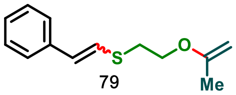


Following general procedure **X**, (2-bromovinyl)benzene (26 µL, 0.24 mmol, 1.2 equiv), 2-mercaptoethan-1-ol (15 µL, 0.20 mmol, 1.0 equiv), and 4CzIPN (4.0 mg, 2.5 mol%) were stirred for 12h. In the next step, 2-bromoprop-1-ene (28 µL, 0.30 mmol, 1.5 equiv), 4CzIPN (0.8 mg, 0.5 mol%) $NiBr_2$·glyme (3.2 mg, 5.0 mol%), dOMebpy (4.3 mg, 10 mol%) and TMG (48 mg, 0.40 mmol, 2.0 equiv) were added and stirred. After 24 h, TMG (24 mg, 0.20 mmol, 1.0 equiv) was added and stirred for another 24h. After the workup, the organic phases were concentrated under vacuum. The crude product was purified by flash chromatography using silica, EtOAc:Hexane to afford the product **79** (26 mg, 58%, 60:40 *dr*).

- **$^1$H NMR (400 MHz, $CD_3CN$)** δ 7.44 (ddt, *J* = 11.9, 6.3, 1.3 Hz, 1H), 7.40 – 7.27 (m, 3H), 7.25 – 7.18 (m, 1H), 6.95 – 6.83 (m, 0.5H), 6.56 – 6.34 (m, 1.5H), 3.93 – 3.85 (m, 3H), 3.72 – 3.61 (m, 1H), 3.08 (q, *J* = 6.2 Hz, 1.5H), 2.95 – 2.80 (m, 0.5H), 1.78 (s, 2H), 1.35 – 1.28 (m, 1H).
- **$^{13}$C NMR (101 MHz, $CD_3CN$)** δ 160.3, 138.1, 138.0, 129.7, 129.4, 129.3, 128.6, 127.9, 127.69, 127.65, 126.4, 126.3, 126.1, 82.7, 67.8, 67.4, 35.0, 32.2, 20.9.
- **HRMS (ESI):** Not detected in HRMS (ESI, CI).

**(2-Methylprop-1-en-1-yl)(2-(styryloxy)ethyl)sulfane (80)**

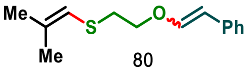


Following general procedure **X**, 1-bromo-2-methylprop-1-ene (25 µL, 0.24 mmol, 1.2 equiv), 2-mercaptoethan-1-ol (15 µL, 0.20 mmol, 1.0 equiv), and 4CzIPN (4.0 mg, 2.5 mol%) were stirred for 12h. In the next step, (2-bromovinyl)benzene (55 mg, 0.30 mmol, 1.5 equiv), 4CzIPN (0.8 mg, 0.5 mol%) $NiBr_2$·glyme (3.2 mg, 5.0 mol%), dOMebpy (4.3 mg, 10 mol%) and TMG (48 mg, 0.40 mmol, 2.0 equiv) were added and stirred. After 24 h, TMG (24 mg, 0.20 mmol, 1.0 equiv) was added and stirred for another 24h. After the workup, the organic phases were concentrated under vacuum. The crude product was purified by flash chromatography using silica, EtOAc:Hexane to afford the product **80** (24 mg, 51%, 60:40 *dr*)

- **$^1$H NMR (400 MHz, $CD_3CN$)** δ 7.62 – 7.52 (m, 1H), 7.41 – 7.18 (m, 4H), 7.15 – 7.04 (m, 1H), 6.29 (d, *J* = 7.1 Hz, 0.3H), 5.87 (d, *J* = 12.9 Hz, 0.4H), 5.76 – 5.64 (m, 1H), 5.24 (d, *J* = 7.1 Hz, 0.3H), 4.23 (td, *J* = 6.8, 0.8 Hz, 0.4H), 4.06 (t, *J* = 6.5 Hz, 0.6H), 3.97 (t, *J* = 6.7 Hz, 1H), 2.96 – 2.80 (m, 2H), 1.78 – 1.74 (m, 2H), 1.70 (d, *J* = 1.4 Hz, 4H).
- **$^{13}$C NMR (101 MHz, $CD_3CN$)** δ 162.2, 148.9, 147.8, 137.4, 129.6, 129.1, 126.6, 126.5, 125.9, 107.2, 106.2, 73.7, 70.5, 34.1, 33.5, 32.7, 25.2, 25.1, 19.6.
- **HRMS (ESI):** calcd. for $C_{14}H_{18}OS^+$ $[M]^+$ 234.1072; found: 234.1078

**Methyl N-(tert-butoxycarbonyl)-S-(4-(((R)-2-((tert-butoxycarbonyl)amino)-3-methoxy-3-oxopropyl)thio)styryl)-L-cysteinate (81)**

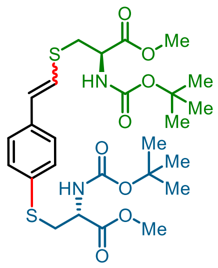


81

Following general procedure **VII**, 1-bromo-4-(2-bromovinyl)benzene (63 mg, 0.24 mmol, 1.2 equiv), the nucleophile methyl (tert-butoxycarbonyl)-*L*-cysteinate (47 mg, 0.20 mmol, 1.0 equiv), and 4CzIPN (4.0 mg, 2.5 mol%) were stirred for 12h. In the next step methyl (tert-butoxycarbonyl)-*L*-cysteinate (70 mg, 0.30 mmol, 1.5 equiv), 4CzIPN (0.8 mg, 0.5 mol%) $NiBr_2$·glyme (3.2 mg, 5.0 mol%), dOMebpy (4.3 mg, 10 mol%) were added and stirred for another 12h. After the workup, the organic phases were concentrated under vacuum. The crude product was purified by flash chromatography using silica, EtOAc:Hexane to afford the product **81** (59 mg, 52%, 60:40 *dr*)

- **$^1$H NMR (400 MHz, $CDCl_3$)** δ 7.36 (m, 1.6 H), 7.32 – 7.29 (m, 1.2H), 7.21 – 7.16 (m, 1.2H), 6.65 – 6.48 (m, 1.2H), 6.36 (d, *J* = 10.8 Hz, 0.4H), 6.15 (d, *J* = 10.8 Hz, 0.4H), 5.41 – 5.30 (m, 1.5H), 4.67 – 4.52 (m, 2H), 3.76 (s, 2H), 3.75 (s, 1H), 3.70 (s, 2H), 3.55 – 3.53 (m, 1H), 3.35 (dd, *J* = 9.9, 4.9 Hz, 1.5H), 3.27 – 3.23 (m, 1.5H), 3.16 (d, *J* = 5.3 Hz, 1H), 1.44 (d, *J* = 1.5 Hz, 9H), 1.42 (s, 4H), 1.41 (s, 5H).
- **$^{13}$C NMR (101 MHz, $CDCl_3$)** δ 171.1, 170.9, 170.8, 170.7, 154.9, 135.4, 131.2, 130.6, 129.2, 128.3, 126.2, 80.3, 80.1, 52.7, 52.6, 52.4, 52.3, 41.3, 37.2, 35.7, 28.34, 28.3, 28.2.
- **HRMS (ESI):** calcd. for $C_{26}H_{38}N_2O_8S_2Na^+$ $[M+Na]^+$ 593.1966; found: 593.1967

**Ethyl 3-((4-((3-ethoxy-3-oxopropyl)thio)styryl)thio)propanoate (82)**

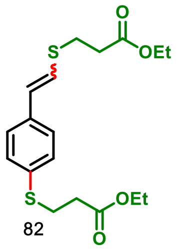


82

Following general procedure **VII**, 1-bromo-4-(2-bromovinyl)benzene (63 mg, 0.24 mmol, 1.2 equiv), ethyl 3-mercaptopropanoate (26 µL, 0.20 mmol, 1.0 equiv), and 4CzIPN (4.0 mg, 2.5 mol%) were stirred for 12h. In the next step 3-mercaptopropanoate (39 µL, 0.30 mmol, 1.5 equiv), 4CzIPN (0.8 mg, 0.5 mol%) $NiBr_2$·glyme (3.2 mg, 5.0 mol%), dOMebpy (4.3 mg, 10 mol%) were added and stirred for another 12h. After the workup, the organic phases were concentrated under vacuum. The crude product was purified by flash chromatography using silica, EtOAc:Hexane to afford the product **82** (45 mg, 61%, 50:50 *dr*)

- **$^1$H NMR (400 MHz, $CD_3CN$)** δ 7.42 – 7.22 (m, 4H), 6.85 (d, *J* = 15.7 Hz, 0.5H), 6.52 – 6.29 (m, 1.5H), 4.08 (dqd, *J* = 10.1, 7.1, 0.9 Hz, 4H), 3.14 (q, *J* = 7.3 Hz, 2H), 3.04 (td, *J* = 7.1, 1.5 Hz, 2H), 2.66 (td, *J* = 7.1, 1.3 Hz, 2H), 2.57 (td, *J* = 7.0, 4.6 Hz, 2H), 1.25 – 1.16 (m, 6H).
- **$^{13}$C NMR (101 MHz, $CD_3CN$)** δ 172.5, 172.4, 172.3, 136.3, 136.1, 135.0, 134.9, 130.6, 130.0, 129.9, 128.4, 127.1, 127.1, 126.1, 125.6, 61.4, 61.3, 35.9, 35.3, 34.9, 31.4, 29.4, 29.2, 28.2, 14.5.
- **HRMS (ESI):** calcd. for $C_{18}H_{24}O_4S_2Na^+$ $[M+Na]^+$ 391.1014; found: 391.1009

**Ethyl 3-((4-(phenylthio)styryl)thio)propanoate (83)**

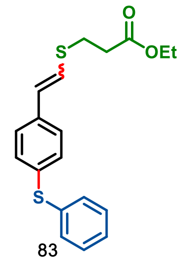


83

Following general procedure **VII**, 1-bromo-4-(2-bromovinyl)benzene (63 mg, 0.24 mmol, 1.2 equiv), ethyl 3-mercaptopropanoate (26 μL, 0.20 mmol, 1.0 equiv), and 4CzIPN (4.0 mg, 2.5 mol%) were stirred for 12h. In the next step, benzenethiol (31 μL, 0.30 mmol, 1.5 equiv), 4CzIPN (0.8 mg, 0.5 mol%) $NiBr_2$·glyme (3.2 mg, 5.0 mol%), dOMebpy (4.3 mg, 10 mol%) were added and stirred for another 12h. After the workup, the organic phases were concentrated under vacuum. The crude product was purified by flash chromatography using silica, EtOAc:Hexane to afford the product **83** (28 mg, 40%, 60:40 *dr*)

- **$^1$H NMR (400 MHz, $CD_3CN$)** δ 7.42 – 7.20 (m, 9H), 6.86 (d, *J* = 15.7 Hz, 0.5H), 6.48 – 6.32 (m, 1.5H), 4.09 (qd, *J* = 7.1, 2.4 Hz, 2H), 3.03 (td, *J* = 7.1, 5.8 Hz, 2H), 2.65 (td, *J* = 7.1, 4.1 Hz, 2H), 1.19 (td, *J* = 7.1, 4.3 Hz, 3H).
- **$^{13}$C NMR (101 MHz, $CD_3CN$)** δ 172.4, 172.2, 137.3, 137.0, 136.7, 136.3, 134.6, 134.4, 132.4, 131.7, 131.6, 131.5, 130.4, 130.3, 130.2, 129.1, 128.3, 128.1, 127.3, 126.9, 126.7, 125.4, 61.4, 35.9, 35.3, 31.4, 28.1, 14.5.
- **HRMS (ESI):** calcd. for $C_{19}H_{20}O_2S_2^+$ $[M]^+$ 344.0899; found: 344.0898

**Ethyl 3-((4-(2-(phenylthio)vinyl)phenyl)thio)propanoate (84)**

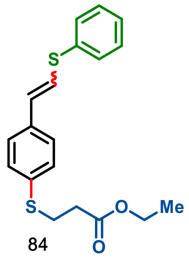


84

Following general procedure **VII**, 1-bromo-4-(2-bromovinyl)benzene (63 mg, 0.24 mmol, 1.2 equiv), ethyl benzenethiol (21 μL, 0.20 mmol, 1.0 equiv), and 4CzIPN (4.0 mg, 2.5 mol%) were stirred for 12h. In the next step, 3-mercaptopropanoate (39 μL, 0.30 mmol, 1.5 equiv), 4CzIPN (0.8 mg, 0.5 mol%) $NiBr_2$·glyme (3.2 mg, 5.0 mol%), dOMebpy (4.3 mg, 10 mol%) were added and stirred for another 12h. After the workup, the organic phases were concentrated under vacuum. The crude product was purified by flash chromatography using silica, EtOAc:Hexane to afford the product **84** (29 mg, 42%, 60:40 *dr*).

- **$^1$H NMR (400 MHz, $CD_3CN$)** δ 7.49 – 7.20 (m, 9H), 7.04 – 6.66 (m, 1H), 6.59 – 6.33 (m, 1H), 4.17 – 4.01 (m, 2H), 3.16 (dt, *J* = 11.2, 7.1 Hz, 1H), 3.05 – 2.95 (m, 1H), 2.70 – 2.50 (m, 2H), 1.23 – 1.14 (m, 3H).
- **$^{13}$C NMR (101 MHz, $CD_3CN$)** δ 172.4, 137.4, 137.1, 134.7, 132.5, 132.3, 131.9, 131.7, 131.6, 131.2, 130.4, 130.3, 130.2, 129.5, 129.1, 128.4, 128.2, 127.3, 126.9, 126.7, 125.5, 125.1, 61.4, 35.9, 35.3, 34.2, 31.4, 28.2, 14.5.
- **HRMS (ESI):** calcd. for $C_{19}H_{20}O_2S_2^+$ $[M]^+$ 344.0899; found: 344.0897

**Ethyl 3-((4-(4,4-diphenylbut-1-en-1-yl)phenyl)thio)propanoate (85)**

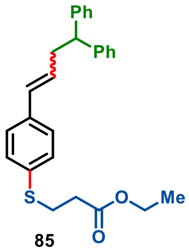

85

Following general procedure V**III**, 1-bromo-4-(2-bromovinyl)benzene (53 mg, 0.20 mmol, 1.0 equiv), ethene-1,1-diyldibenzene (54 mg, 0.30 mmol, 1.5 equiv), DIPEA (39 mg, 0.30 mmol, 1.5 equiv) and 4CzIPN (4.0 mg, 2.5 mol%), were stirred for 24 h. In the next step, 3-mercaptopropanoate (39 µL, 0.30 mmol, 1.5 equiv), 4CzIPN (0.8 mg, 0.5 mol%) $NiBr_2$·glyme (3.2 mg, 5.0 mol%), dOMebpy (4.3 mg, 10 mol%) were added and stirred for another 12h. After the workup, the organic phases were concentrated under vacuum. The crude product was purified by flash chromatography using silica, EtOAc:Hexane to afford the product **85** (30 mg, 36%, 60:40 *dr*).

- **$^{1}$H NMR (400 MHz, $CDCl_3$)** δ 7.36 – 7.05 (m, 16H), 4.07 (dddd, *J* = 11.7, 7.2, 4.4, 2.2 Hz, 3H), 3.18 – 2.91 (m, 3H), 2.67 – 2.52 (m, 3H), 1.17 (td, *J* = 7.1, 3.3 Hz, 3H).
- **$^{13}$C NMR (101 MHz, $CDCl_3$)** δ 171.71, 171.59, 144.19, 143.24, 141.67, 139.65, 139.33, 135.89, 133.46, 131.01, 129.76, 129.66, 129.33, 129.16, 128.51, 128.42, 128.27, 128.23, 127.99, 127.85, 127.54, 126.63, 126.27, 124.97, 60.78, 60.71, 51.45, 38.08, 35.54, 34.86, 34.72, 34.45, 29.69, 29.02, 27.76, 14.16.
- **HRMS (ESI):** calcd. for $C_{27}H_{29}O_2S^+$ $[M+H]^+$ 417.1888; found: 417.1887

**Ethyl 3-((4-(diphenylphosphoryl)styryl)thio)propanoate (86)**

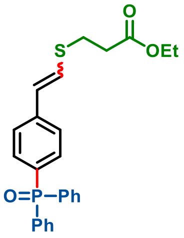

86

Following general procedure **IX**, 1-bromo-4-(2-bromovinyl)benzene (63 mg, 0.24 mmol, 1.2 equiv), ethyl 3-mercaptopropanoate (26 µL, 0.20 mmol, 1.0 equiv), and 4CzIPN (4.0 mg, 2.5 mol%) were stirred for 12h. In the next step, diphenylphosphine oxide (61 mg, 0.30 mmol, 1.5 equiv), 4CzIPN (0.8 mg, 0.5 mol%) $NiBr_2$·glyme (3.2 mg, 5.0 mol%), dOMebpy (4.3 mg, 10 mol%) were added and stirred for another 24h. After the workup, the organic phases were concentrated under vacuum. The crude product was purified by flash chromatography using silica, EtOAc:Hexane to afford the product **86** (32 mg, 46%, 60:40 *dr*).

- **$^{1}$H NMR (400 MHz, $CD_3CN$)** δ 7.68 – 7.39 (m, 14H), 7.05 (d, *J* = 15.8 Hz, 0.5H), 6.59 – 6.45 (m, 1.5H), 4.09 (qd, *J* = 7.1, 3.3 Hz, 2H), 3.12 – 3.00 (m, 2H), 2.67 (td, *J* = 7.0, 3.9 Hz, 2H), 1.19 (td, *J* = 7.1, 5.6 Hz, 3H).

- **$^{13}$C NMR (101 MHz, $CD_3CN$)** δ 172.3, 133.2, 133.1, 133.02, 133.00, 132.8, 132.7, 132.6, 131.8, 129.8, 129.6 (d, *J* = 12.1 Hz), 129.4 (d, *J* = 12.2 Hz), 126.4 (d, *J* = 12.2 Hz), 125.8, 125.1, 61.4, 35.9, 35.2, 31.5, 28.0, 14.5.
- **$^{31}$P NMR (162 MHz, $CD_3CN$)** δ 28.5, 28.4.
- **HRMS (ESI):** calcd. for $C_{25}H_{26}O_3PS^+$ $[M+H]^+$ 437.1340; found: 437.1338

**Ethyl 3-((4-((dimethyl(oxo)-λ$^6$-sulfaneylidene)amino)styryl)thio)propanoate (87)**

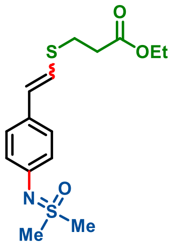

87

Following general procedure **IX**, 1-bromo-4-(2-bromovinyl)benzene (63 mg, 0.24 mmol, 1.2 equiv), ethyl 3-mercaptopropanoate (26 µL, 0.20 mmol, 1.0 equiv), and 4CzIPN (4.0 mg, 2.5 mol%) were stirred for 12h. In the next step, iminodimethyl- λ$^6$-sulfanone (28 mg, 0.30 mmol, 1.5 equiv), 4CzIPN (0.8 mg, 0.5 mol%) $NiBr_2$·glyme (3.2 mg, 5.0 mol%), dOMebpy (4.3 mg, 10 mol%) and $^t$BuNH$_2$ (55 µL, 0.44 mmol, 2.5 equiv) were added and stirred for another 24h. After the workup, the organic phases were concentrated under vacuum. The crude product was purified by flash chromatography using silica, EtOAc:Hexane to afford the product **87** (34 mg, 52%, 60:40 *dr*)

- **$^1$H NMR (400 MHz, $CD_3CN$)** δ 7.37 – 7.29 (m, 1.3H), 7.25 – 7.19 (m, 0.7H), 7.04 – 6.91 (m, 2H), 6.68 (d, *J* = 15.6 Hz, 0.5H), 6.56 – 6.37 (m, 1H), 6.20 (d, *J* = 10.8 Hz, 0.5H), 4.13 (q, *J* = 7.1 Hz, 2H), 3.13 (d, *J* = 3.6 Hz, 6H), 3.04 (td, *J* = 7.1, 1.2 Hz, 2H), 2.69 (td, *J* = 7.1, 1.4 Hz, 2H), 1.23 (td, *J* = 7.1, 2.2 Hz, 3H).
- **$^{13}$C NMR (101 MHz, $CD_3CN$)** δ 172.5, 172.4, 146.5, 146.1, 131.2, 131.2, 130.4, 129.0, 127.4, 126.4, 125.2, 123.9, 123.4, 122.5, 61.4, 61.3, 42.4, 42.3, 35.3, 31.2, 28.4, 14.5.
- **HRMS (ESI):** calcd. for $C_{15}H_{22}NO_3S_2^+$ $[M+H]^+$ 328.1041; found: 328.1032

**Methyl *N*$^τ$-(4-(2-bromovinyl)phenyl)-*N*$^α$-(tert-butoxycarbonyl)-*L*-histidinate (88)**

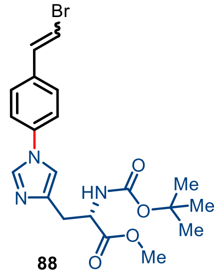

88

A 5 mL crimp-top vial was filled with 1-bromo-4-(2-bromovinyl)benzene (54 mg, 0.20 mmol, 1.0 equiv), the corresponding methyl (*tert*-butoxycarbonyl)-*L*-histidinate (81 mg, 0.30 mmol, 1.5 equiv), TMG (36 mg, 1.5 equiv) and 4CzIPN (0.8 mg, 0.5 mol%), $NiBr_2$·glyme (3.2 mg, 5.0 mol%) were added. The vial was then crimped. Solvent (0.4 mL) was added to the mixture. The vial was degassed and refilled with nitrogen using the Schlenk line technique (three times) and stirred for 12 h at 60 °C under irradiation of a single blue LED (455 (± 15) nm). The reaction mixture was quenched with $H_2O$ (15 mL). The resulting mixture was extracted with EtOAc (2 × 15 mL). The combined organic phases were dried over $Na_2SO_4$. The solvent was

removed by vacuum. Finally, the crude product was purified by flash column chromatography using silica, EtOAc:Hexane to afford the product **88** (52 mg, 57%, 80:20 *dr*).

- **$^{1}$H NMR (400 MHz, CDCl$_3$)** δ 7.52 – 7.41 (m, 3H), 7.31 – 7.26 (m, 0.5H)., 6.93 (d, *J* = 8.3 Hz, 1.5H), 6.71 (d, *J* = 9.2 Hz, 1H), 6.68 – 6.61 (m, 1H), 6.29 (d, *J* = 9.2 Hz, 1H), 5.80 (d, *J* = 8.4 Hz, 1H), 4.62 – 4.46 (m, 1H), 3.71 (d, *J* = 16.3 Hz, 3H), 3.16 – 2.97 (m, 2H), 1.43 (d, *J* = 2.2 Hz, 9H).
- **$^{13}$C NMR (101 MHz, CDCl$_3$)** δ 172.2, 155.5, 136.4, 132.2, 132.1, 130.1, 127.7, 123.1, 122.6, 116.1, 79.8, 53.3, 52.3, 29.9, 28.3.
- **HRMS (ESI):** calcd. for $C_{20}$ $H_{25}$ Br $N_3$ $O_4$ Na $[M+H]^+$ 450.1028; found: 450.1027

**Methyl *N*-(tert-butoxycarbonyl)-*S*-(4-(4-((*S*)-2-((tert-butoxycarbonyl)amino)-3-methoxy-3-oxopropyl)-1*H*-imidazol-1-yl)styryl)-*L*-cysteinate (89)**

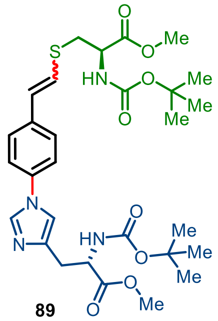


89

In the second step, the compound **88** (95 mg, 0.2 mmol, 1.0 equiv), the respective nucleophile methyl (tert-butoxycarbonyl)-*L*-cysteinate (70 mg, 0.30 mmol, 1.5 equiv), 4CzIPN (2.5 mg, 1.5 mol%) were added . The vial was degassed and refilled with nitrogen using the Schlenk-line technique (three times). The mixture was stirred for 12 h at room temperature under the irradiation of a single blue LED (455 (± 15) nm). The reaction mixture was quenched with $H_2O$ (15 mL). The resulting mixture was extracted with EtOAc (2 × 15 mL). The combined organic phases were dried over $Na_2SO_4$. The solvent was removed by vacuum. Finally, the crude product was purified by flash column chromatography using silica, EtOAc:Hexane to afford the product **89** (52 mg, 43%, 70:30 *dr*)

- **$^{1}$H NMR (400 MHz, CD$_3$CN)** δ 7.80 (dd, *J* = 12.7, 1.4 Hz, 0.6H), 7.55 – 7.44 (m, 2.4H), 7.38 (m, 2H), 7.27 – 7.18 (m, 0.7H), 6.98 – 6.91 (m, 1H), 6.86 – 6.74 (m, 1H), 6.43 (dd, *J* = 28.8, 9.2 Hz, 0.3H), 6.36 – 6.13 (m, 1.5H), 5.73 (s, 0.5H), 4.45 – 4.28 (m, 2H), 3.65 – 3.57 (m, 6H), 2.99 (t, *J* = 5.9 Hz, 3H), 2.92 (d, *J* = 5.6 Hz, 1H), 1.39 (d, *J* = 2.7 Hz, 18H).
- **$^{13}$C NMR (101 MHz, CD$_3$CN)** δ 173.3, 156.4, 137.7, 137.5, 135.3, 134.0, 132.8, 132.7, 132.5, 131.98, 131.95, 131.4, 129.9, 128.9, 127.1, 124.2, 121.8, 117.6, 115.1, 80.0, 54.5, 53.1, 52.7, 52.7, 33.3, 30.3, 29.9, 28.5.
- **HRMS (ESI):** Not detected in HRMS (ESI, CI)

**Methyl (S)-3-(4-(4-(2-bromovinyl)phenoxy)phenyl)-2-((tert-butoxycarbonyl)amino)propanoate (90)**

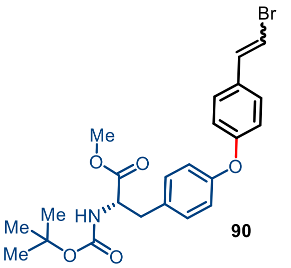


**90**

A 5 mL crimp-top vial was filled with 1-bromo-4-(2-bromovinyl)benzene (54 mg, 0.20 mmol, 1.0 equiv), the corresponding methyl (*tert*-butoxycarbonyl)-*L*-tyrosinate (90 mg, 0.30 mmol, 1.5 equiv), ${}^{t}BuNH_2$ (33 µL, 0.3 mmol, 1.5 equiv), and 4CzIPN (0.8 mg, 0.5 mol%), $NiBr_2$·glyme (3.2 mg, 5.0 mol%) were added. The vial was then crimped. Solvent (0.4 mL) was added to the mixture. The vial was degassed and refilled with nitrogen using the Schlenk line technique (three times) and stirred for 12 h at 60 °C under irradiation of a single blue LED (455 (± 15) nm). The reaction mixture was quenched with $H_2O$ (15 mL). The resulting mixture was extracted with EtOAc (2 × 15 mL). The combined organic phases were dried over $Na_2SO_4$. The solvent was removed by vacuum. Finally, the crude product was purified by flash column chromatography using silica, EtOAc:Hexane to afford the product **90** (66 mg, 69%,70:30 *dr*).

- **$^1$H NMR (400 MHz, $CDCl_3$)** δ 7.55 – 7.51 (m, 0.5H), 7.45 – 7.39 (m, 1.5H), 7.17 – 7.08 (m, 4H), 7.04 (dq, *J* = 9.2, 2.6 Hz, 0.5H), 7.00 – 6.95 (m, 1.5H), 6.60 (d, *J* = 6.9 Hz, 0.5H), 6.25 (d, *J* = 12.4 Hz, 1H), 5.54 (d, *J* = 6.9 Hz, 0.5H), 5.01 (d, *J* = 8.4 Hz, 1H), 4.57 (q, *J* = 6.6 Hz, 1H), 3.72 (s, 3H), 3.18 – 3.06 (m, 1H), 3.01 (d, *J* = 14.7 Hz, 1H), 1.42 (s, 9H).
- **$^{13}$C NMR (101 MHz, $CDCl_3$)** δ 172.2, 156.0, 154.9, 143.9, 142.3, 134.1, 133.7, 131.7, 131.4, 131.1, 130.6, 130.2, 127.1, 120.1, 117.1, 116.9, 112.4, 79.9, 54.4, 52.2, 37.6, 28.3.
- **HRMS (ESI):** calcd. for $C_{23}H_{26}BrNO_5Na$ $[M+Na]^+$ 498.0892; found: 498.0894

**Methyl (*S*)-2-((tert-butoxycarbonyl)amino)-3-(4-(4-(2-(diphenylphosphoryl)vinyl)phenoxy)phenyl)propanoate (91)**

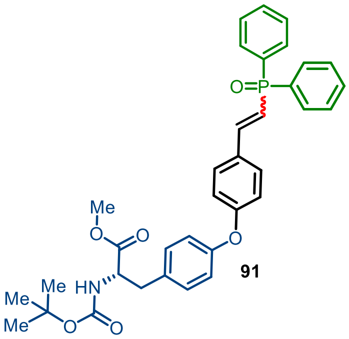


**91**

In the second step, the compound **90** (95 mg, 0.2 mmol, 1.0 equiv), respective nucleophile diphenylphosphine oxide (60 mg, 0.30 mmol, 1.5 equiv), 4CzIPN (0.8 mg, 0.5 mol%), $NiBr_2$·glyme (3.2 mg, 5.0 mol%), dOMebpy (10 mol%) and 0.4 mL DMA were added. The vial was degassed and refilled with nitrogen using the Schlenk-line technique (three times). The mixture was stirred for 24 h at room temperature under the irradiation of a single blue LED (455 (± 15) nm). The reaction mixture was quenched with $H_2O$ (15 mL). The resulting mixture was extracted with EtOAc (2 × 15 mL). The combined organic phases were dried over $Na_2SO_4$. The solvent was removed by vacuum. Finally, the crude product was

purified by flash column chromatography using silica, EtOAc:Hexane to afford the product **91** (50 mg, 41%,75:25 *dr*).

- **$^{1}$H NMR (400 MHz, $CDCl_3$)** δ 7.75 – 7.51 (m, 9H), 7.48 – 7.40 (m, 5H), 7.35 (ddd, *J* = 7.7, 4.8, 3.0 Hz, 1.5H), 7.14 – 7.08 (m, 1.5H), 7.04 – 6.93 (m, 1.5H), 6.67 (d, *J* = 6.9 Hz, 0.5H), 6.30 (d, *J* = 12.4 Hz, 0.5H), 5.62 (d, *J* = 6.9 Hz, 0.5H), 5.00 (d, *J* = 8.2 Hz, 1H), 4.56 (q, *J* = 6.6 Hz, 1H), 3.69 (d, *J* = 15.4 Hz, 3H), 3.10 (dd, *J* = 13.9, 5.8 Hz, 1H), 3.04 – 2.89 (m, 1H), 1.41 (s, 9H).
- **$^{13}$C NMR (101 MHz, $CDCl_3$)** δ 172.2, 156.0, 155.8, 154.9, 145.6, 143.8, 139.1 (d, *J* = 2.9 Hz), 138.5 (d, *J* = 3.0 Hz), 133.1 (d, *J* = 7.3 Hz), 132.5 (d, *J* = 2.8 Hz), 132.4, 132.2 (d, *J* = 3.8 Hz), 132.1 (d, *J* = 2.4 Hz), 131.9 (d, *J* = 2.6 Hz), 131.86 (d, *J* = 2.7 Hz), 131.80 (d, *J* = 2.7 Hz), 128.8 (d, *J* = 12.9 Hz), 128.5 (d, *J* = 3.5 Hz), 128.3, 125.39 (d, *J* = 12.5 Hz), 117.2, 117.0, 110.7 (d, *J* = 307.1 Hz), 79.9, 54.4, 52.2, 37.6, 28.3.
- **$^{31}$P NMR (162 MHz, $CDCl_3$)** δ 30.0, 29.9.
- **HRMS (ESI):** calcd. for $C_{35}H_{36}NO_6P$ $[M+H]^+$ 598.2358; found: 598.2364

## 9. Notes and references


[1] J. Luo, J. Zhang, *ACS Catal.* **2016**, *6*, 873-877.

[2] E. Speckmeier, T. G. Fischer, K. Zeitler, *J. Am. Chem. Soc.* **2018**, *140*, 15353-15365.

[3] L. L. Miller, E. Riekena, *J. Org. Chem.* **1969**, *34*, 3359-3362.

[4] C. H. Chrisman, M. Kudisch, K. O. Puffer, T. K. Stewart, Y. M. L. Lamb, C.-H. Lim, R. Escobar, P. Thordarson, J. W. Johannes, G. M. Miyake, *J. Am. Chem. Soc.* **2023**, *145*, 12293-12304.

[5] a) Y. Fang, T. Liu, L. Chen, D. Chao, *Chem. Commun.* **2022**, *58*, 7972-7975; b) V. J. Mayerhofer, M. Lippolis, C. J. Teskey, *Angew. Chem. Int. Ed.* **2024**, *63*, e202314870; c) Z. Qu, T. Tian, Y. Tan, X. Ji, G.-J. Deng, H. Huang, *Green Chem.* **2022**, *24*, 7403-7409.

[6] a) M. Villa, A. Fermi, F. Calogero, A. Gualandi, P. Franchi, M. Lucarini, B. Ventura, P. G. Cozzi, P. Ceroni, *Angew. Chem. Int. Ed.* **2025**, 64, e202420009. b) M. Villa, A. Fermi, F. Calogero, X. Wu, A. Gualandi, P. G. Cozzi, A. Troisi, B. Ventura, P. Ceroni, *Chem. Sci.* **2024**, *15*, 14739-14745. d) K. Reynolds, B. Johnston, B. Campbell, A. Li, D. Nocera, *ChemRxiv* **2025**, DOI 10.26434/chemrxiv-2025-vnnll. This content is a preprint and has not been peer-reviewed.

[7] S. K. Pagire, T. Föll, O. Reiser, *Acc. Chem. Res.* **2020**, *53*, 782.

[8] a) R. D. Bradley, B. D. McManus, J. G. Yam, V. Carta, A. Bahamonde, *Angew. Chem. Int. Ed.* **2023**, *62*, e202310753; b) O. R. Taylor, P. J. Saucedo, A. Bahamonde, *J. Org. Chem.* **2024**, *89*, 16093-16105. c) B. D. McManus, L. C. Hung, O. R. Taylor, P. Q. Nguyen, A. L. Cedeño, K. Arriola, R. D. Bradley, P. J. Saucedo, R. J. Hannan, Y. A. Luna, P. Farias, A. Bahamonde, *J. Am. Chem. Soc.* **2024**, *146*, 32135-32146.

[9] a) N. A. Till, L. Tian, Z. Dong, G. D. Scholes, D. W. C. MacMillan, *J. Am. Chem. Soc.* **2020**, *142*, 15830-15841; b) R. Sun, Y. Qin, D. G. Nocera, *Angew. Chem. Int. Ed.* **2020**, *59*, 9527-9533. c) D. A. Cagan, D. Bím, N. P. Kazmierczak, R. G. Hadt, *ACS Catal.* **2024**, *14*, 9055-9076. d) J. Düker, M. Philipp, T. Lentner, J. A. Cadge, J. E. A. Lavarda, R. M. Gschwind, M. S. Sigman, I. Ghosh, B. König, *ACS Catal.* **2025**, *15*, 817-827.

[10] S. An, K. H. Song, S. Lee, *Org. Biomol. Chem.* **2021**, *19*, 7827-7831.

[11] G. Zhang, H. Zeng, S. Zheng, M. C. Neary, P. A. Dub, *ACS Catal.* **2022**, *12*, 5425-5429.

[12] G. W. Kabalka, S. K. Guchhait, *Org. Lett.* **2003**, *5*, 729-731.

[13] P. Woźnicki, M. Stankevič, *Eur. J. Org. Chem.* **2021**, *2021*, 3484-3491.

[14] a) H. Wang, Y. Li, Z. Tang, S. Wang, H. Zhang, H. Cong, A. Lei, *ACS Catal.* **2018**, *8*, 10599-10605; b) L. Liu, Y. Wang, Z. Zeng, P. Xu, Y. Gao, Y. Yin, Y. Zhao, *Adv. Synth. Catal.* **2013**, *355*, 659-666.

## 10. NMR spectra of the isolated compounds

$^{1}H$ NMR (400 MHz, $CDCl_3$) spectra of compound **1**

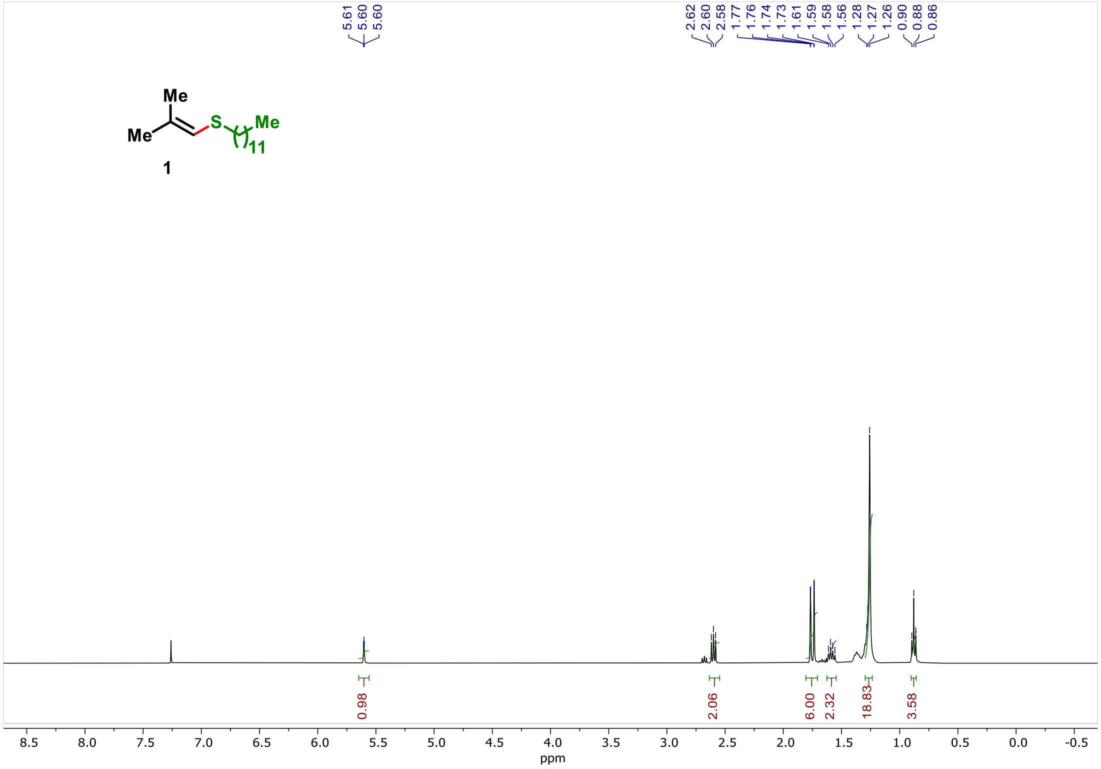


$^{13}C$ NMR (101 MHz, $CDCl_3$) spectra of compound **1**

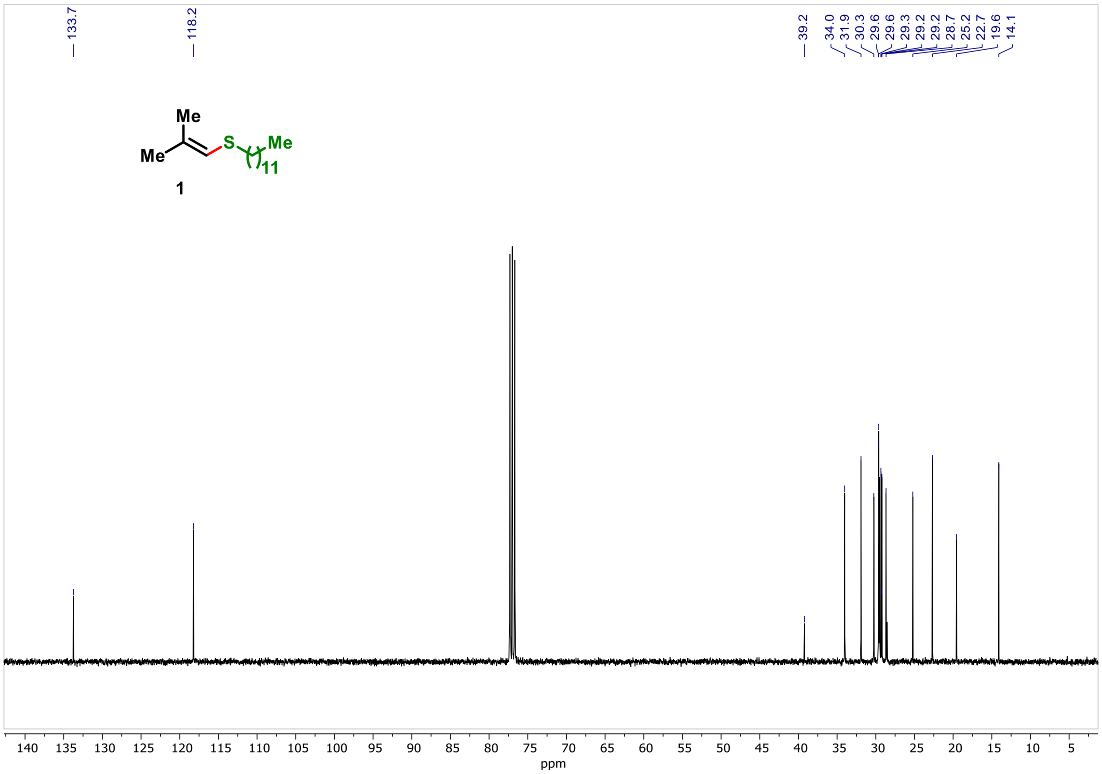

$^{1}$H NMR (400 MHz, $CDCl_3$) spectra of compound **2**

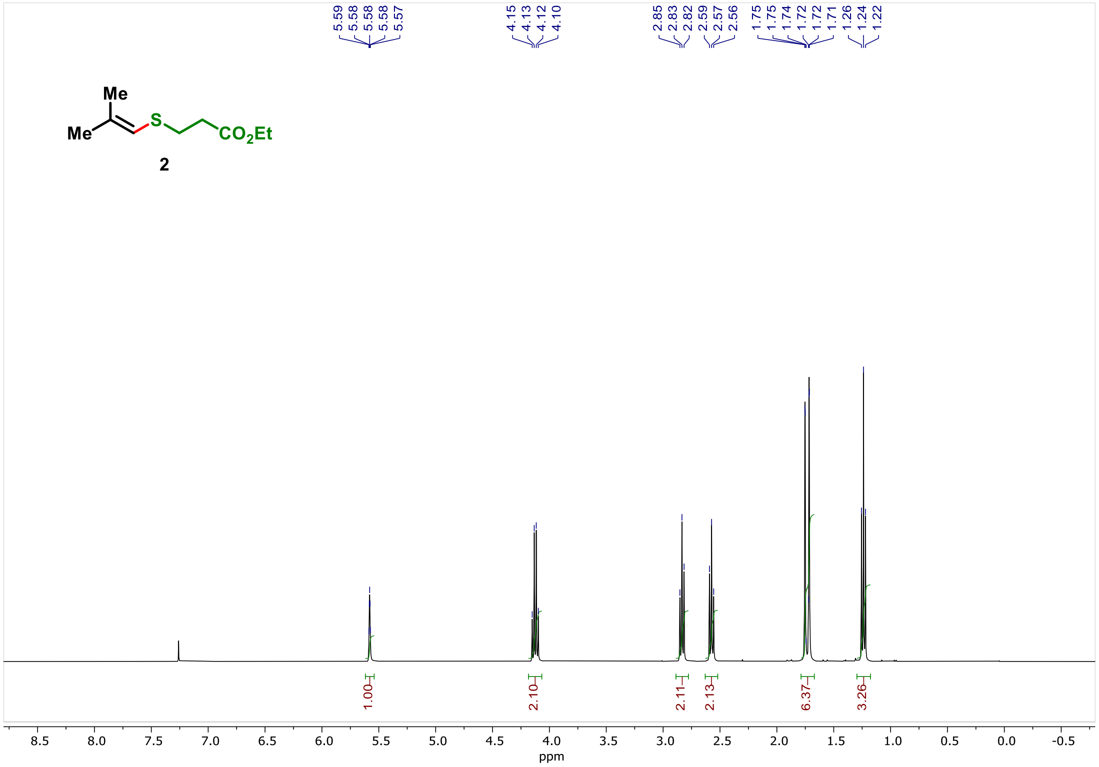

$^{13}$C NMR(101 MHz, $CDCl_3$)  spectra of compound **2**

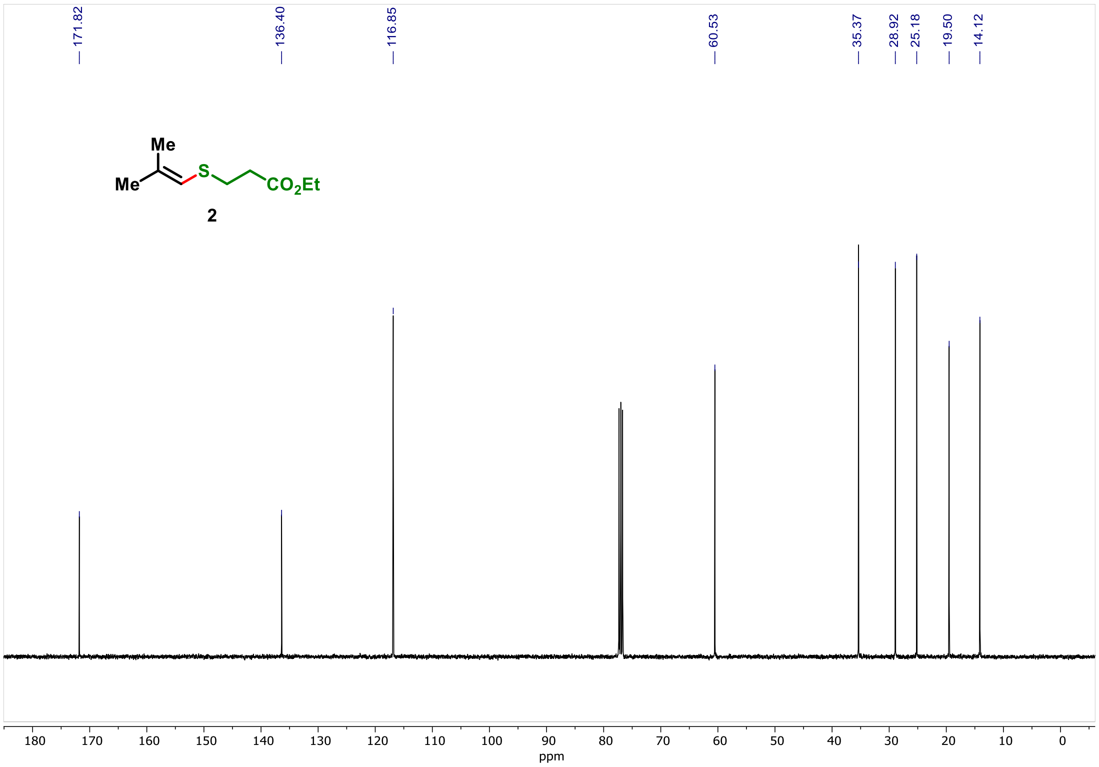

$^{1}$H NMR (400 MHz, $CD_3CN$) spectra of compound **3**

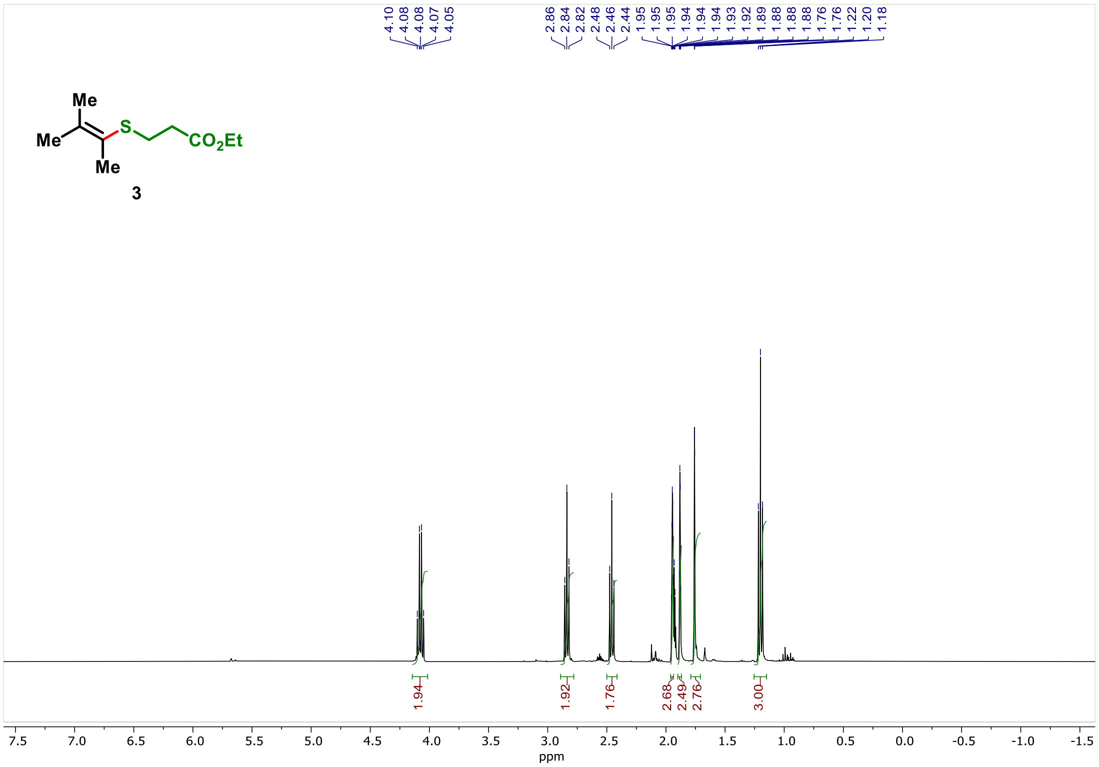

$^{13}$C NMR (101 MHz, $CD_3CN$) spectra of compound **3**

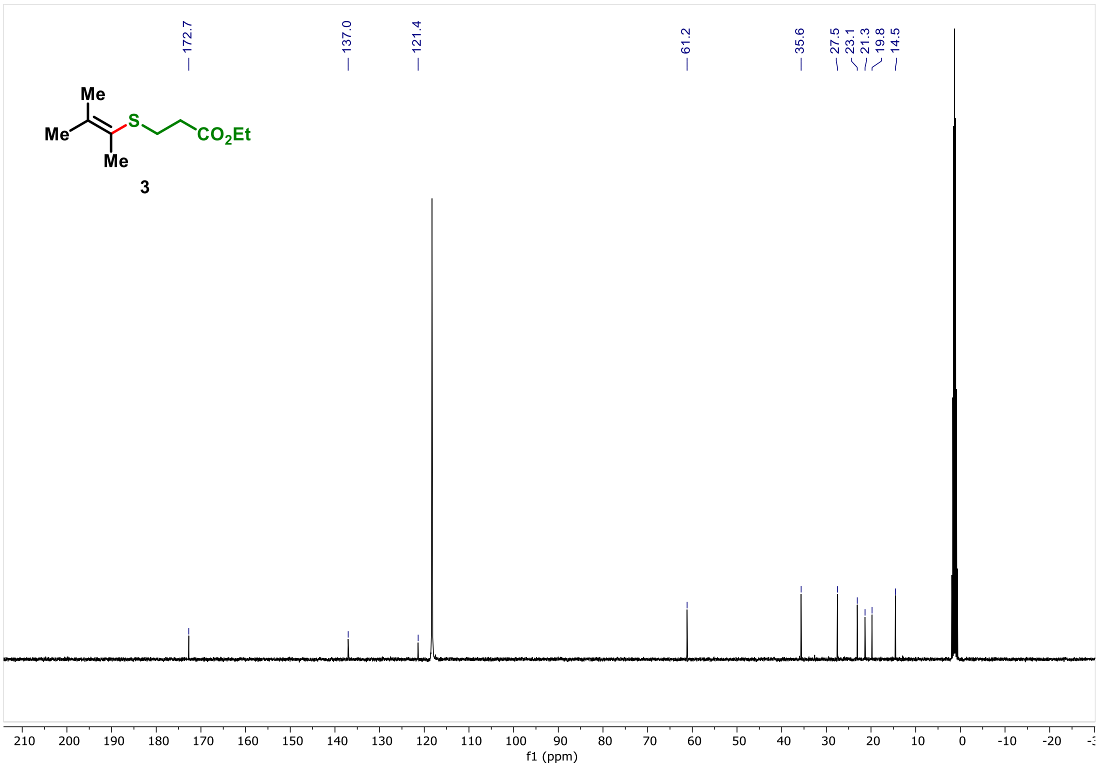

$^{1}$H NMR (400 MHz, $CD_3CN$) spectra of compound **4**

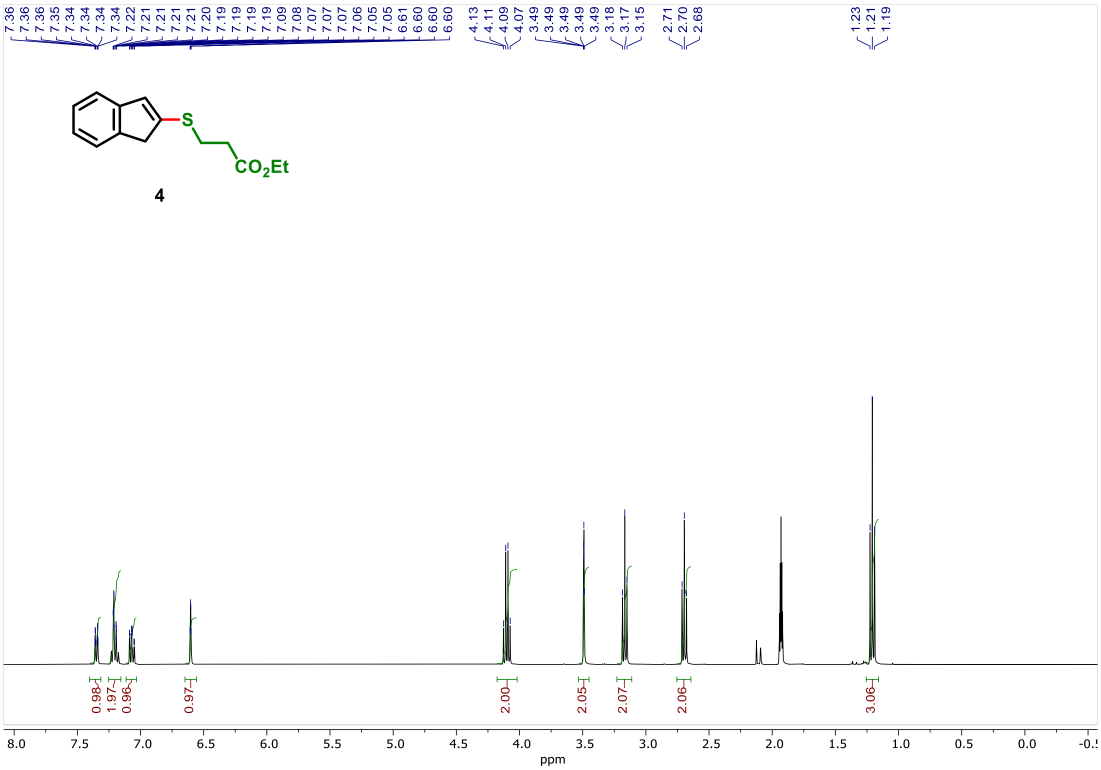


$^{13}$C NMR (101 MHz, $CD_3CN$) spectra of compound **4**

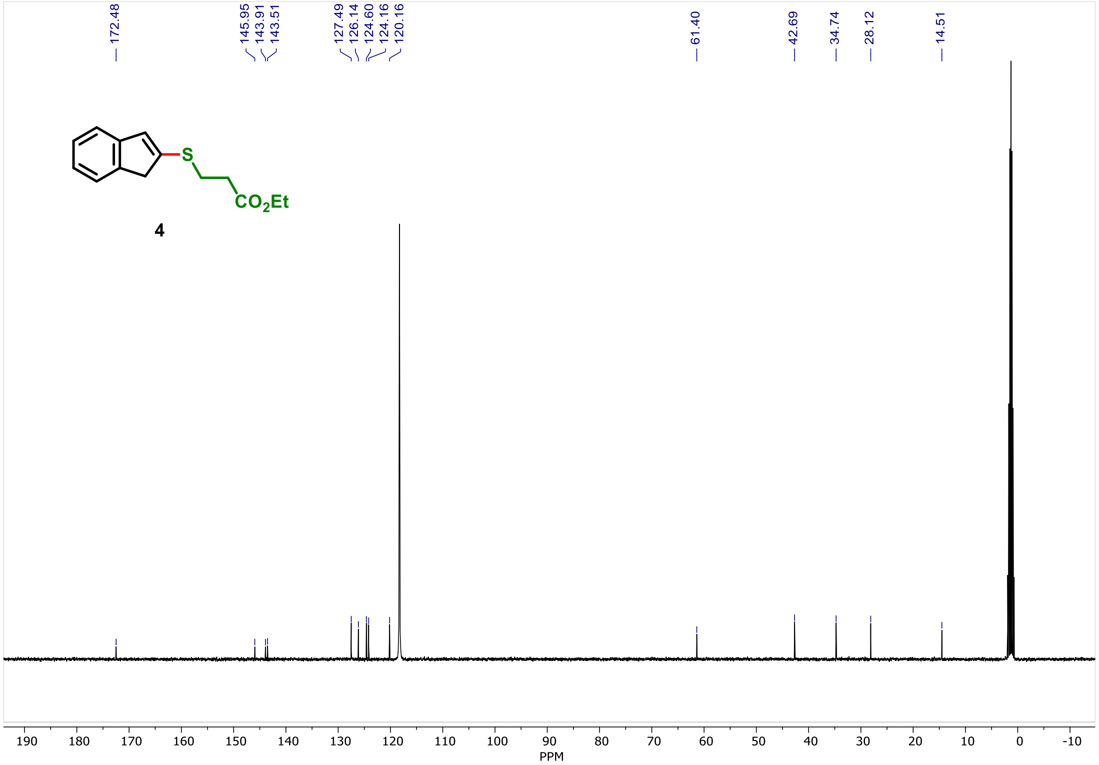

$^{1}H$ NMR (400 MHz, $CD_3CN$) spectra of compound **5**

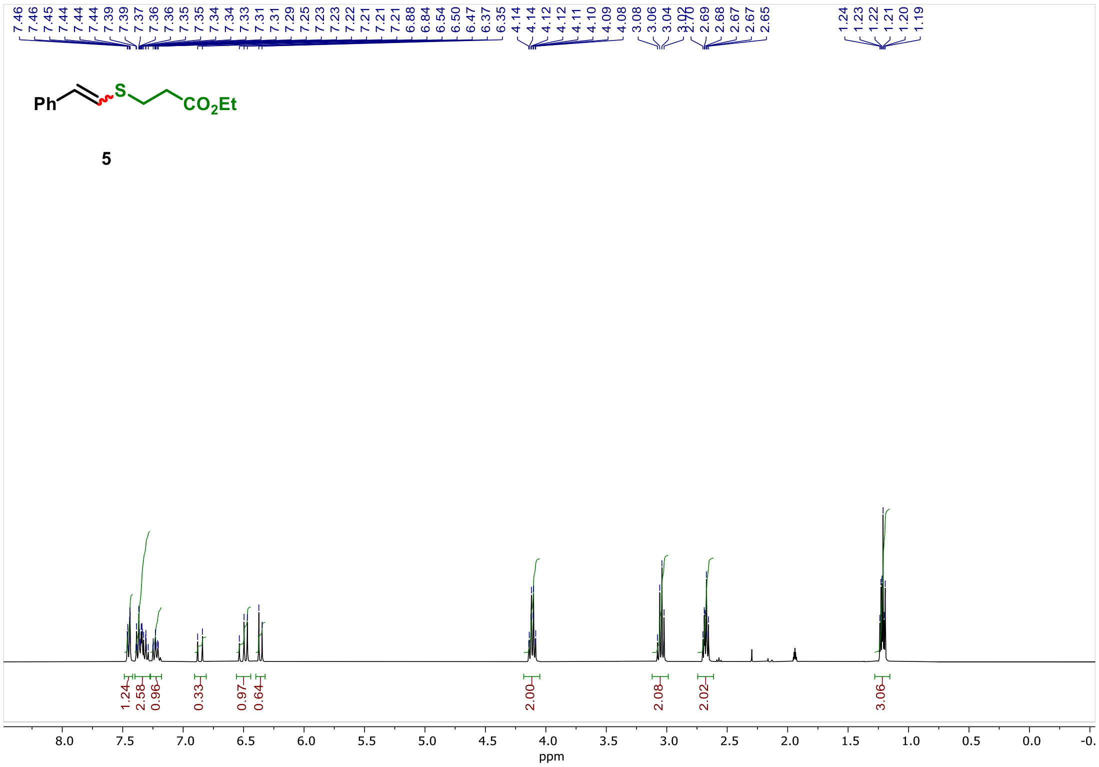

$^{13}C$ NMR (101 MHz, $CD_3CN$) spectra of compound **5**

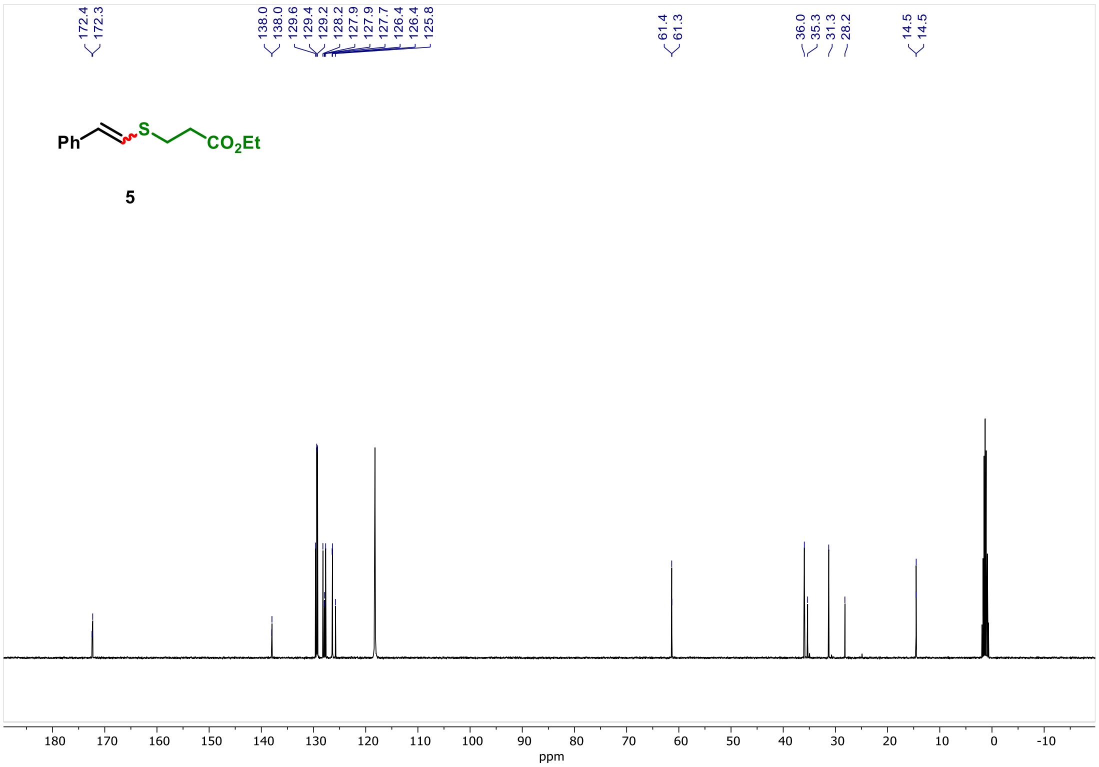

$^{1}$H NMR (400 MHz, $CDCl_3$) spectra of compound **6**

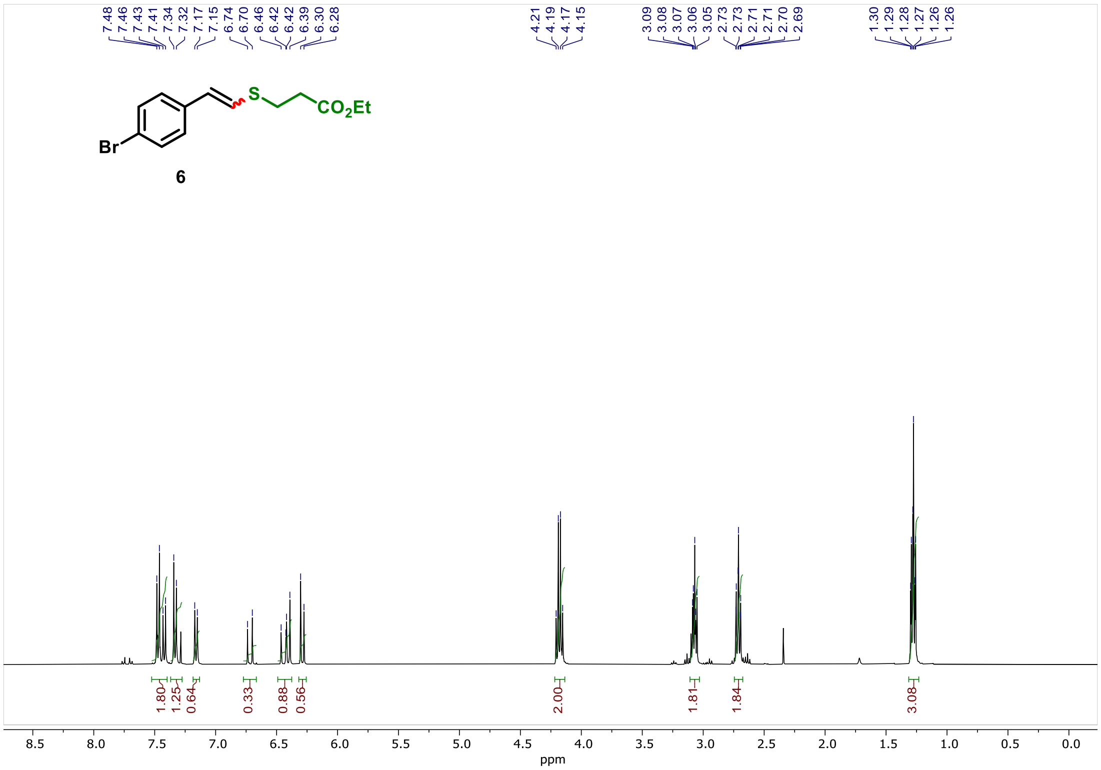


$^{13}$C NMR (101 MHz, $CDCl_3$) spectra of compound **6**

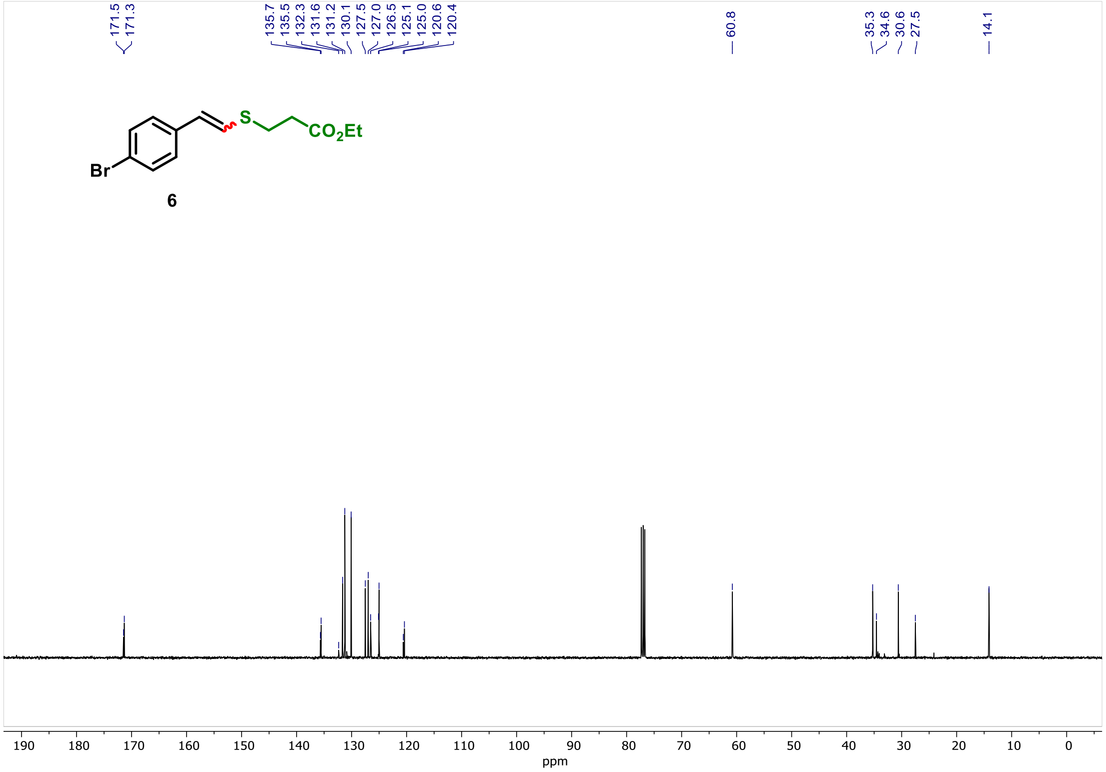

$^{1}H$ NMR (400 MHz, $CDCl_3$) spectra of compound **7**

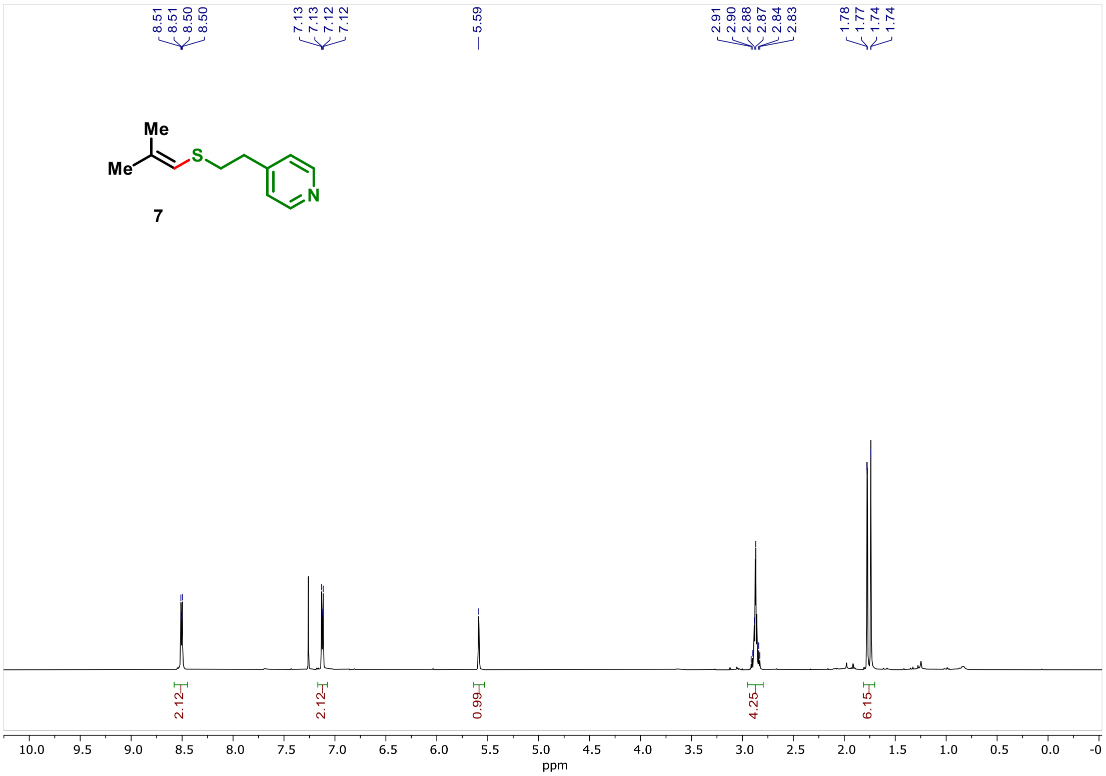


$^{13}C$ NMR (101 MHz, $CDCl_3$) spectra of compound **7**

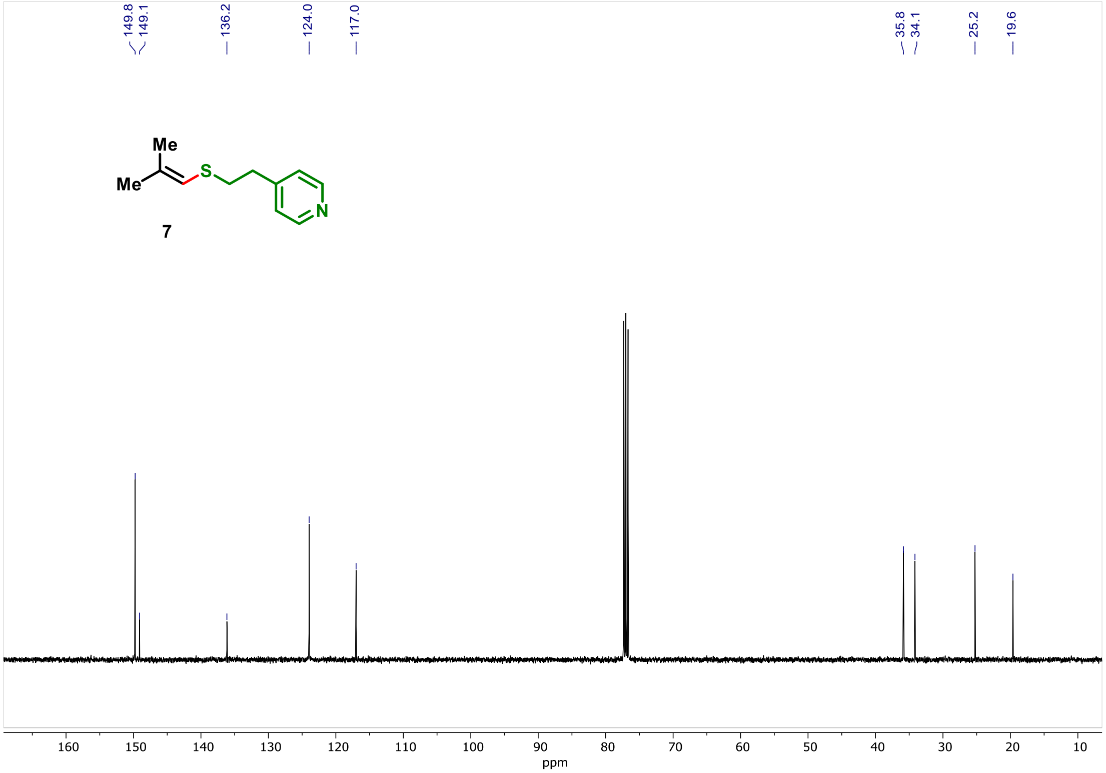

$^{1}$H NMR (400 MHz, $CDCl_3$) spectra of compound **8**

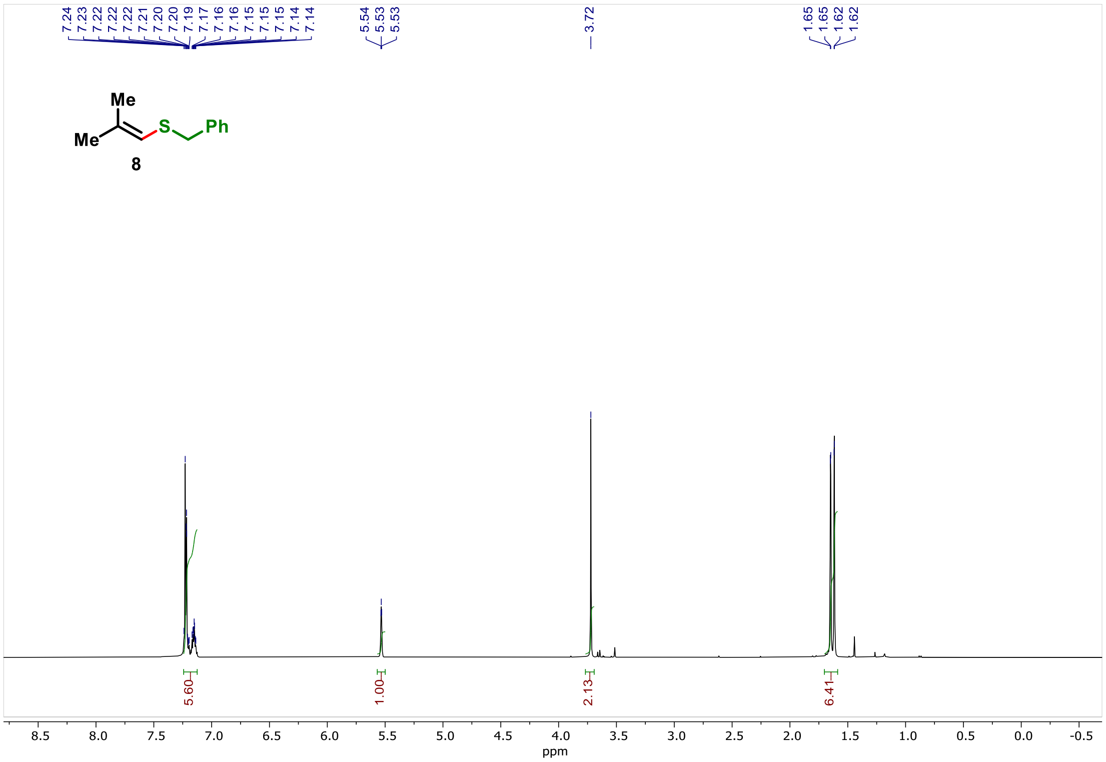


$^{13}$C NMR (101 MHz, $CDCl_3$) spectra of compound **8**

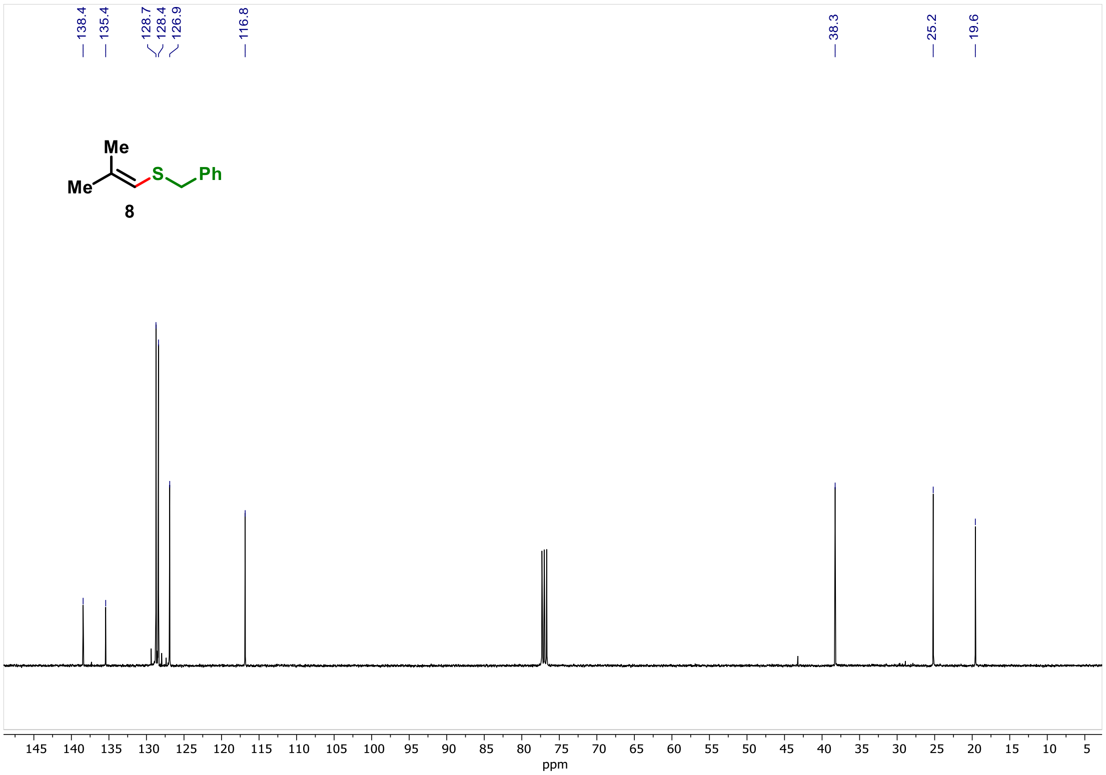

$^{1}H$ NMR (400 MHz, $CDCl_3$) spectra of compound **9**

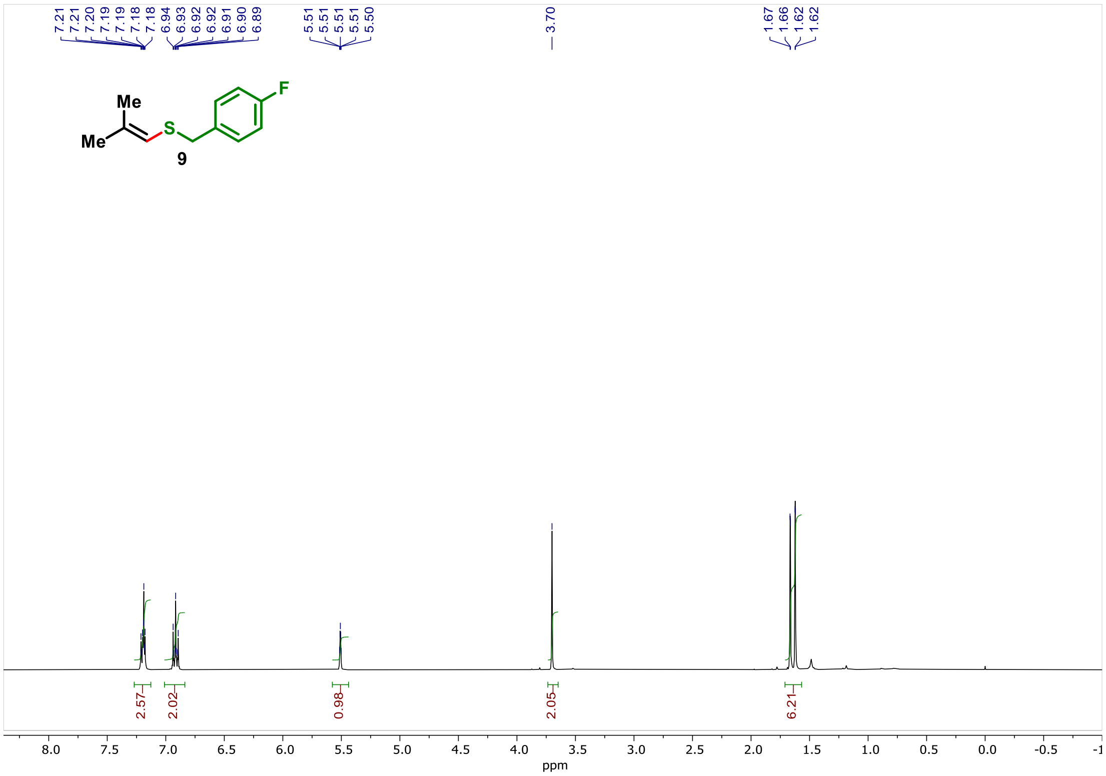


$^{13}C$ NMR (101 MHz, $CDCl_3$) spectra of compound **9**

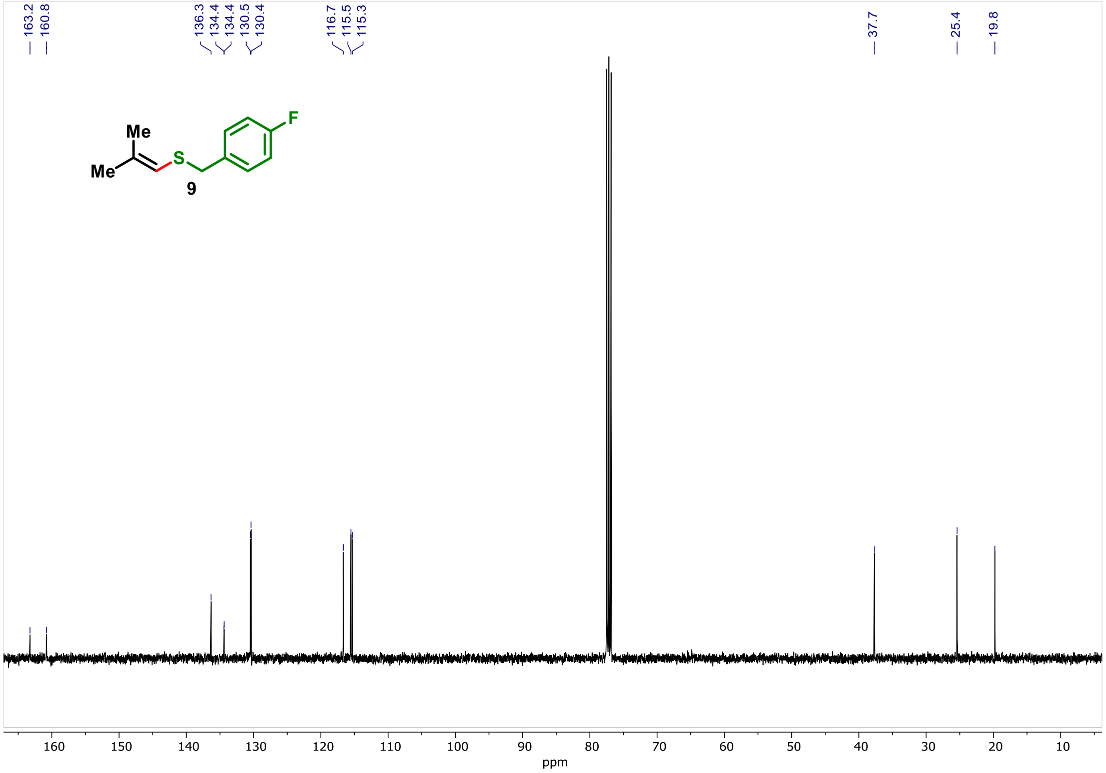

$^{19}F$ NMR (376 MHz, $CDCl_3$) spectra of compound **9**

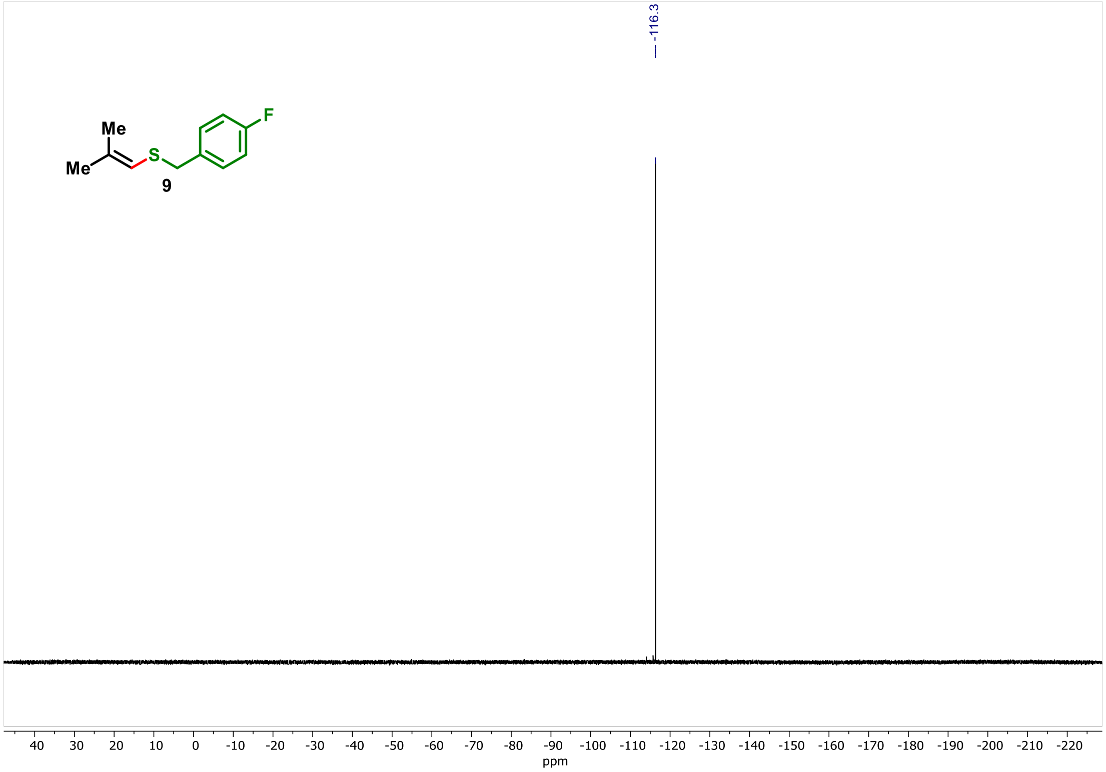

$^1H$ NMR (400 MHz, $CDCl_3$) spectra of compound **10**

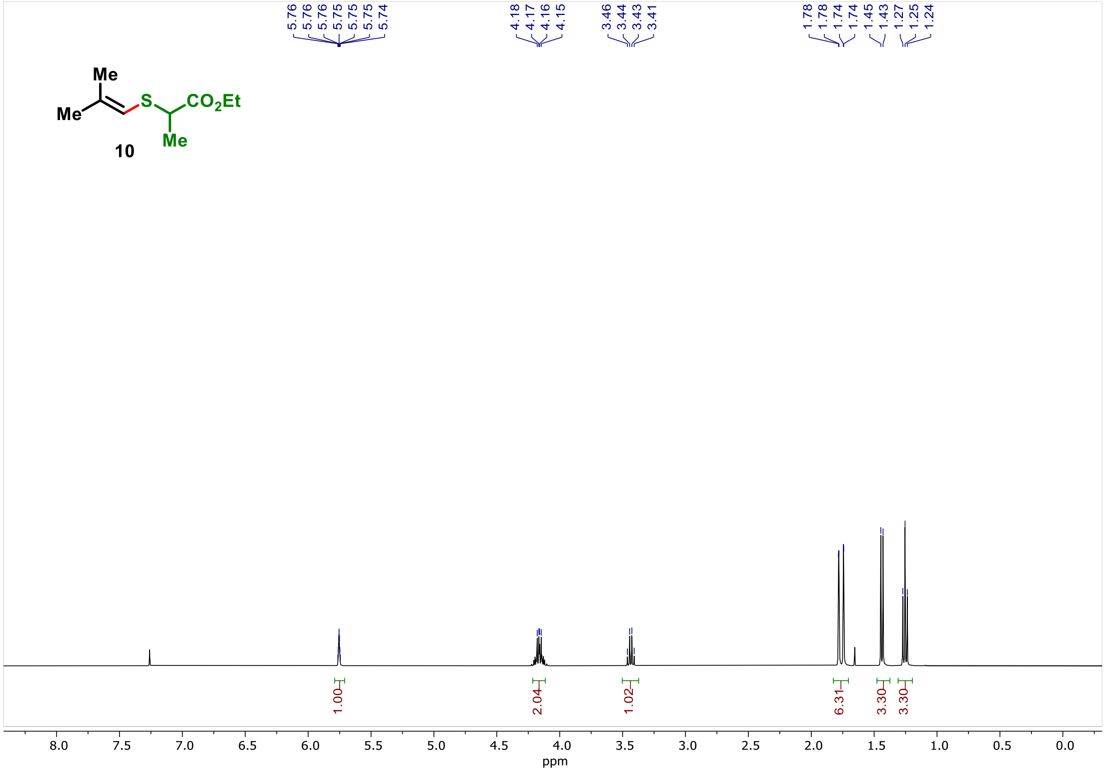


$^{13}C$ NMR (101 MHz, $CDCl_3$) spectra of compound **10**

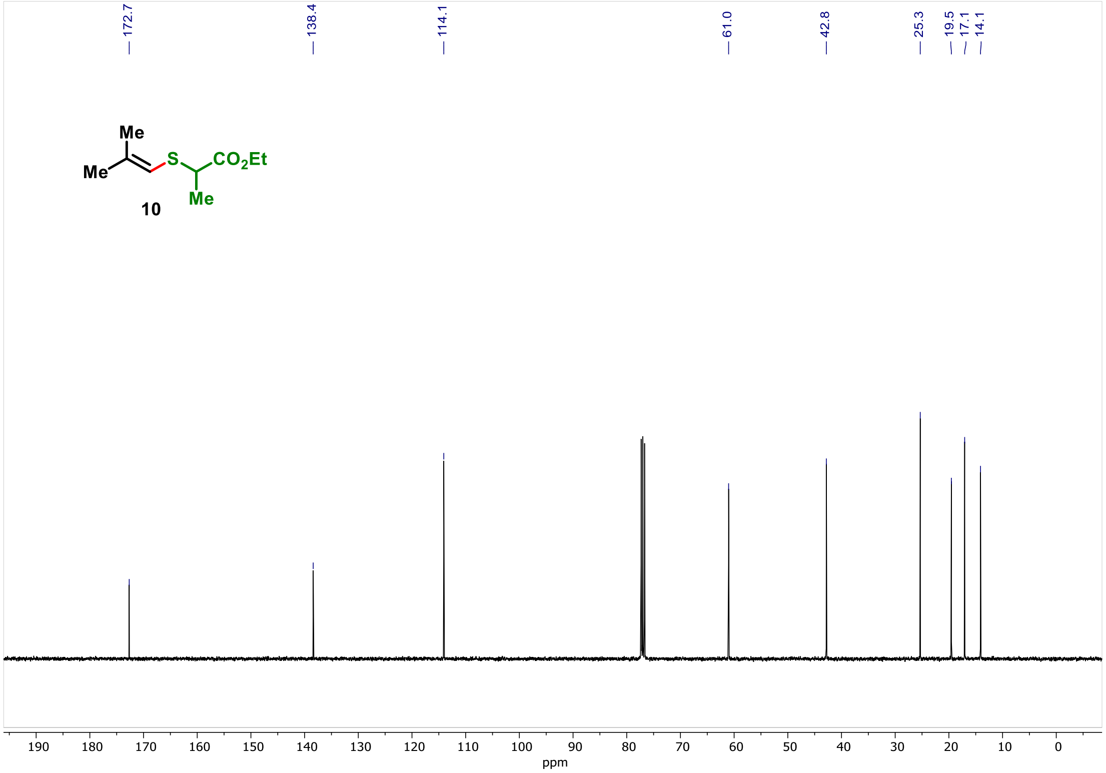

$^{1}$H NMR (400 MHz, $CDCl_3$) spectra of compound **11**

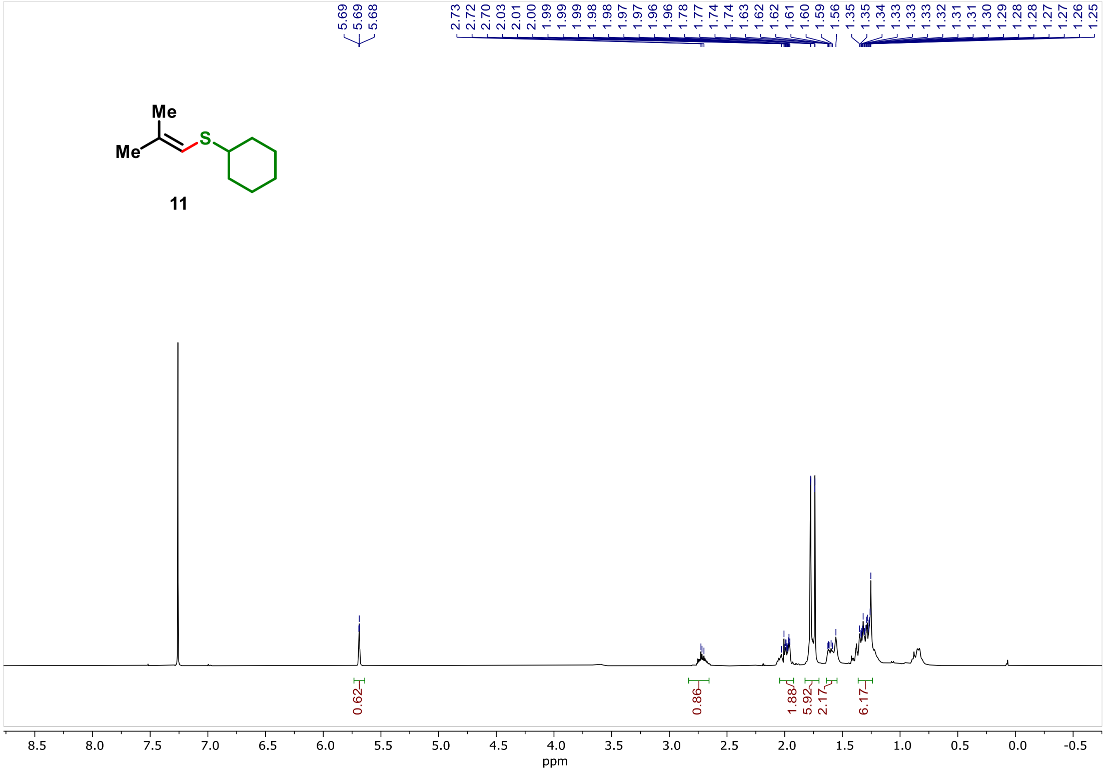


$^{13}$C NMR (101 MHz, $CDCl_3$) spectra of compound **11**

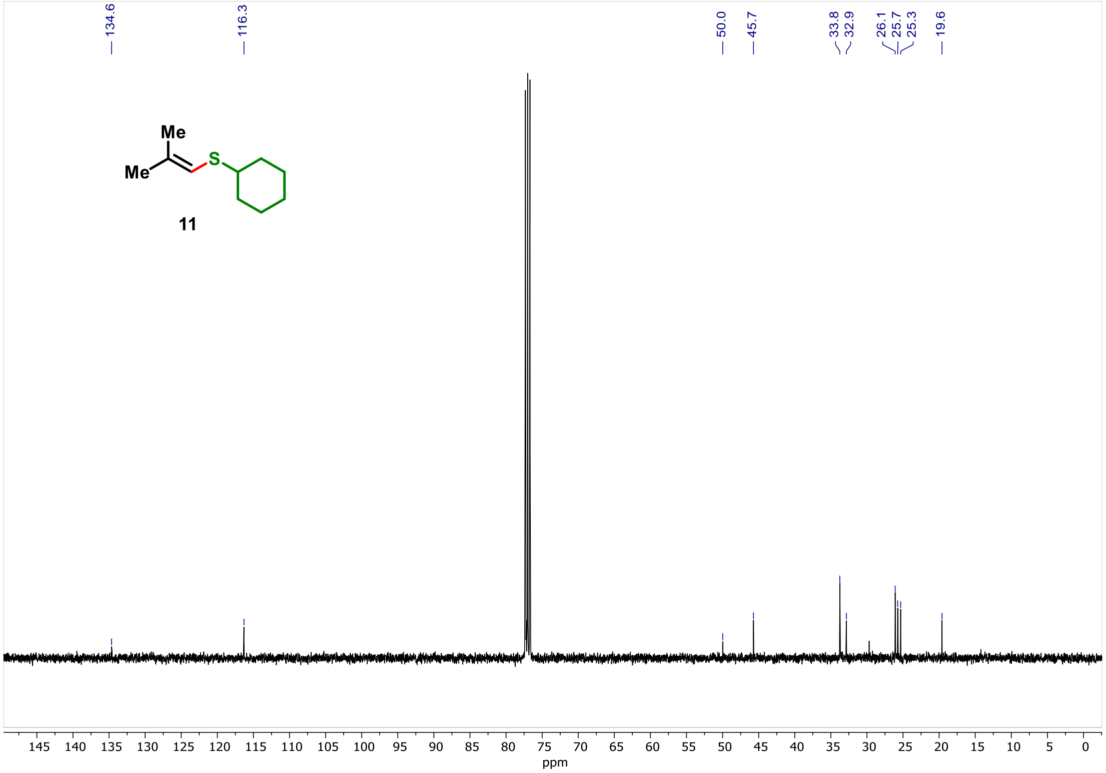

$^{1}H$ NMR (400 MHz, $CDCl_3$) spectra of compound **12**

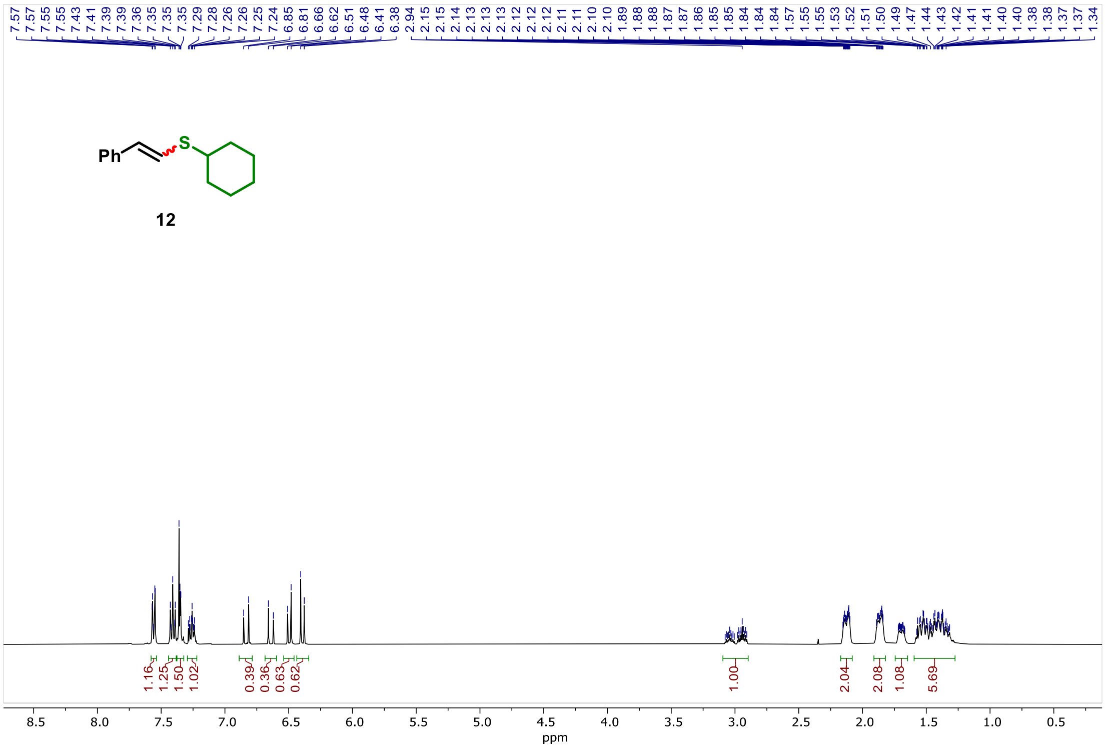

$^{13}C$ NMR (101 MHz, $CDCl_3$) spectra of compound **12**

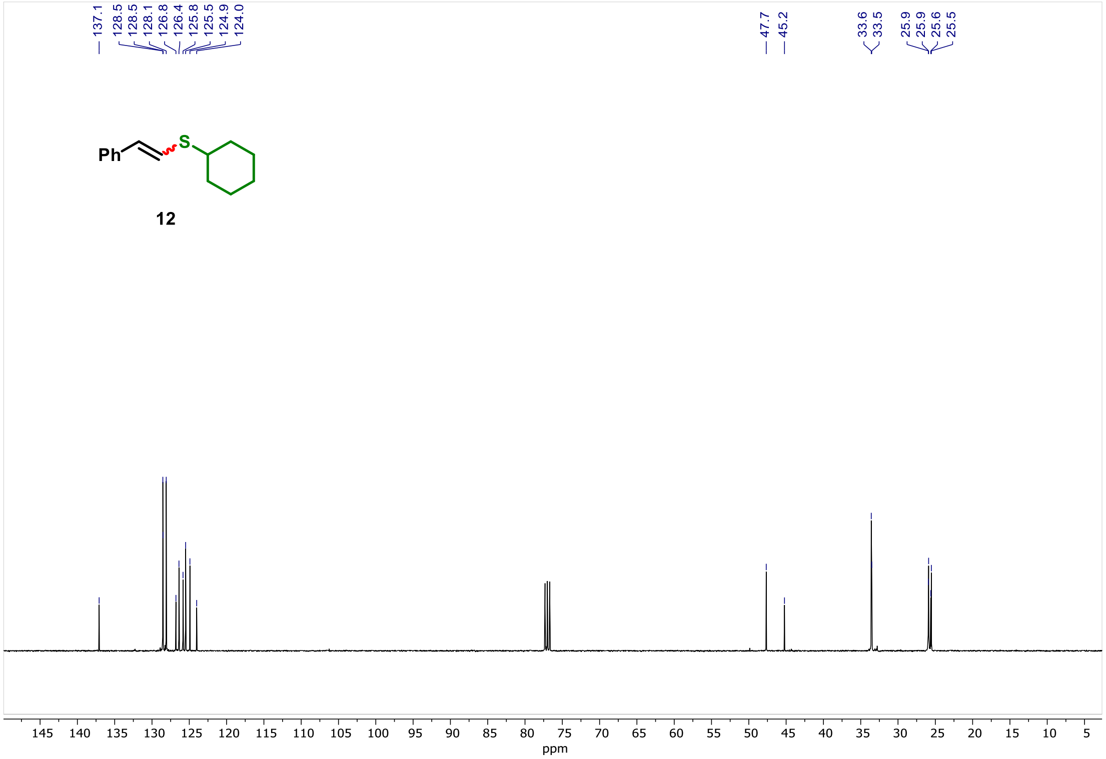

$^{1}H$ NMR (400 MHz, $CDCl_3$) spectra of compound **13**

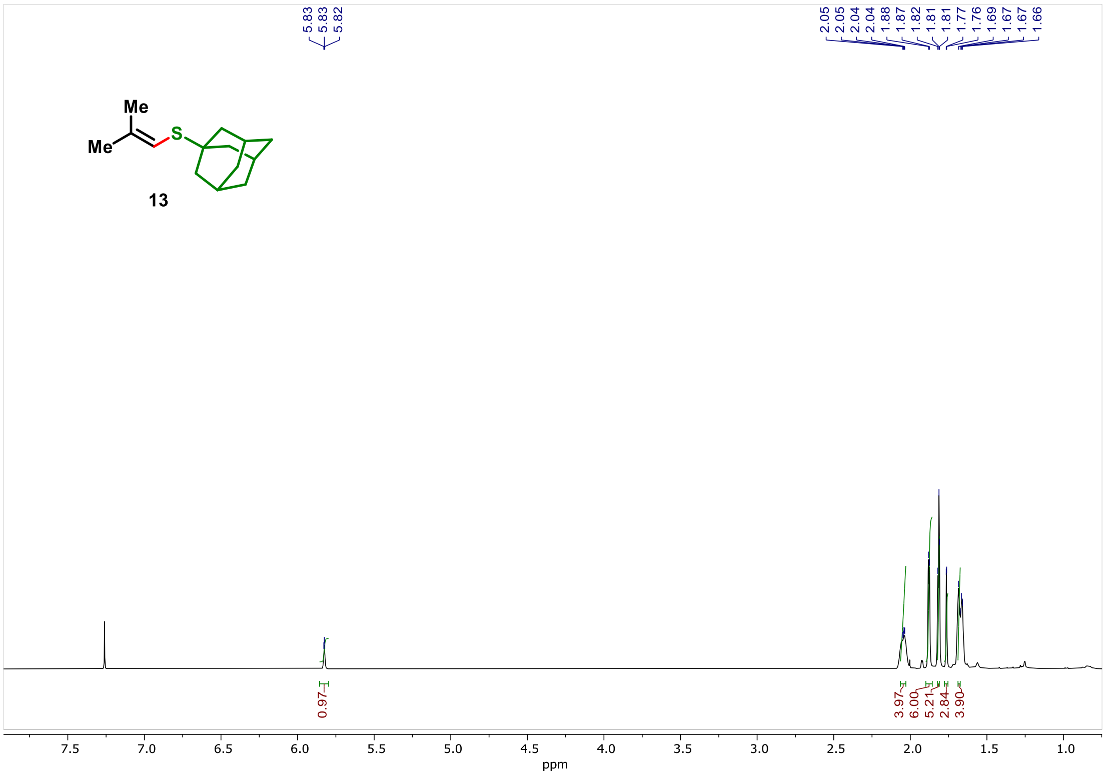


$^{13}C$ NMR (101 MHz, $CDCl_3$) spectra of compound **13**

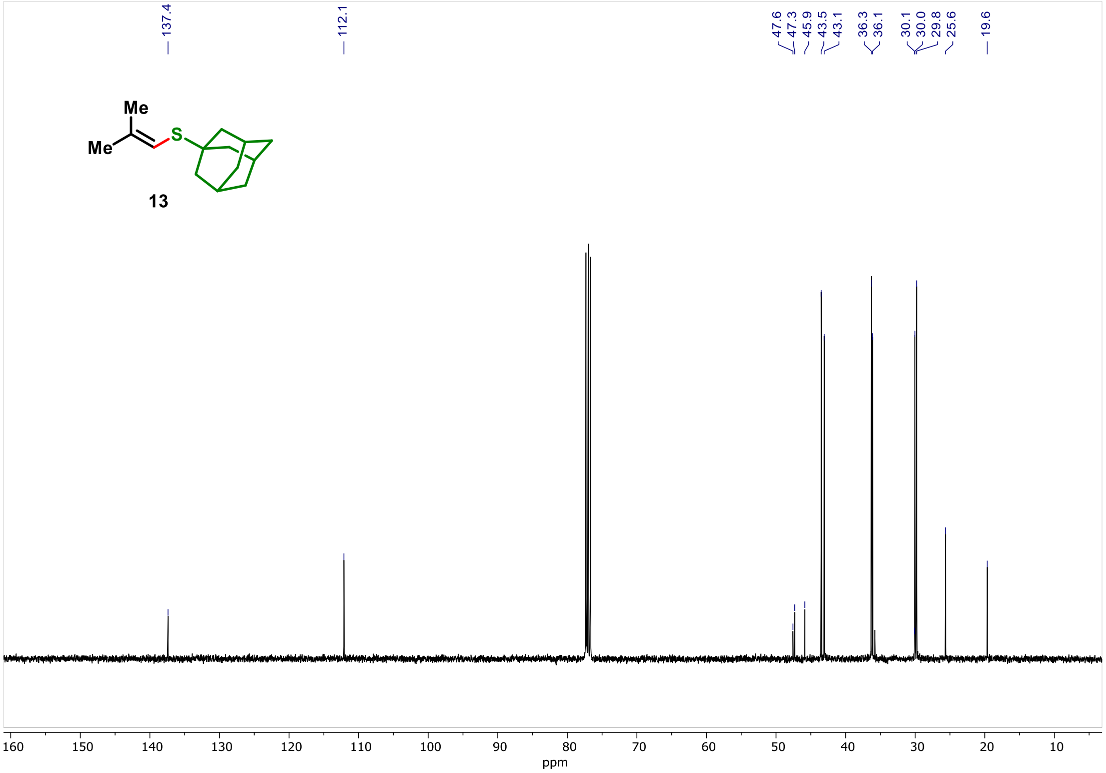

$^{1}$H NMR (400 MHz, $CDCl_3$) spectra of compound **14**

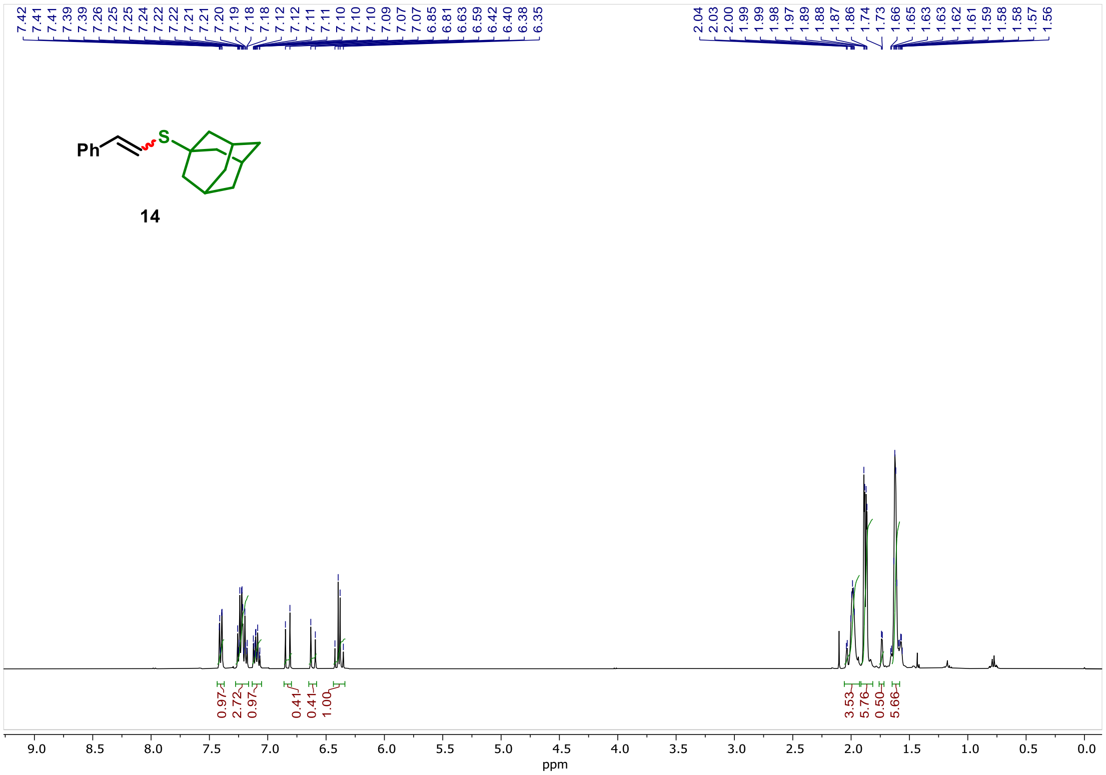


$^{13}$C NMR (101 MHz, $CDCl_3$) spectra of compound **14**

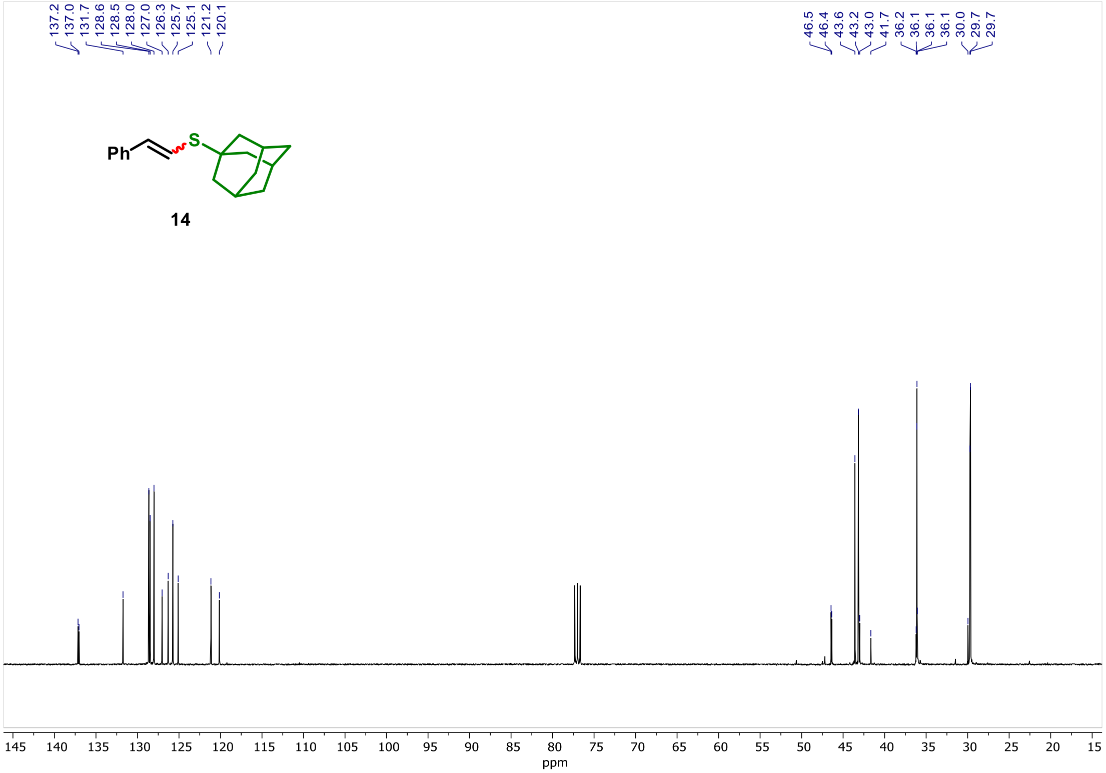

$^{1}$H NMR (400 MHz, $CDCl_3$) spectra of compound **15**

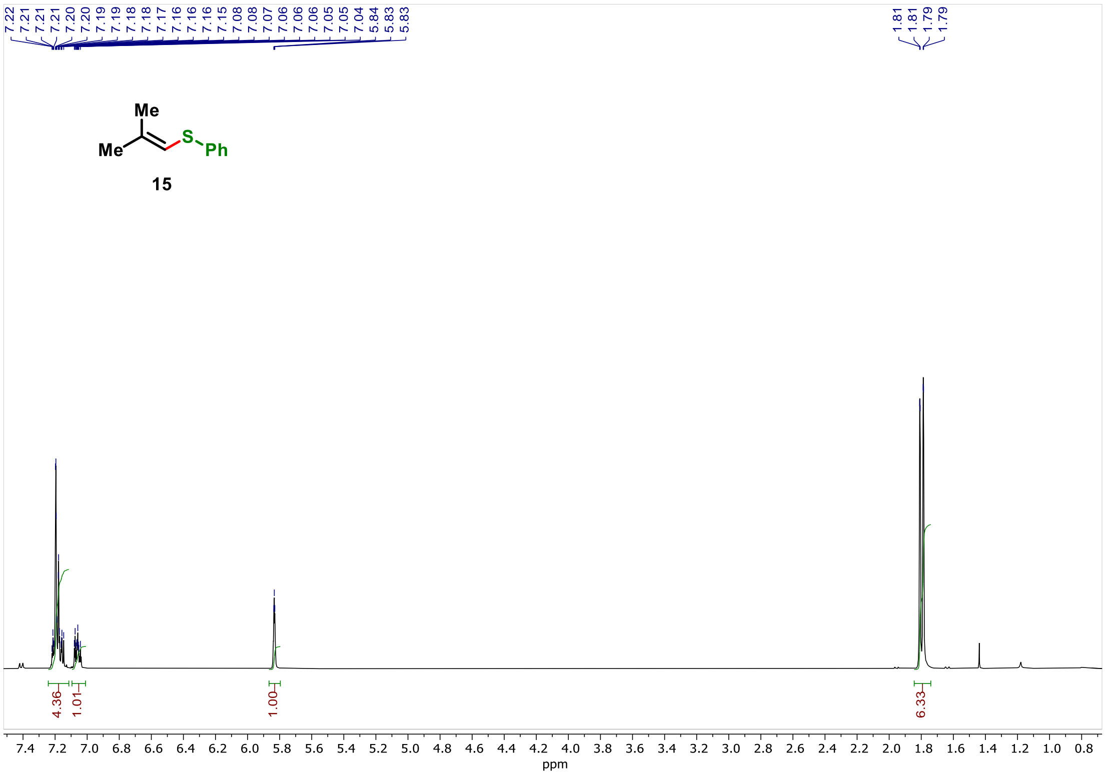


$^{13}$C NMR (101 MHz, $CDCl_3$) spectra of compound **15**

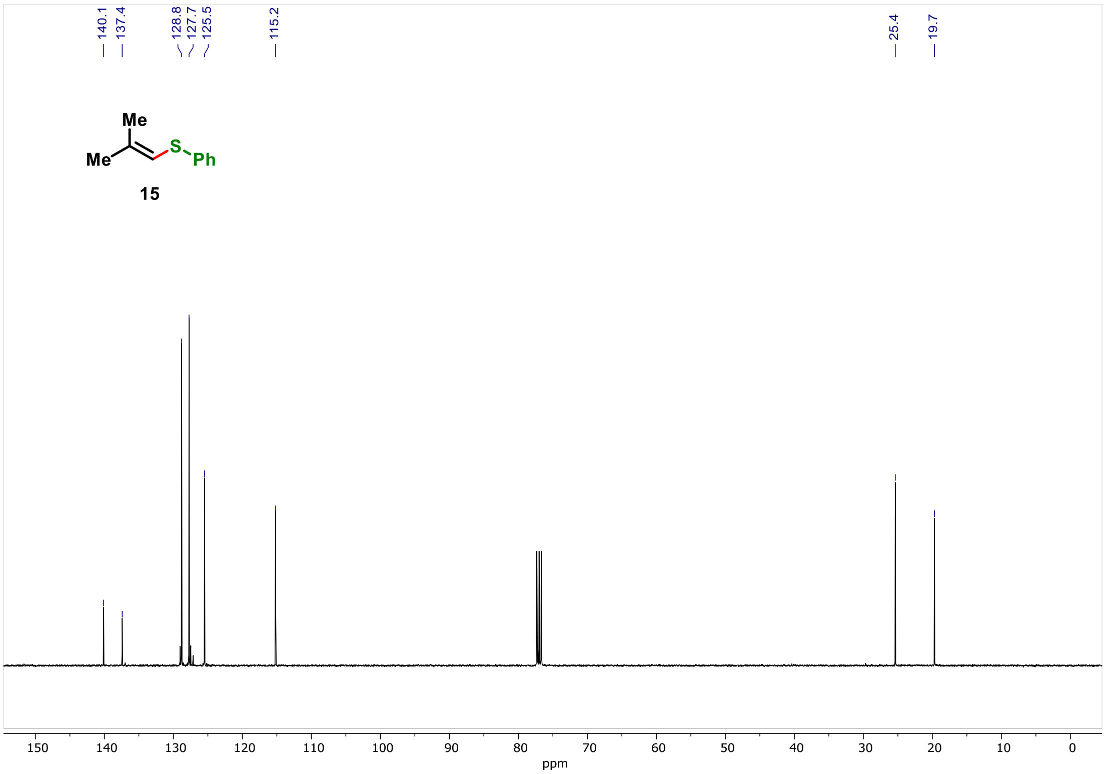

$^{1}$H NMR (400 MHz, $CDCl_3$) spectra of compound **16**

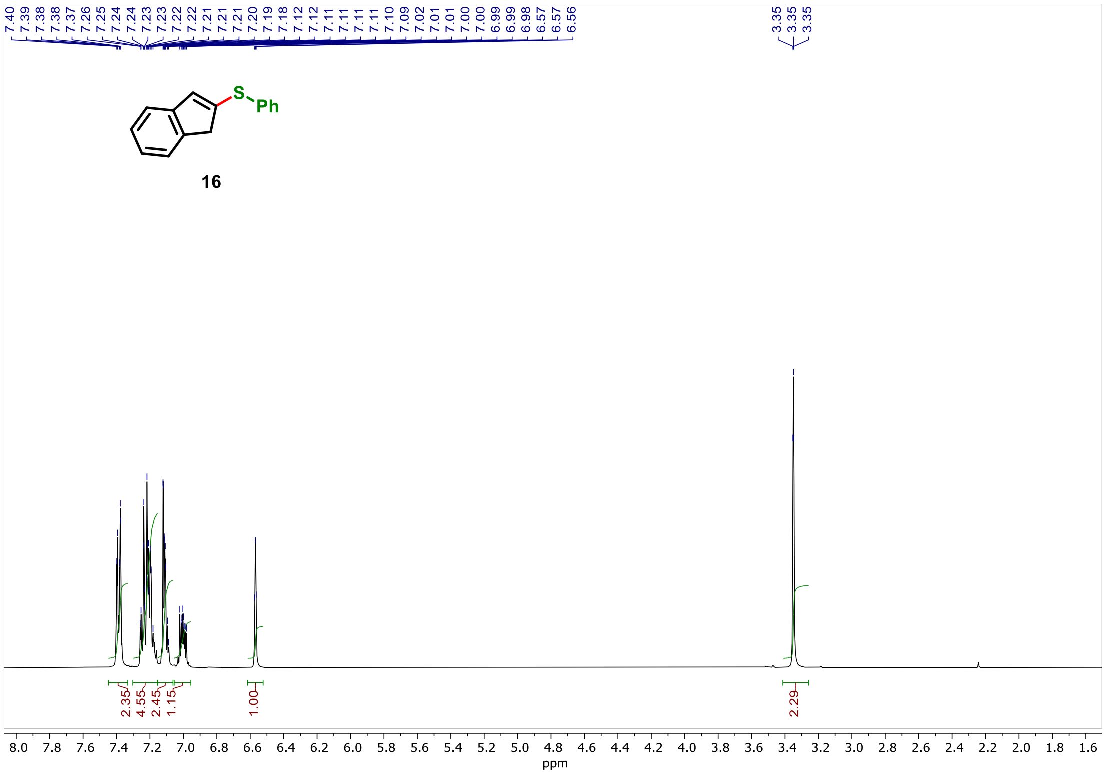


$^{13}$C NMR (101 MHz, $CDCl_3$) spectra of compound **16**

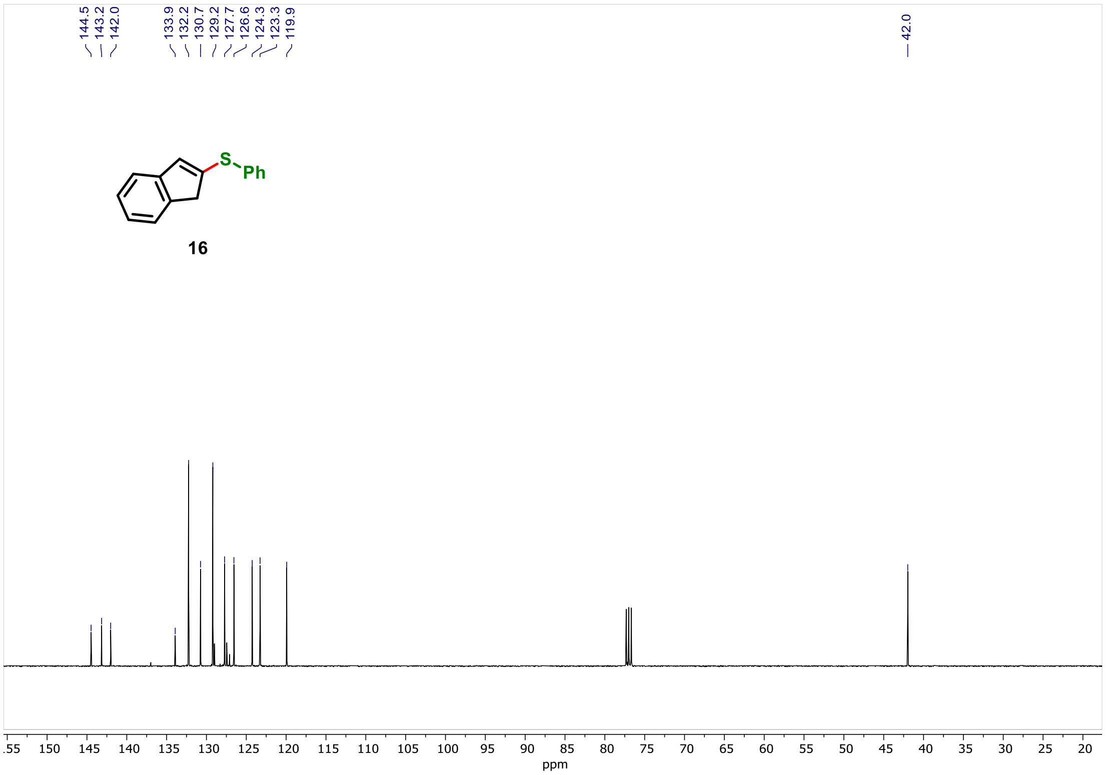

$^{1}H$ NMR (400 MHz, $CDCl_3$) spectra of compound **17**

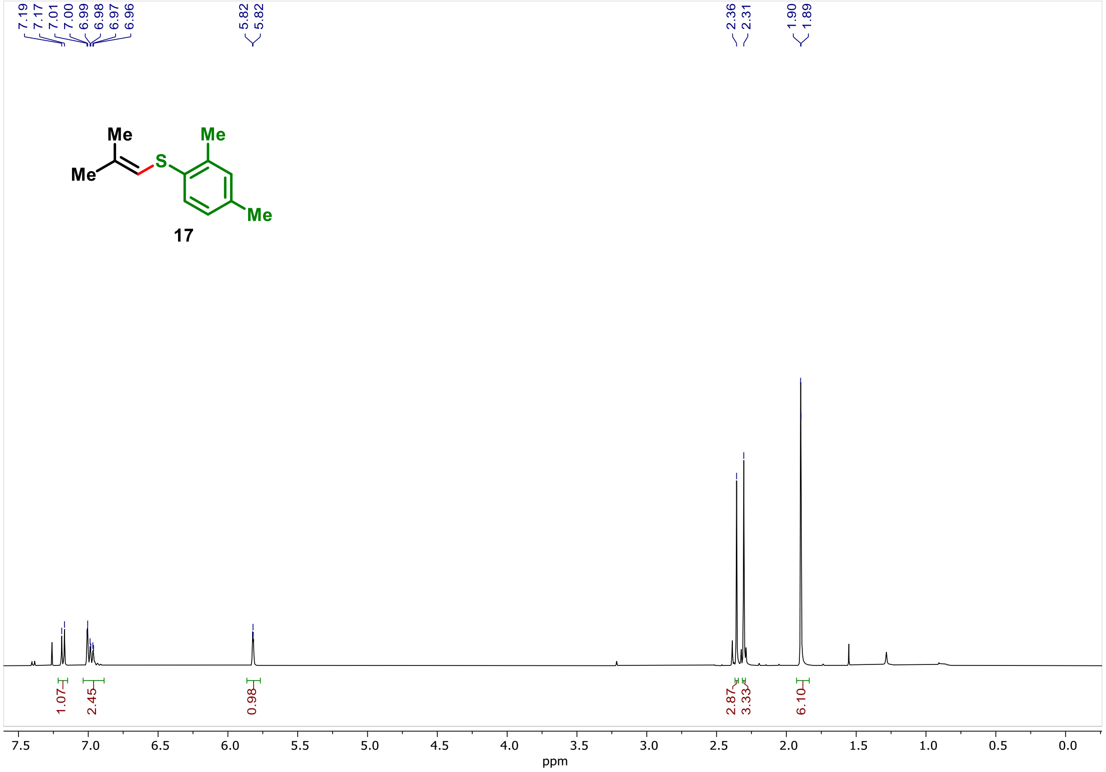


$^{13}C$ NMR (101 MHz, $CDCl_3$) spectra of compound **17**

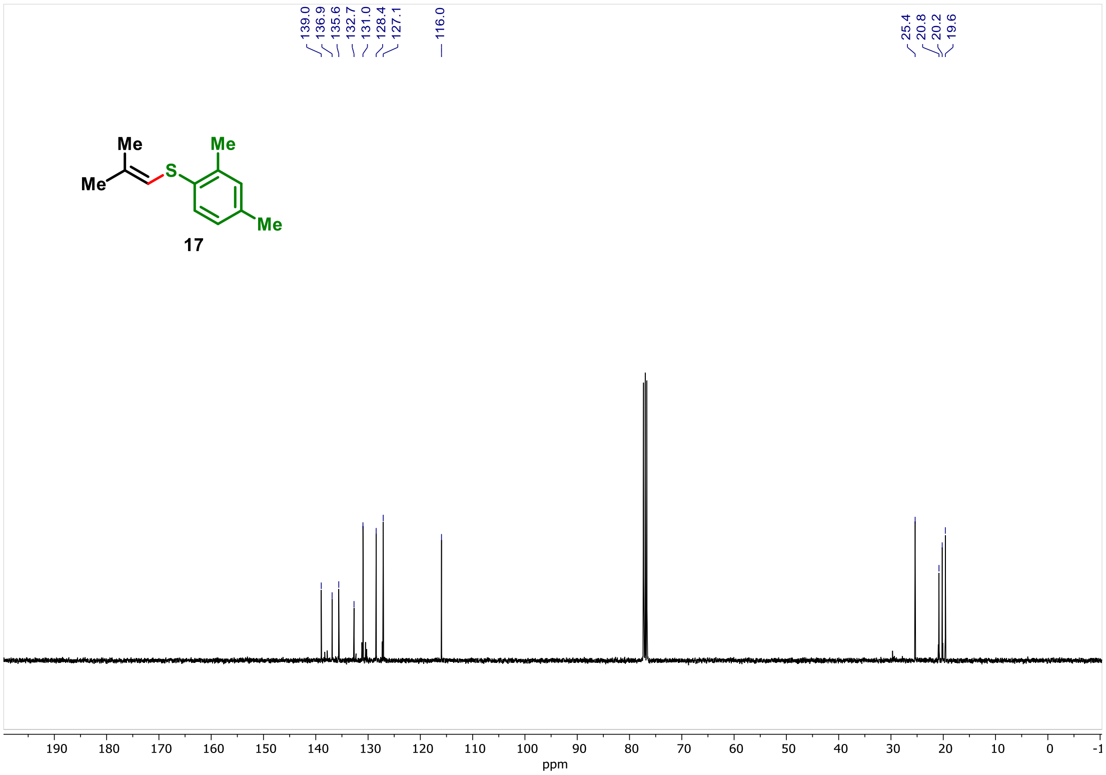

$^{1}H$ NMR (400 MHz, $CD_3CN$) spectra of compound **18**

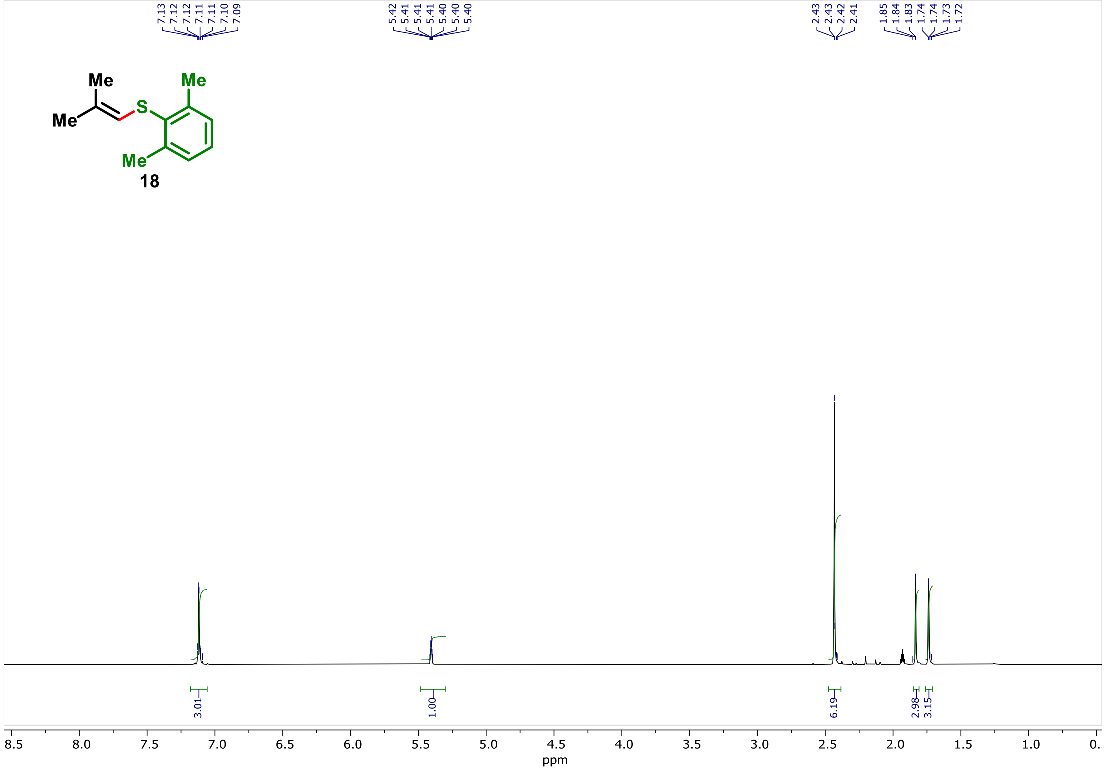


$^{13}C$ NMR (101 MHz, $CDCl_3$) spectra of compound **18**

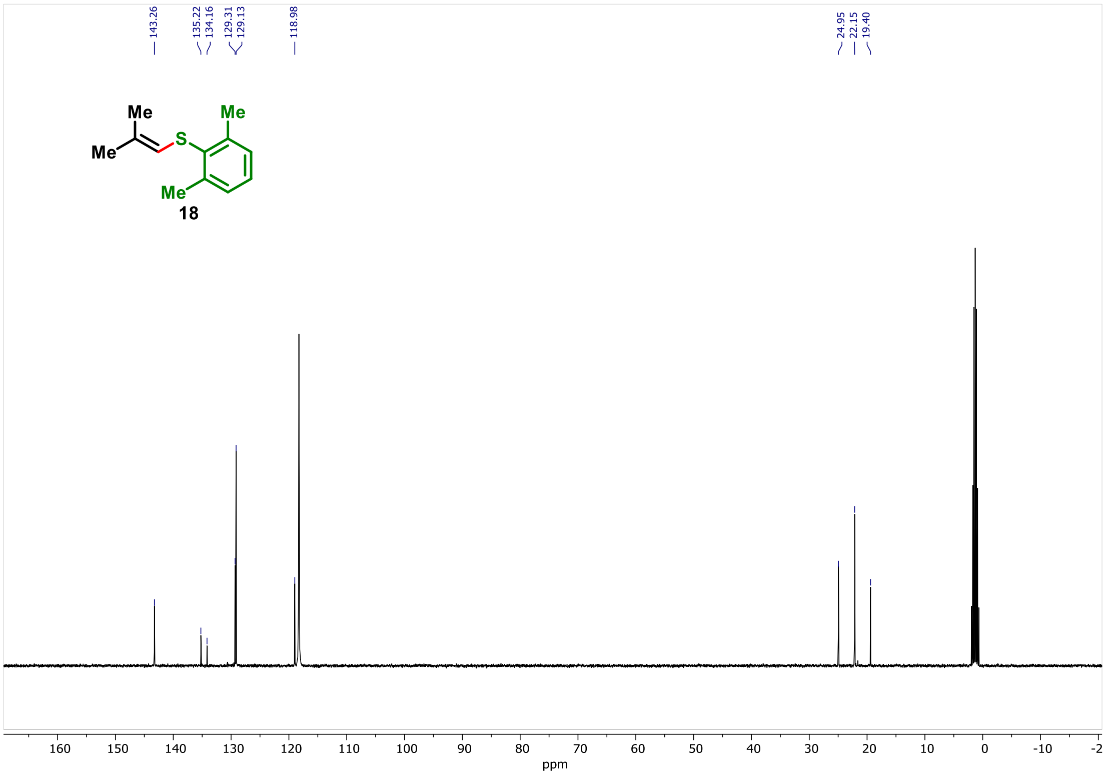

$^{1}$H NMR (400 MHz, $CDCl_3$) spectra of compound **19**

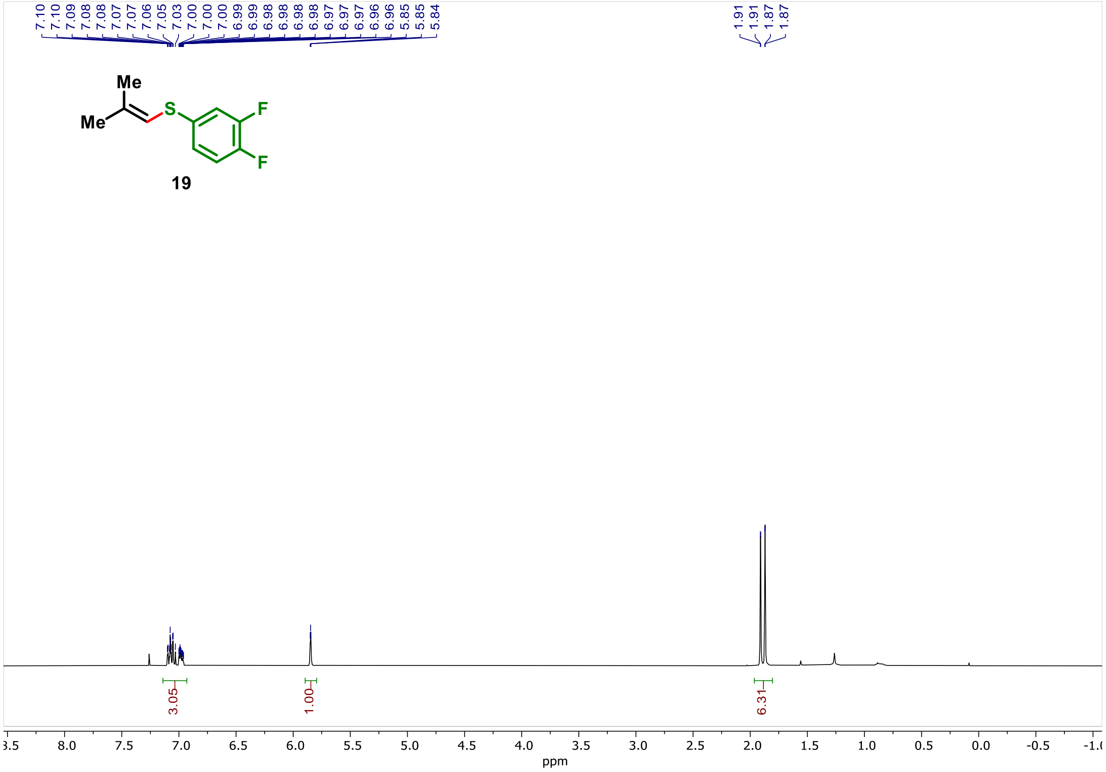


$^{13}$C NMR (101 MHz, $CDCl_3$) spectra of compound **19**

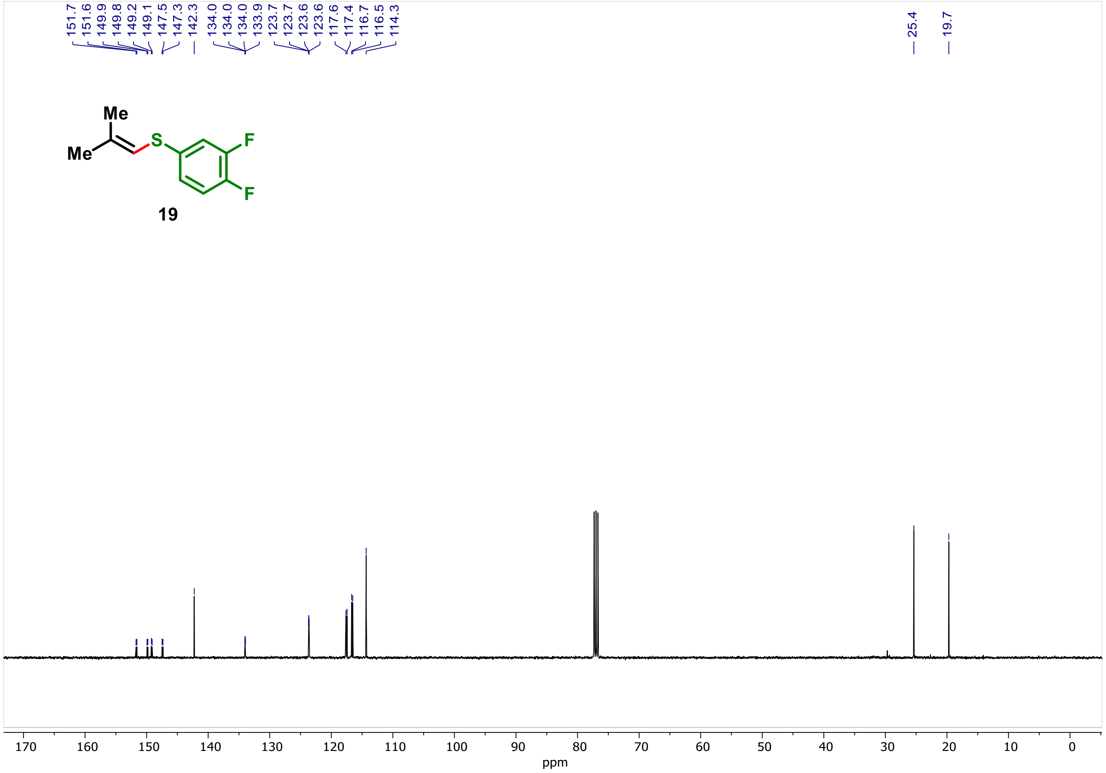

<sup></sup>$^{19}$F NMR (376 MHz, $CDCl_3$) spectra of compound **19**

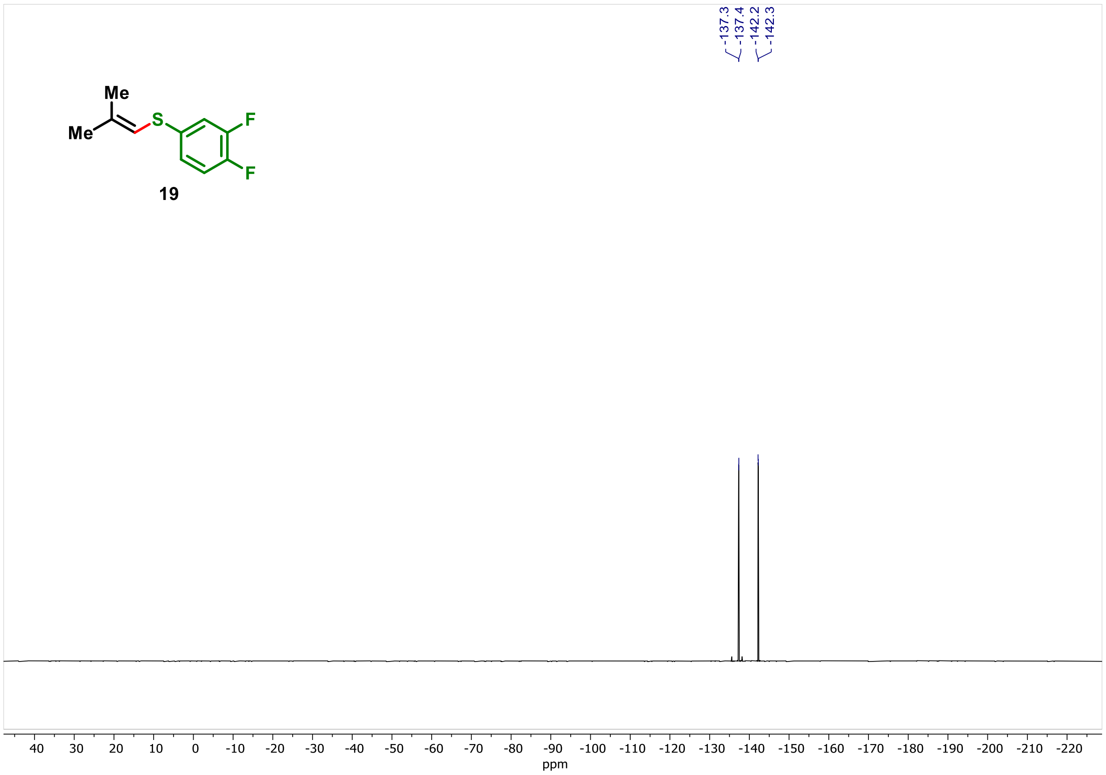

$^{1}$H NMR (400 MHz, $CDCl_3$) spectra of compound **20**

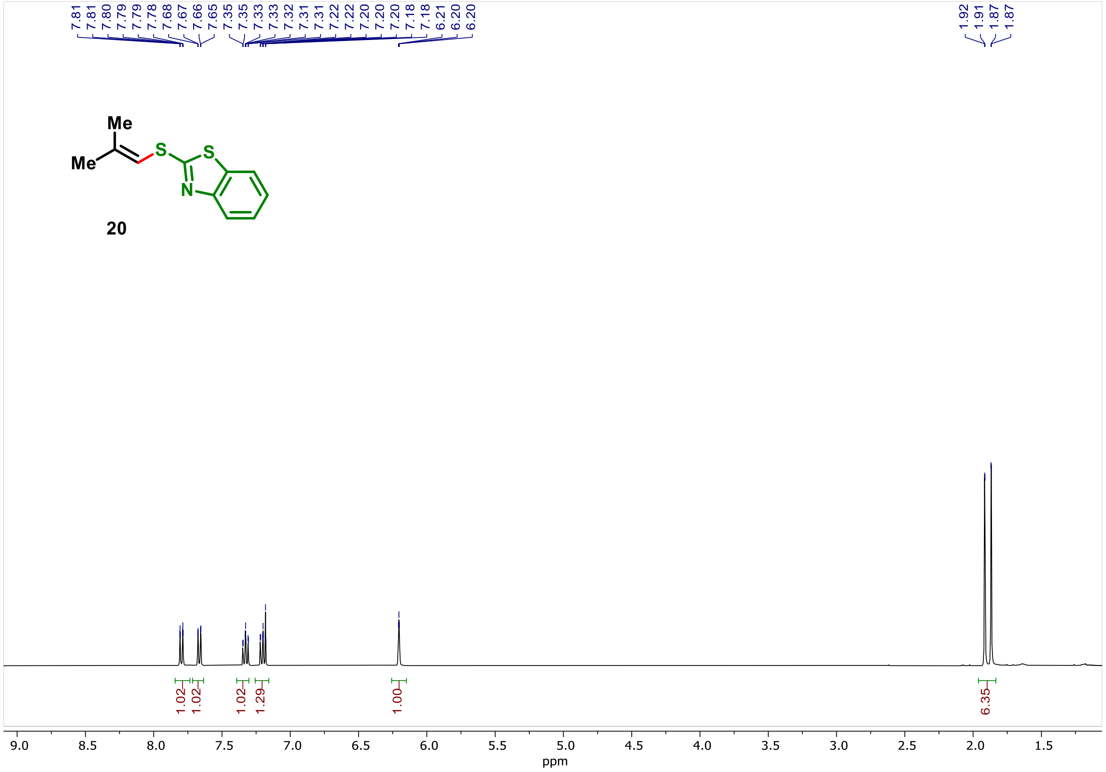

$^{13}$C NMR (101 MHz, $CDCl_3$) spectra of compound **20**

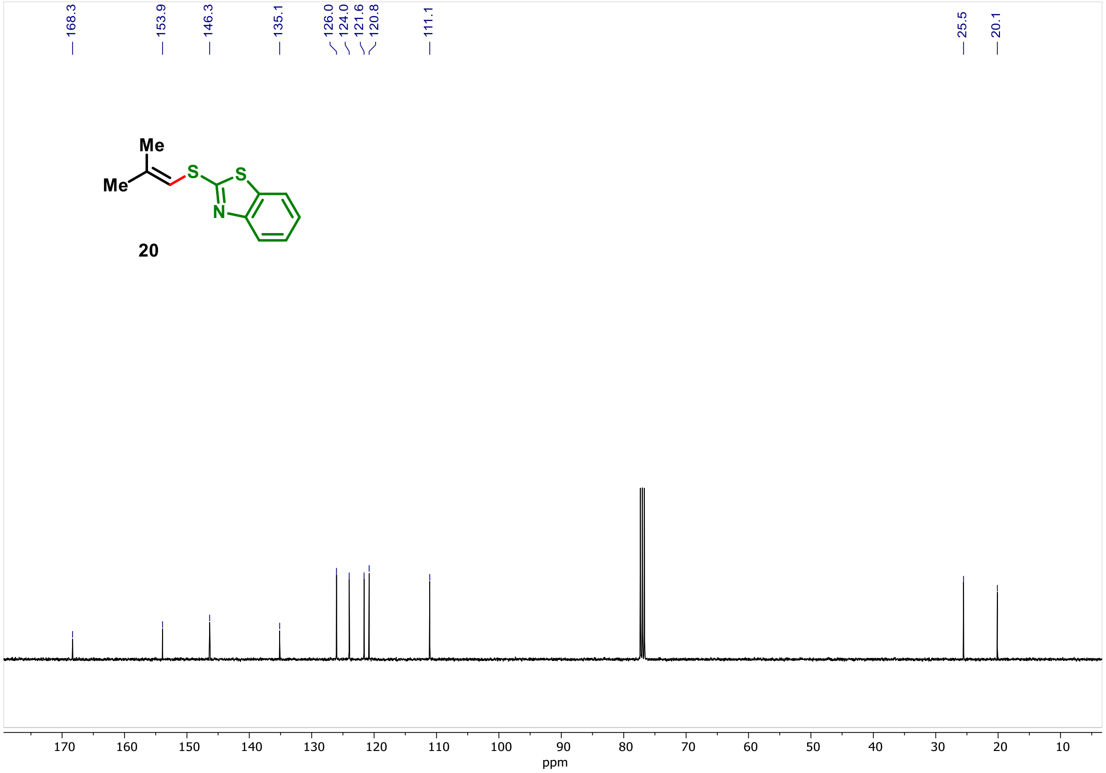

$^{1}$H NMR (400 MHz, $CDCl_3$) spectra of compound **22**

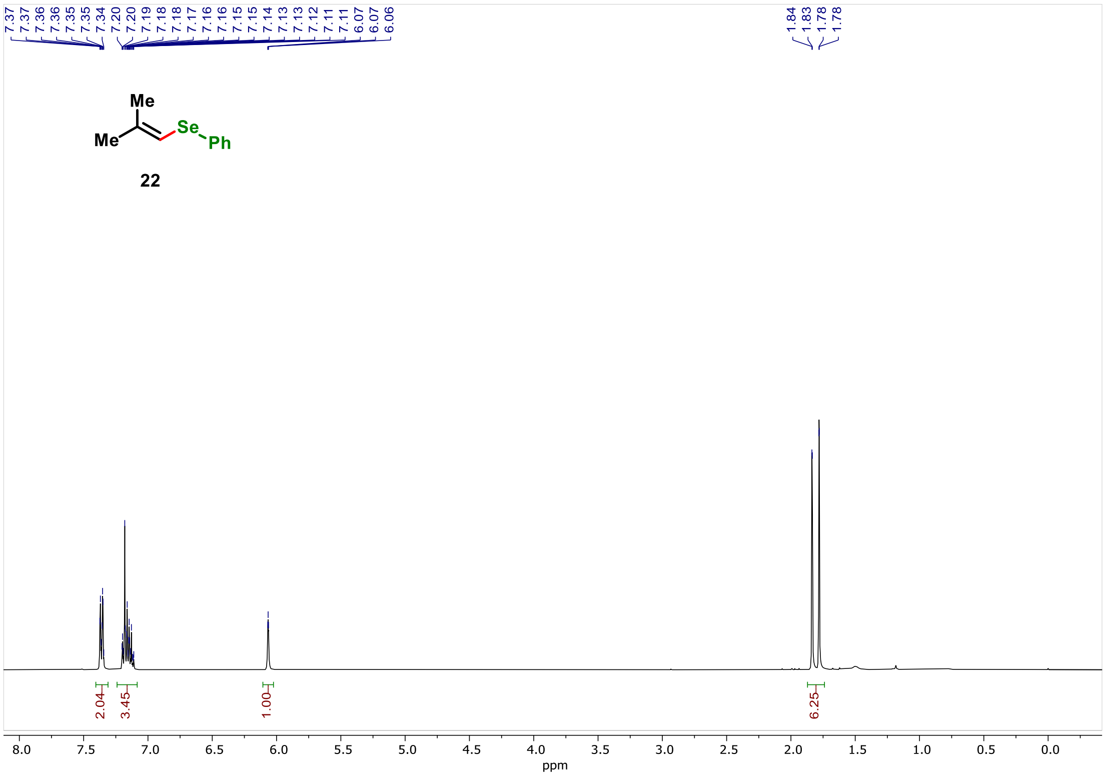


$^{13}$C NMR (101 MHz, $CDCl_3$) spectra of compound **22**

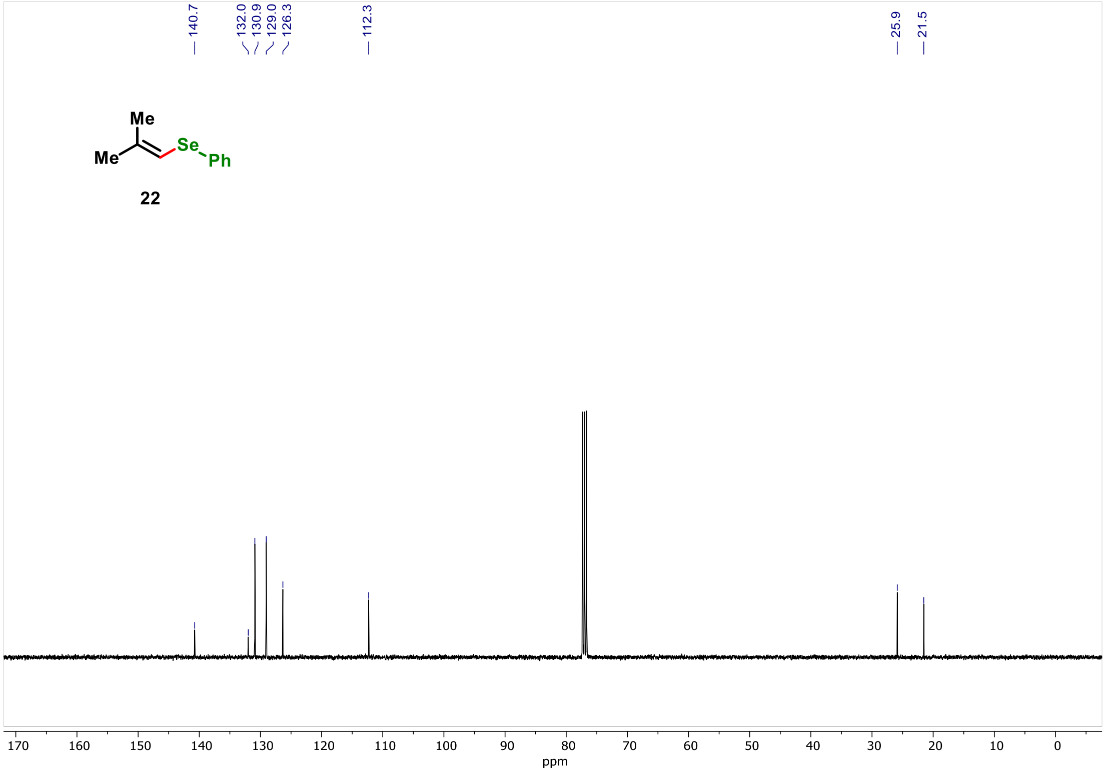

$^{1}H$ NMR (400 MHz, $CD_3CN$) spectra of compound **23**

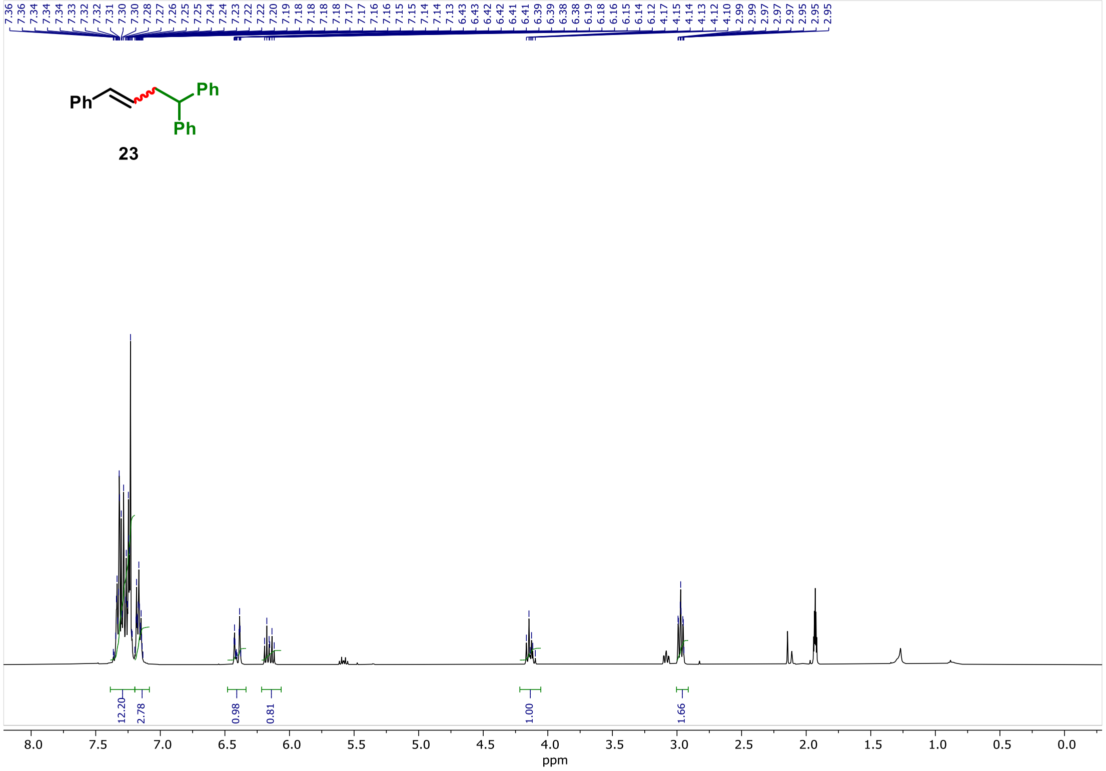


$^{13}C$ NMR (101 MHz, $CD_3CN$) spectra of compound **23**

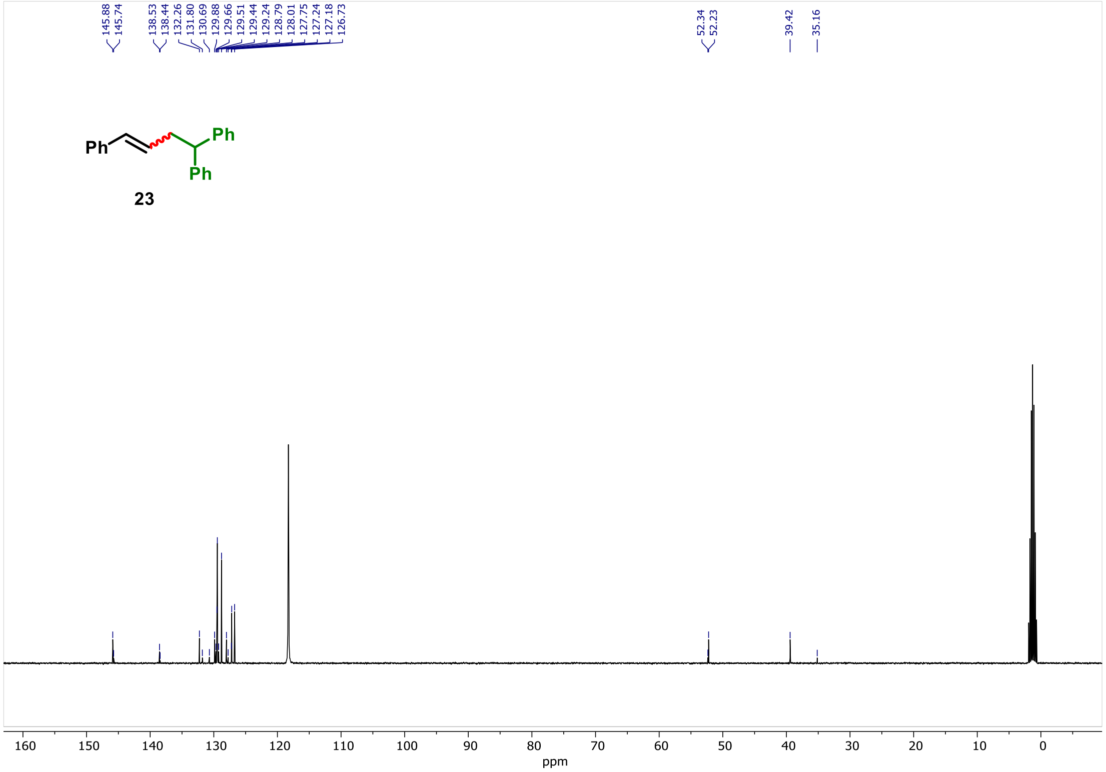

$^{1}H$ NMR (400 MHz, $CDCl_3$) spectra of compound **27**

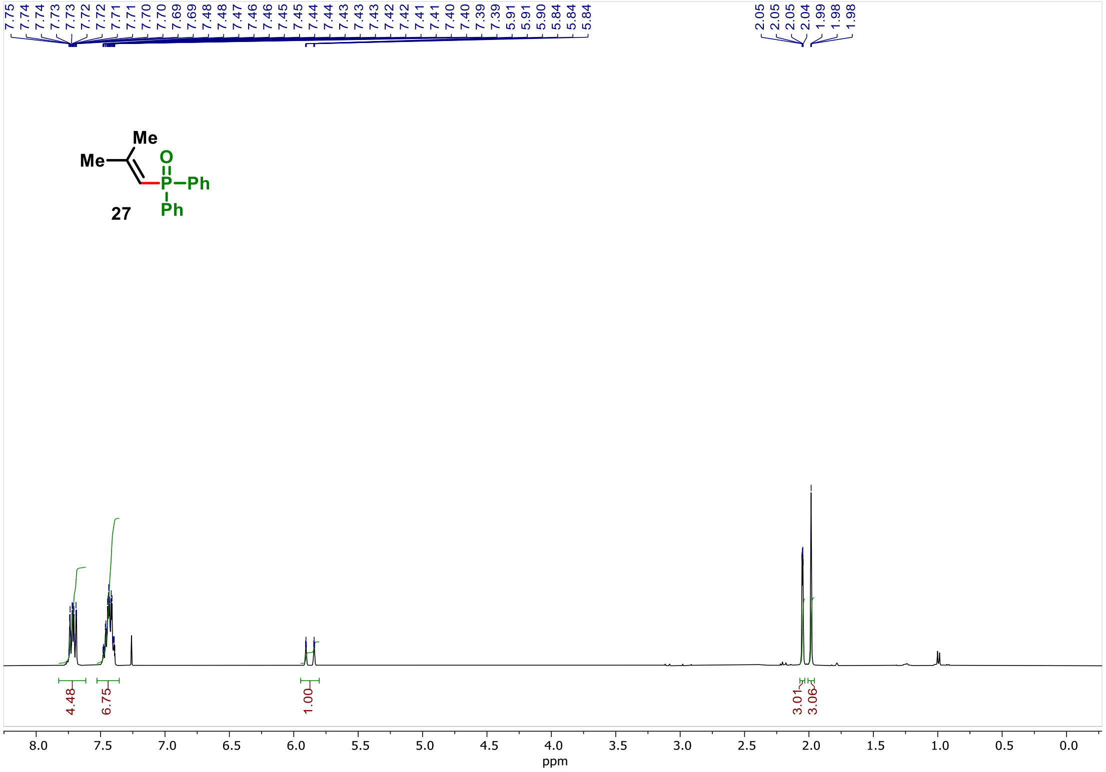

$^{13}C$ NMR (101 MHz, $CDCl_3$) spectra of compound **27**

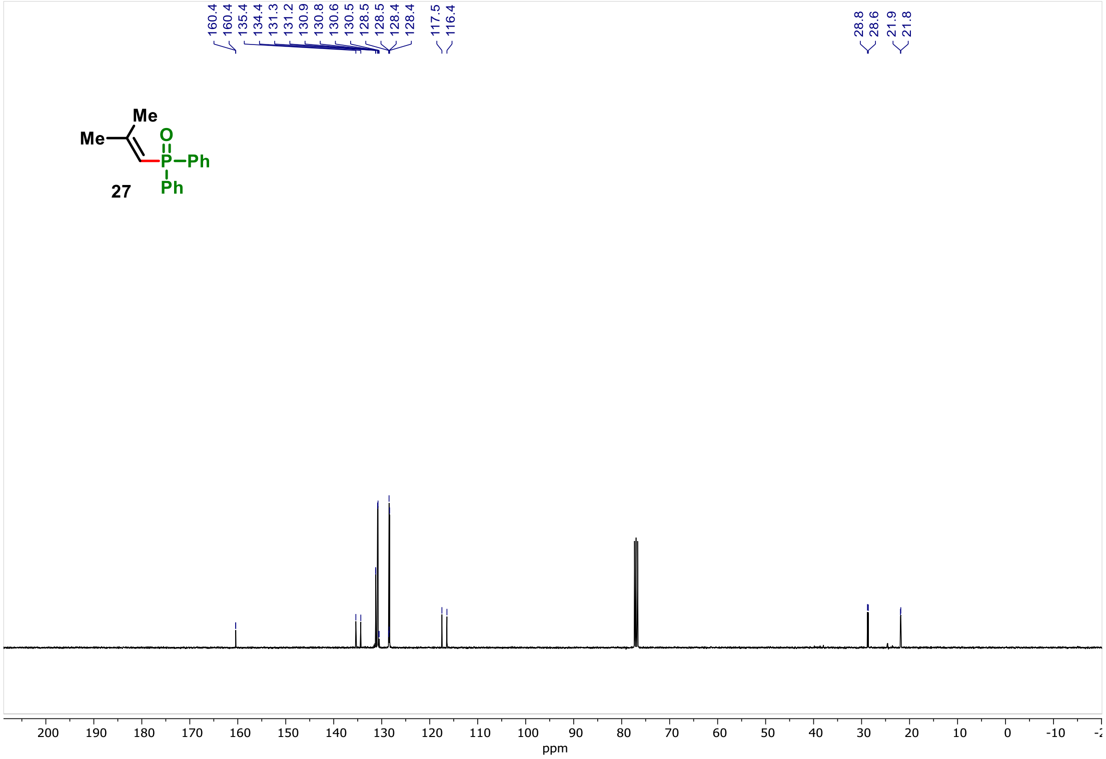

$^{31}$P NMR (162 MHz, $CDCl_3$) spectra of compound **27**

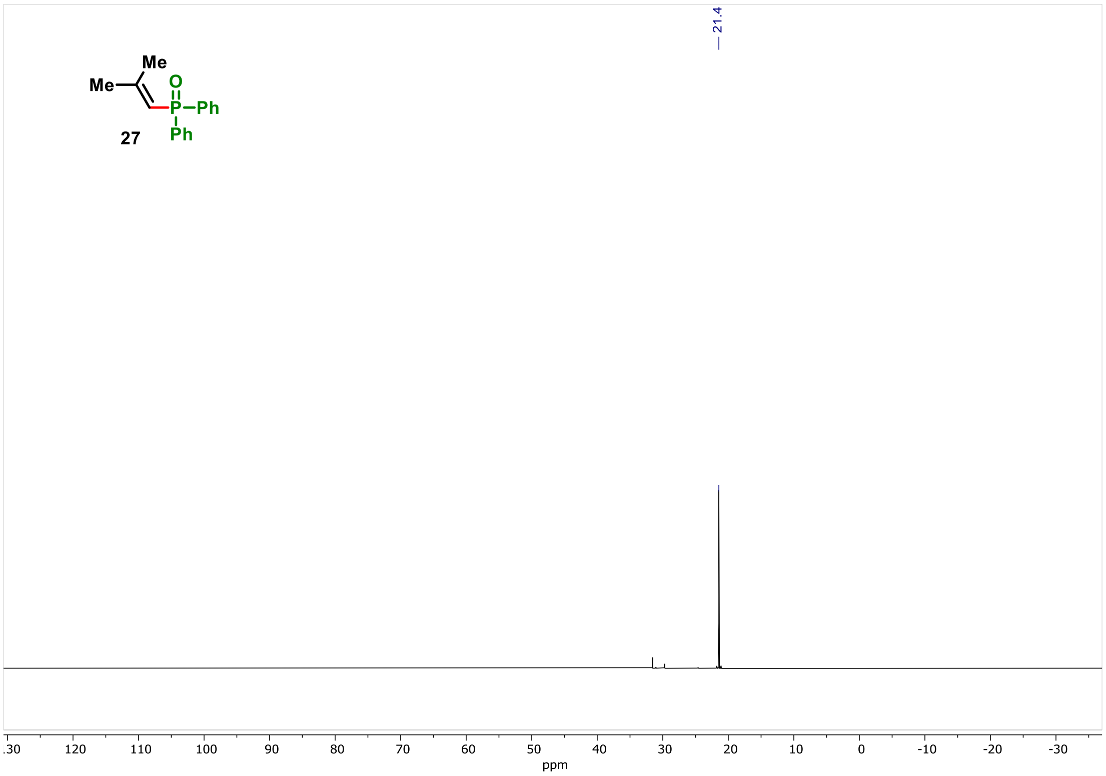

$^{1}H$ NMR (400 MHz, $CD_3CN$) spectra of compound **28**

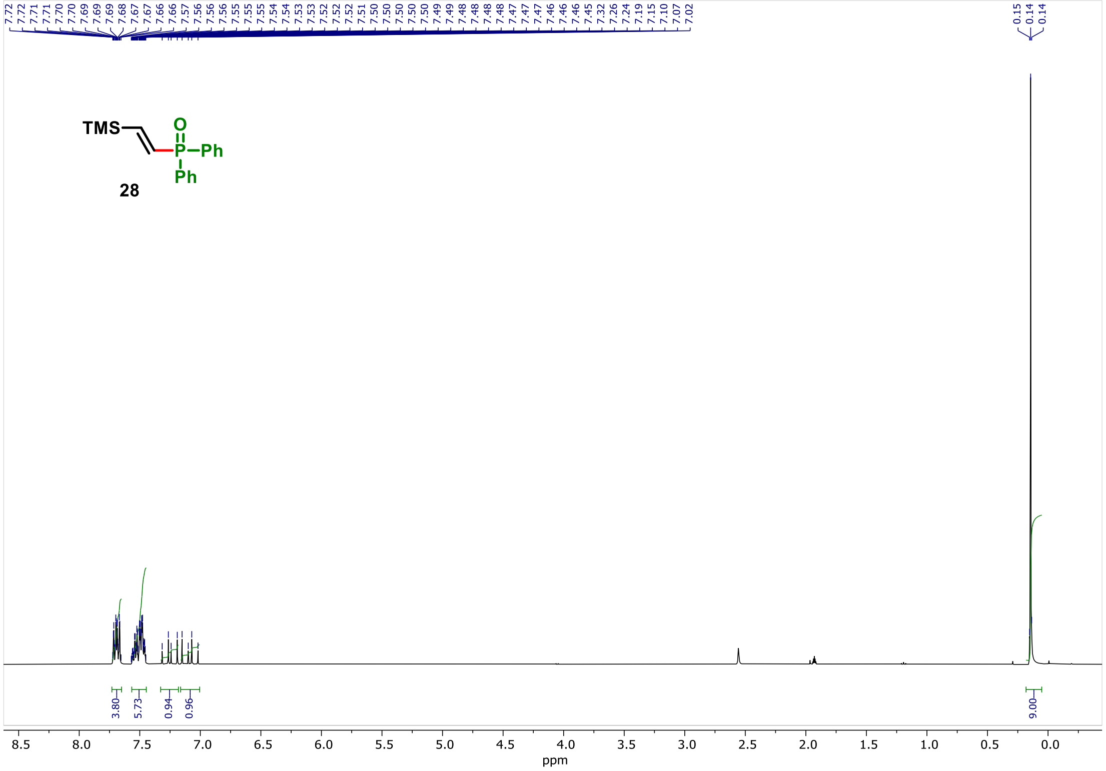


$^{13}C$ NMR (101 MHz, $CD_3CN$) spectra of compound **28**

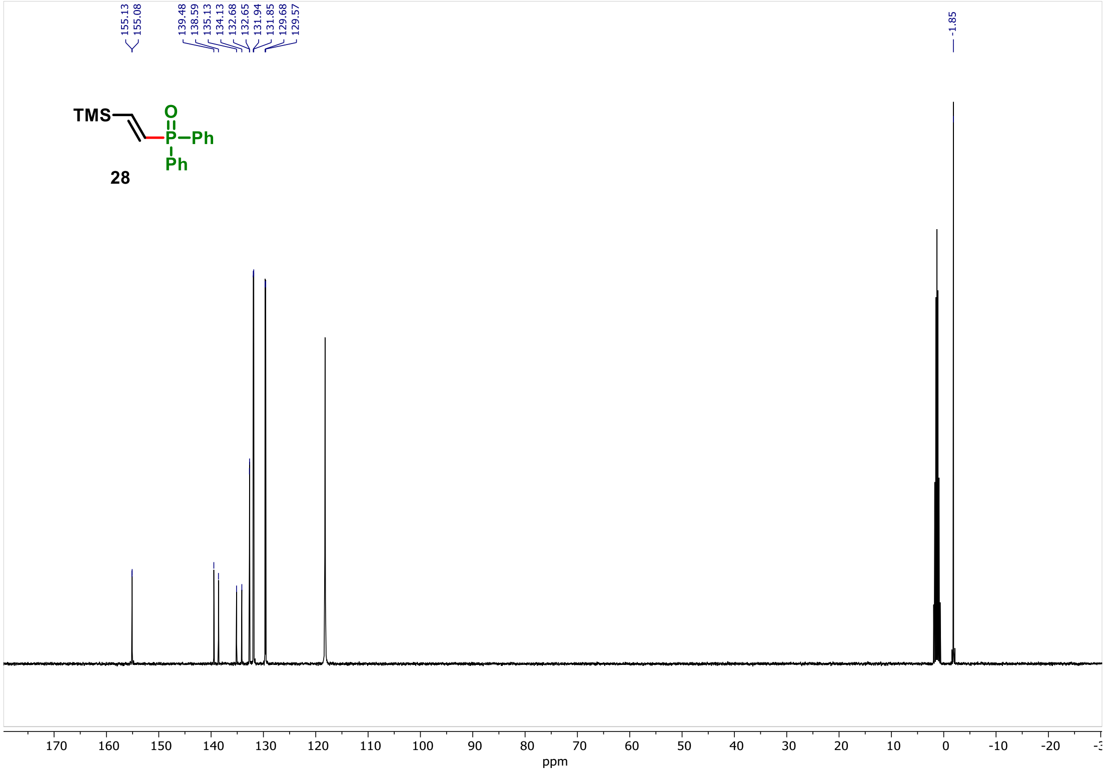

$^{31}$P NMR (162 MHz, $CD_3CN$) spectra of compound **28**

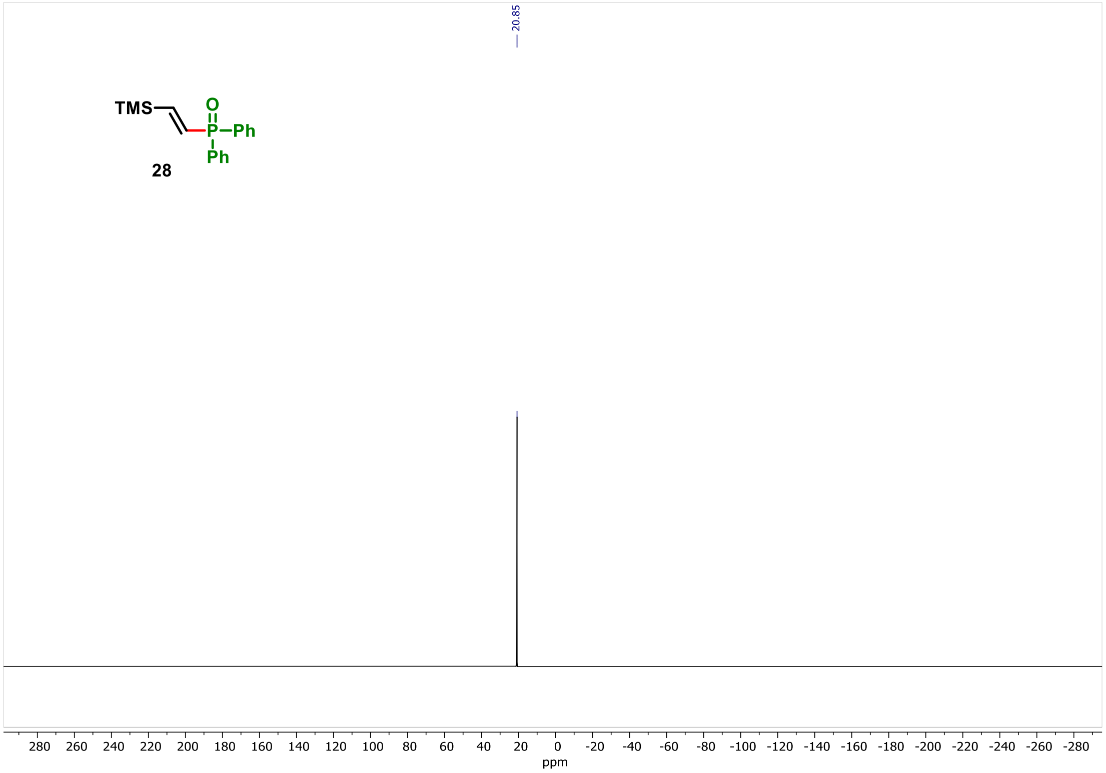

<sup></sup>

$^{1}$H NMR (400 MHz, $CDCl_3$) spectra of compound **30**

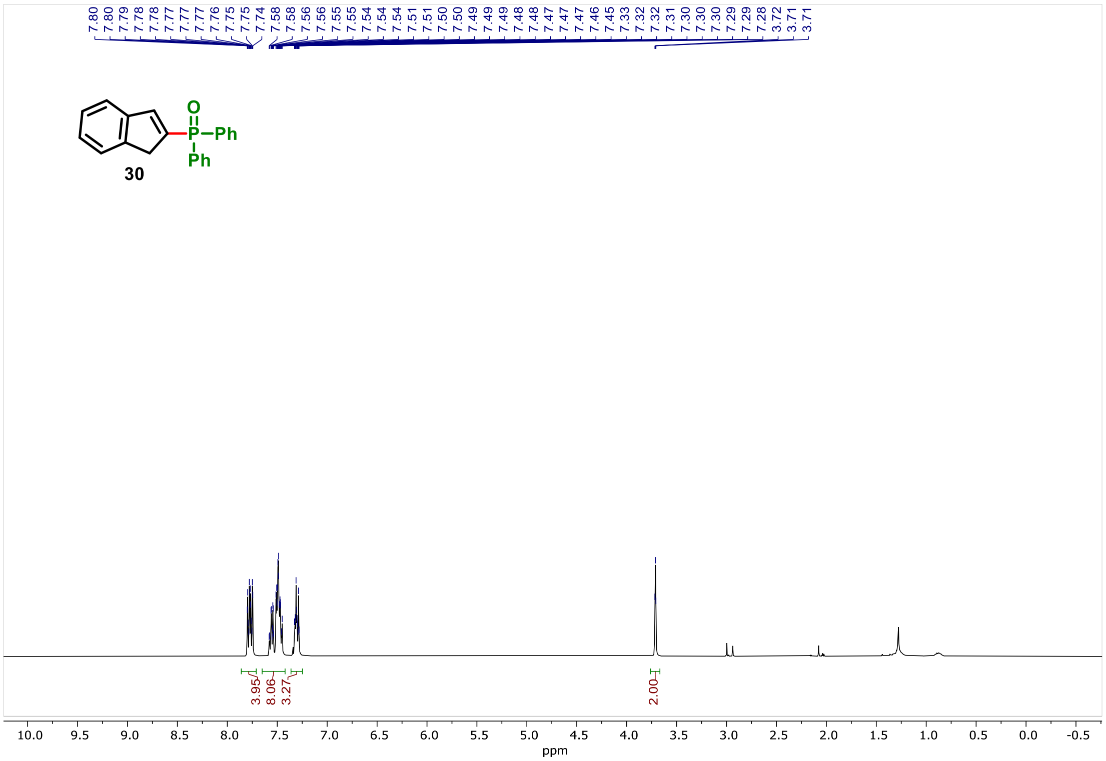

$^{13}$C NMR (101 MHz, $CDCl_3$) spectra of compound **30**

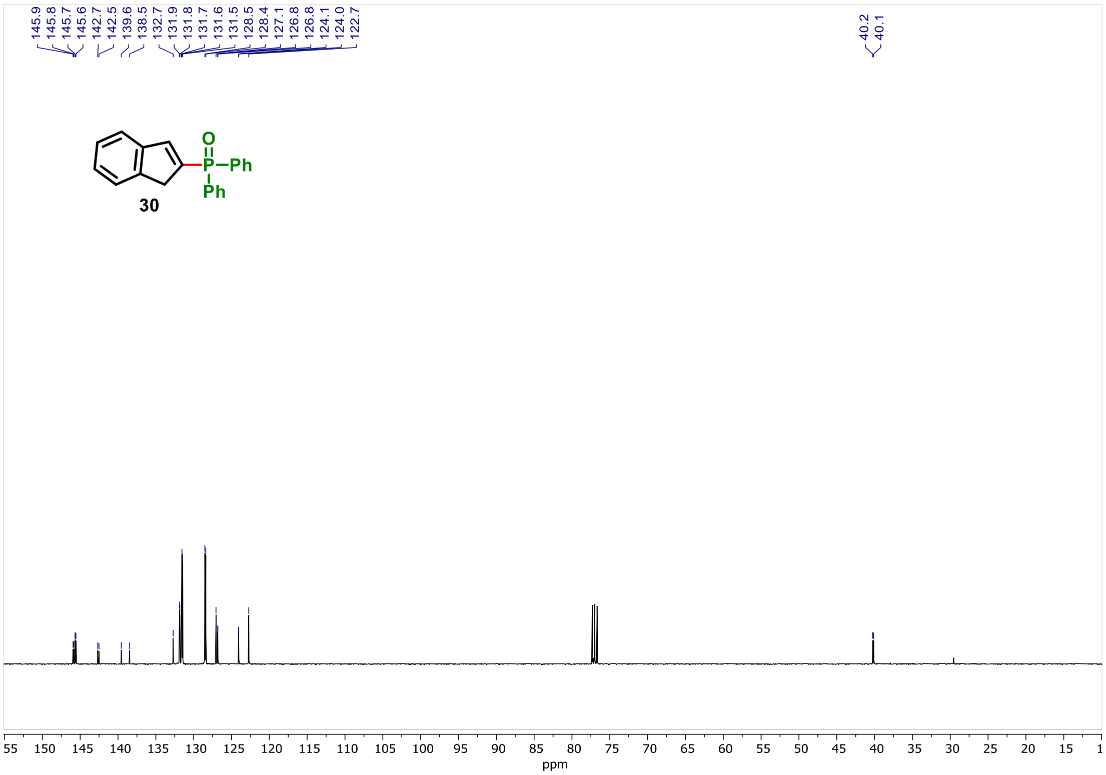

$^{31}$P NMR (162 MHz, $CDCl_3$) spectra of compound **30**

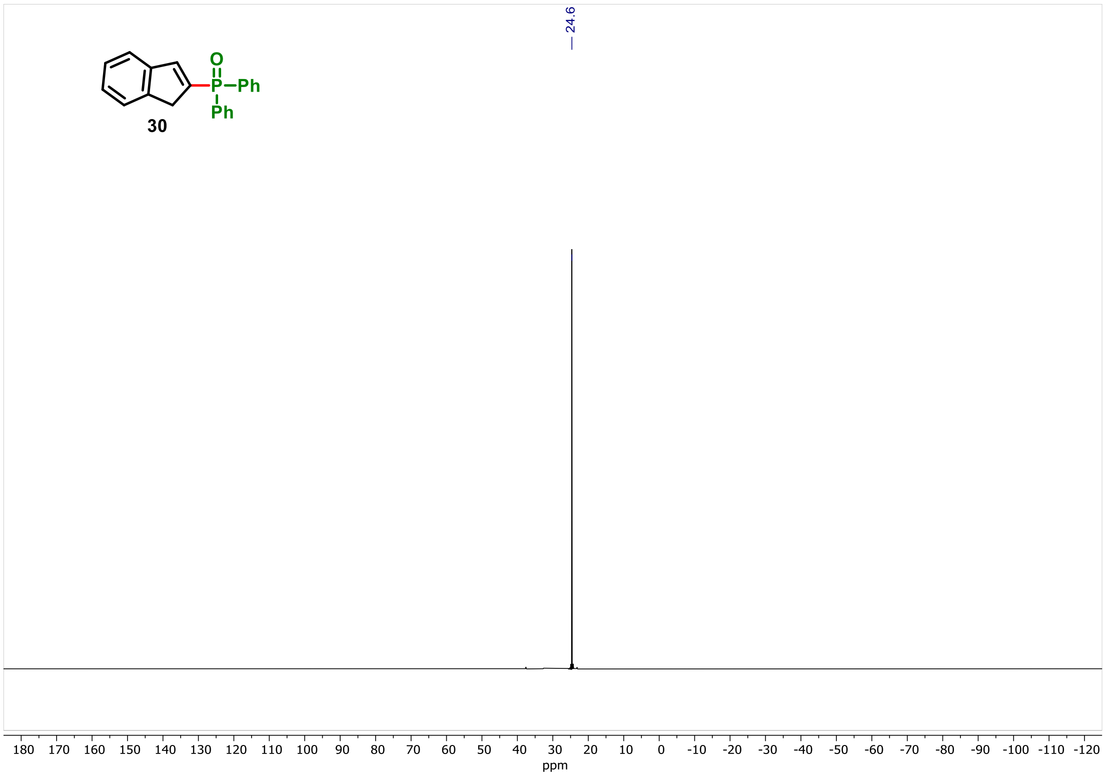

$^{1}H$ NMR (400 MHz, $CD_3CN$) spectra of compound **32**

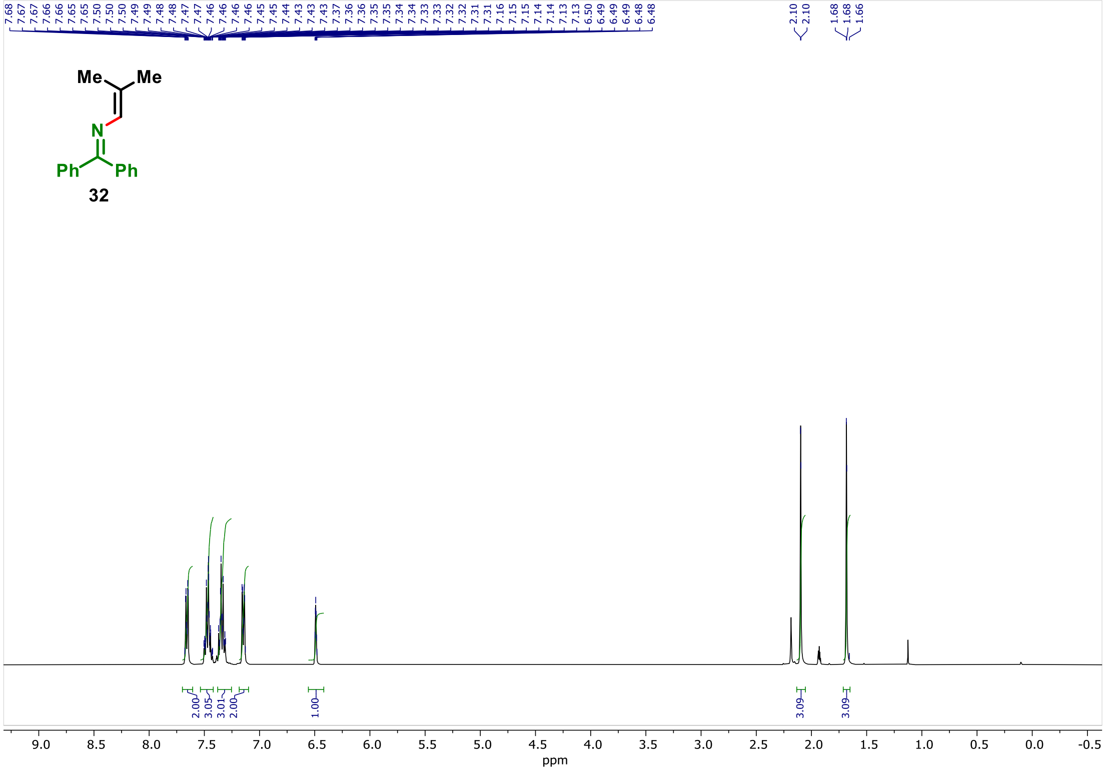

$^{13}C$ NMR (101 MHz, $CD_3CN$) spectra of compound **32**

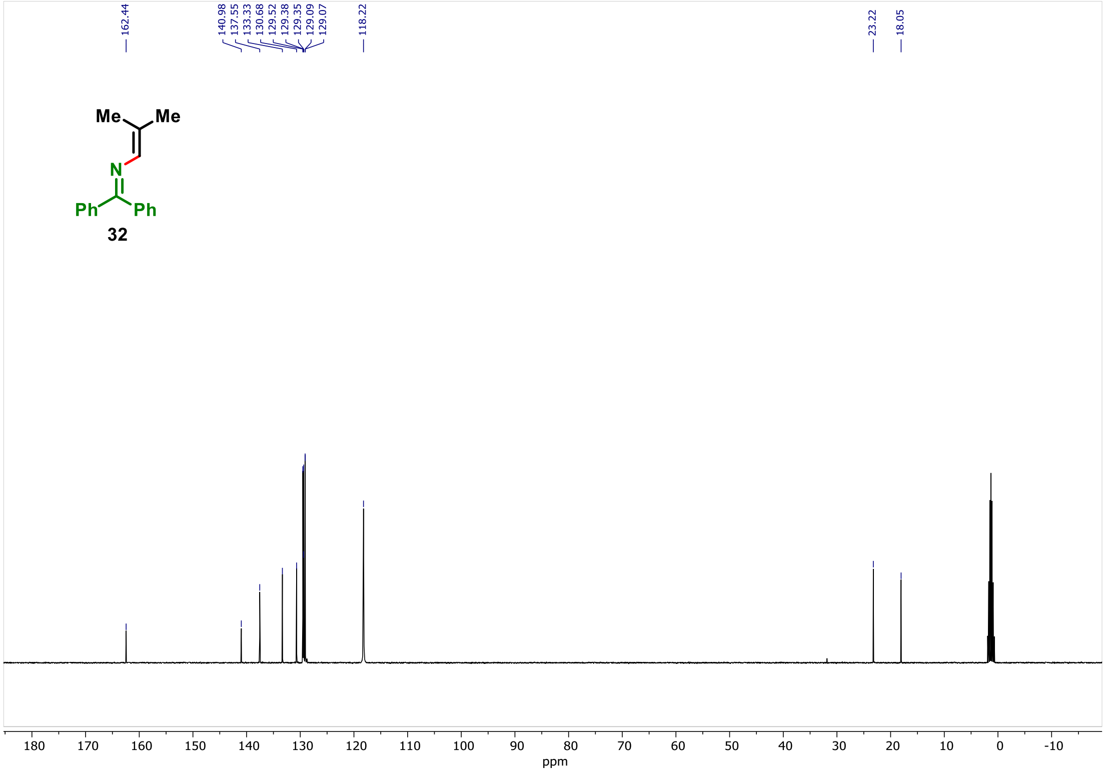

$^{1}H$ NMR (400 MHz, $CD_3CN$) spectra of compound **33**

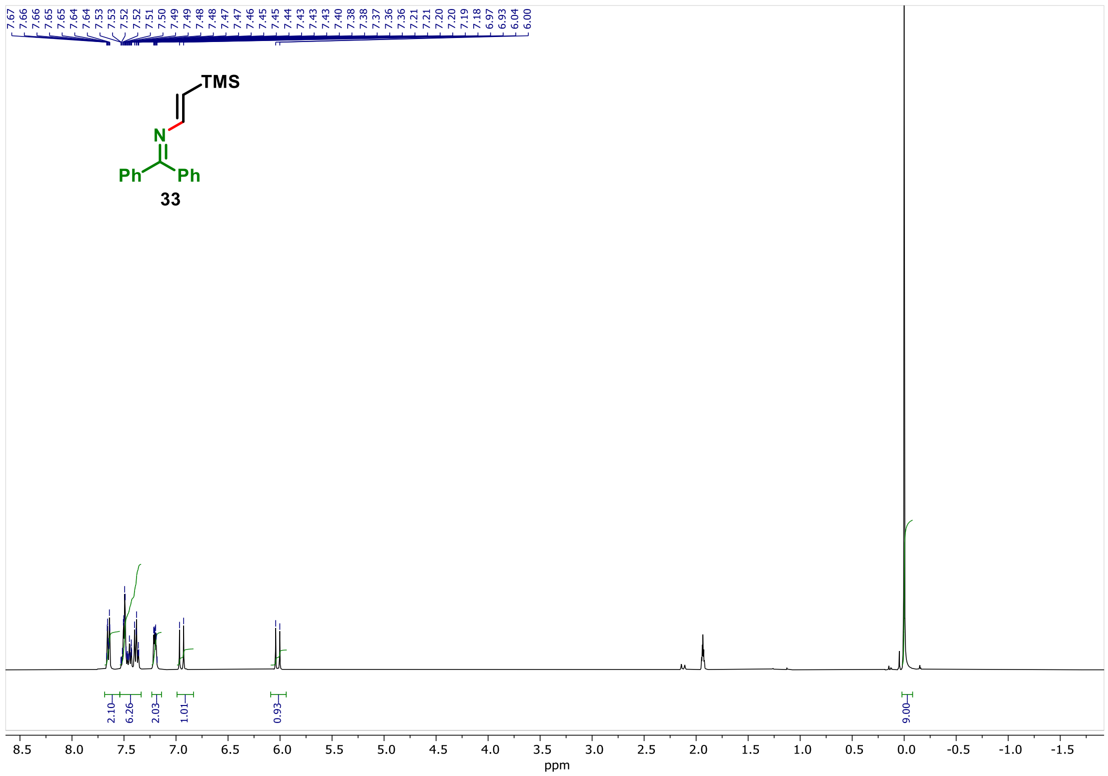

$^{13}C$ NMR (101 MHz, $CD_3CN$) spectra of compound **33**

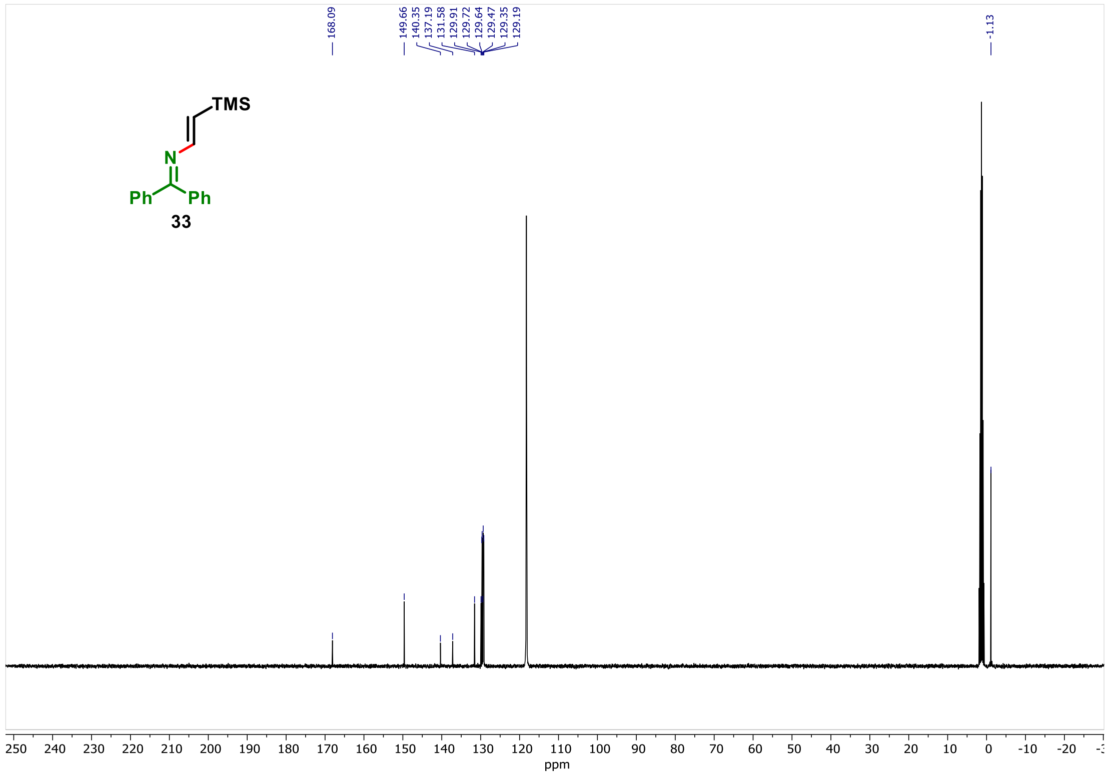

$^{1}$H NMR (400 MHz, $CD_3CN$) spectra of compound **34**

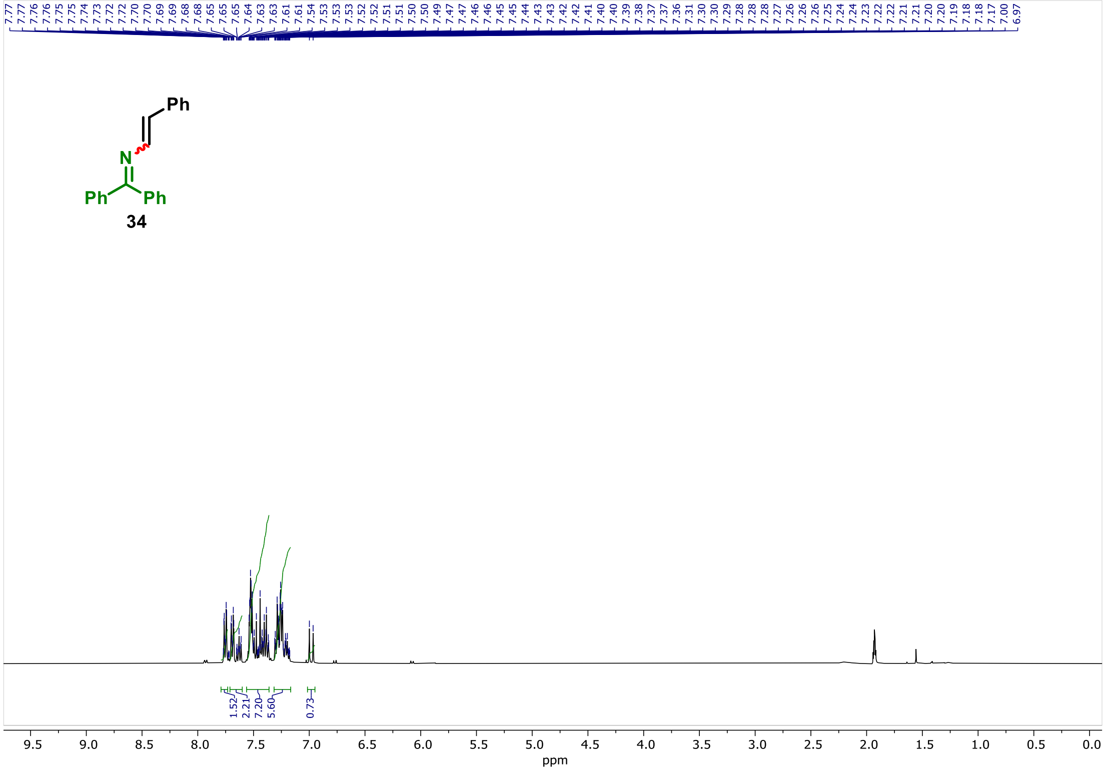


$^{13}$C NMR (101 MHz, $CD_3CN$) spectra of compound **34**

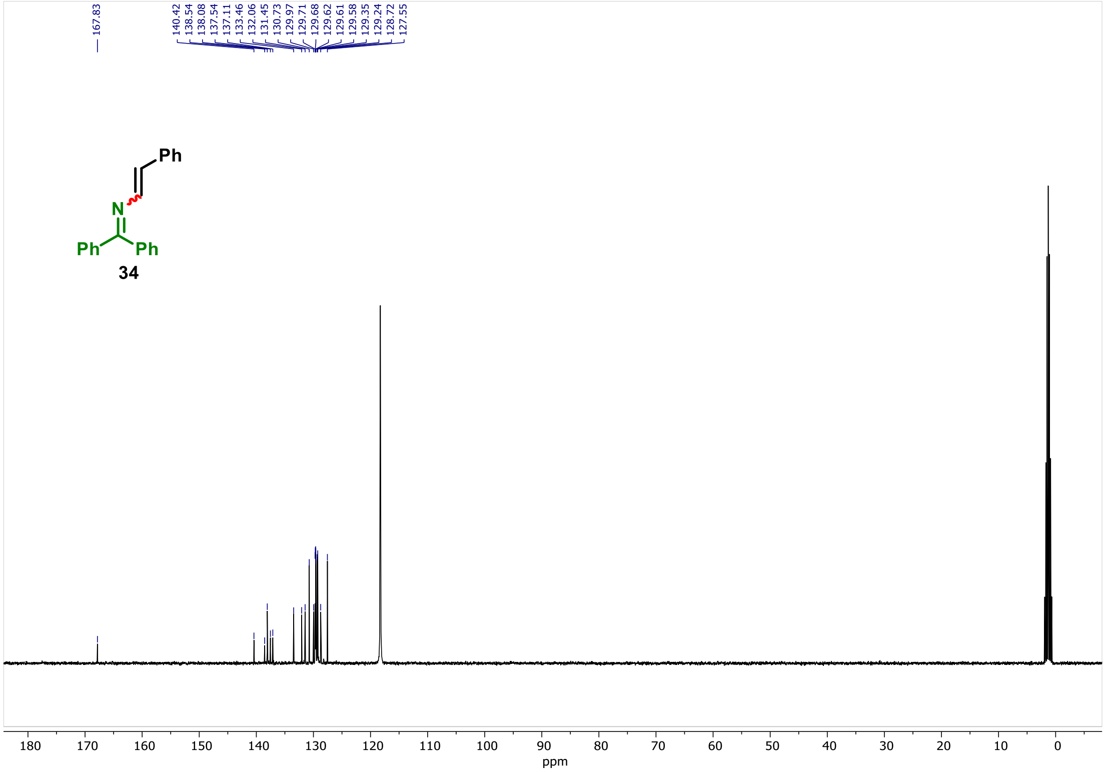

$^{1}$H NMR (400 MHz, $CD_3CN$) spectra of compound **35**

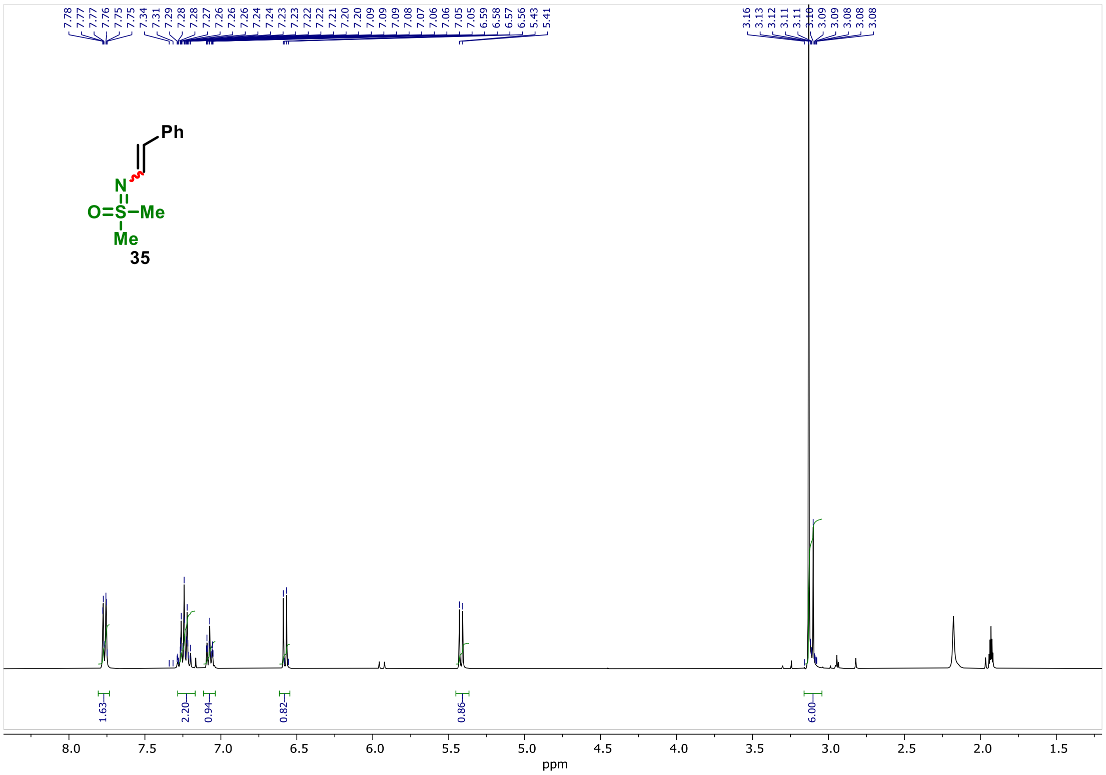


$^{13}$C NMR (101 MHz, $CD_3CN$) spectra of compound **35**

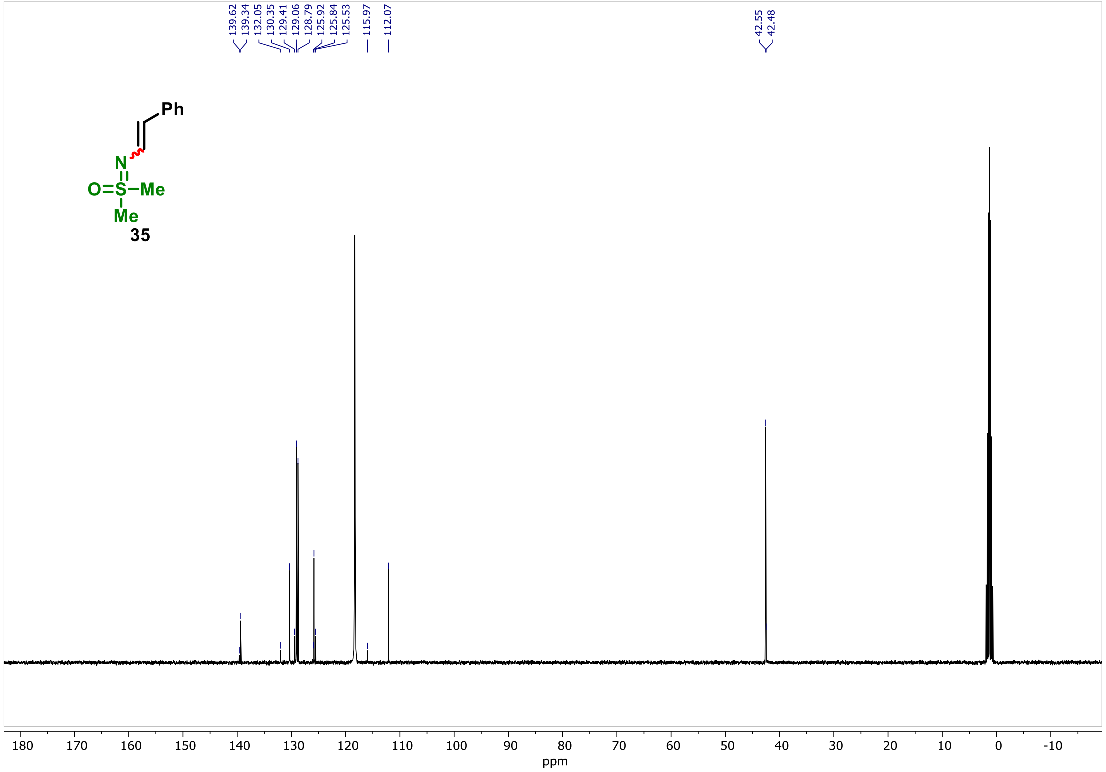

$^{1}H$ NMR (400 MHz, $CD_3CN$) spectra of compound **36**

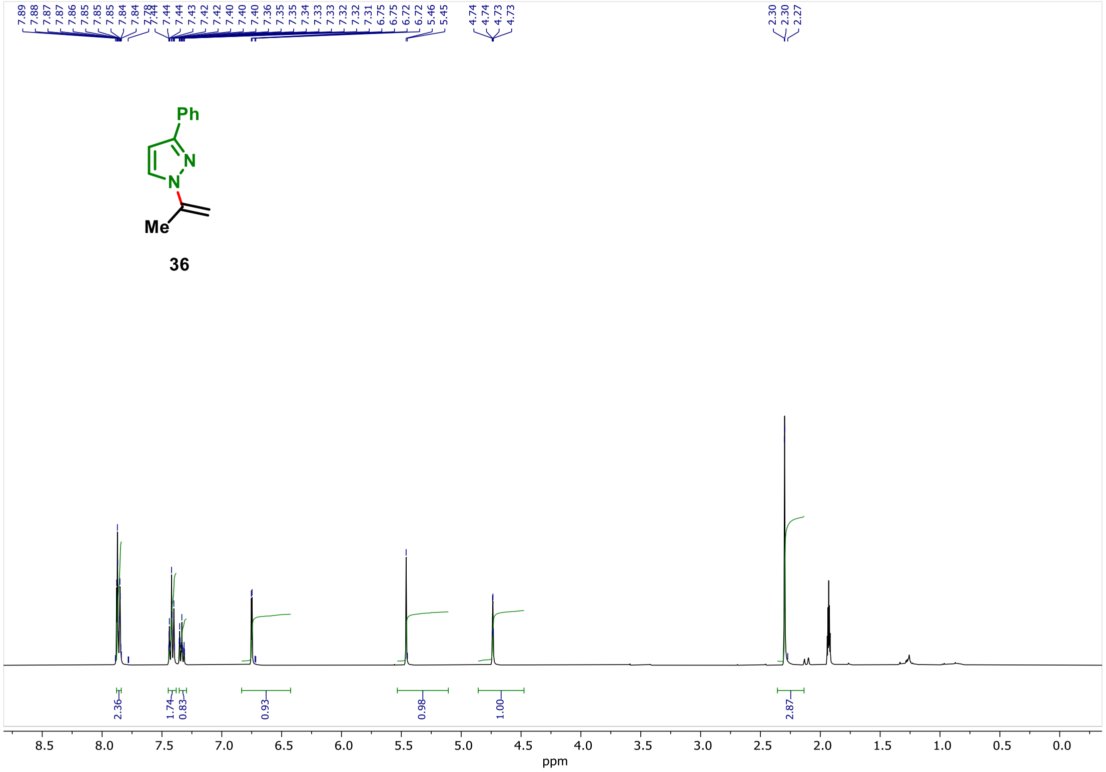


$^{13}C$ NMR (101 MHz, $CD_3CN$) spectra of compound **36**

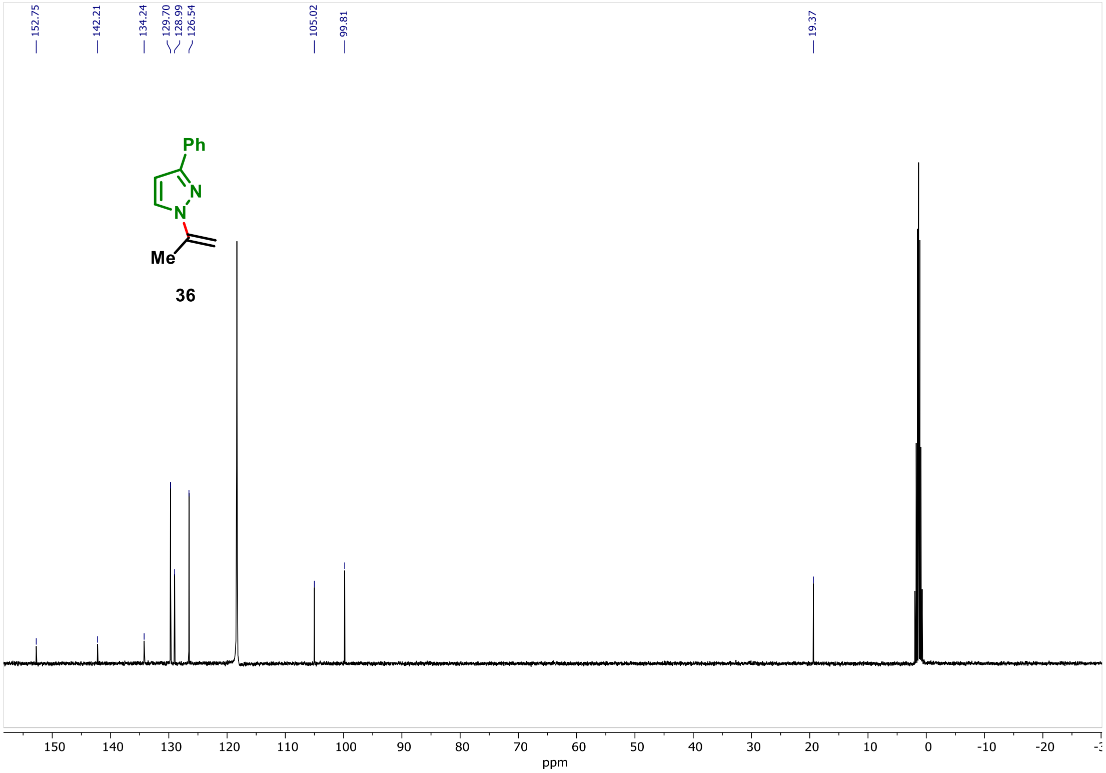

$^{1}$H NMR (400 MHz, $CD_3CN$) spectra of compound **37**

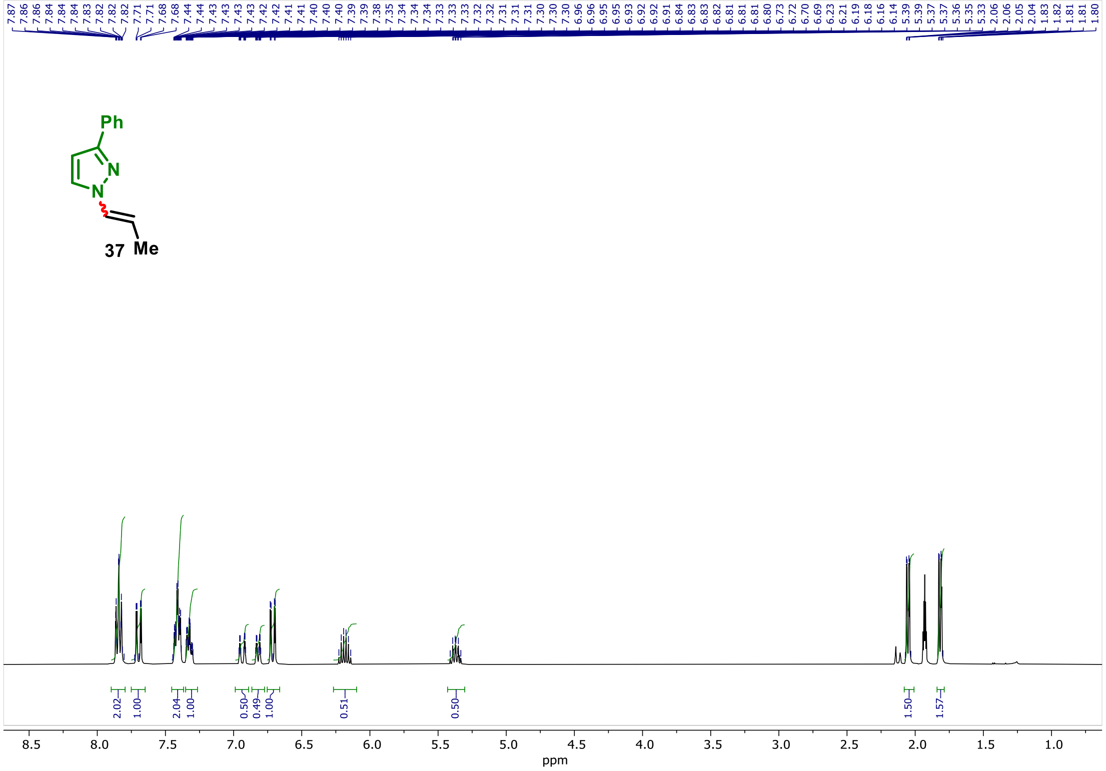

$^{13}$C NMR (101 MHz, $CD_3CN$) spectra of compound **37**

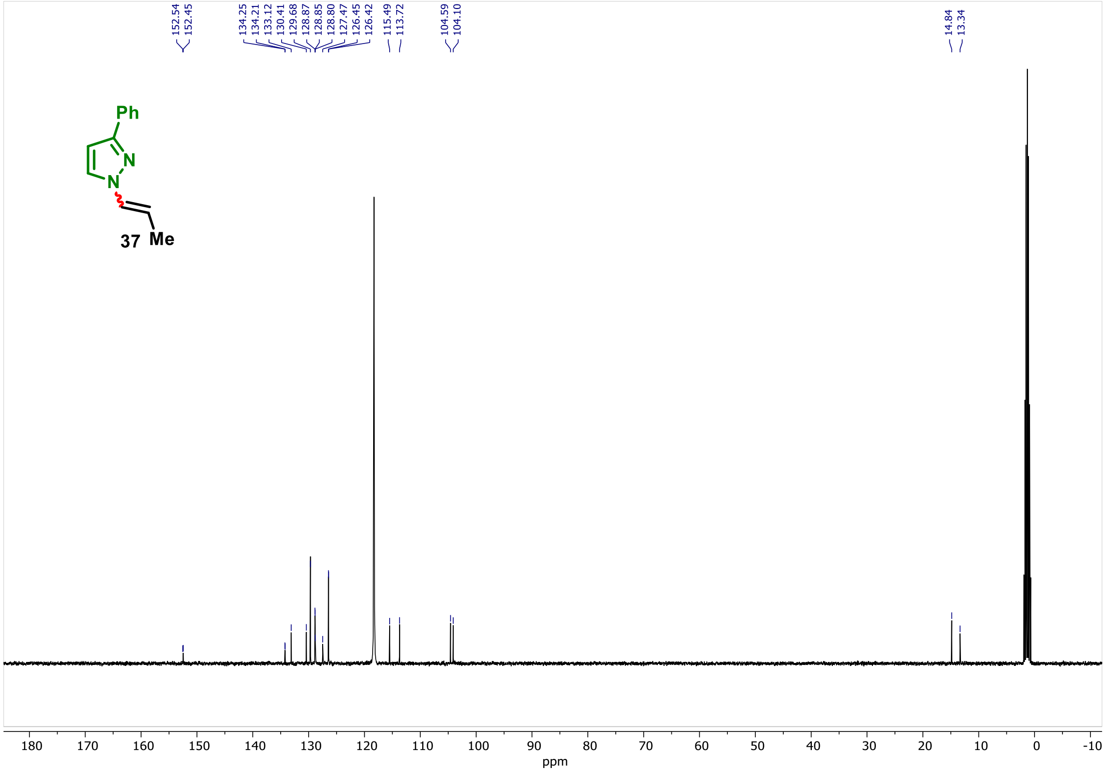

$^{1}$H NMR (400 MHz, $CD_3CN$) spectra of compound **38**

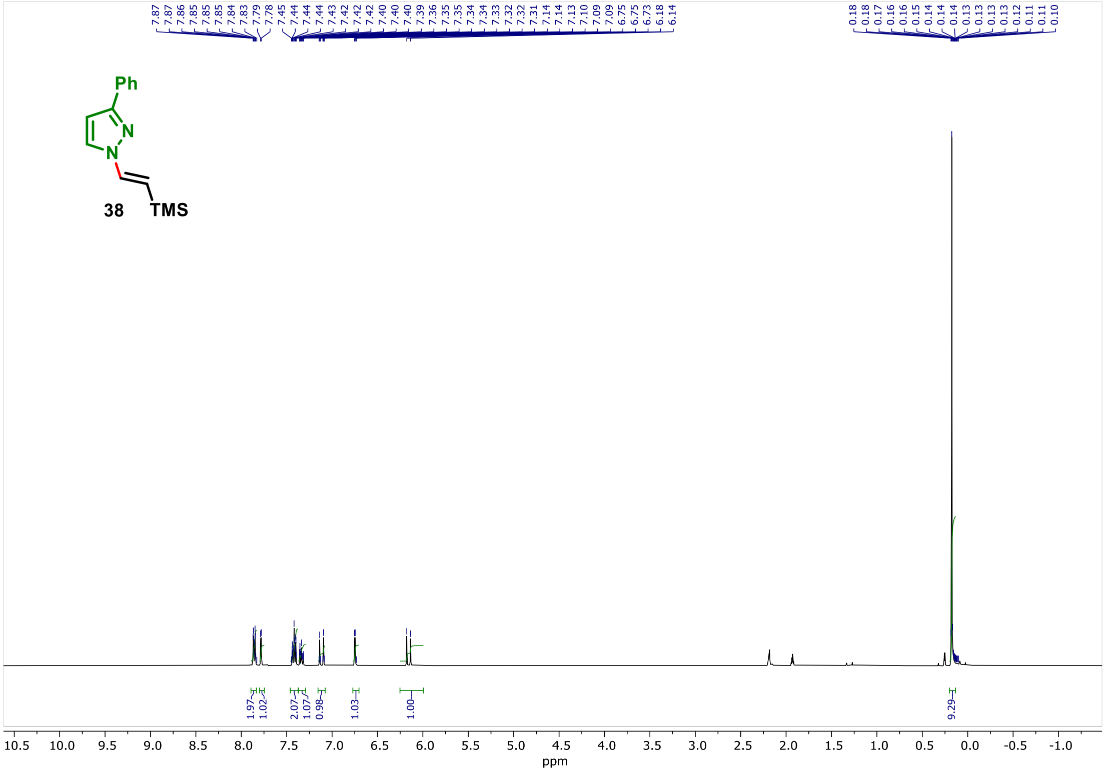

$^{13}$C NMR (101 MHz, $CD_3CN$) spectra of compound **38**

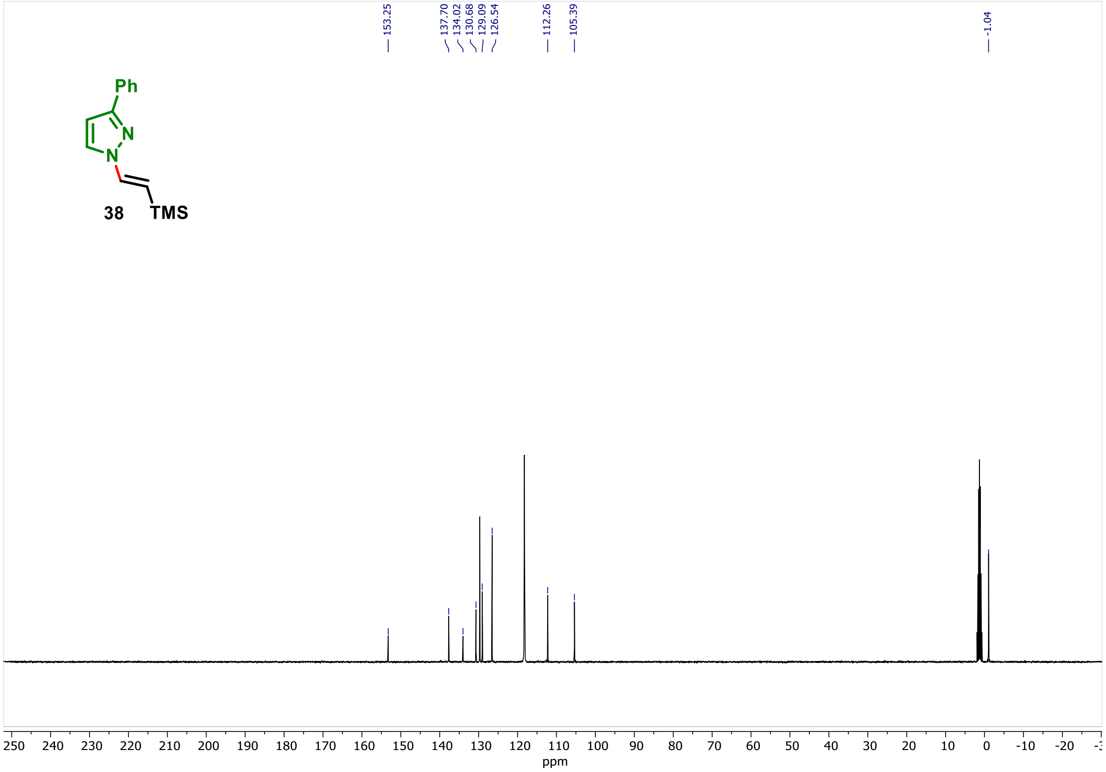

$^{1}$H NMR (400 MHz, $CD_3CN$) spectra of compound **39**

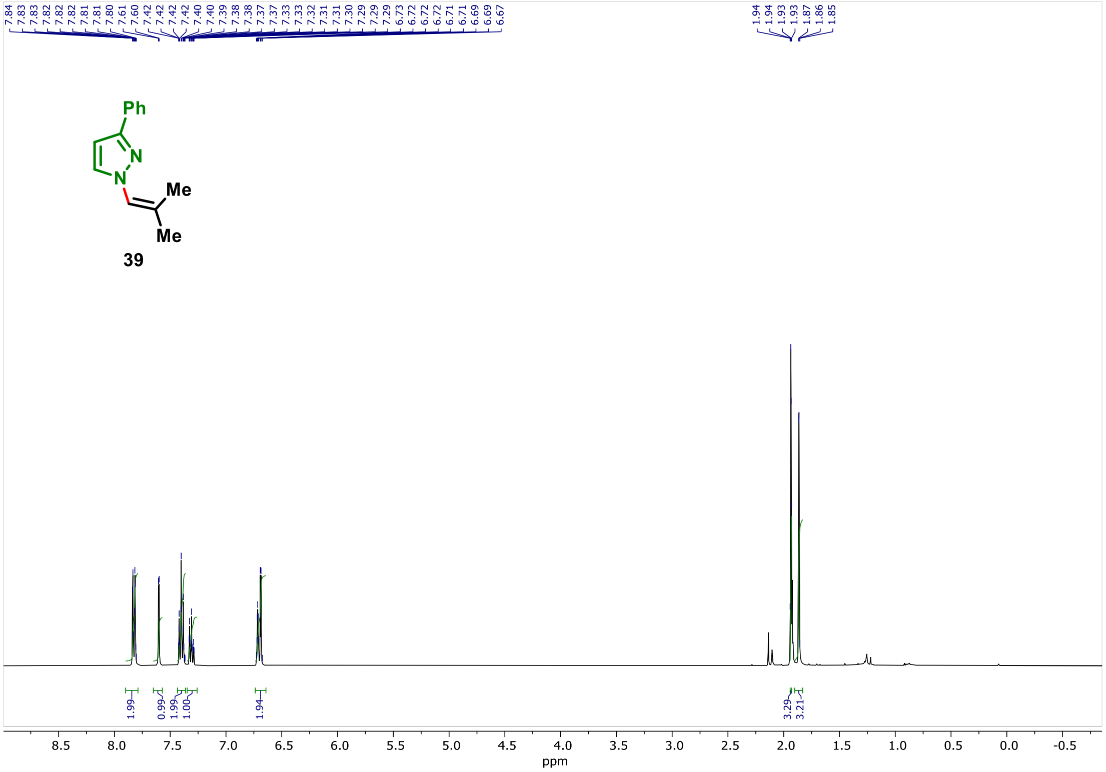

$^{13}$C NMR (101 MHz, $CD_3CN$) spectra of compound **39**

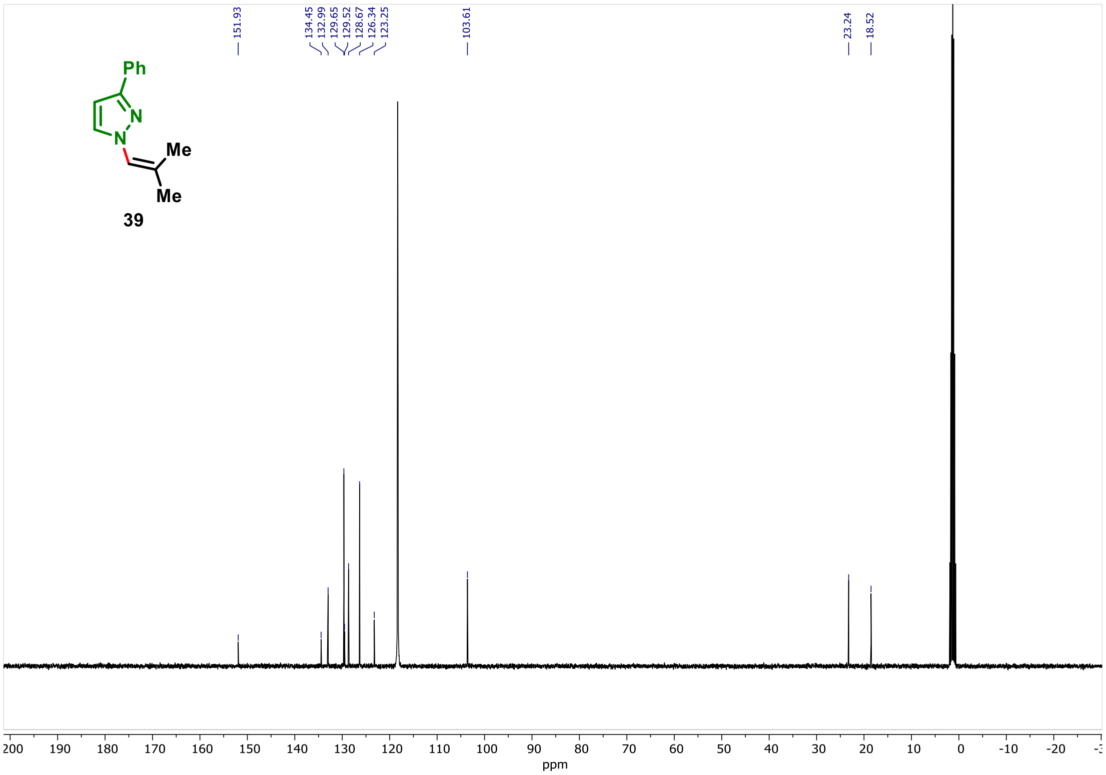

$^{1}$H NMR (400 MHz, $CD_3CN$) spectra of compound **40**

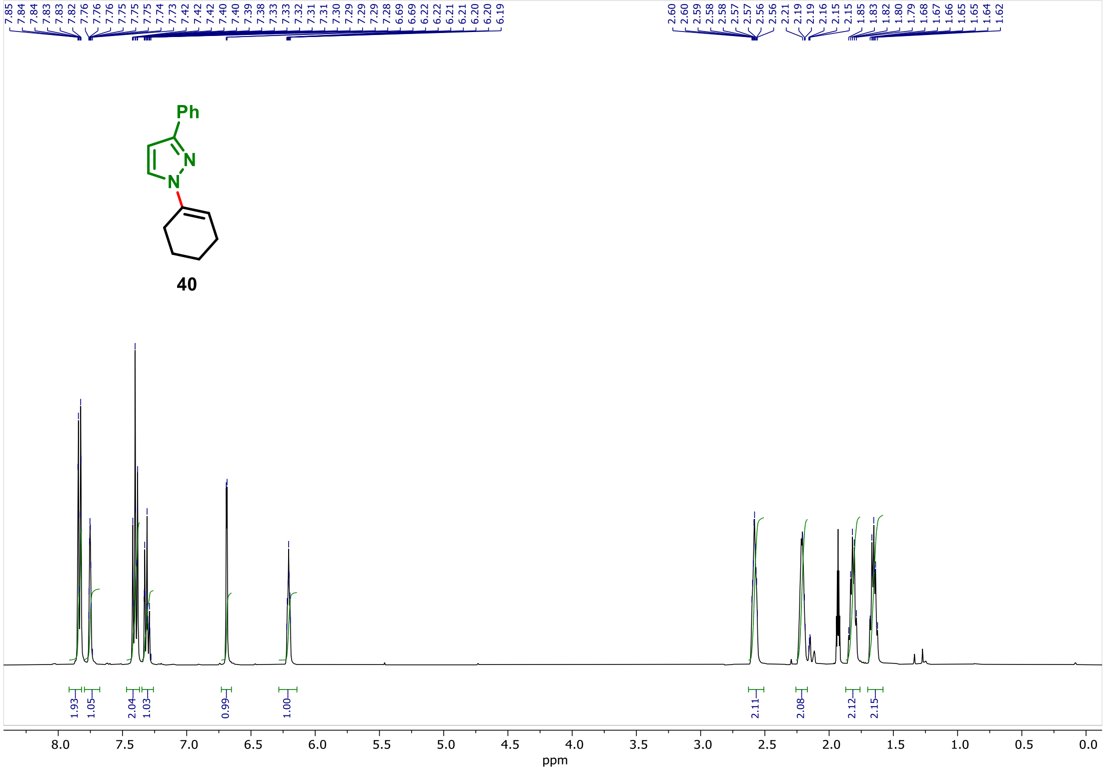


$^{13}$C NMR (101 MHz, $CD_3CN$) spectra of compound **40**

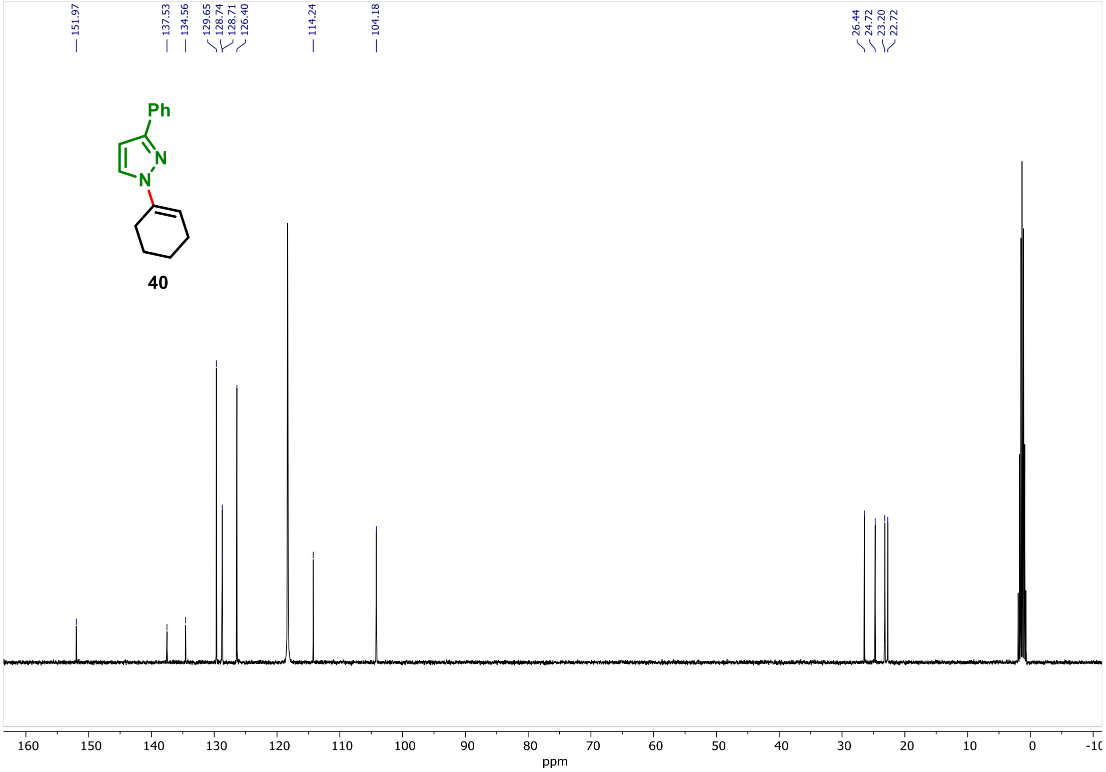

$^{1}$H NMR (400 MHz, $CD_3CN$) spectra of compound **41**

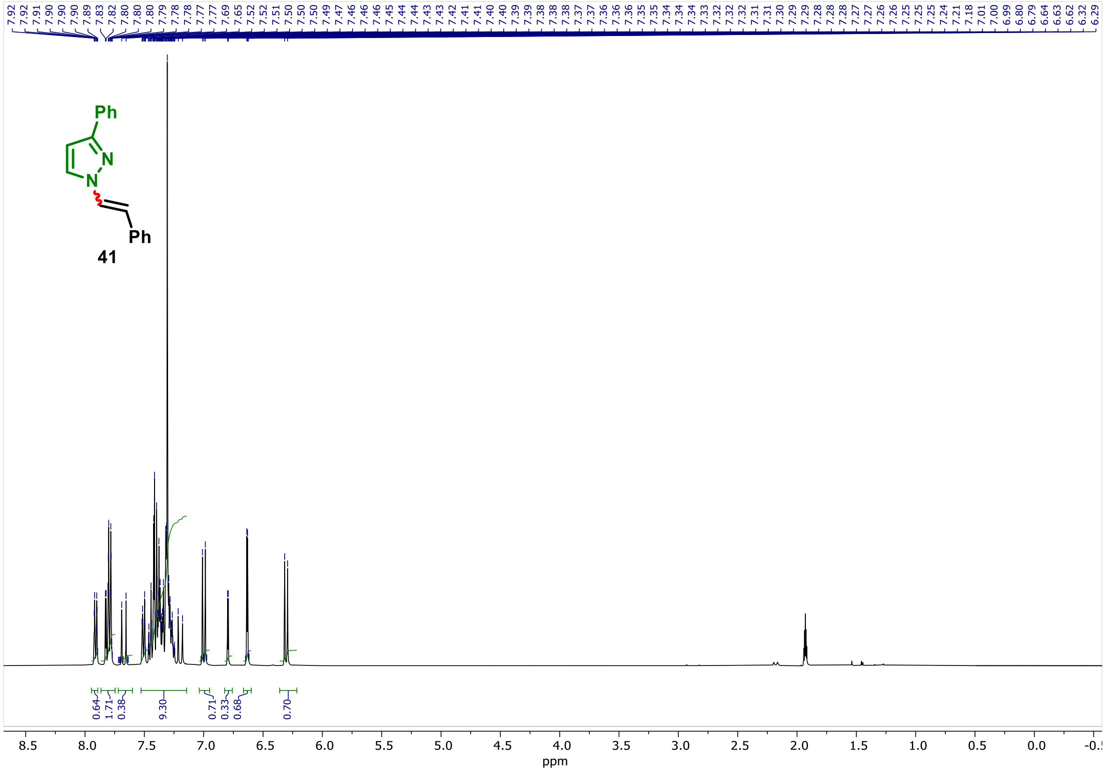

$^{13}$C NMR (101 MHz, $CD_3CN$) spectra of compound **41**

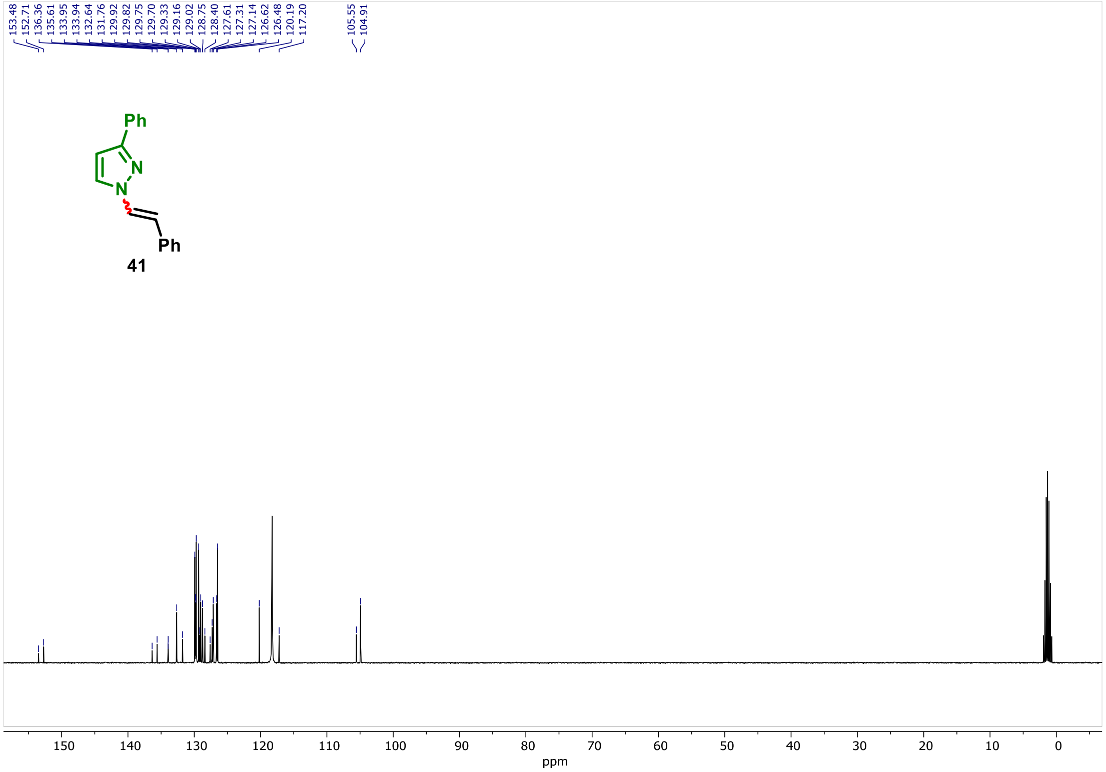

$^{1}$H NMR (400 MHz, $CD_3CN$) spectra of compound **42**

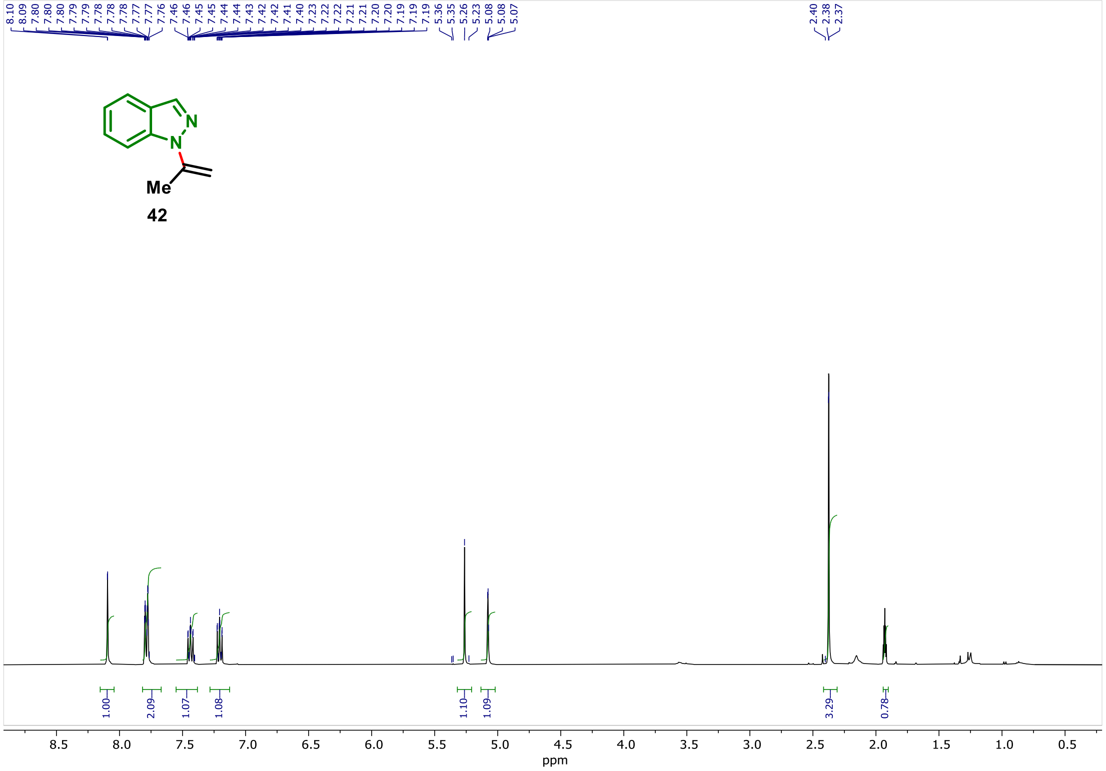


$^{13}$C NMR (101 MHz, $CD_3CN$) spectra of compound **42**

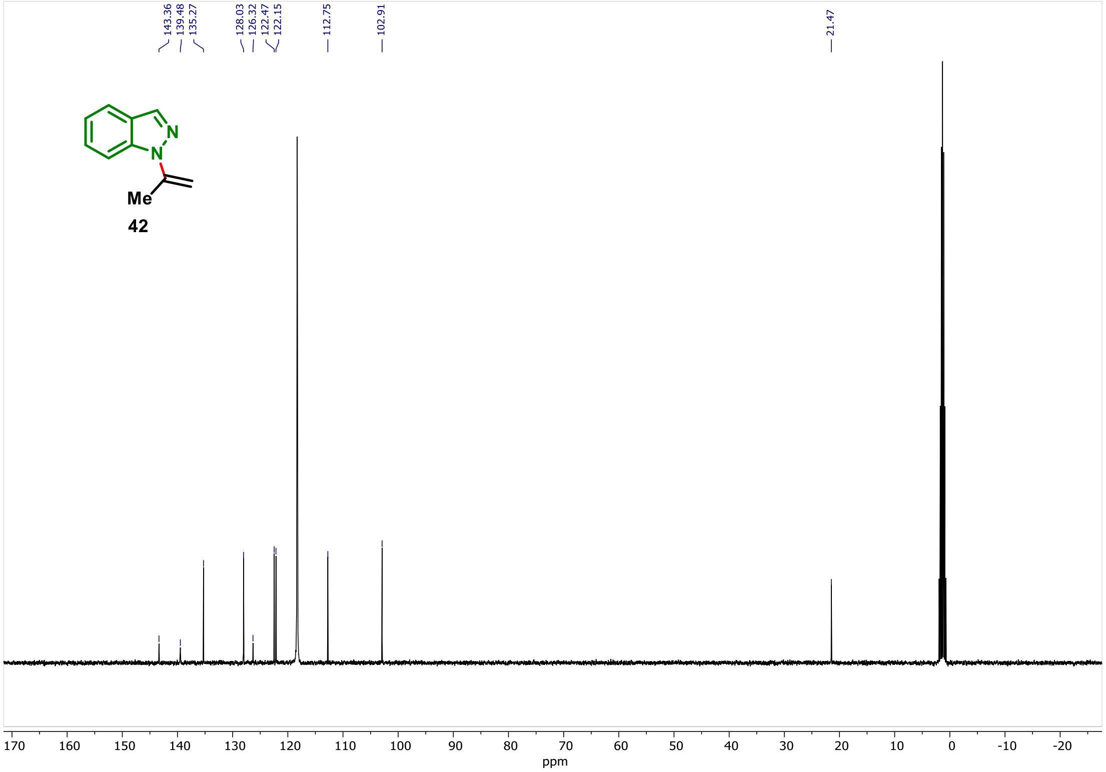

$^{1}$H NMR (400 MHz, $CD_3CN$) spectra of compound **43**

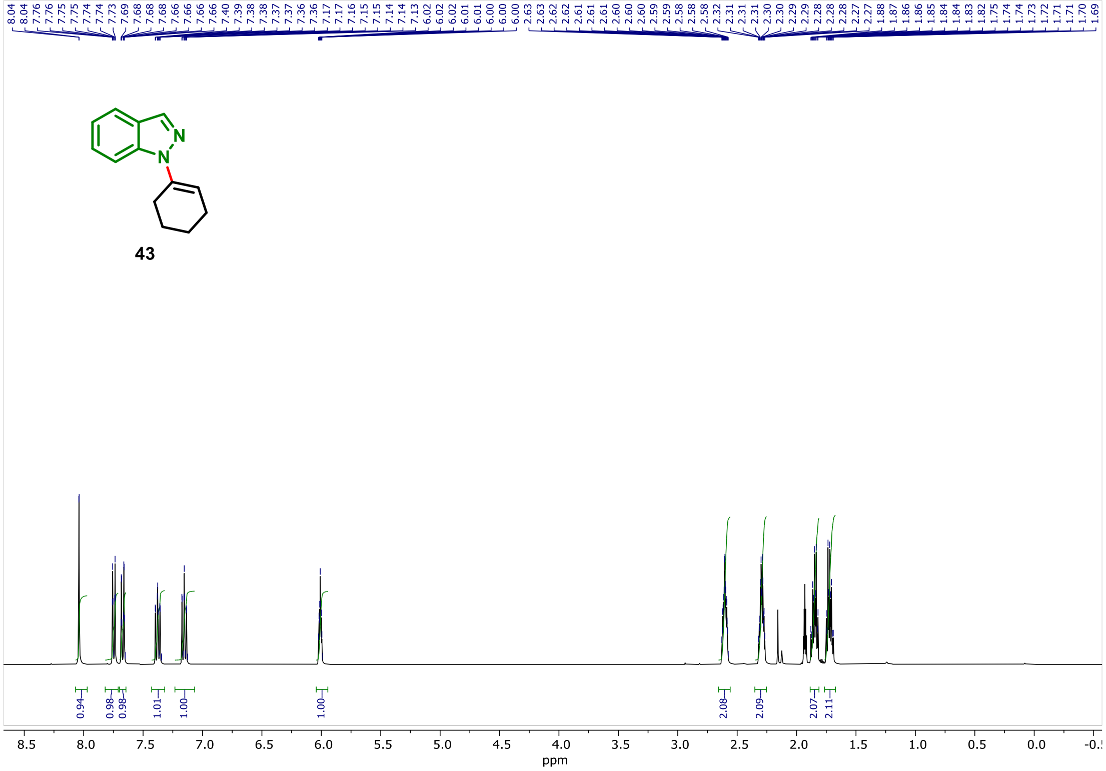


$^{13}$C NMR (101 MHz, $CD_3CN$) spectra of compound **43**

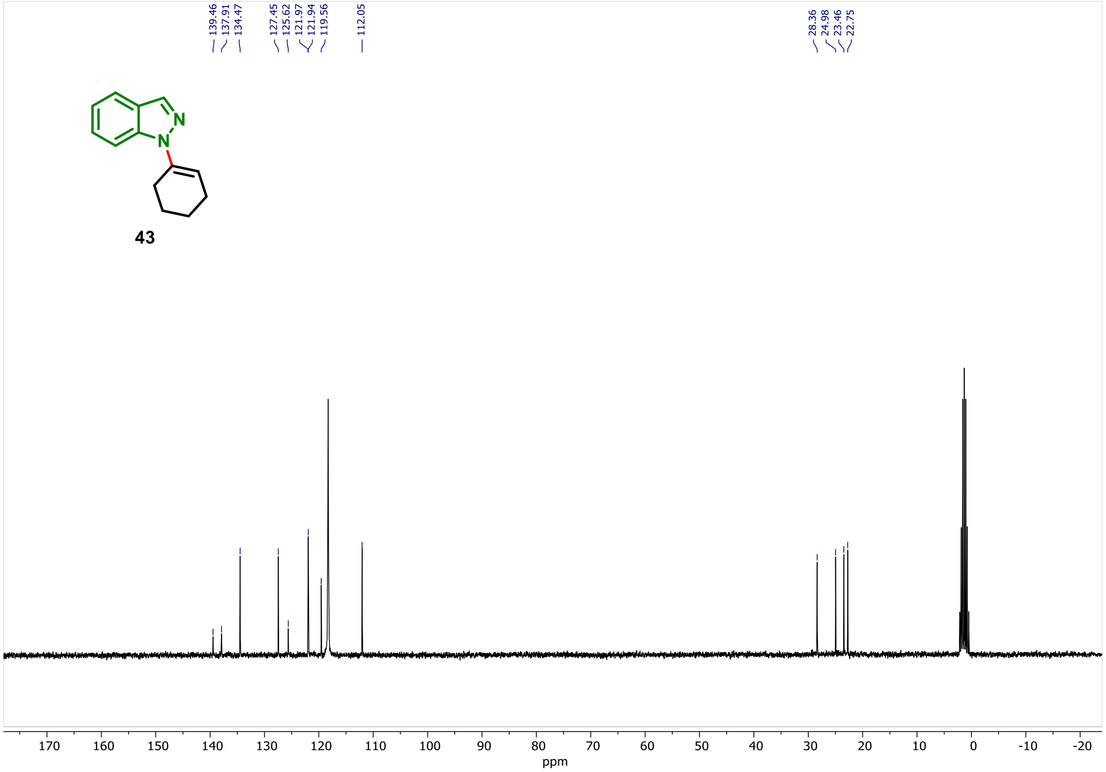

$^{1}$H NMR (400 MHz, $CD_3CN$) spectra of compound **44**

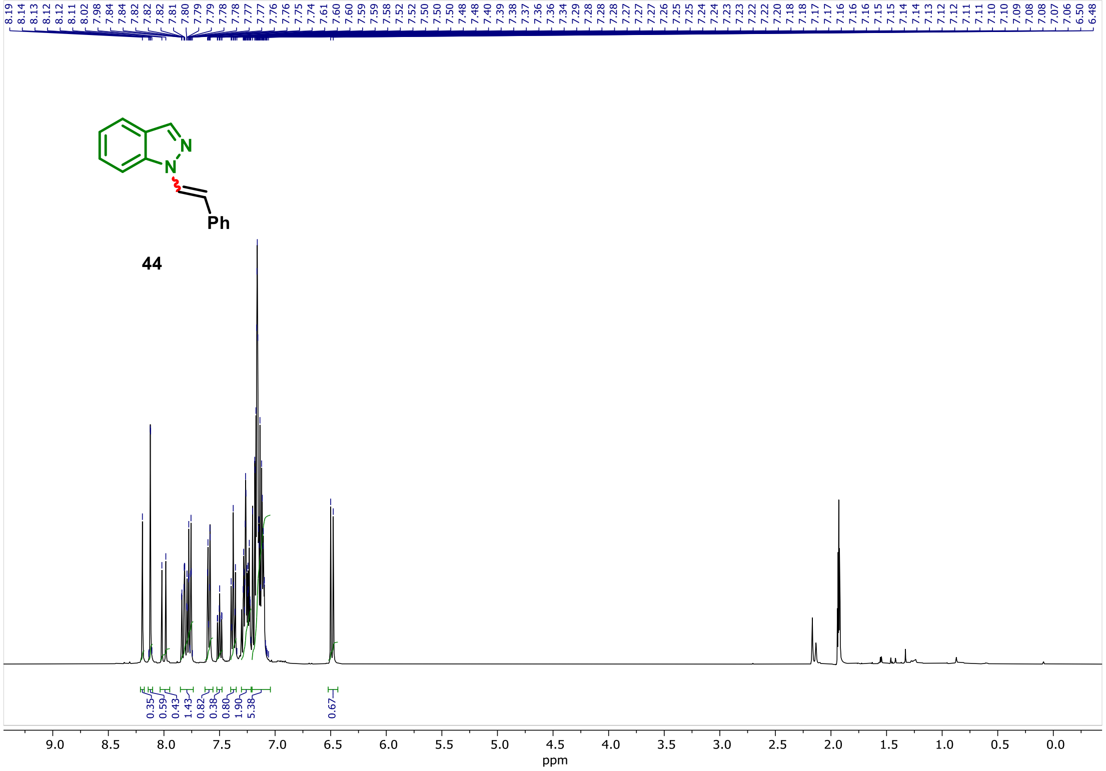


$^{13}$C NMR (101 MHz, $CD_3CN$) spectra of compound **44**

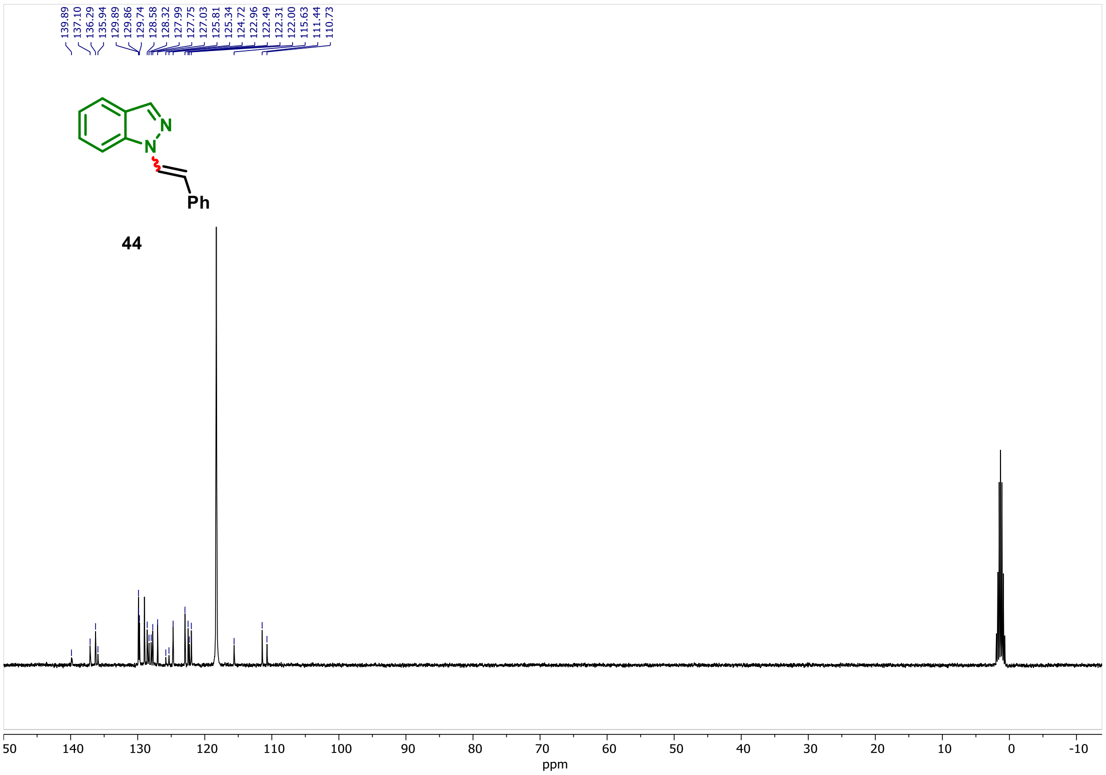

$^{1}$H NMR (400 MHz, $CD_3CN$) spectra of compound **45**

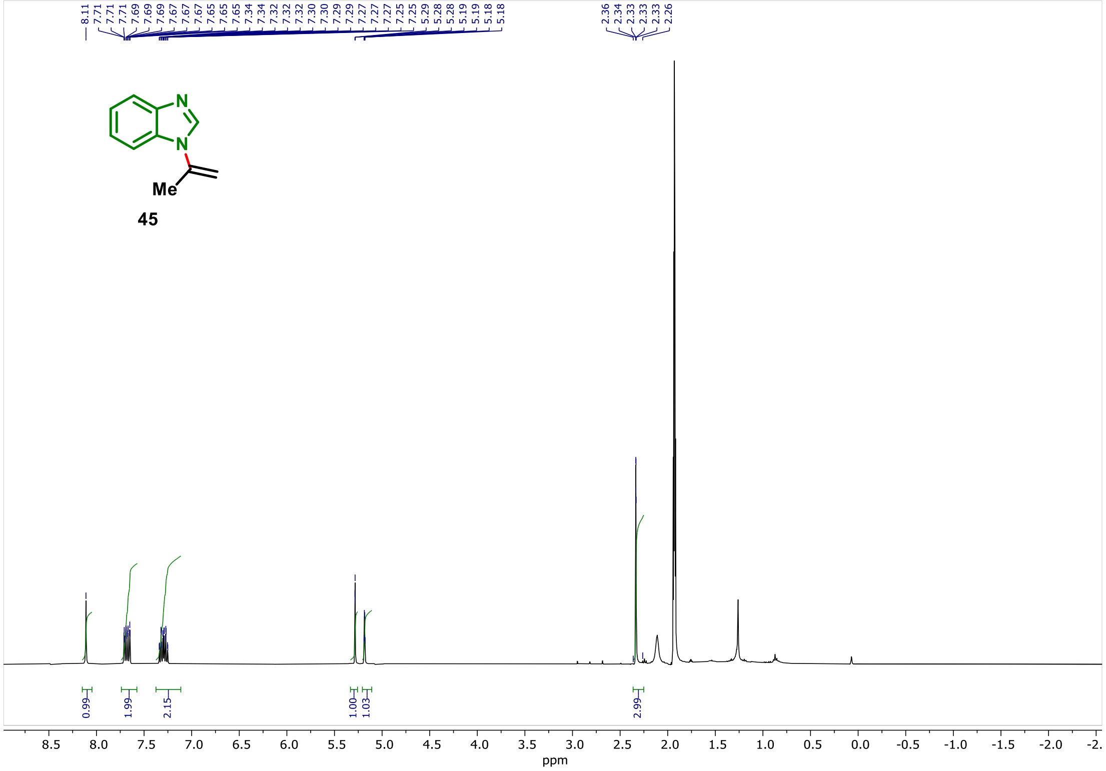


$^{13}$C NMR (101 MHz, $CD_3CN$) spectra of compound **45**

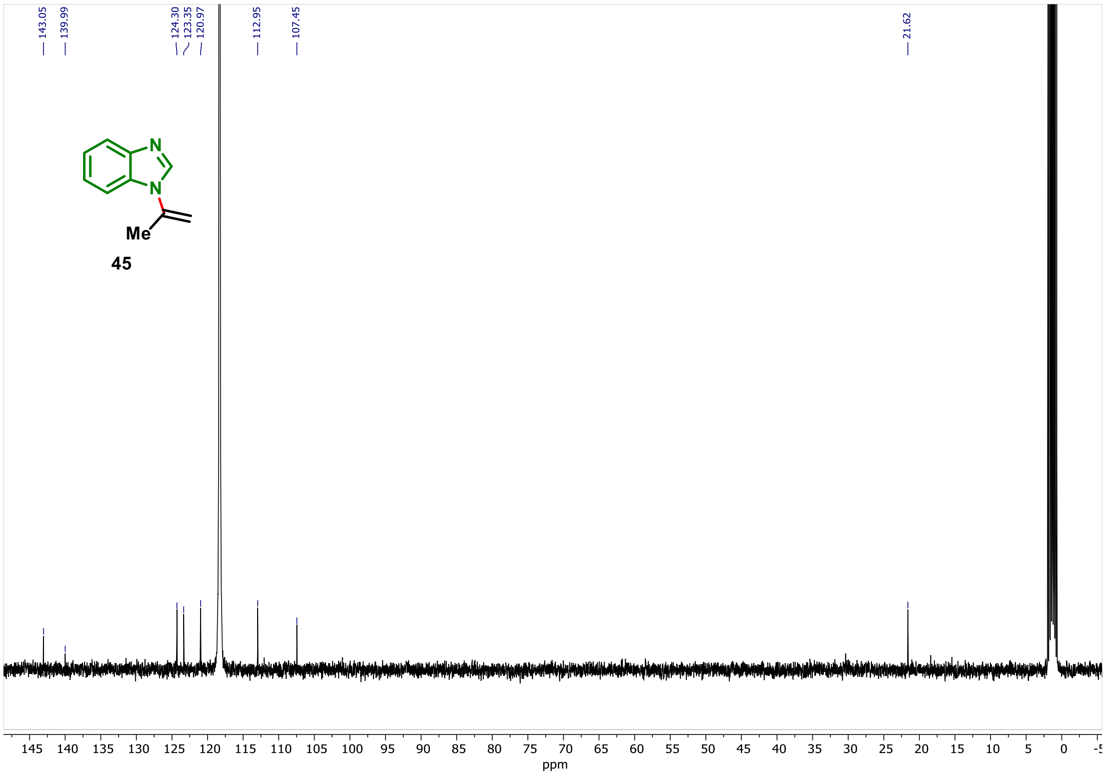

$^{1}$H NMR (400 MHz, $CD_3CN$) spectra of compound **46**

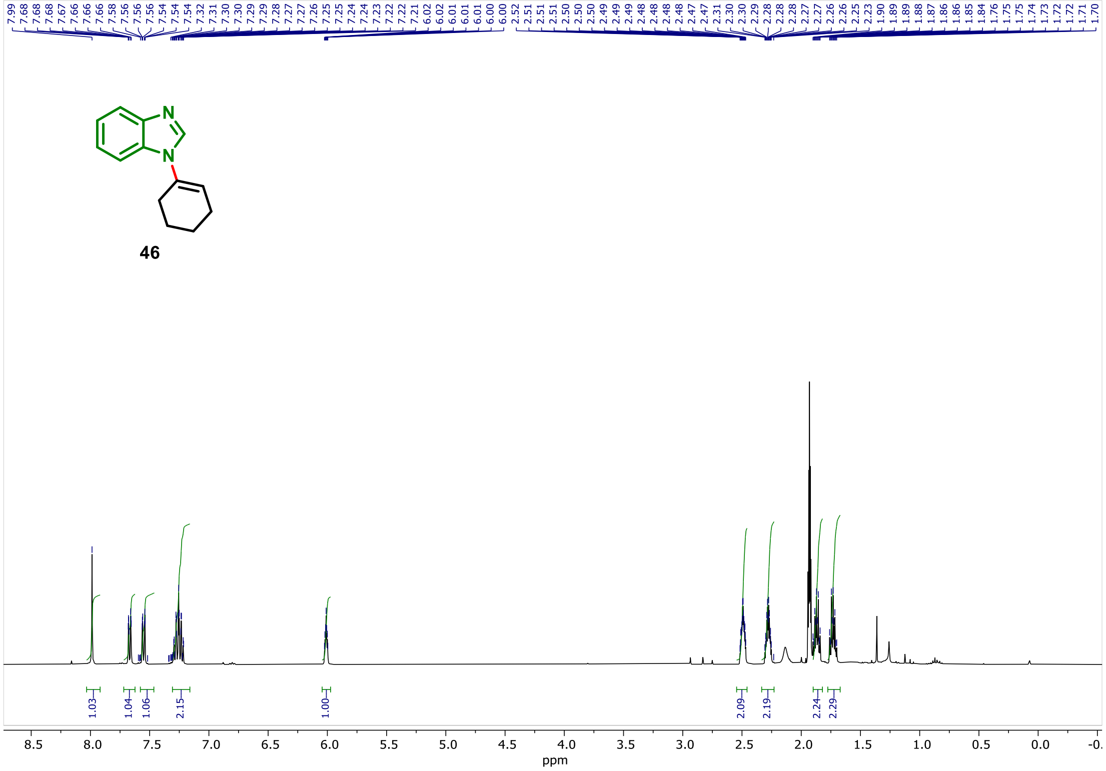


$^{13}$C NMR (101 MHz, $CD_3CN$) spectra of compound **46**

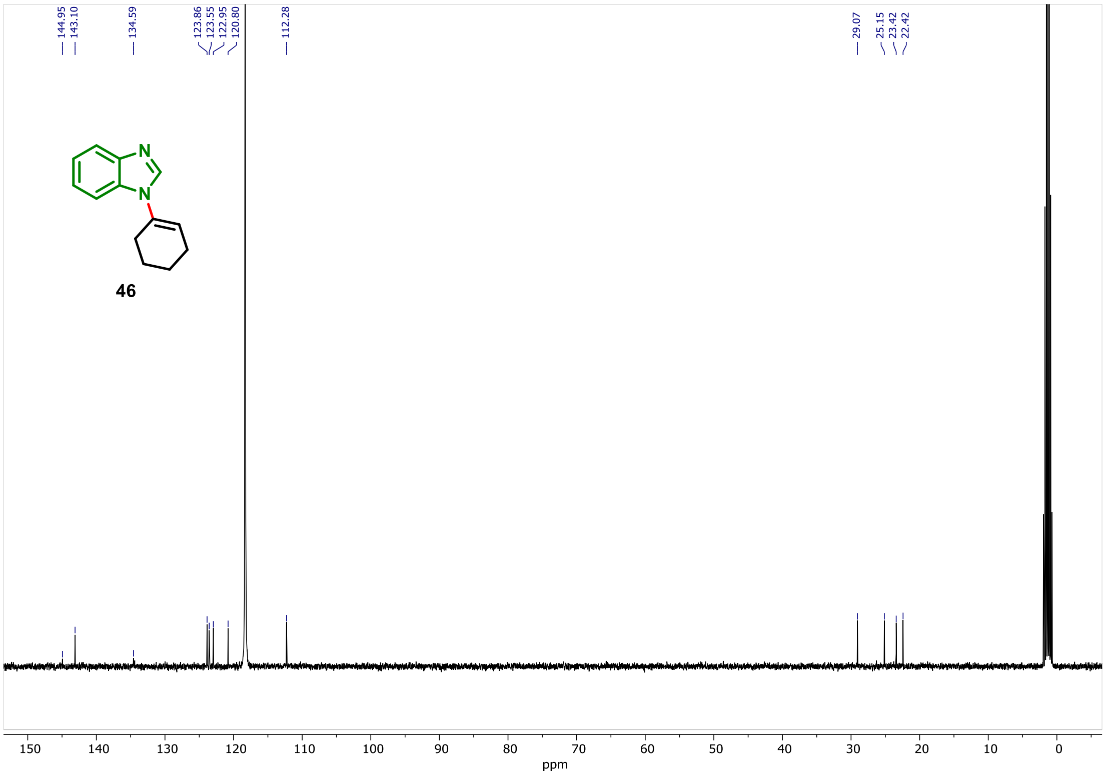

$^{1}H$ NMR (400 MHz, $CD_3CN$) spectra of compound **47**

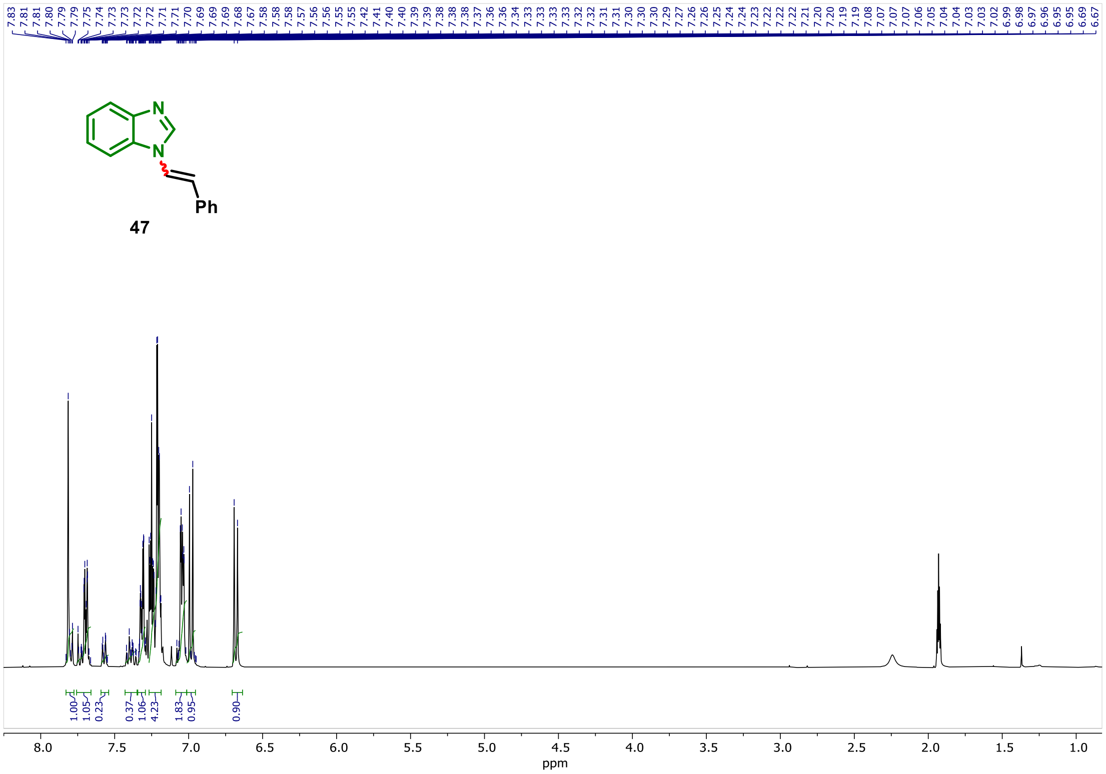

$^{13}C$ NMR (101 MHz, $CD_3CN$) spectra of compound **47**

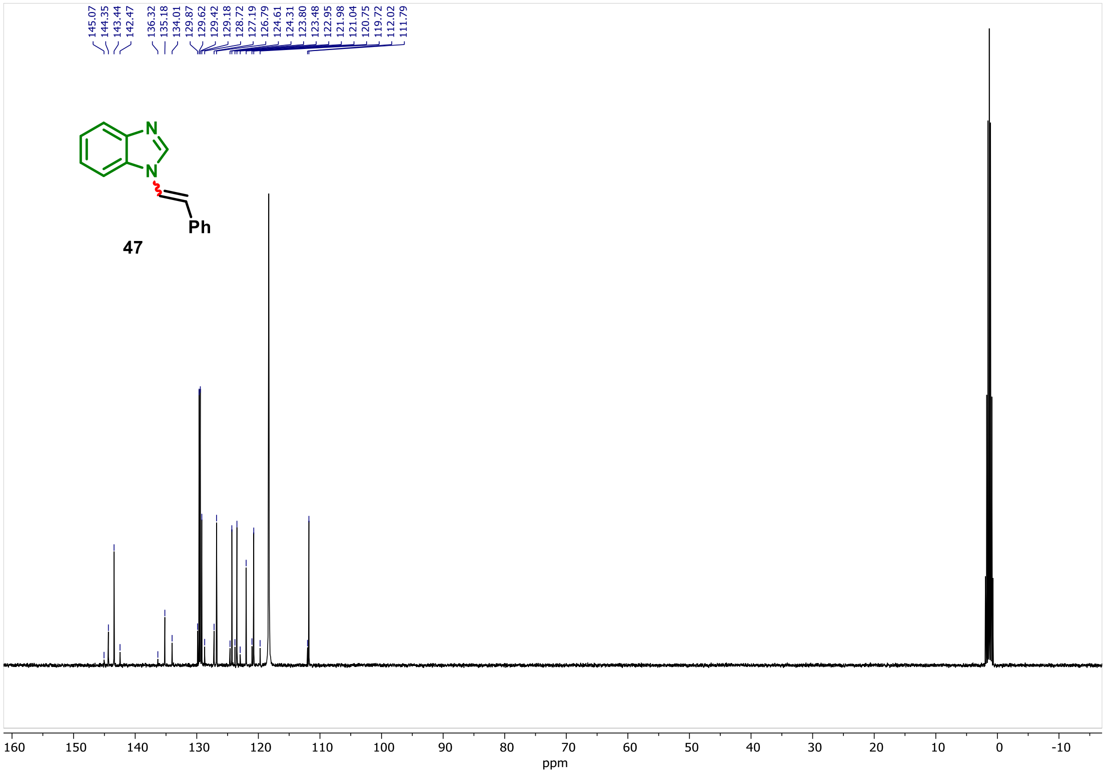

$^1H$ NMR (400 MHz, $CD_3CN$) spectra of compound **48**

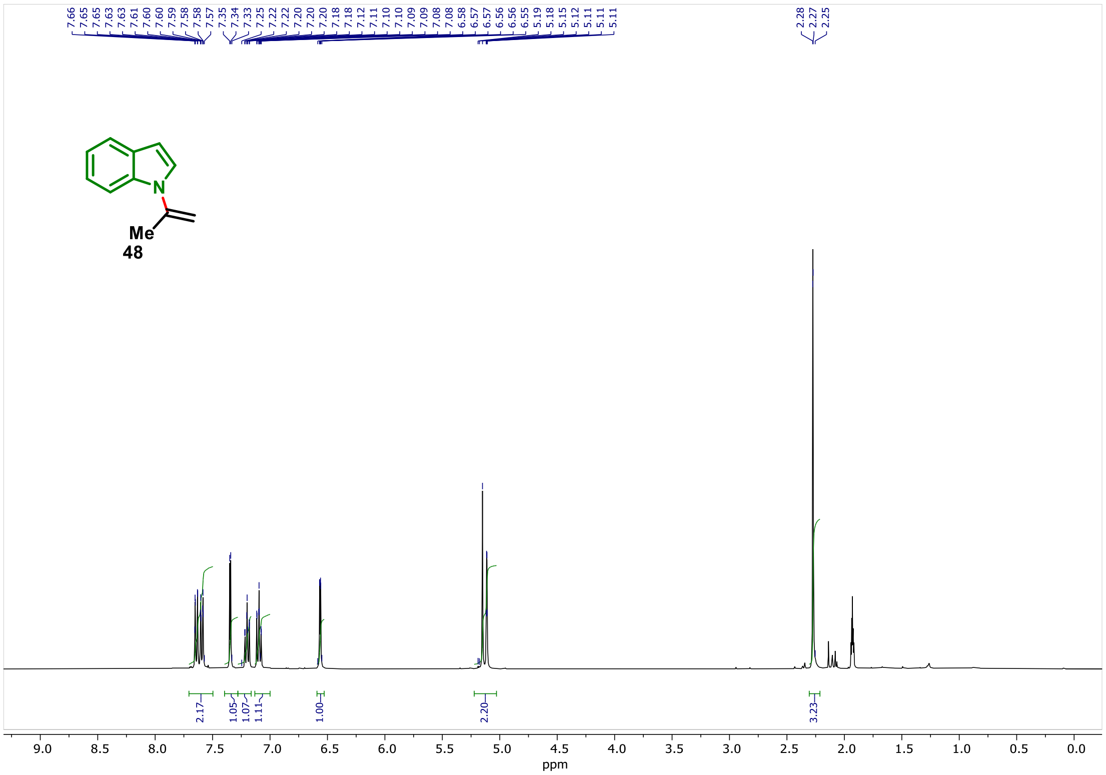


$^{13}C$ NMR (101 MHz, $CD_3CN$) spectra of compound **48**

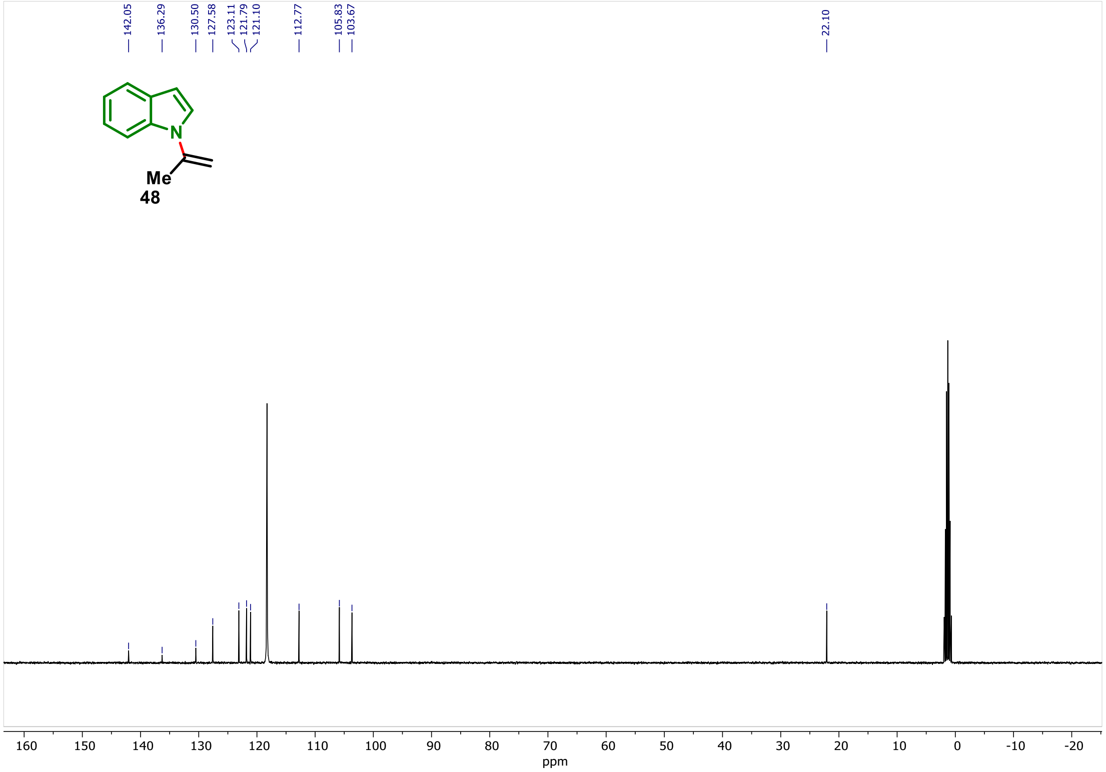

$^{1}H$ NMR (400 MHz, $CD_3CN$) spectra of compound **49**

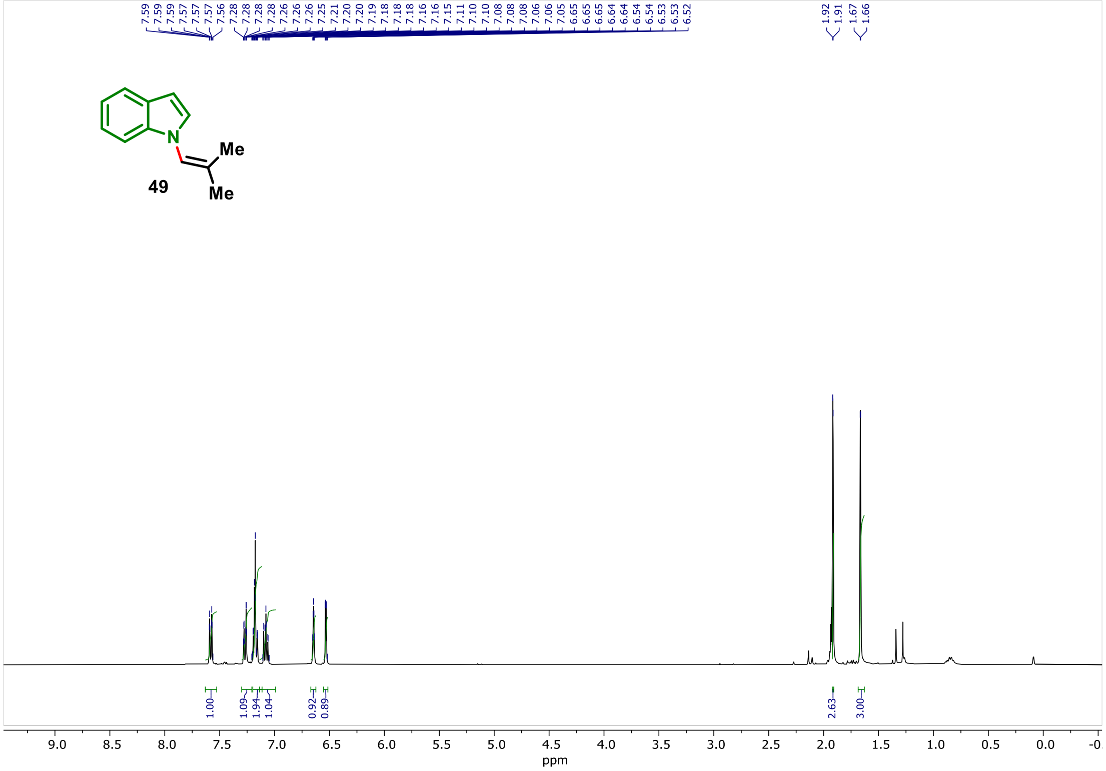


$^{13}C$ NMR (101 MHz, $CD_3CN$) spectra of compound **49**

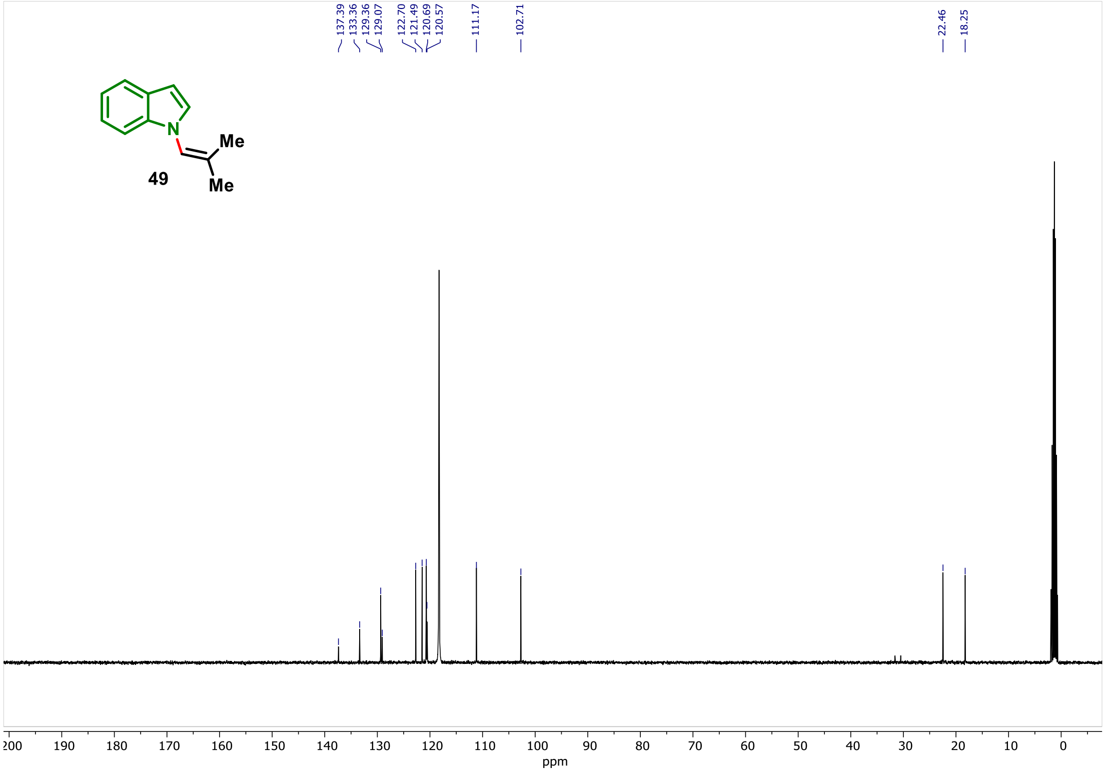

$^{1}$H NMR (400 MHz, $CD_3CN$) spectra of compound **50**

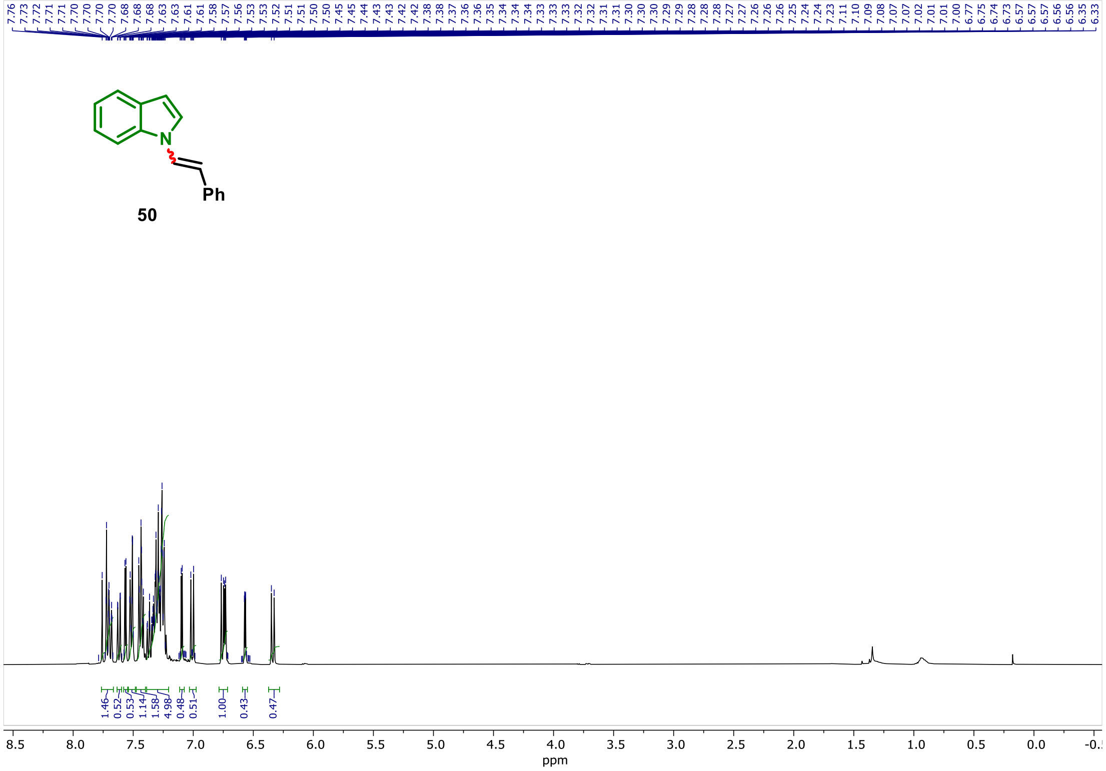


$^{13}$C NMR (101 MHz, $CD_3CN$) spectra of compound **50**

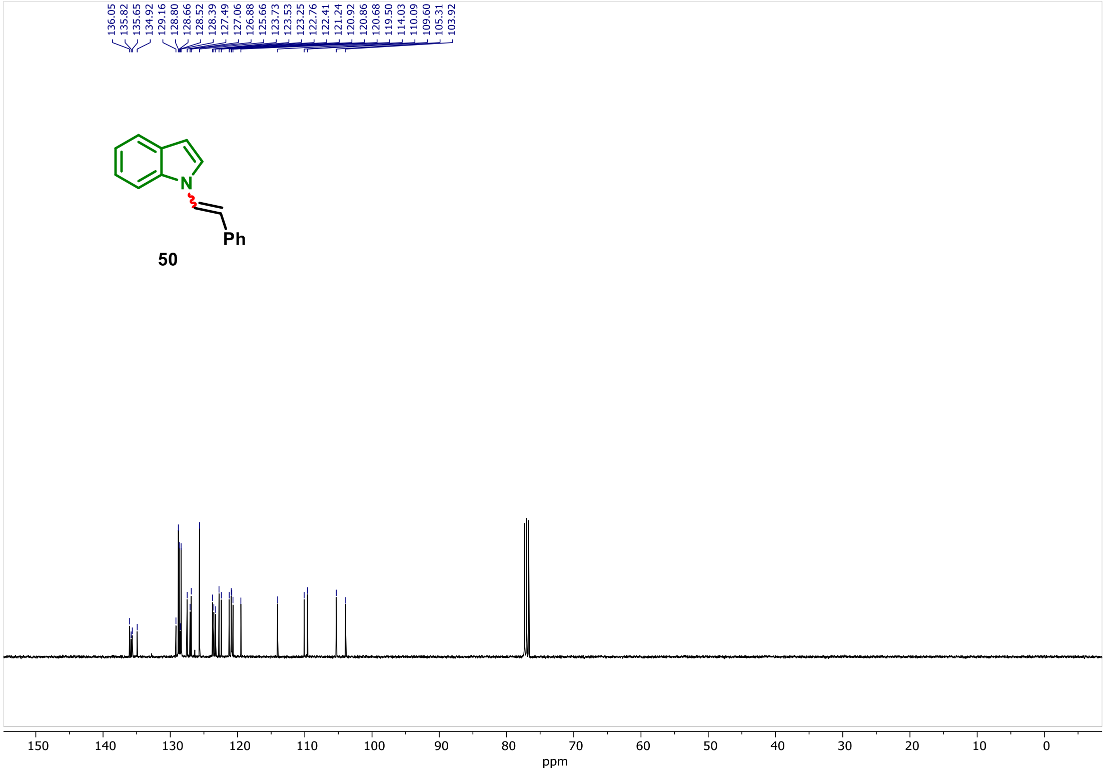

$^{1}H$ NMR (400 MHz, $CD_3CN$) spectra of compound **51**

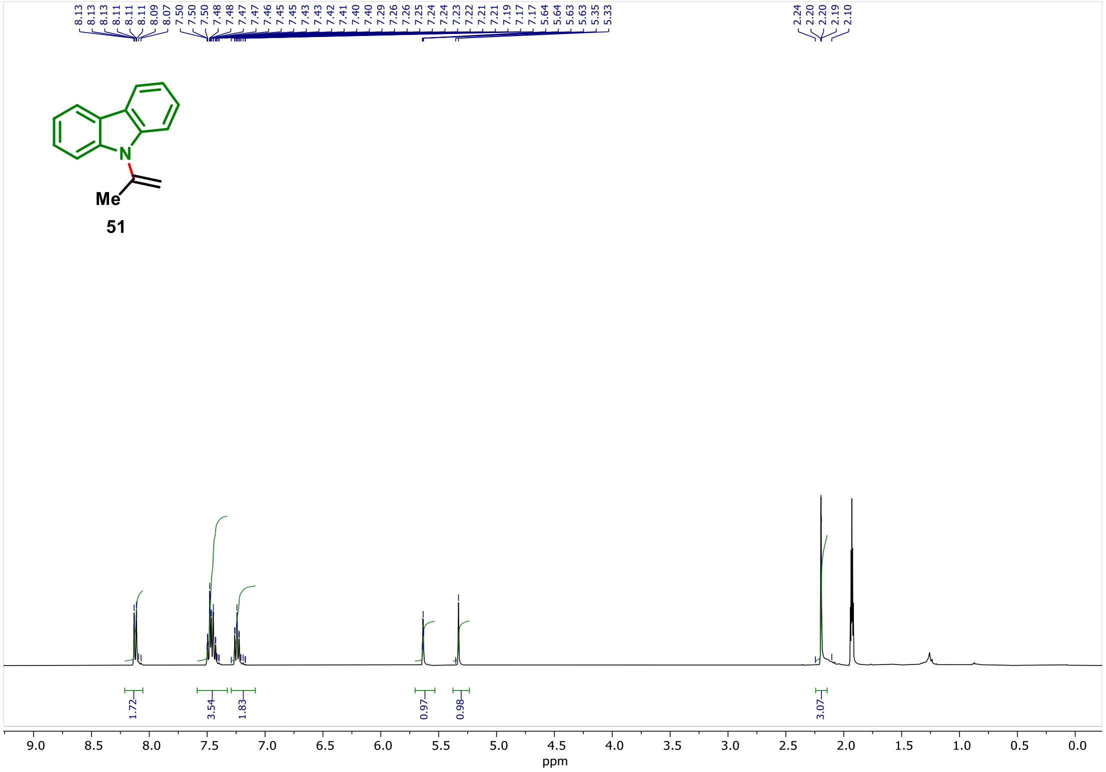

$^{13}C$ NMR (101 MHz, $CD_3CN$) spectra of compound **51**

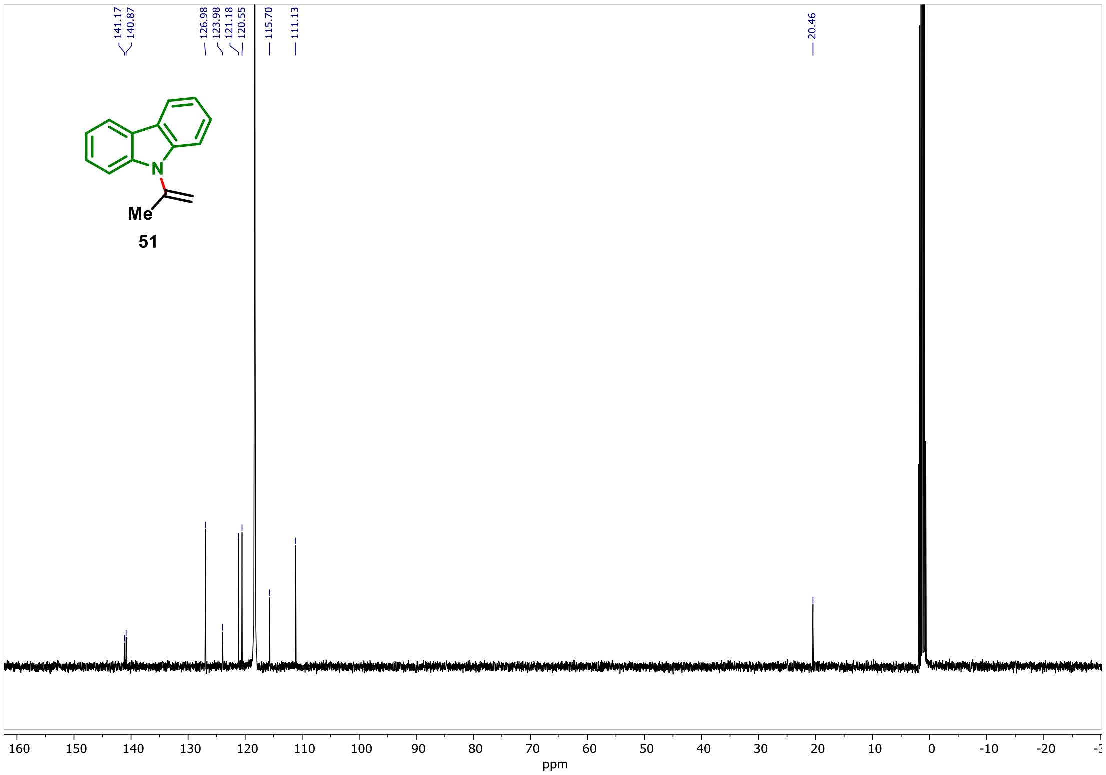

$^{1}$H NMR (400 MHz, $CD_3CN$) spectra of compound **52**

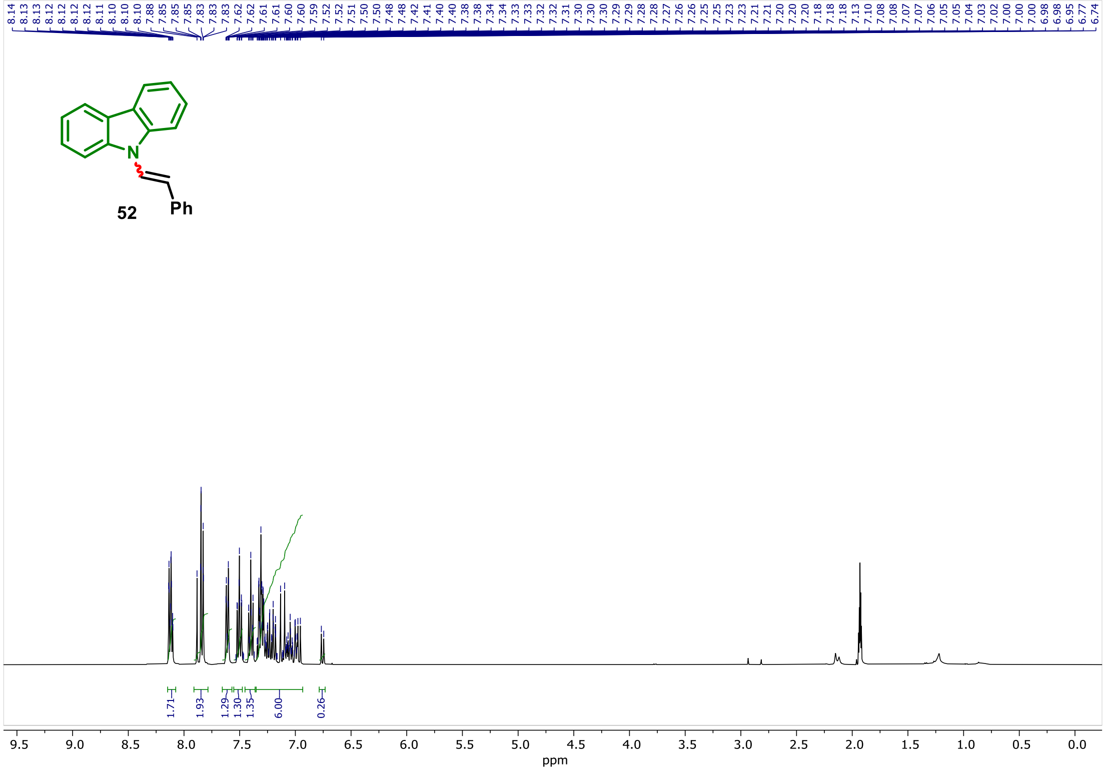


$^{13}$C NMR (101 MHz, $CD_3CN$) spectra of compound **52**

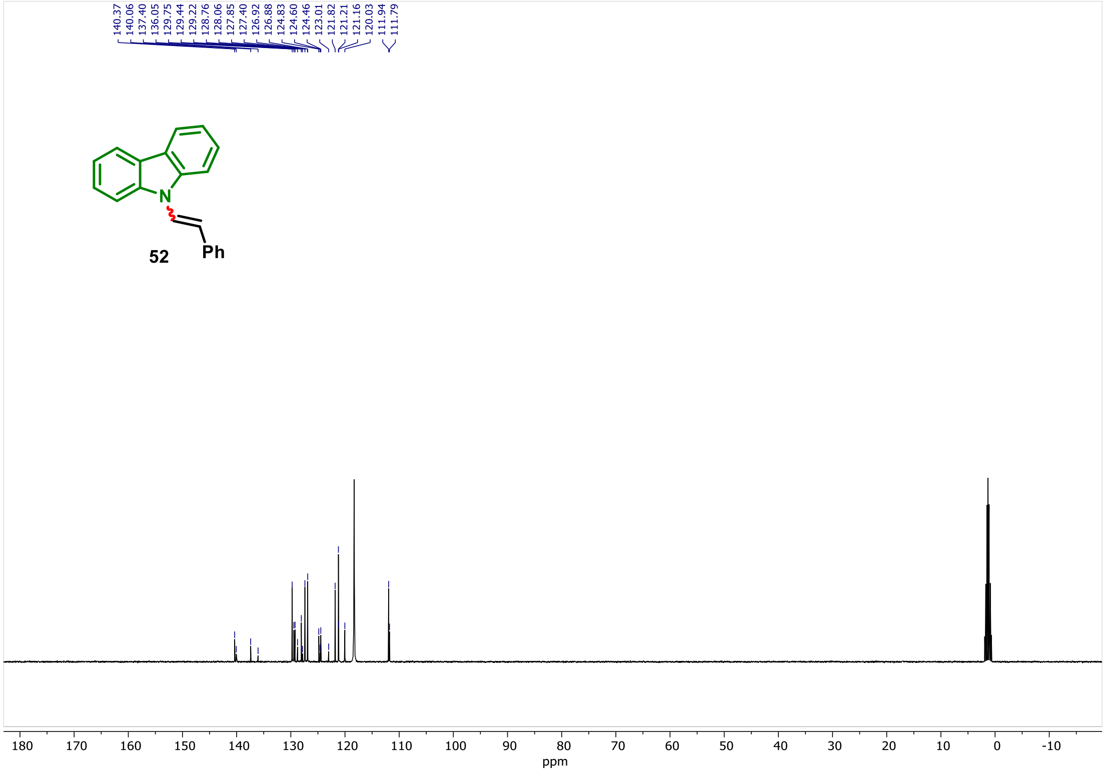

$^{1}H$ NMR (400 MHz, $CD_3CN$) spectra of compound **53**

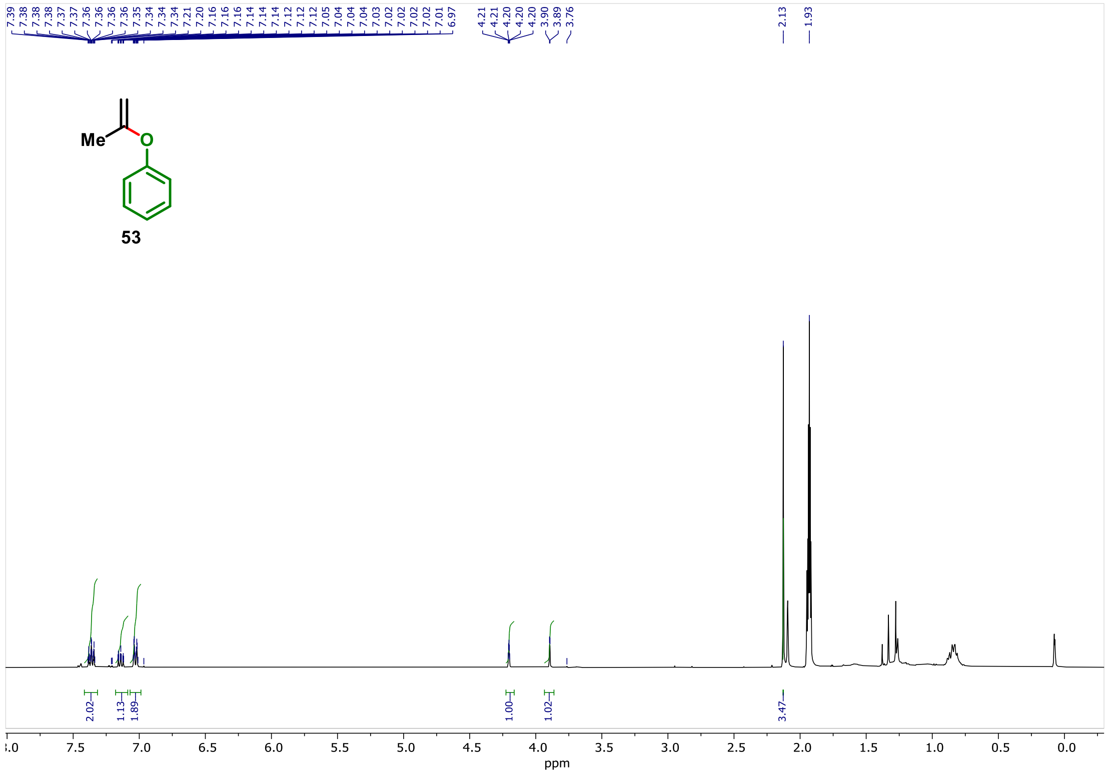

$^{13}C$ NMR (101 MHz, $CD_3CN$) spectra of compound **53**

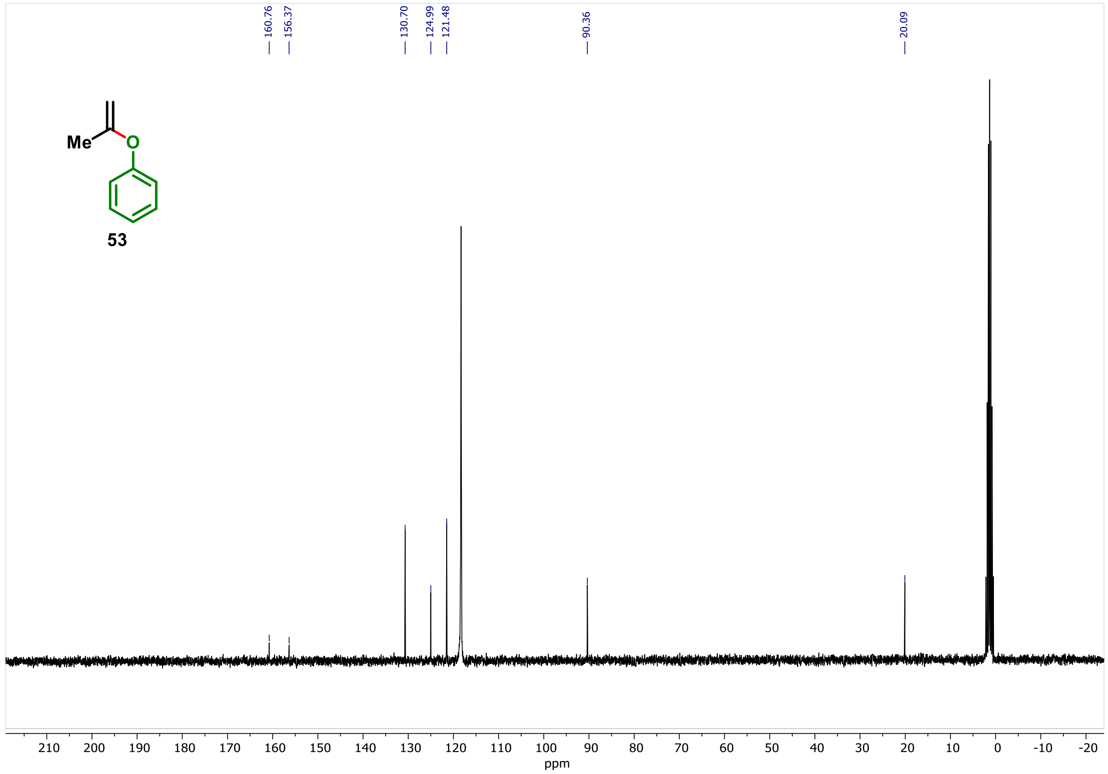

$^{1}$H NMR (400 MHz, $CD_3CN$) spectra of compound **54**

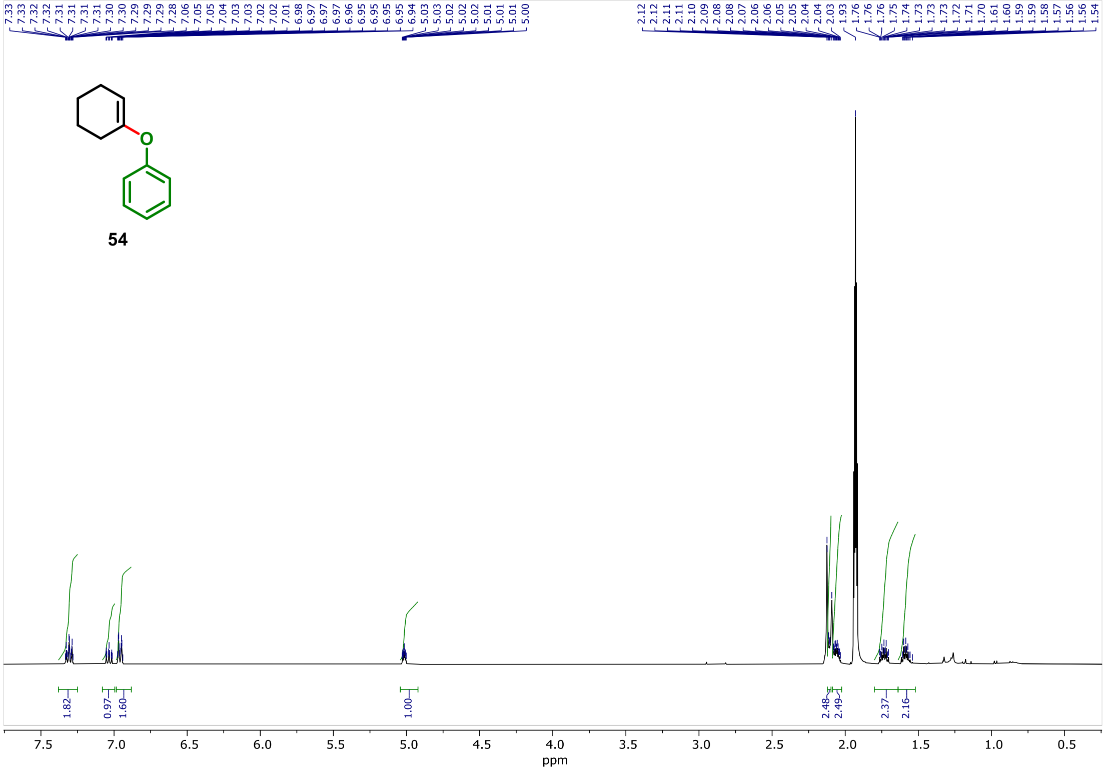

$^{13}$C NMR (101 MHz, $CD_3CN$) spectra of compound **54**

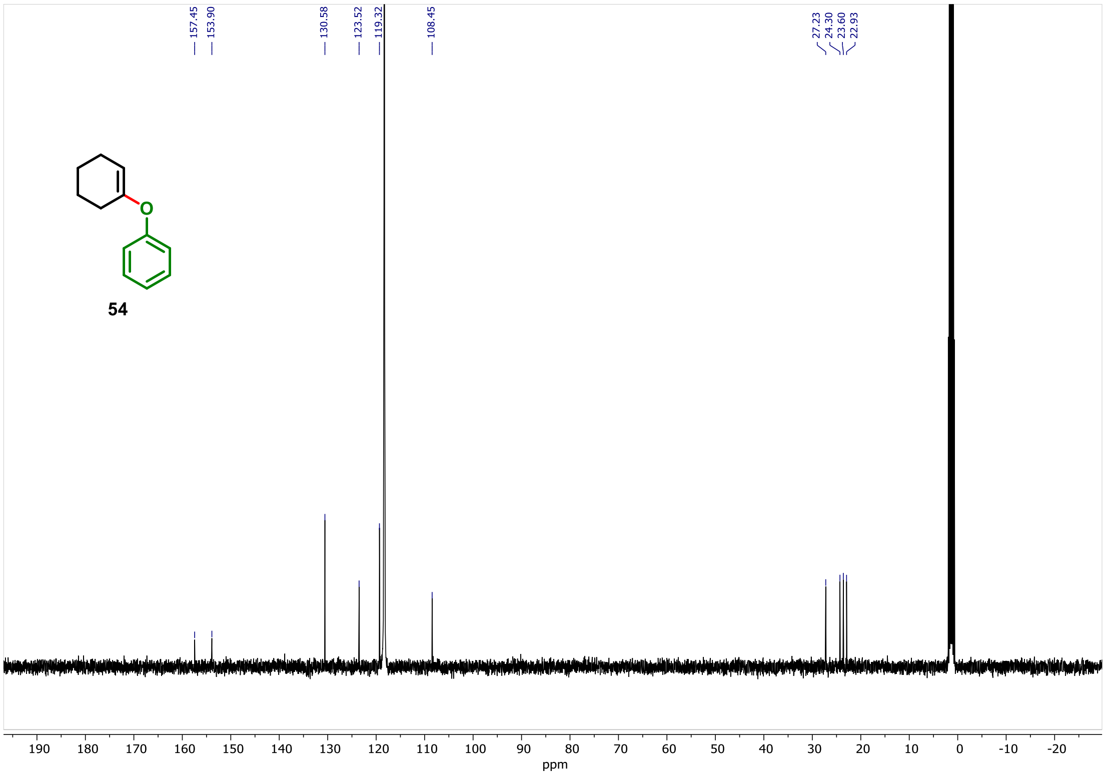

$^{1}H$ NMR (400 MHz, $CD_3CN$) spectra of compound **55**

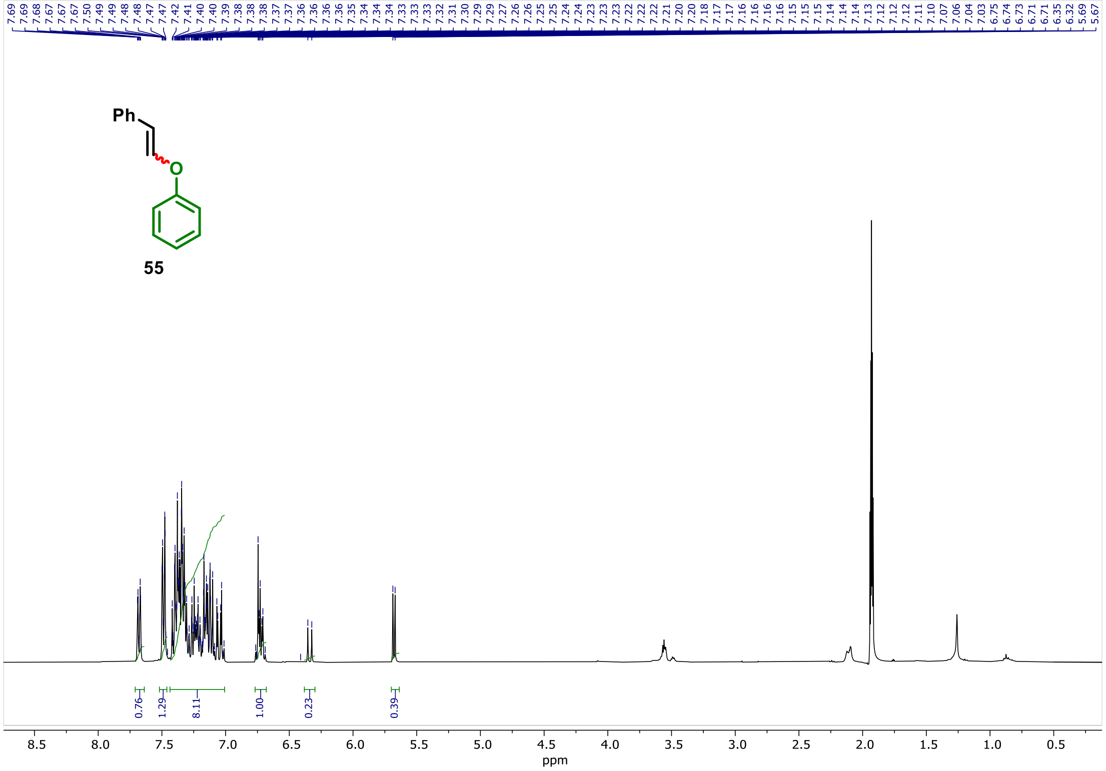


$^{13}C$ NMR (101 MHz, $CD_3CN$) spectra of compound **55**

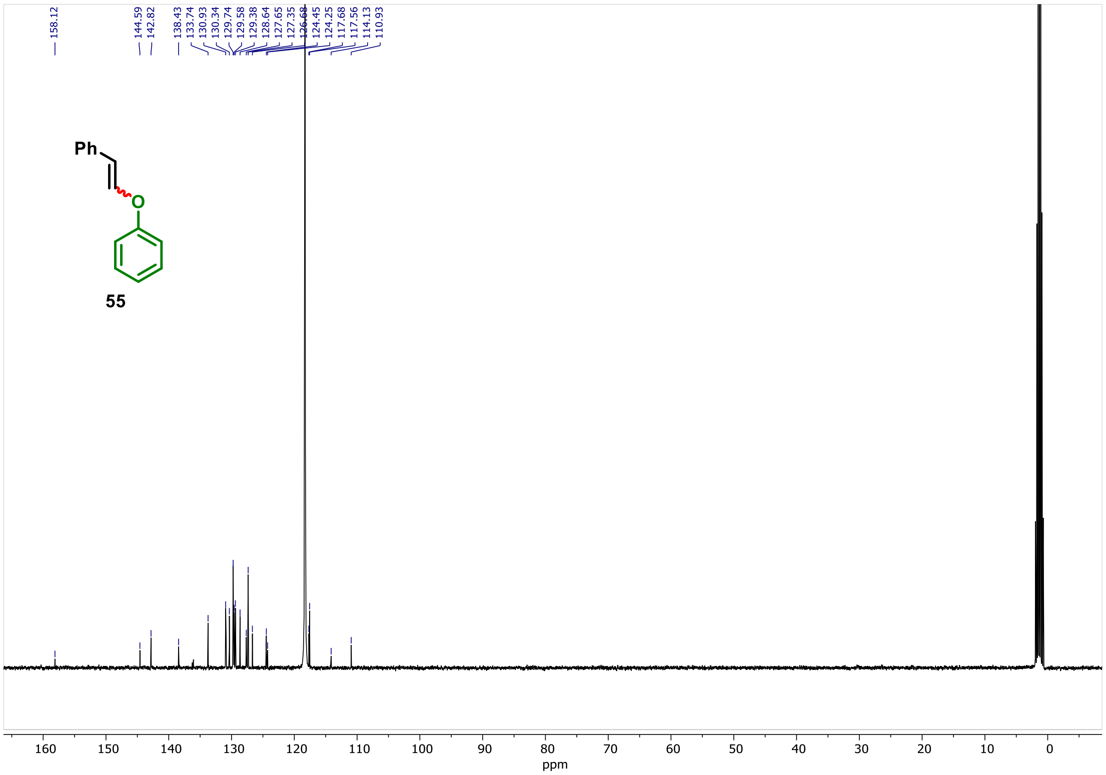

$^{1}H$ NMR (400 MHz, $CD_3CN$) spectra of compound **56**

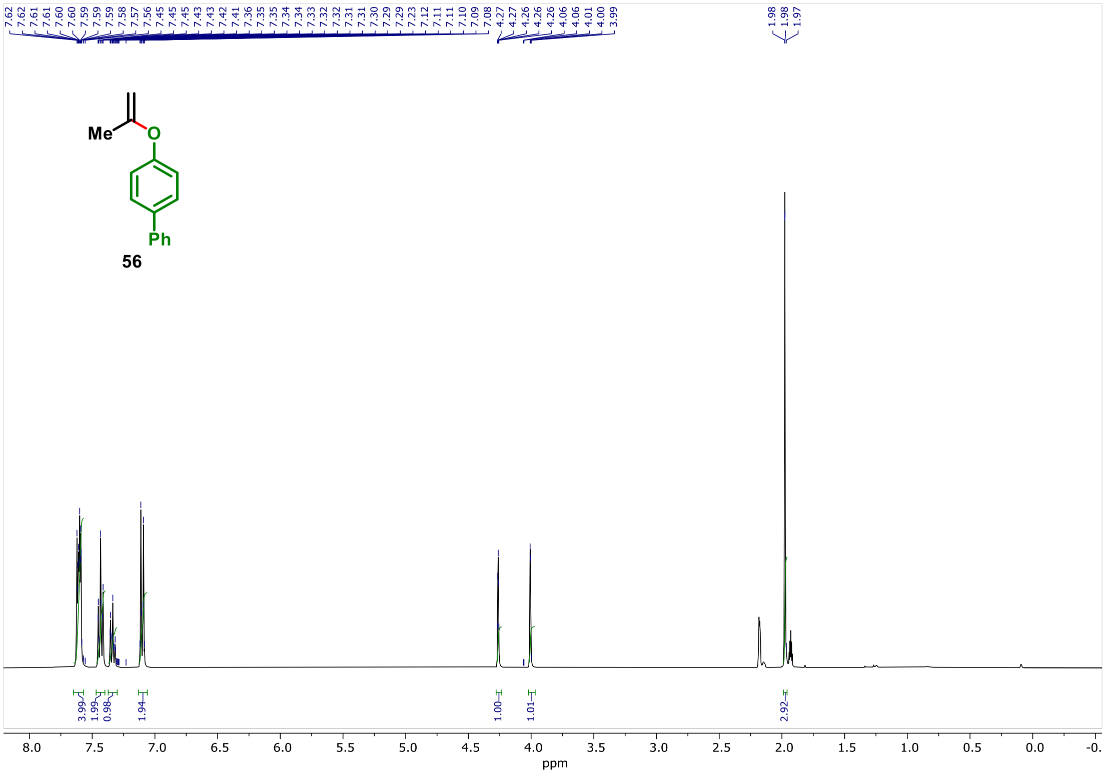

$^{13}C$ NMR (101 MHz, $CD_3CN$) spectra of compound **56**

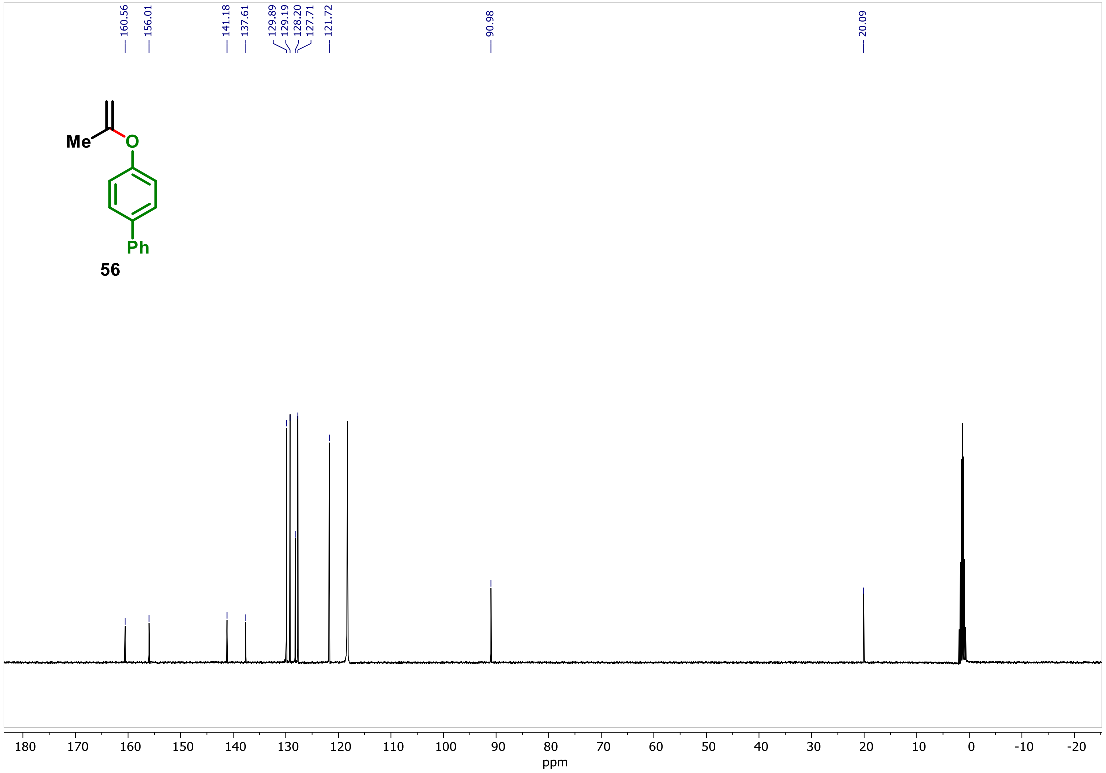

$^{1}H$ NMR (400 MHz, $CD_3CN$) spectra of compound **57**

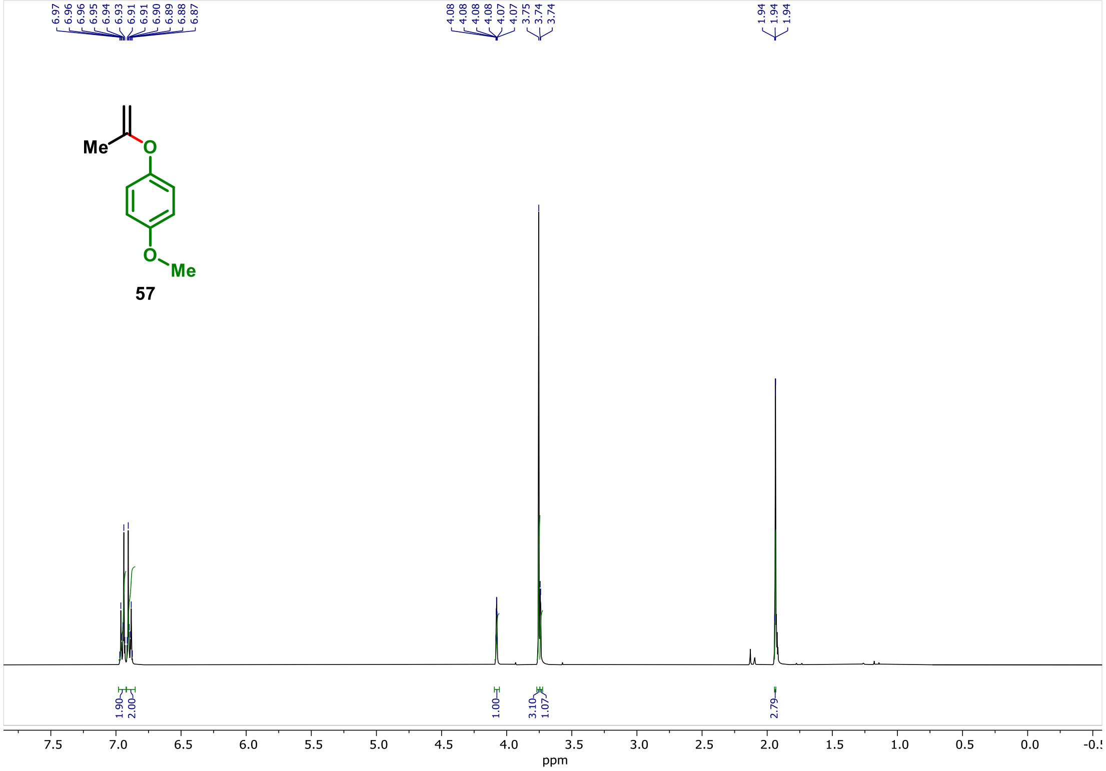


$^{13}C$ NMR (101 MHz, $CD_3CN$) spectra of compound **57**

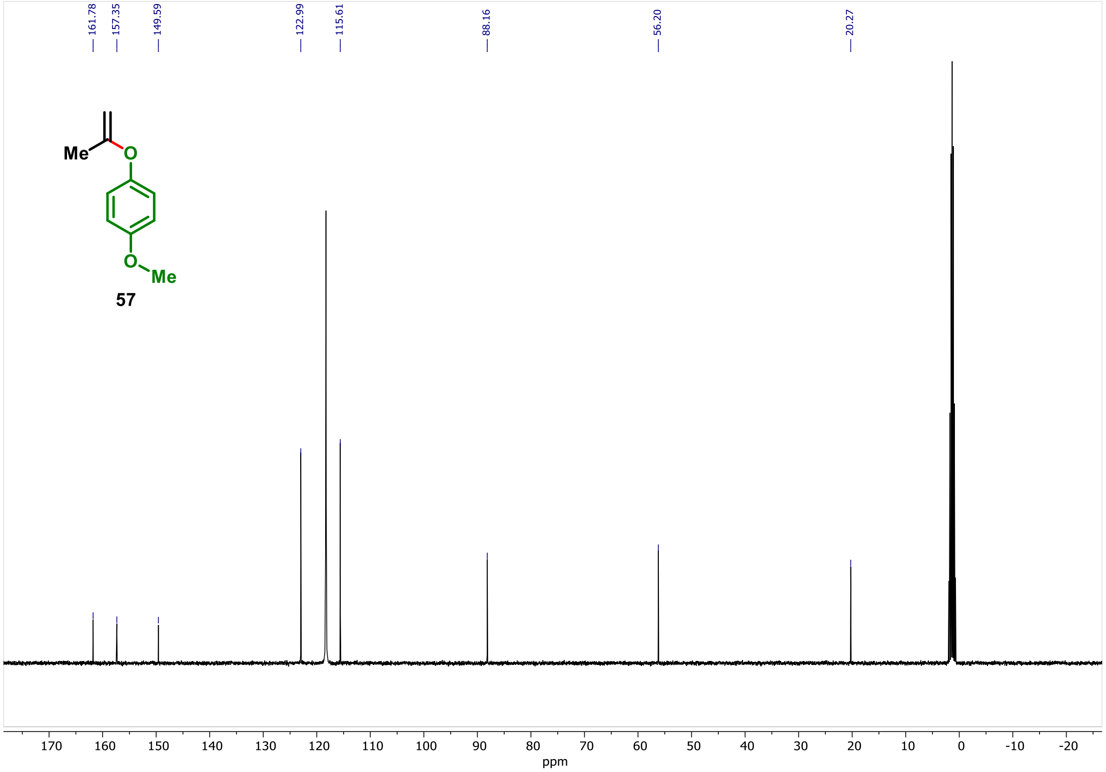

$^{1}H$ NMR (400 MHz, $CD_3CN$) spectra of compound **58**

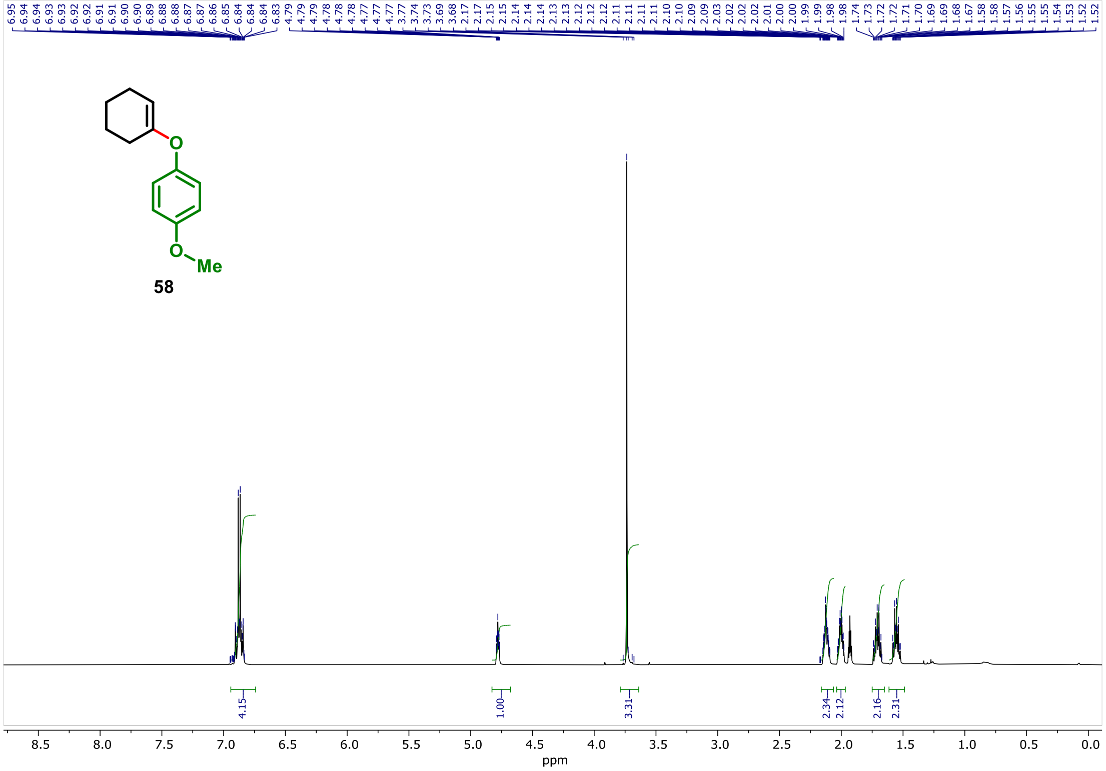


$^{13}C$ NMR (101 MHz, $CD_3CN$) spectra of compound **58**

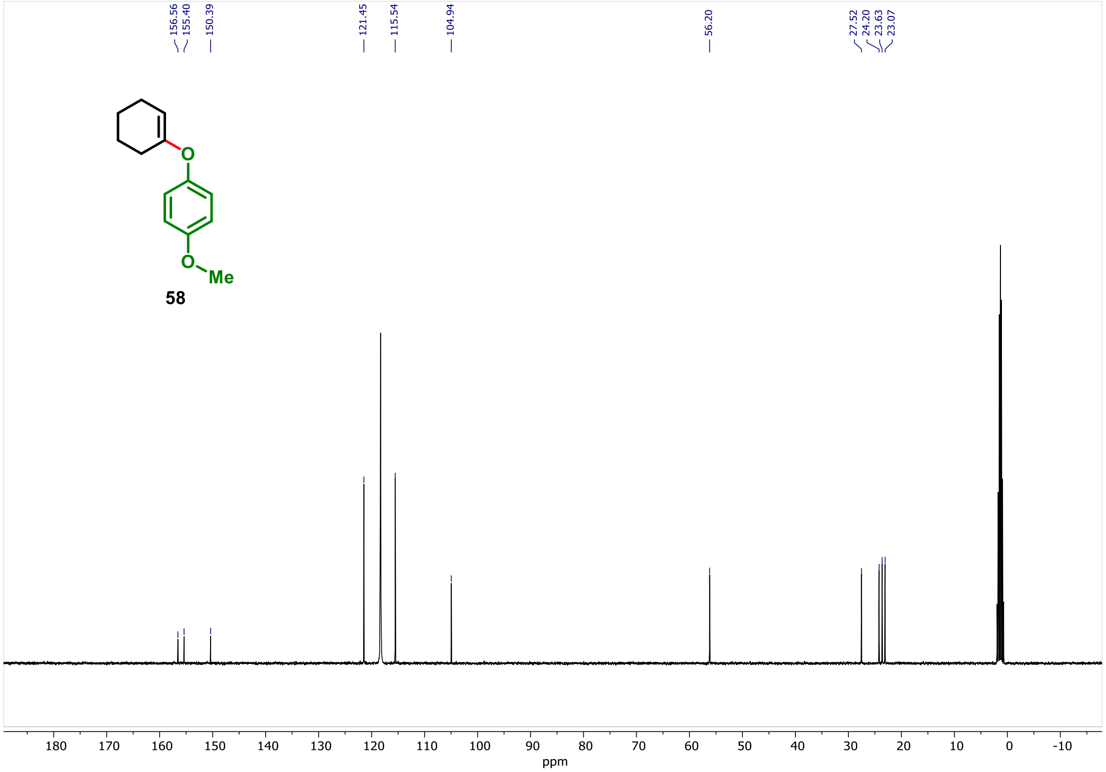

$^{1}$H NMR (400 MHz, $CD_3CN$) spectra of compound **59**

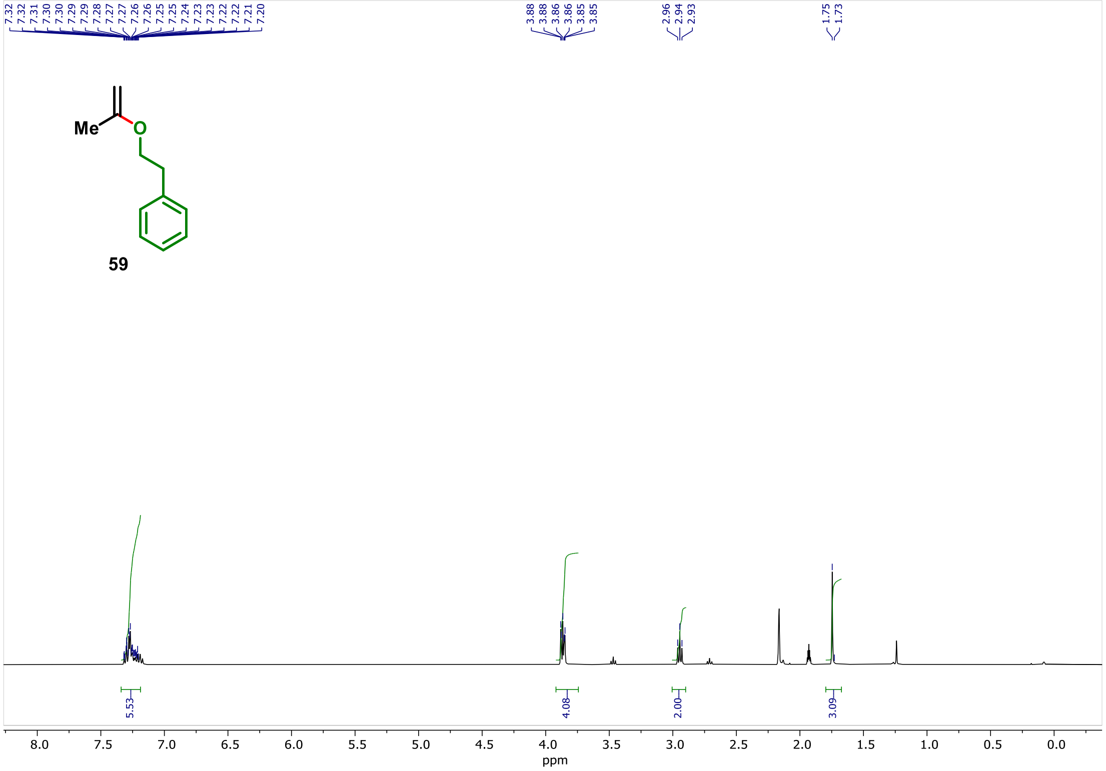


$^{13}$C NMR (101 MHz, $CD_3CN$) spectra of compound **59**

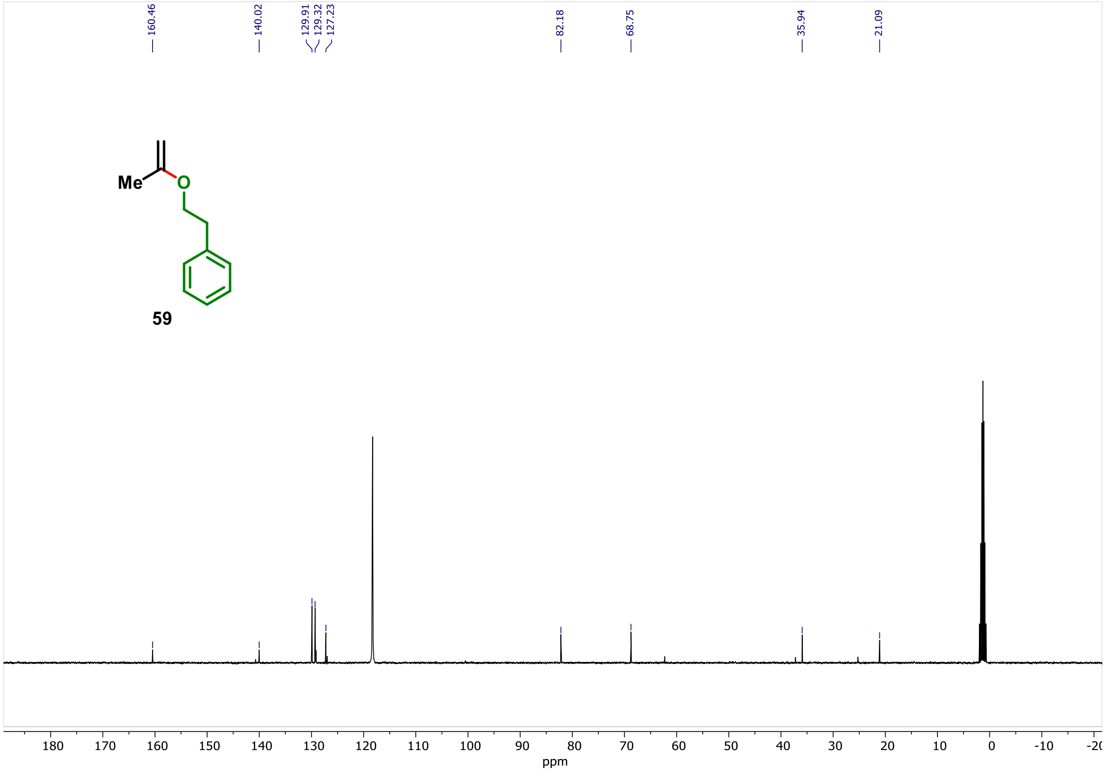

$^{1}$H NMR (400 MHz, $CD_3CN$) spectra of compound **60**

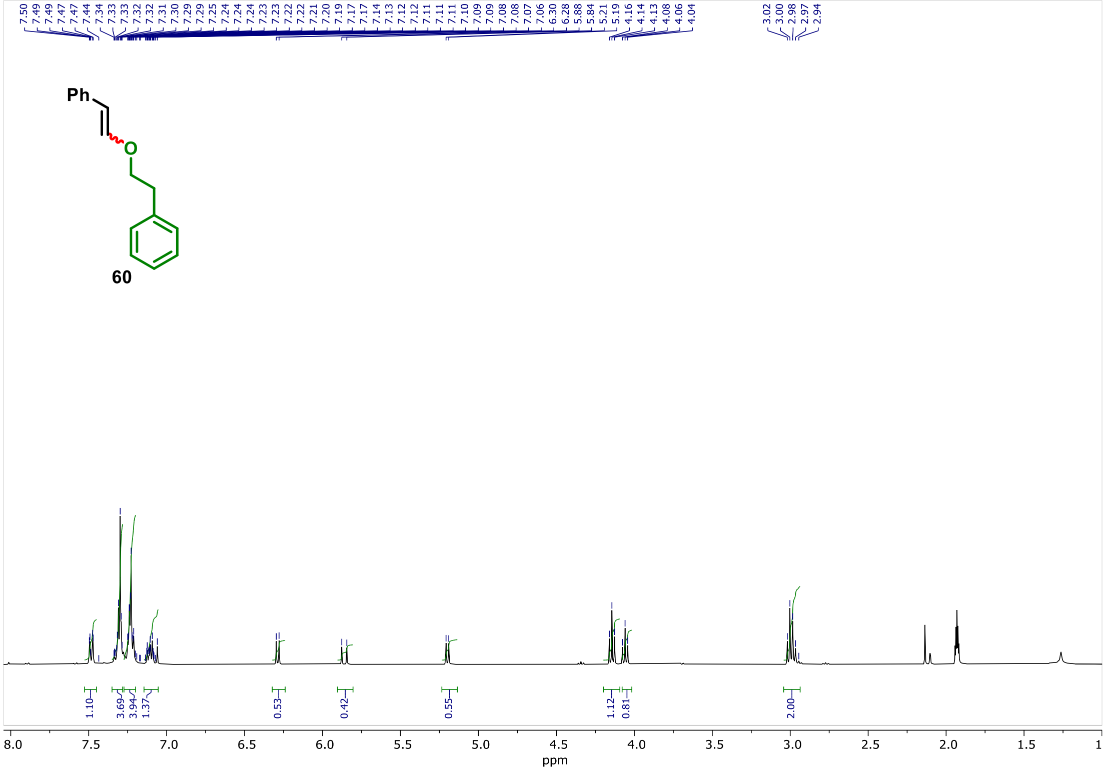


$^{13}$C NMR (101 MHz, $CD_3CN$) spectra of compound **60**

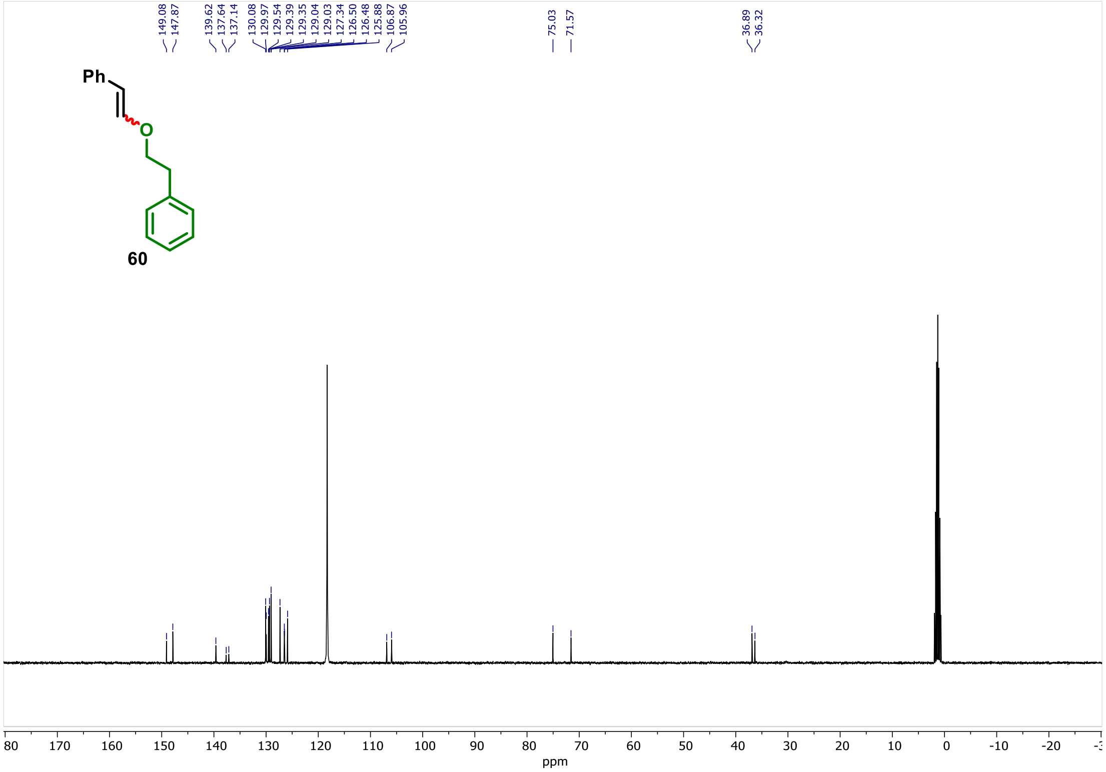

$^{1}$H NMR (400 MHz, $CD_3CN$) spectra of compound **61**

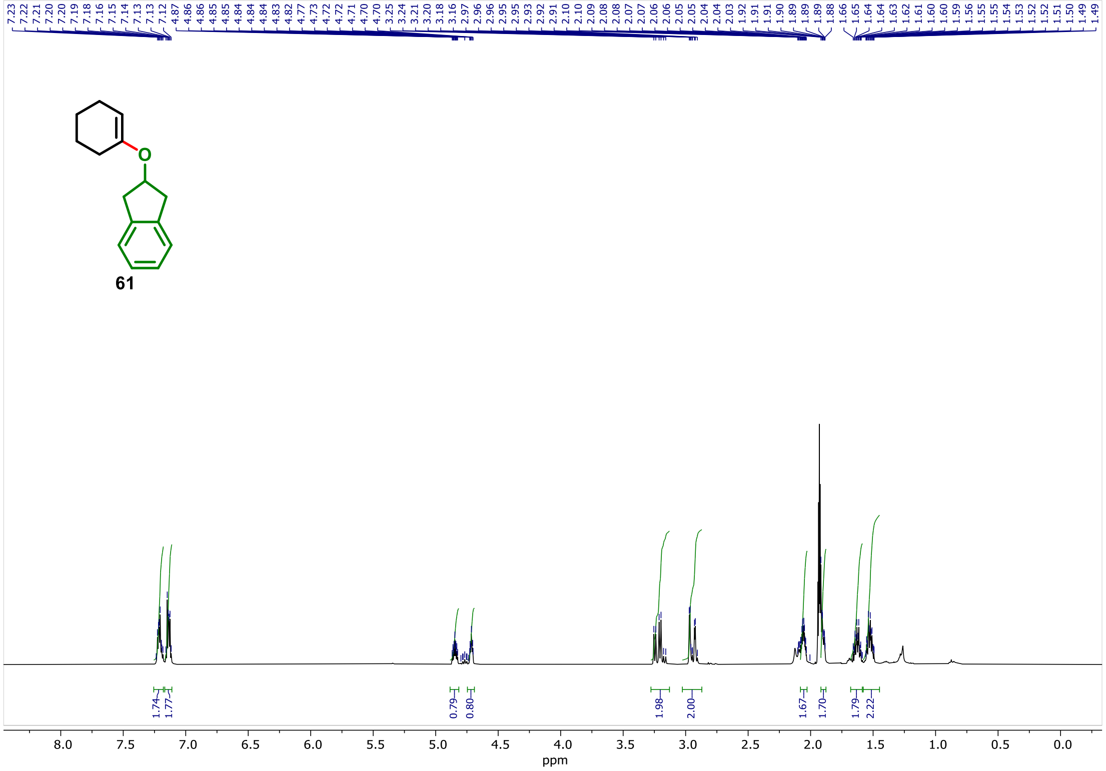


$^{13}$C NMR (101 MHz, $CD_3CN$) spectra of compound **61**

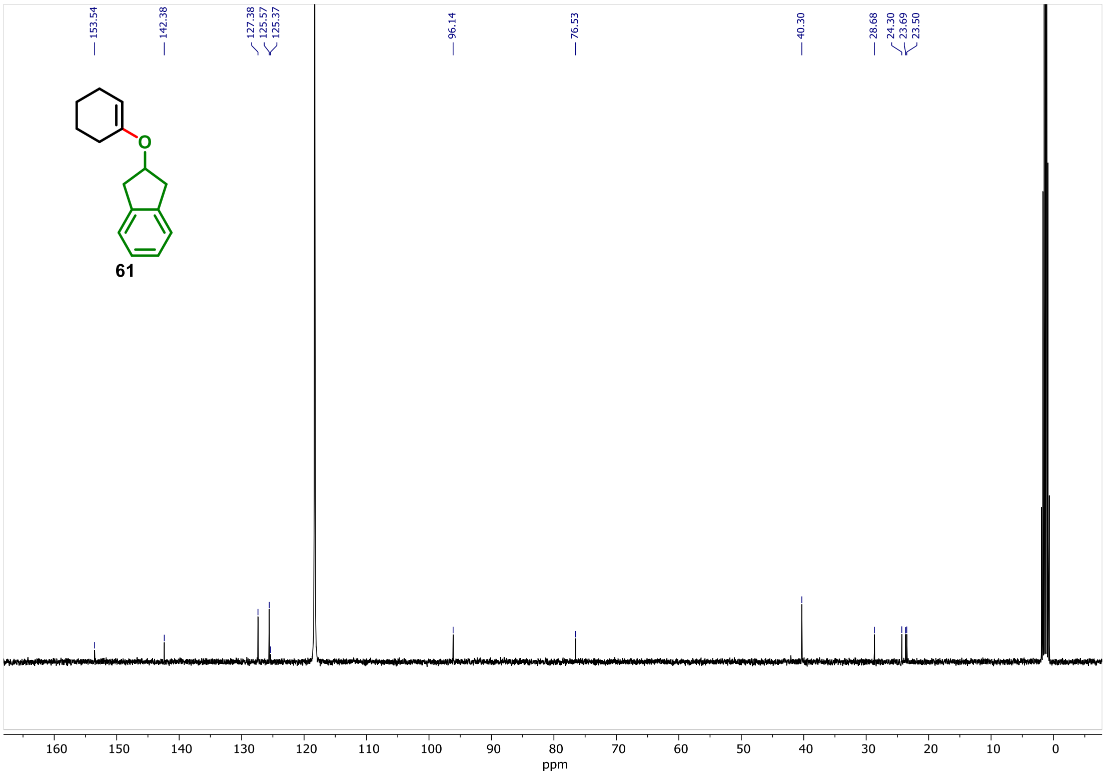

$^{1}$H NMR (400 MHz, $CD_3CN$) spectra of compound **62**

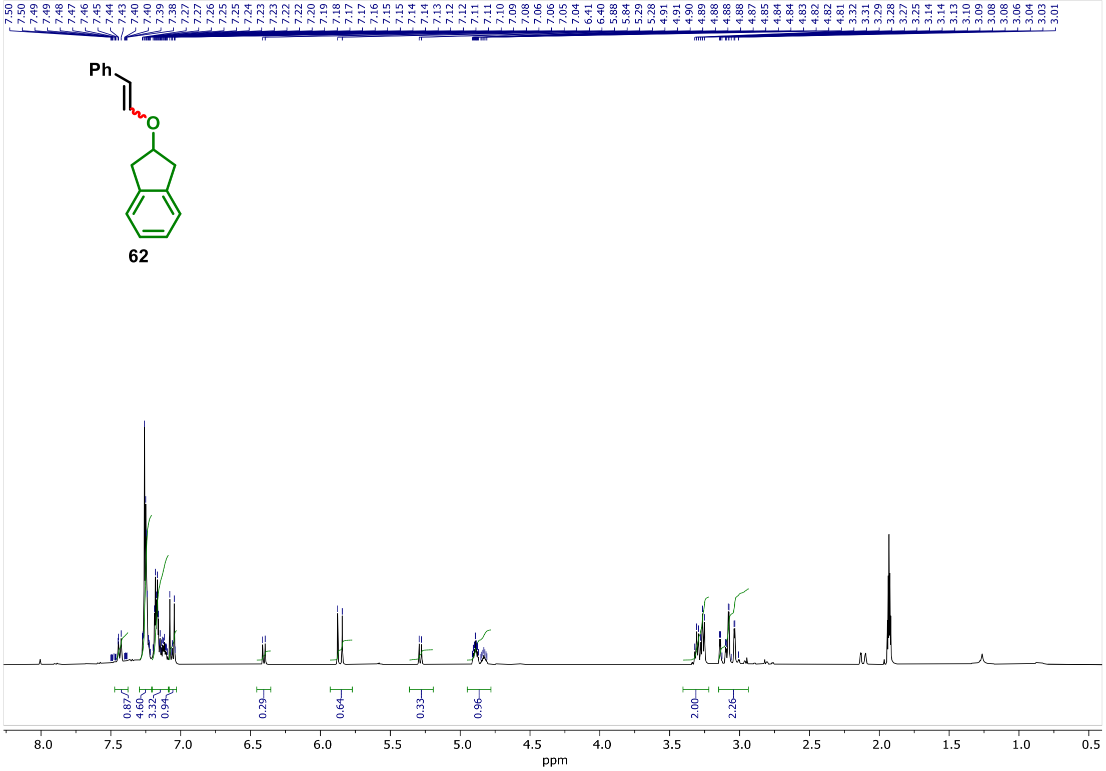


$^{13}$C NMR (101 MHz, $CD_3CN$) spectra of compound **62**

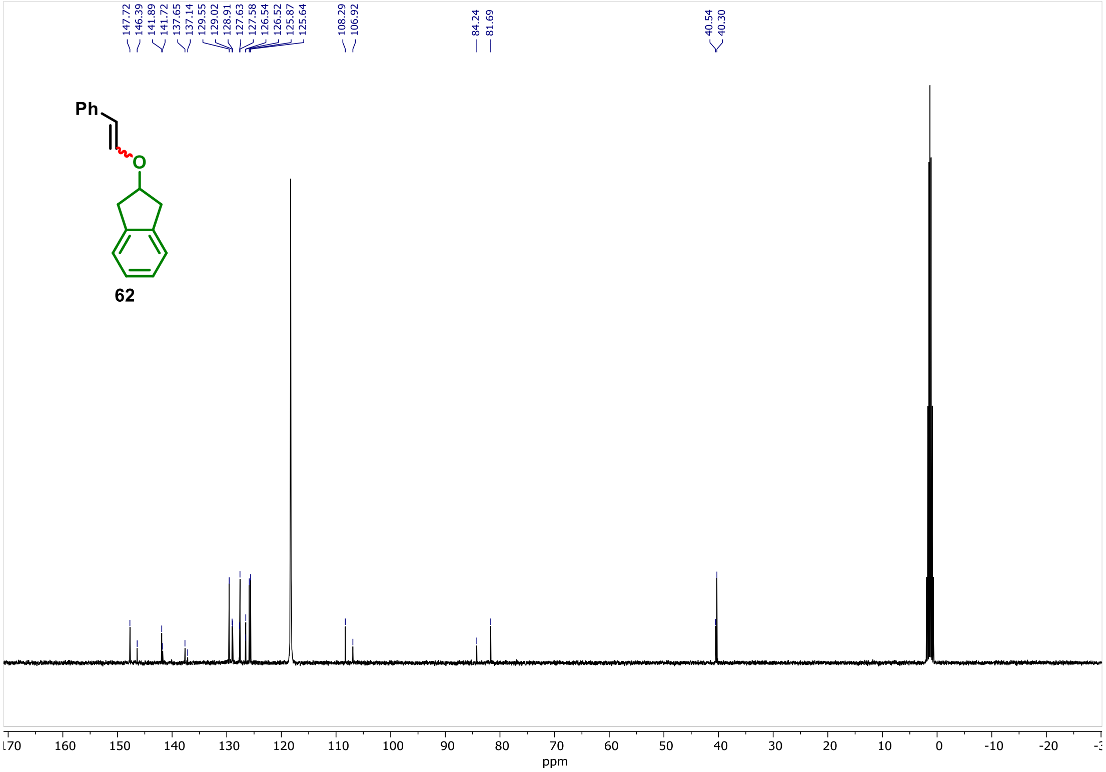

$^{1}H$ NMR (400 MHz, $CDCl_3$) spectra of compound **63**

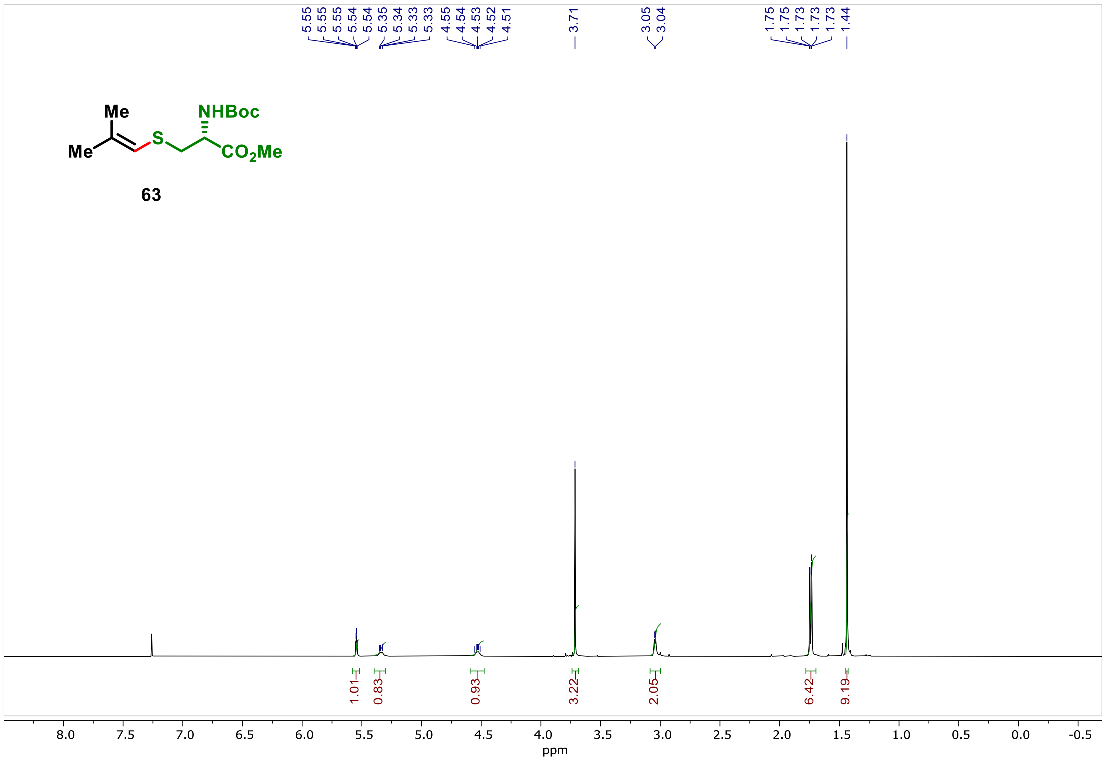


$^{13}C$ NMR (101 MHz, $CDCl_3$) spectra of compound **63**

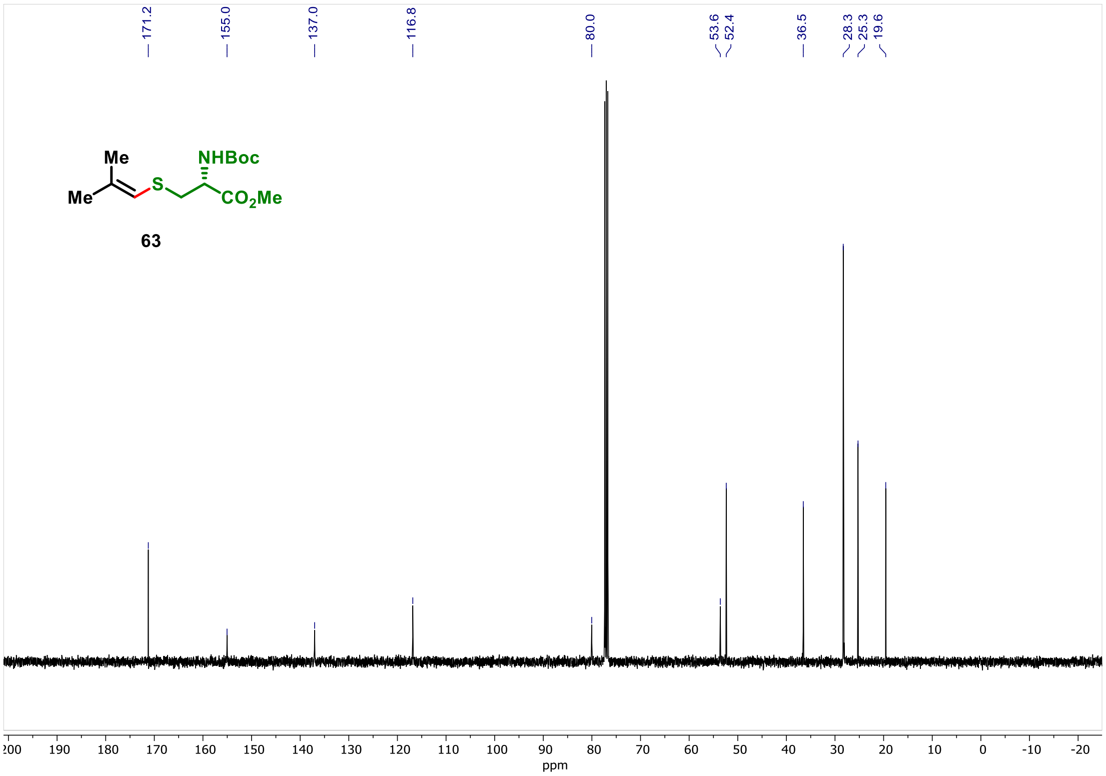

$^{1}$H NMR (400 MHz, $CDCl_3$) spectra of compound **64**

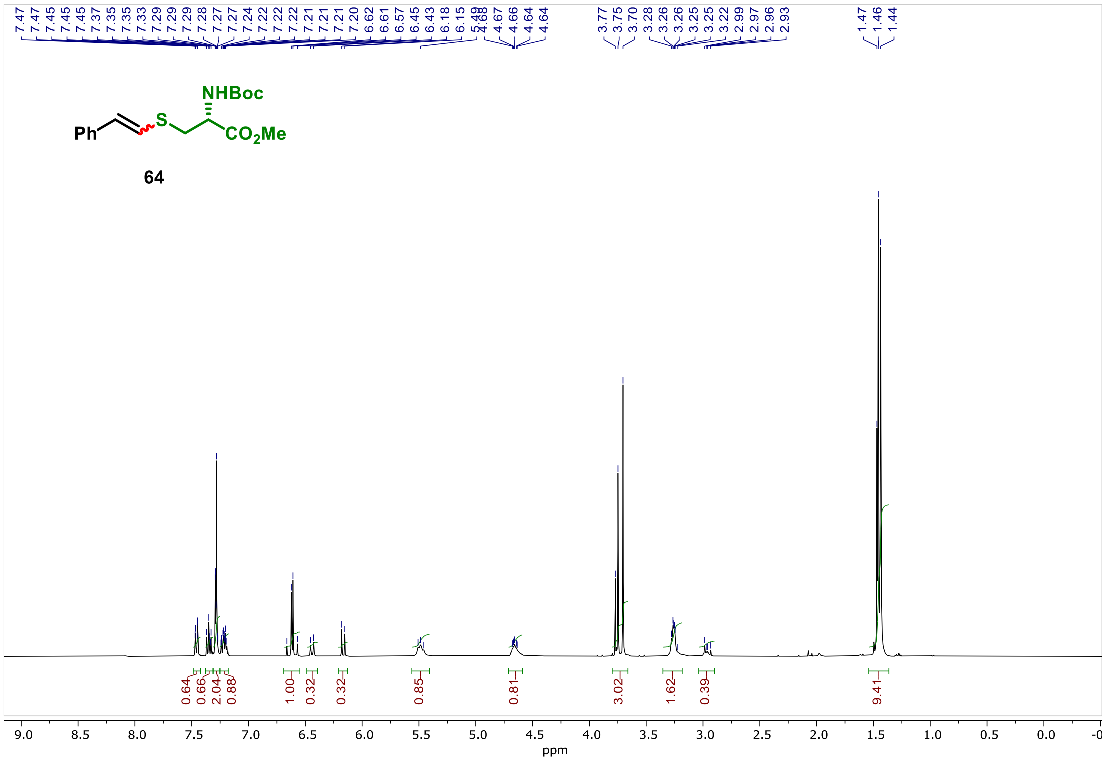

$^{13}$C NMR (101 MHz, $CDCl_3$) spectra of compound **64**

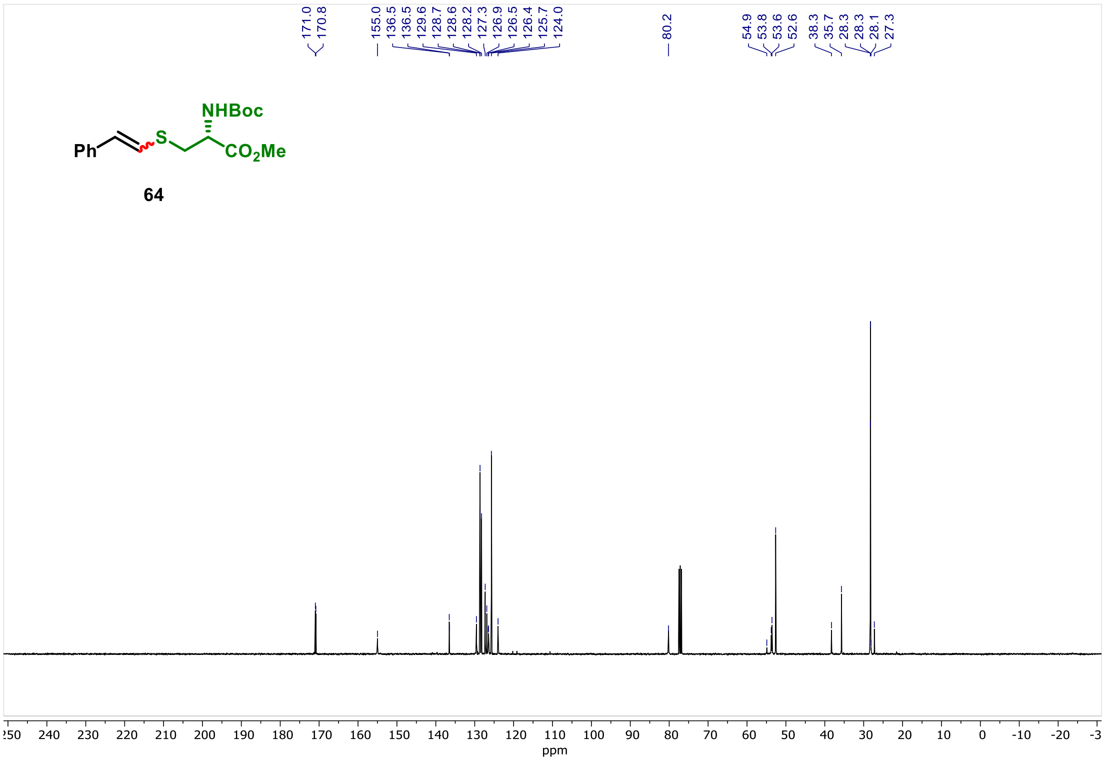

$^{1}$H NMR (400 MHz, $CDCl_3$) spectra of compound **65**

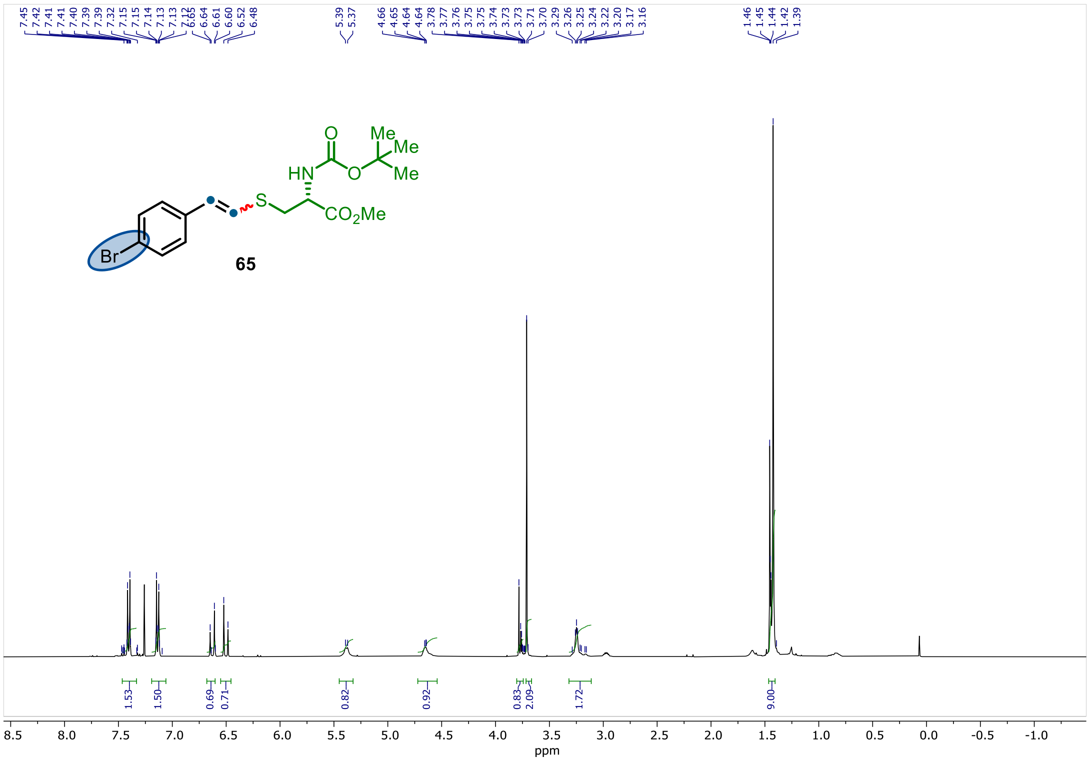


$^{13}$C NMR (101 MHz, $CDCl_3$) spectra of compound **65**

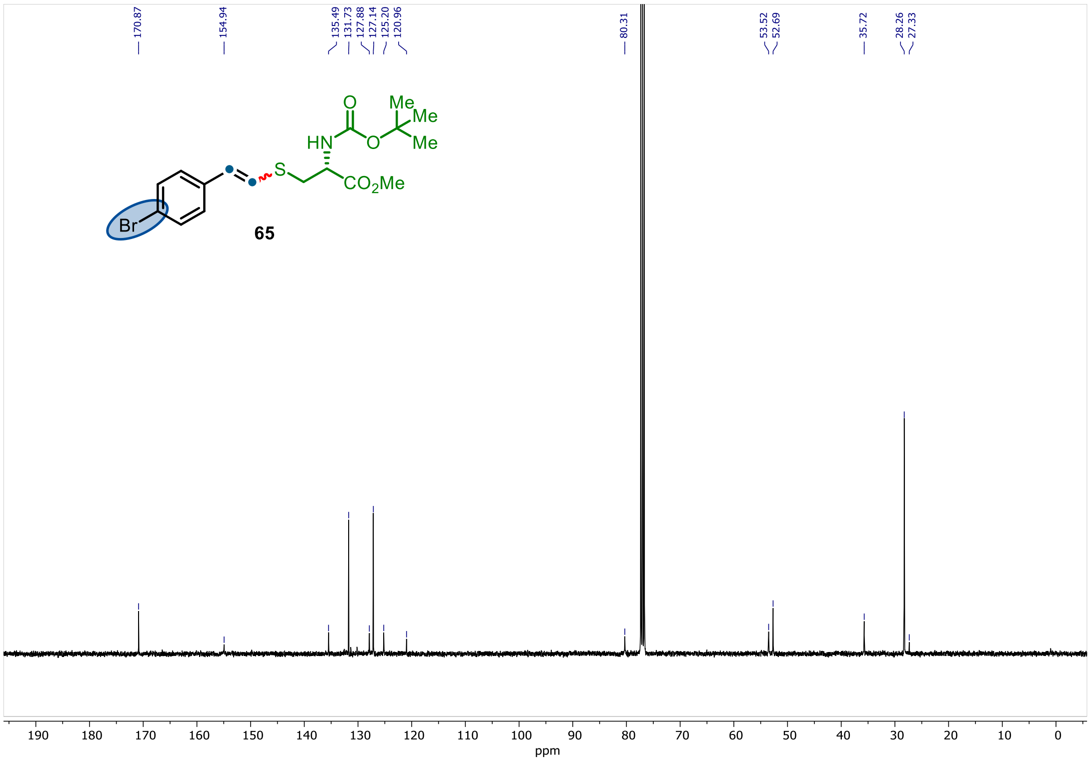

$^{1}$H NMR (400 MHz, DMSO) spectra of compound **66**

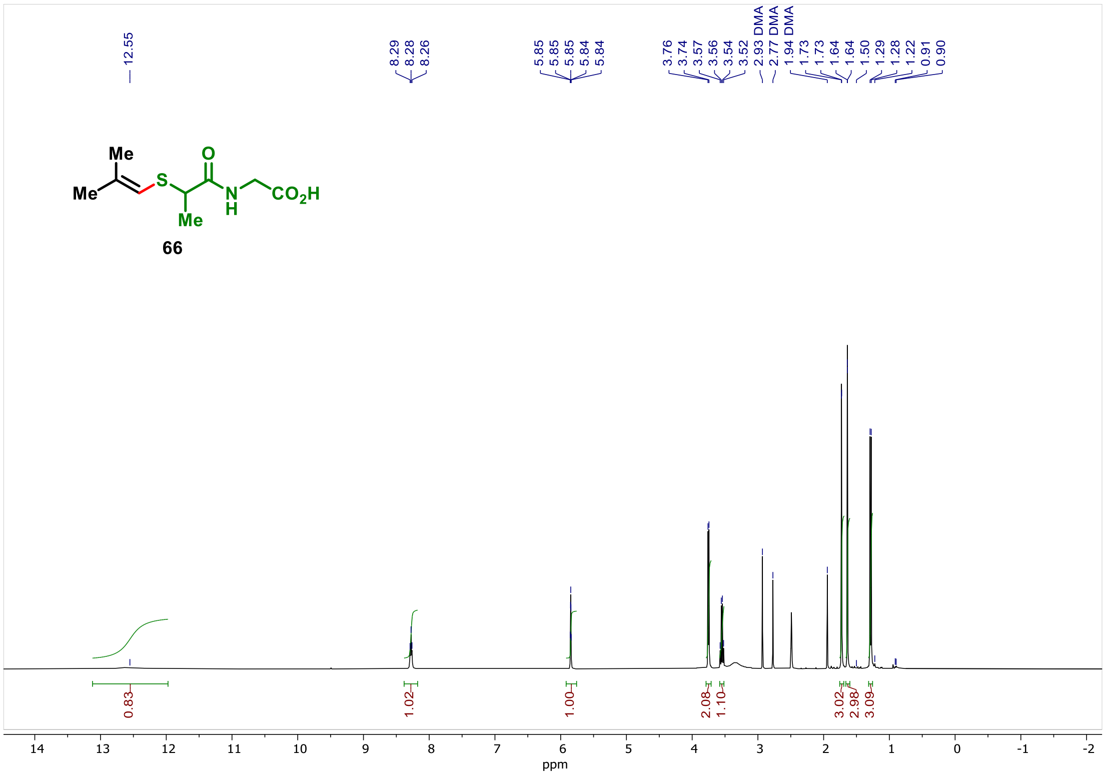


$^{13}$C NMR (101 MHz, DMSO) spectra of compound **66**

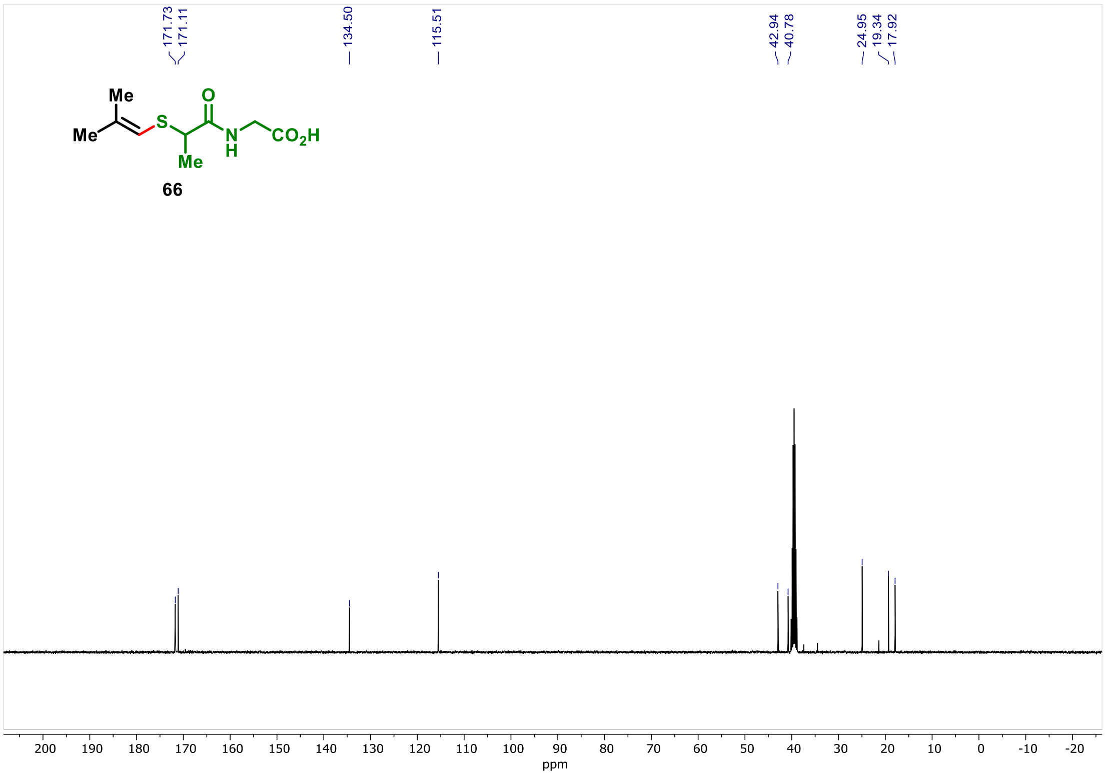

$^{1}$H NMR (400 MHz, $CDCl_3$) spectra of compound **67**

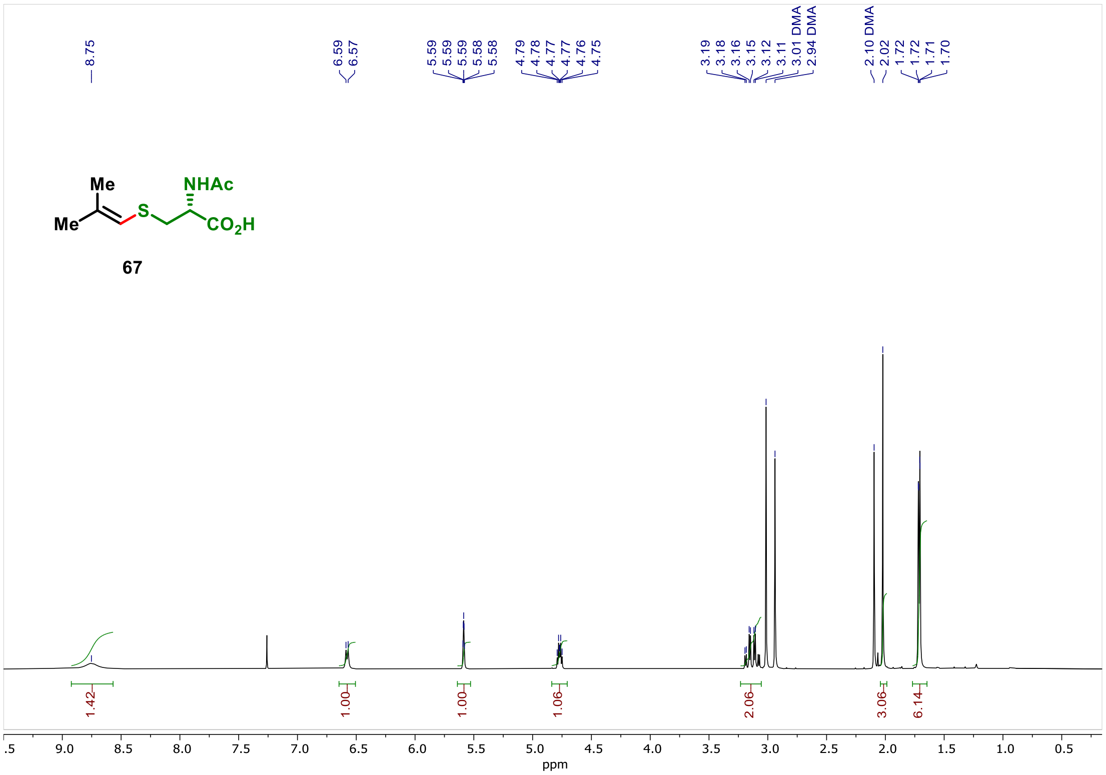


$^{13}$C NMR (101 MHz, $CDCl_3$) spectra of compound **67**

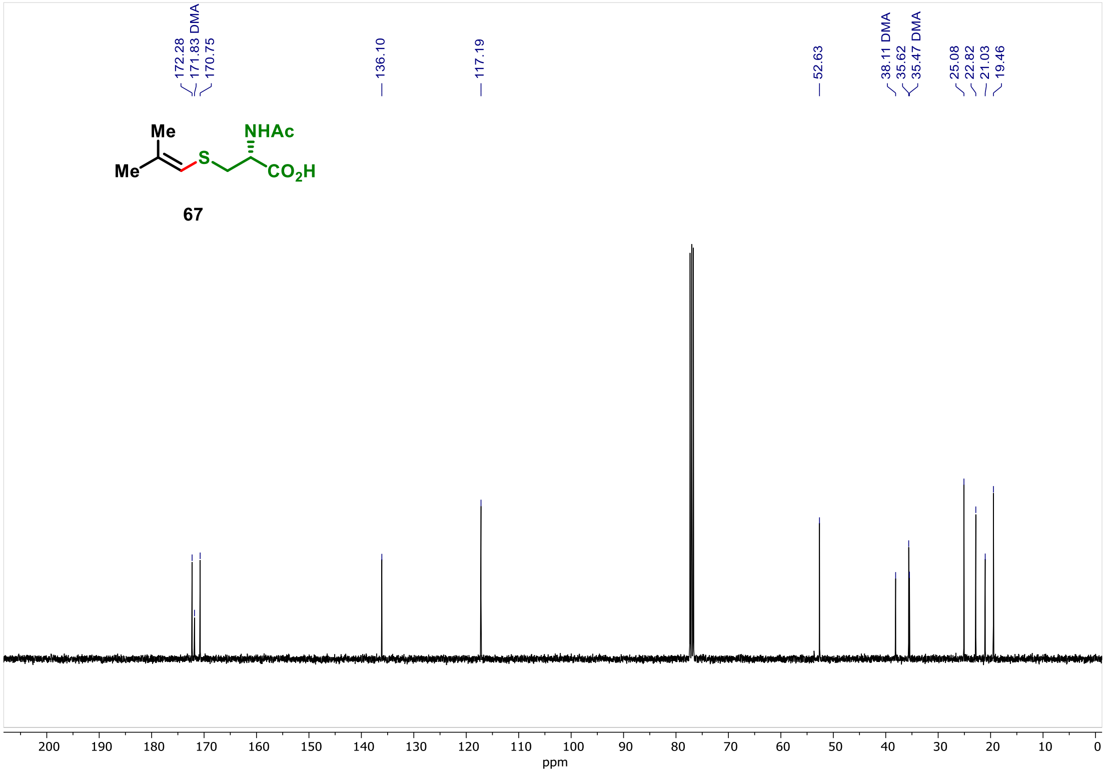

$^{1}H$ NMR (400 MHz, $CDCl_3$) spectra of compound **68**

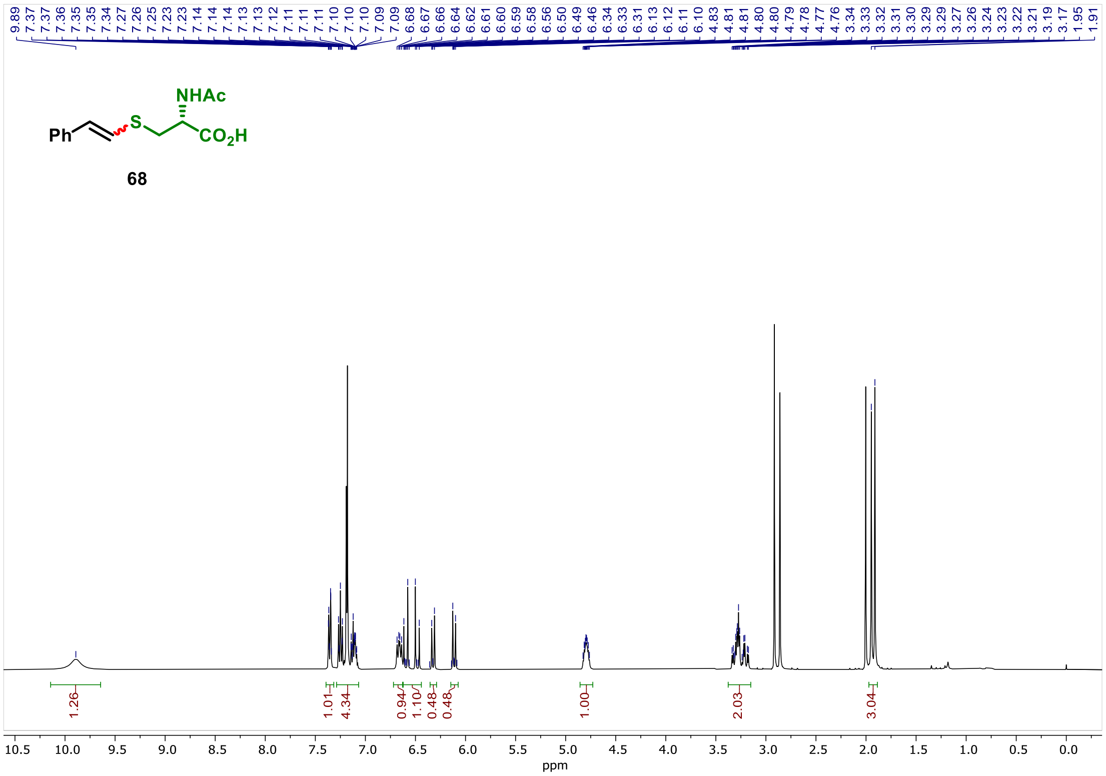

$^{13}C$ NMR (101 MHz, $CDCl_3$) spectra of compound **68**

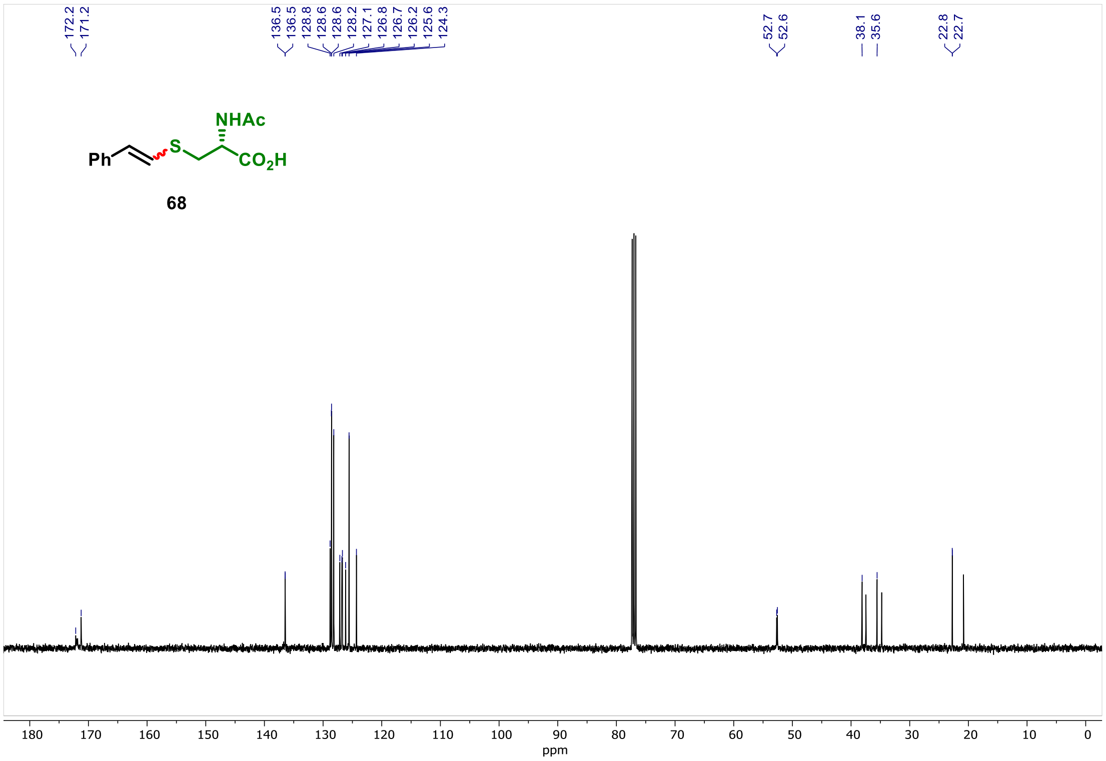

$^{1}$H NMR (400 MHz, $CD_3CN$) spectra of compound **69**

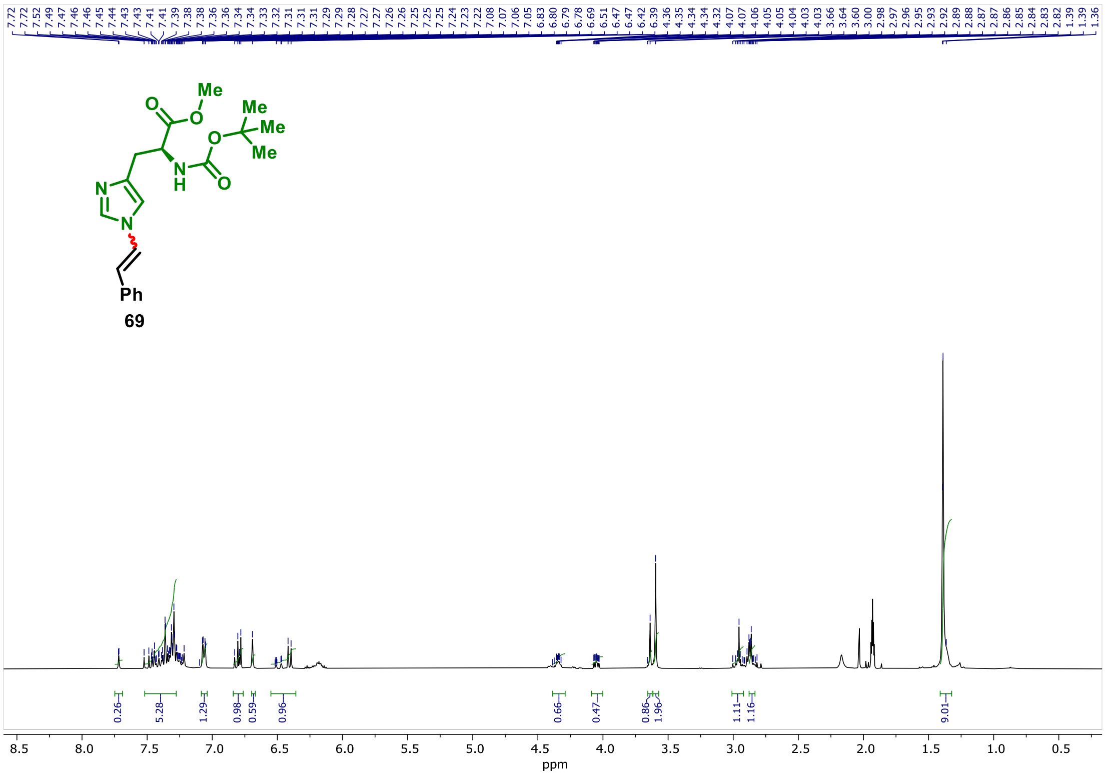


$^{13}$C NMR (101 MHz, $CD_3CN$) spectra of compound **69**

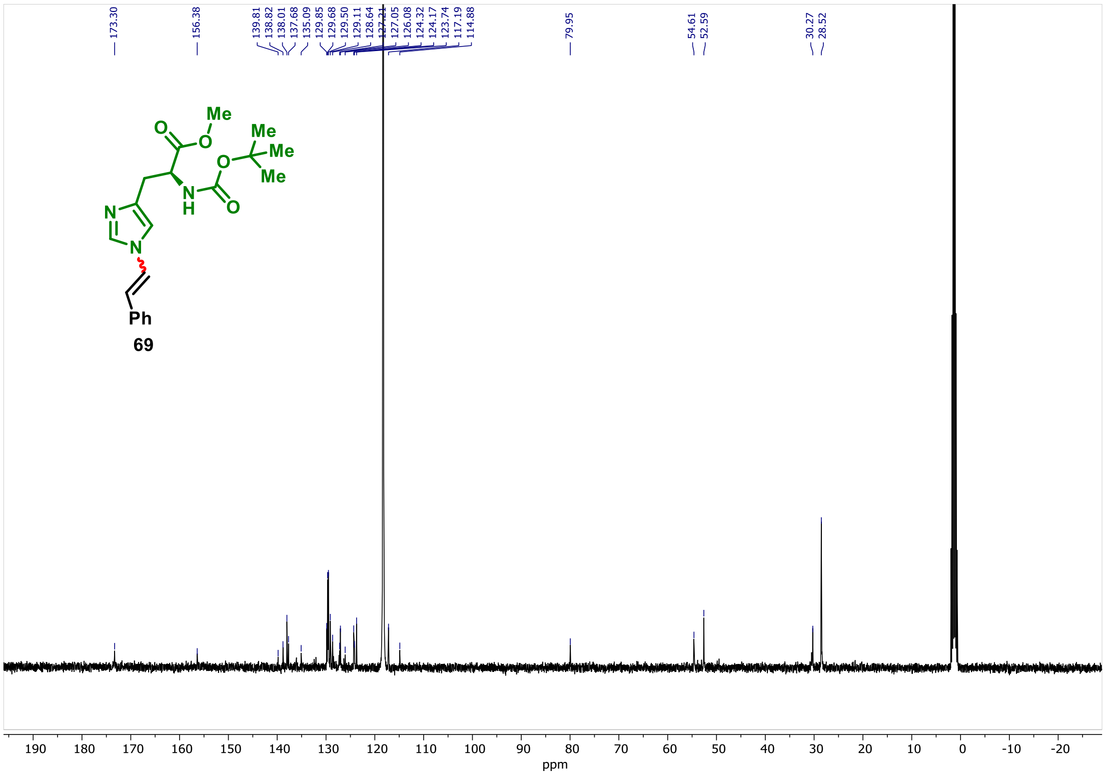

$^{1}H$ NMR (400 MHz, $CD_3CN$) spectra of compound **70**

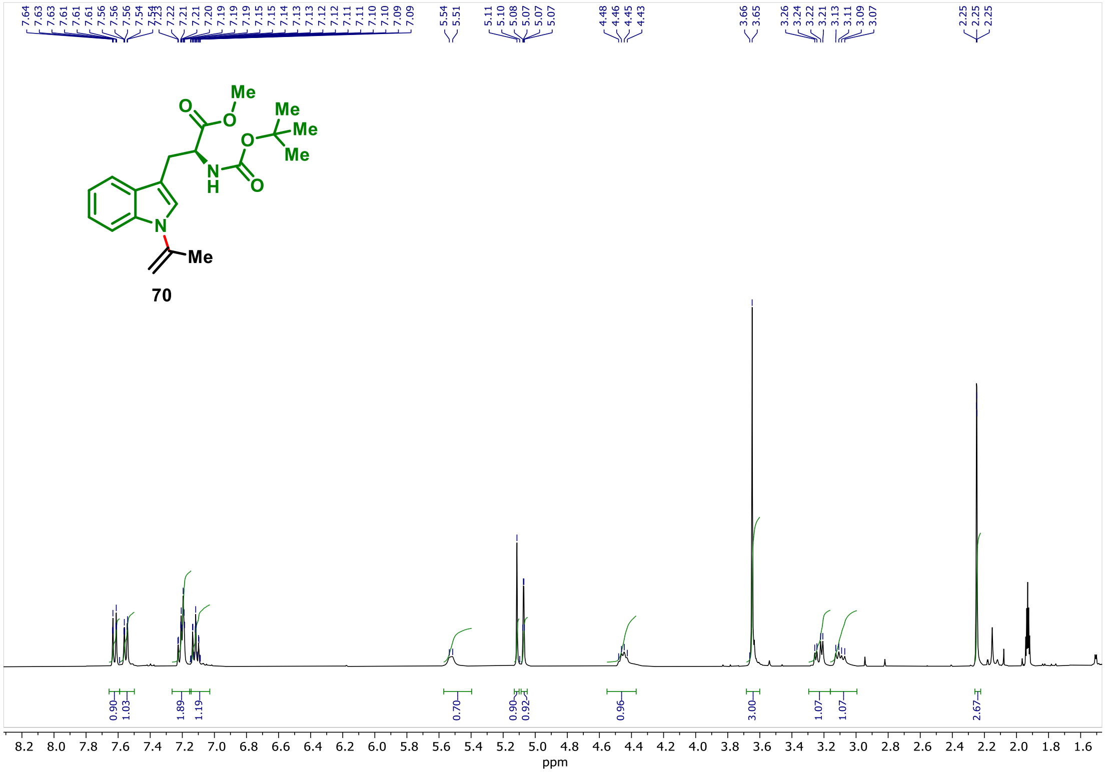

$^{13}C$ NMR (101 MHz, $CD_3CN$) spectra of compound **70**

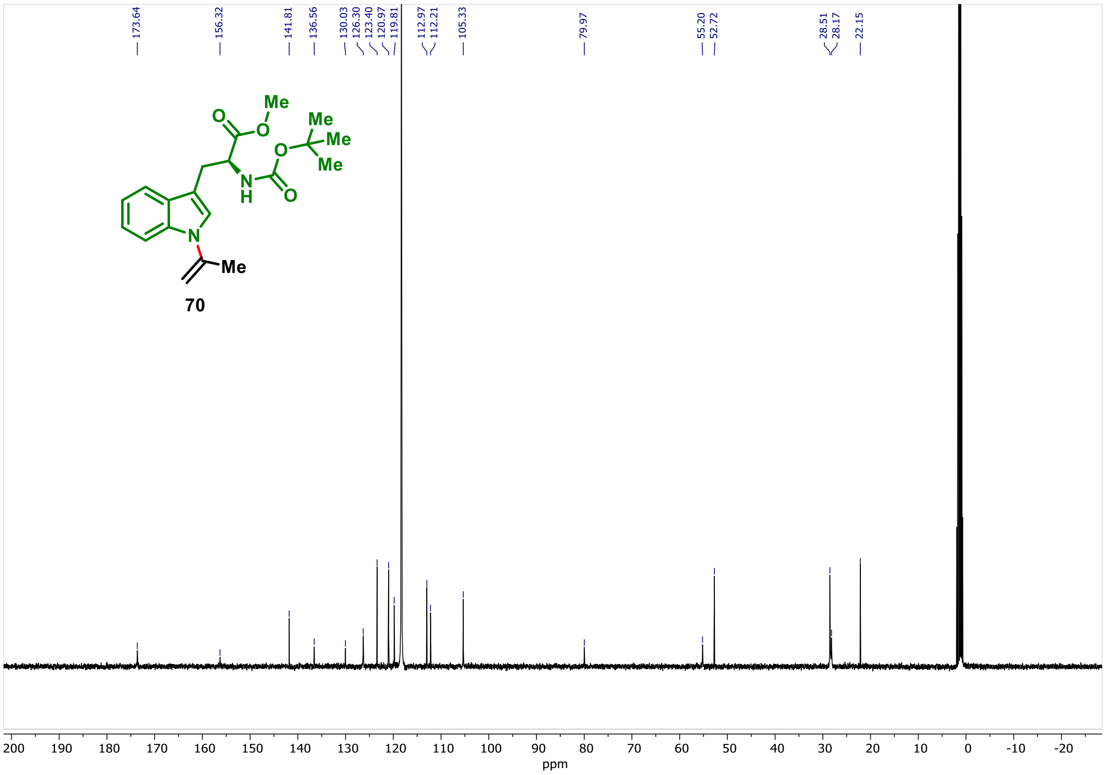

$^{1}$H NMR(400 MHz, $CD_3CN$) spectra of compound **71**

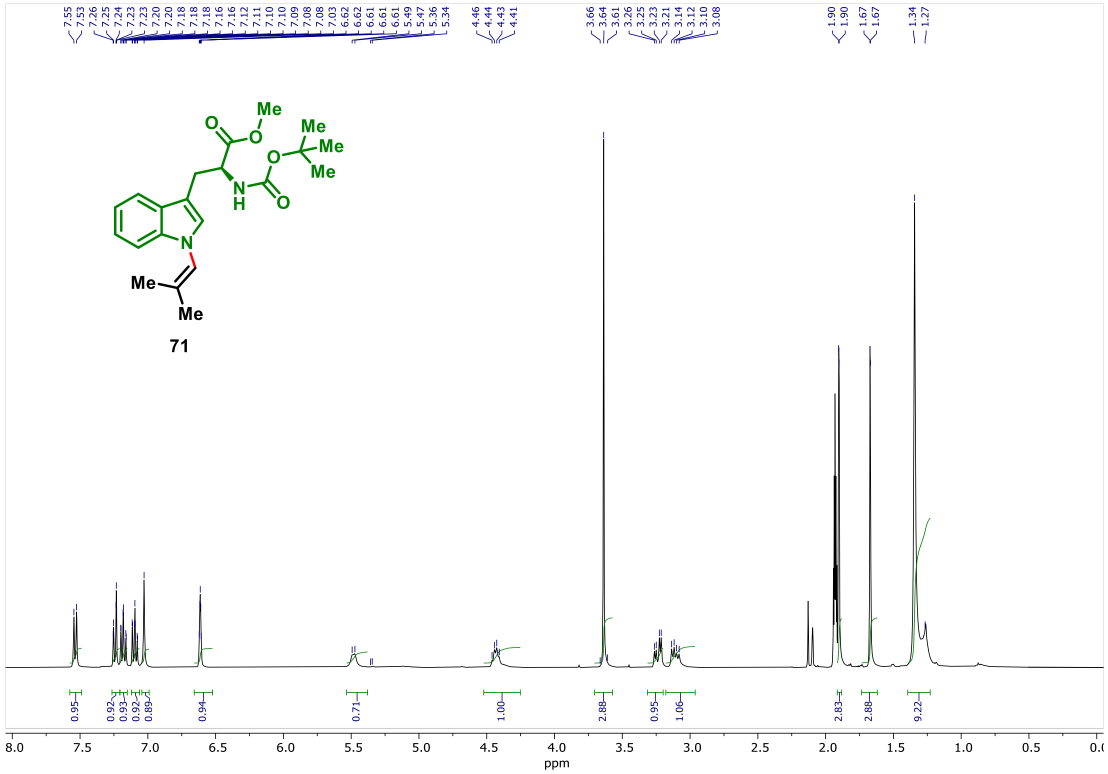

$^{13}$C NMR (101 MHz, $CD_3CN$) spectra of compound **71**

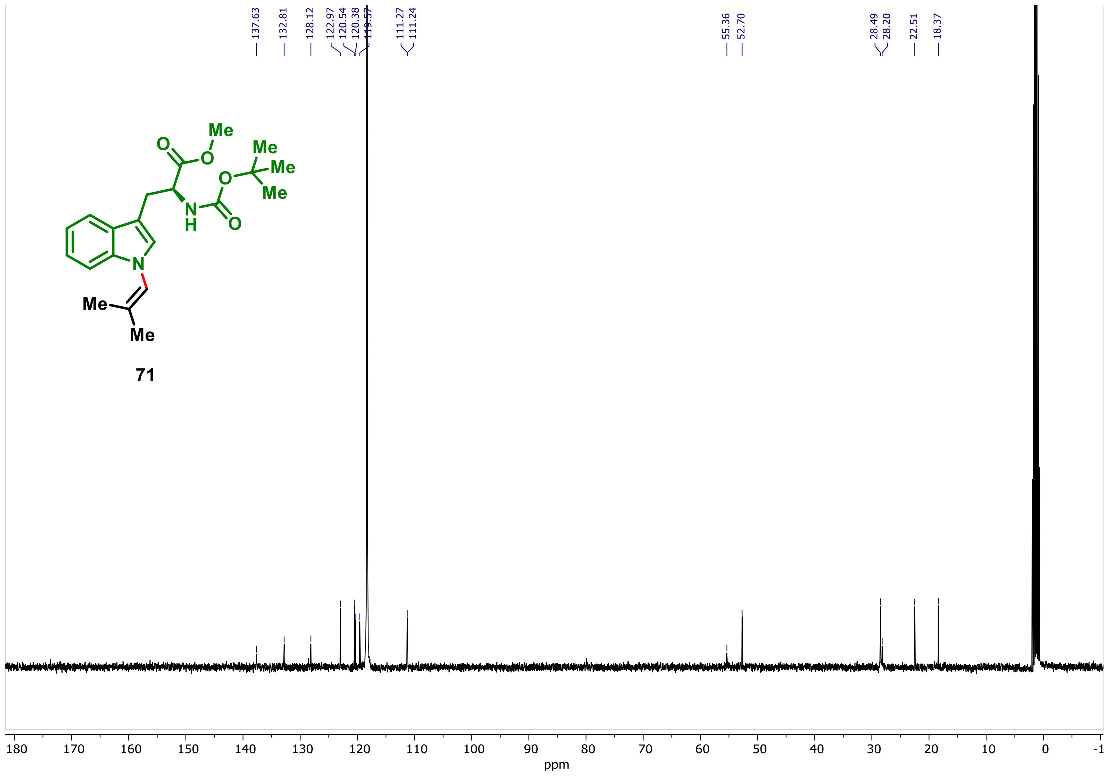

$^{1}$H NMR (400 MHz, $CD_3CN$) spectra of compound **72**

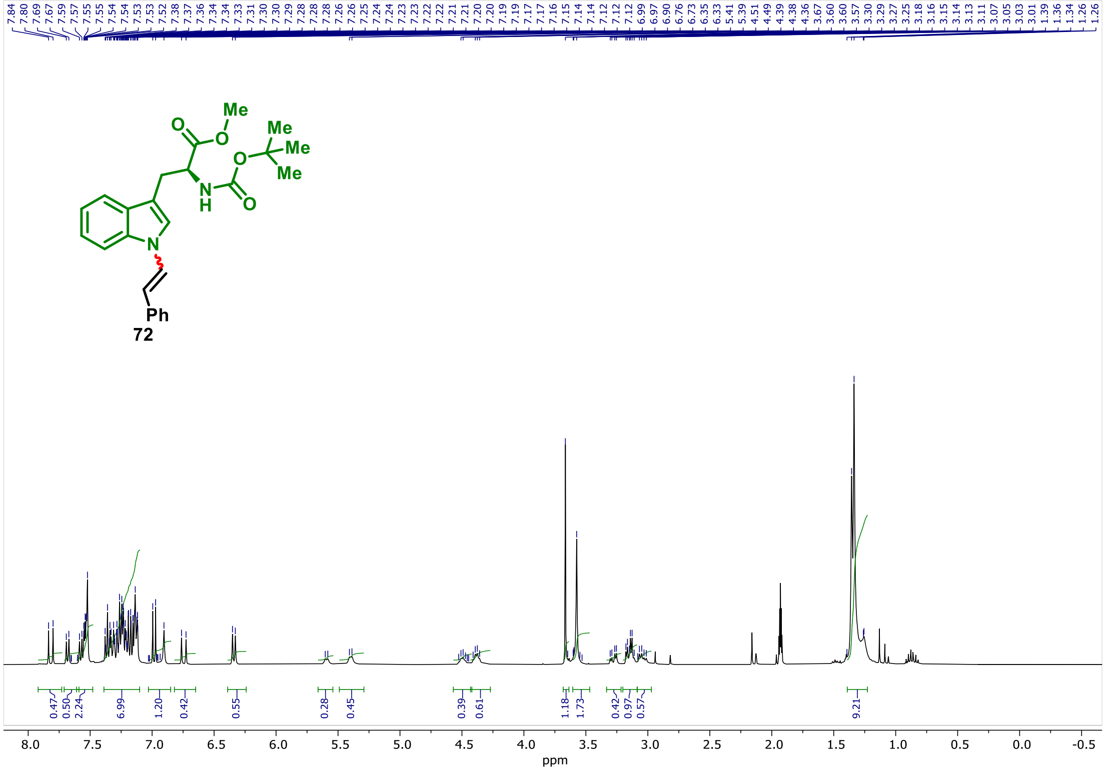


$^{13}$C NMR (101 MHz, $CD_3CN$) spectra of compound **72**

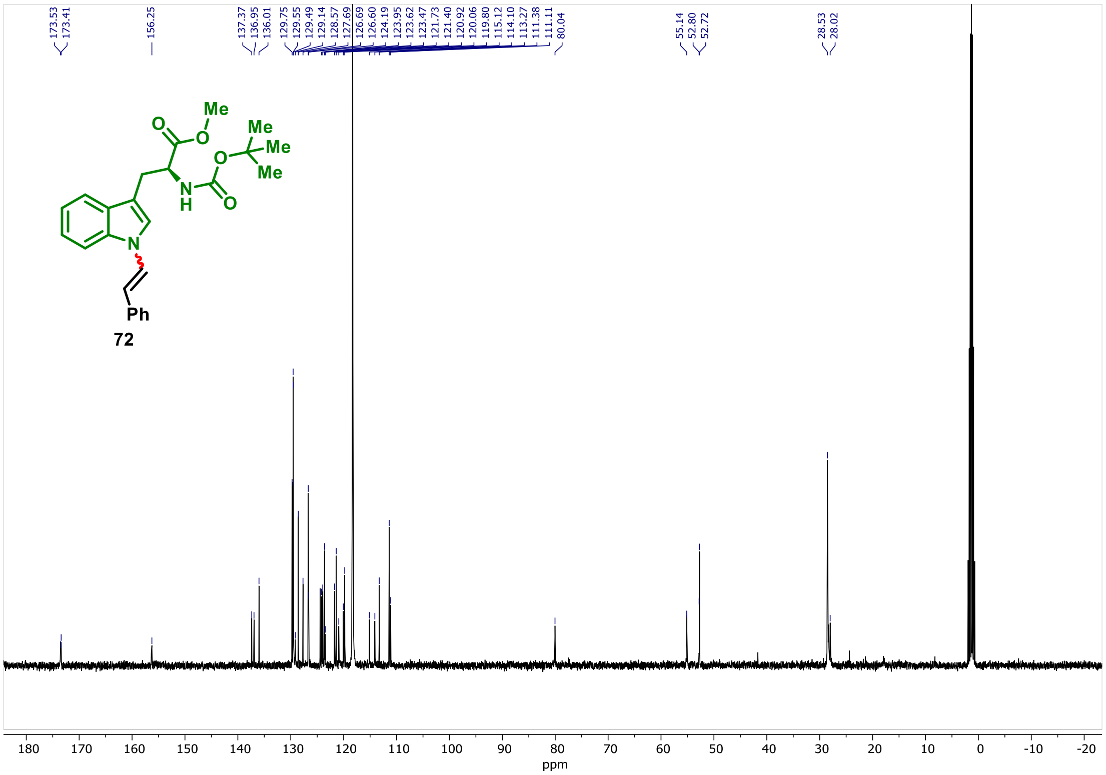

$^{1}$H NMR (400 MHz, $CD_3CN$) spectra of compound **73**

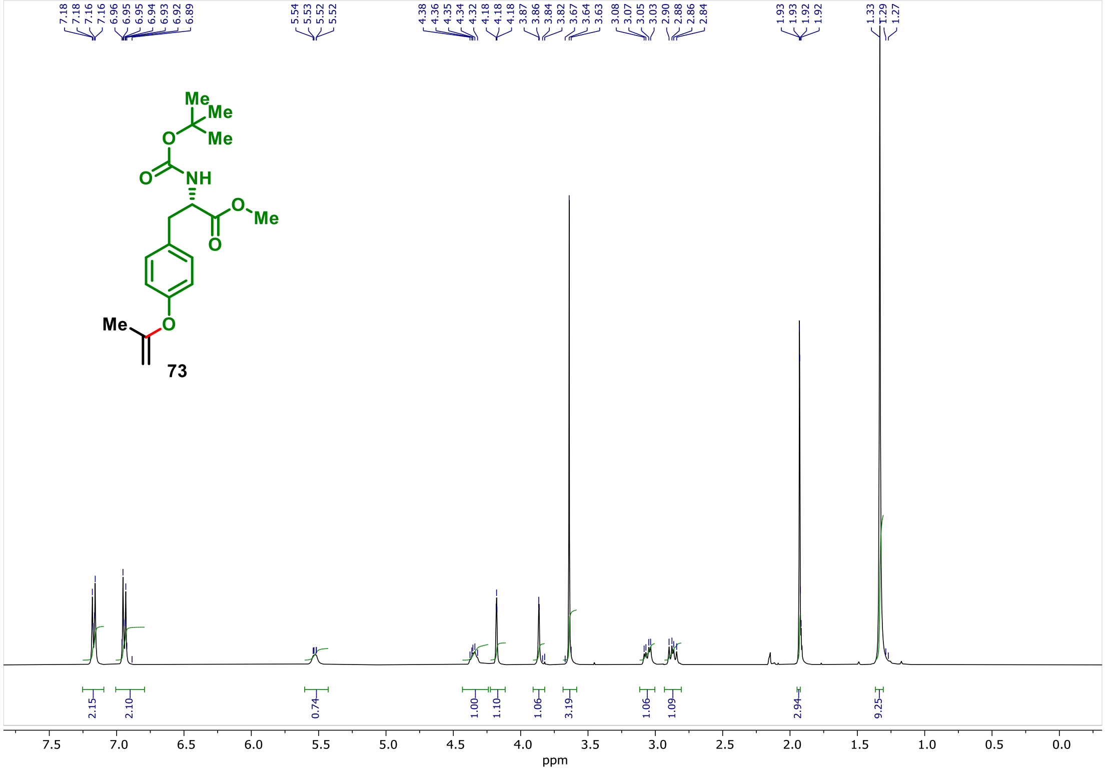


$^{13}$C NMR (101 MHz, $CD_3CN$) spectra of compound **73**

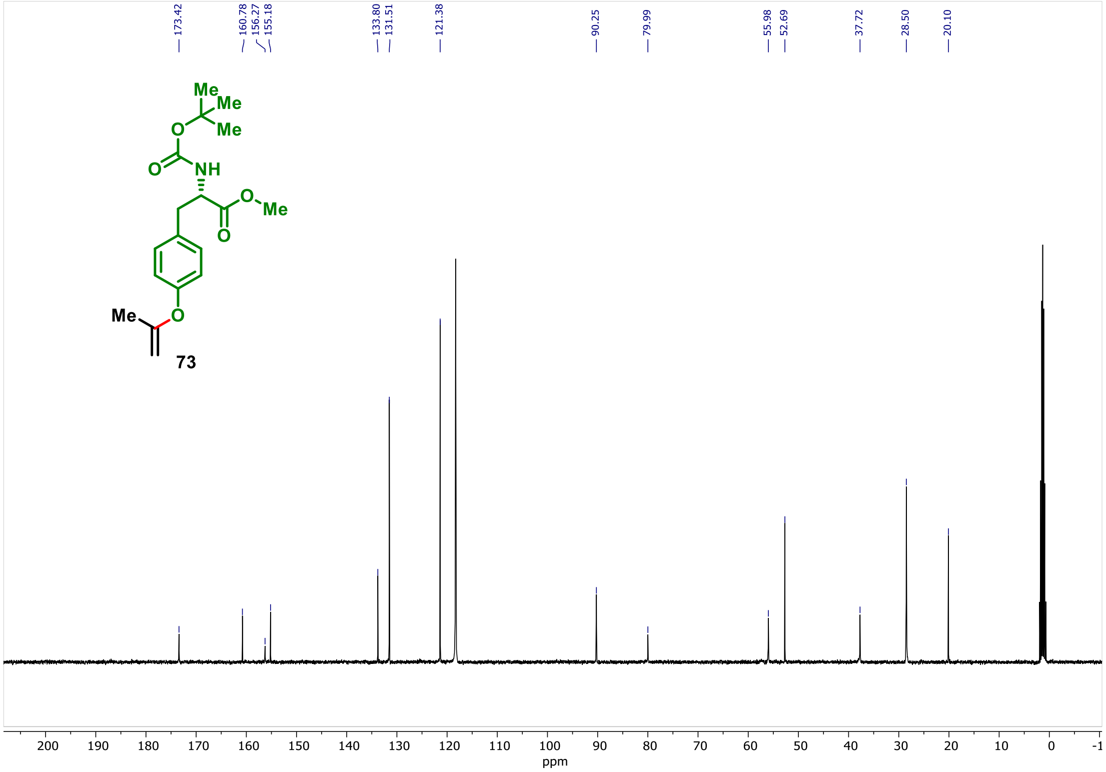

$^{1}$H NMR (400 MHz, $CD_3CN$) spectra of compound **74**

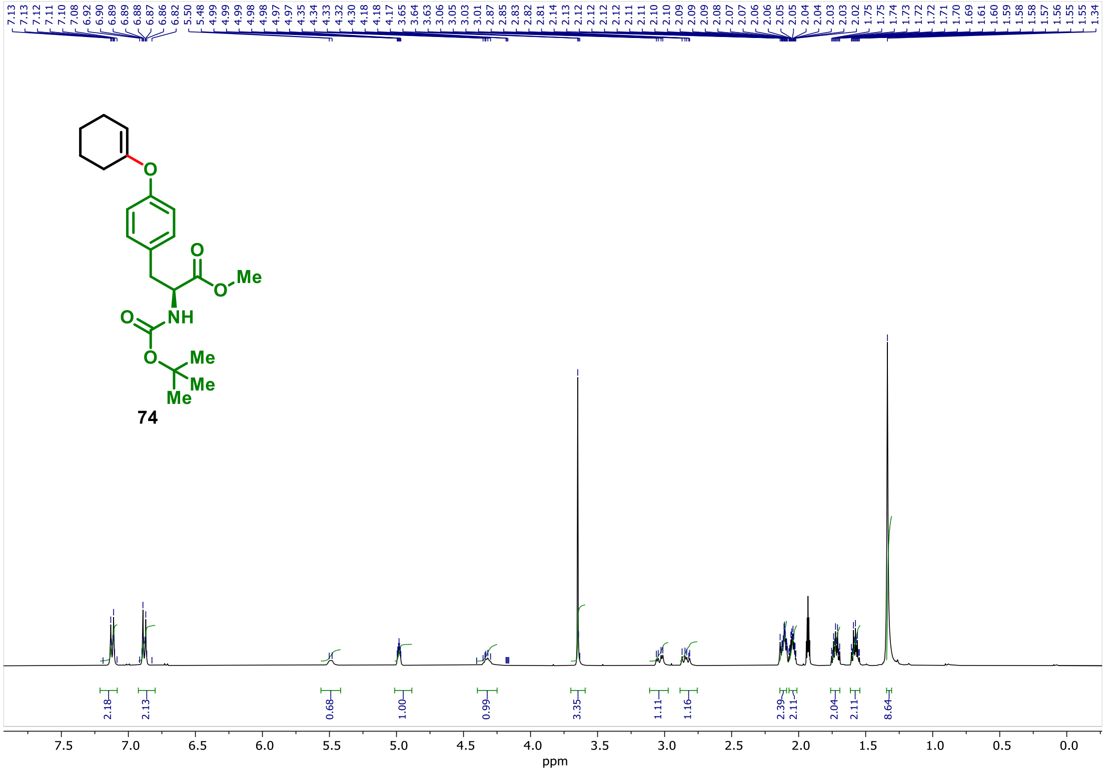


$^{13}$C NMR (101 MHz, $CD_3CN$) spectra of compound **74**

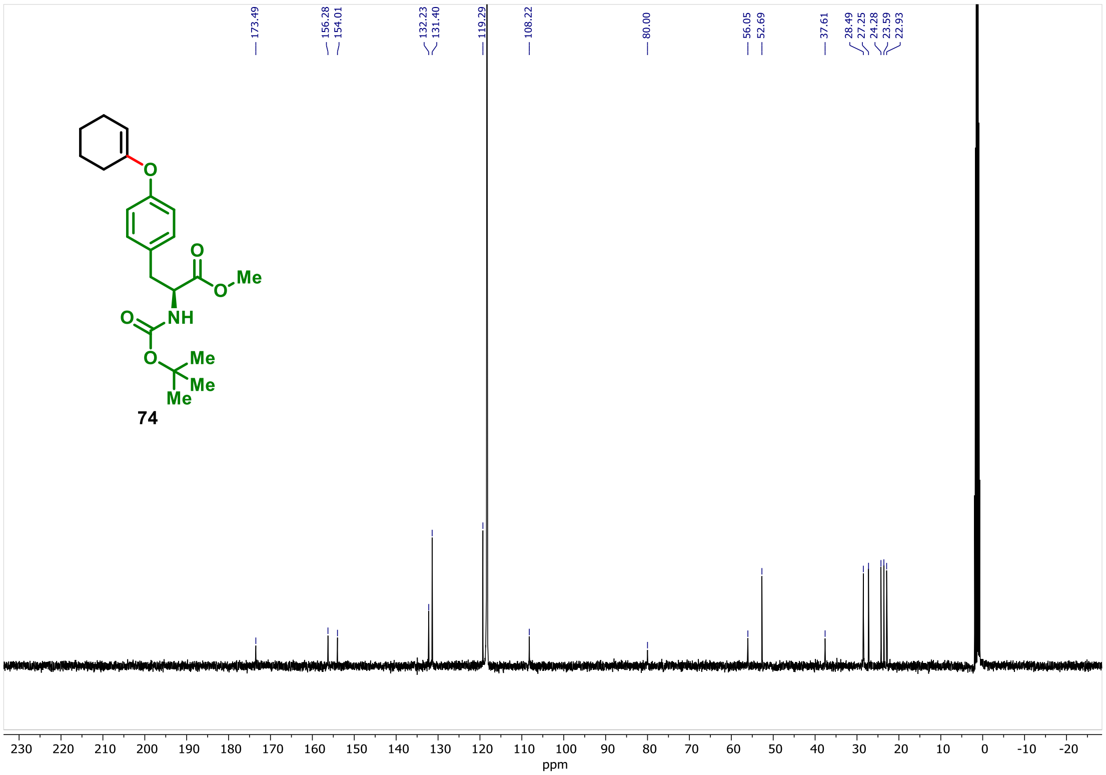

$^{1}$H NMR (400 MHz, $CD_3CN$) spectra of compound **75**

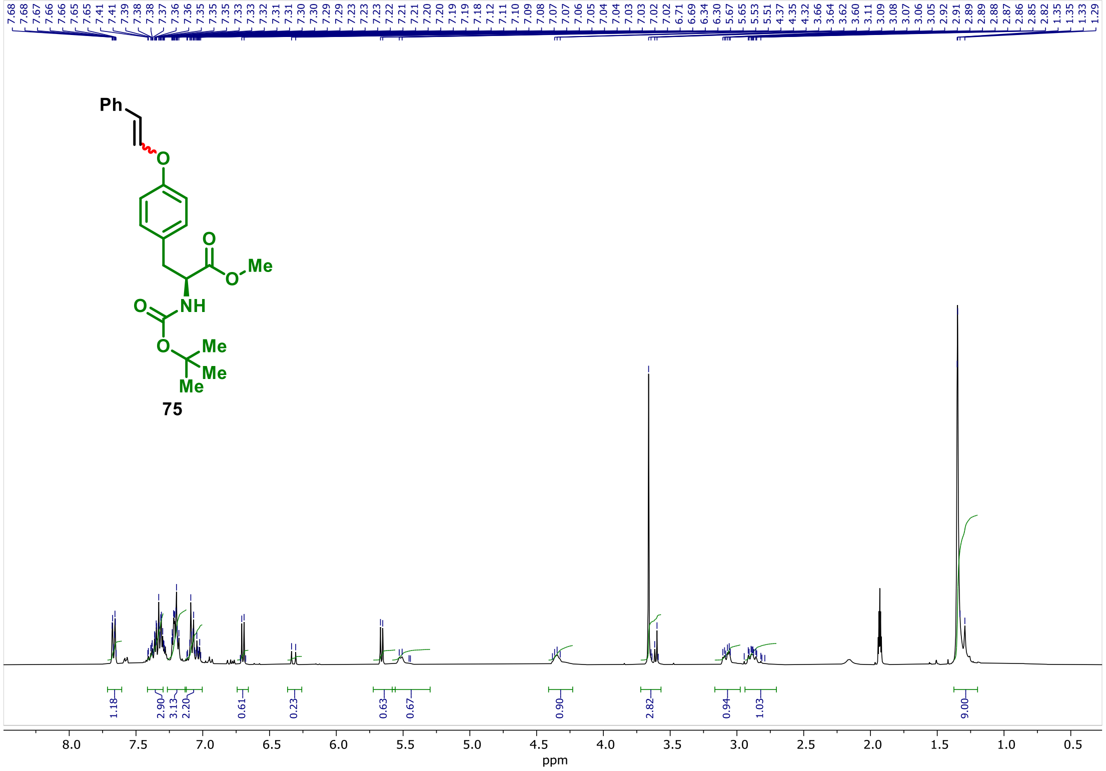


$^{13}$C NMR (101 MHz, $CD_3CN$) spectra of compound **75**

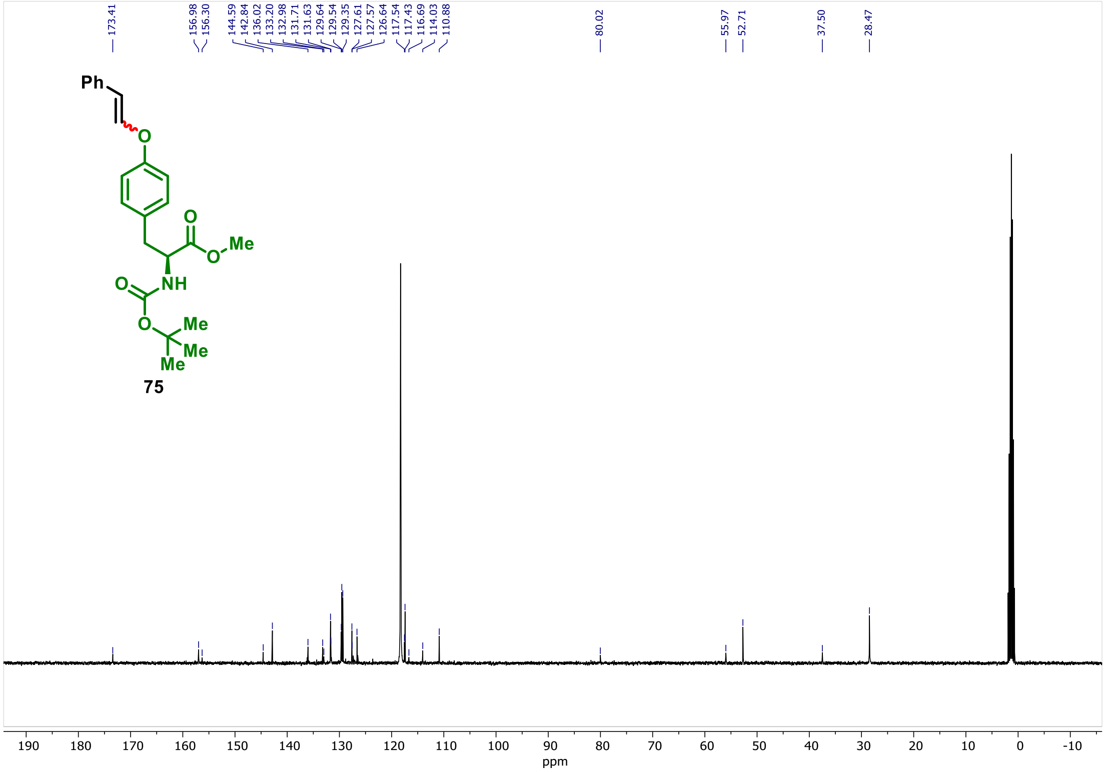

$^{1}$H NMR (400 MHz, $CD_3CN$) spectra of compound **76**

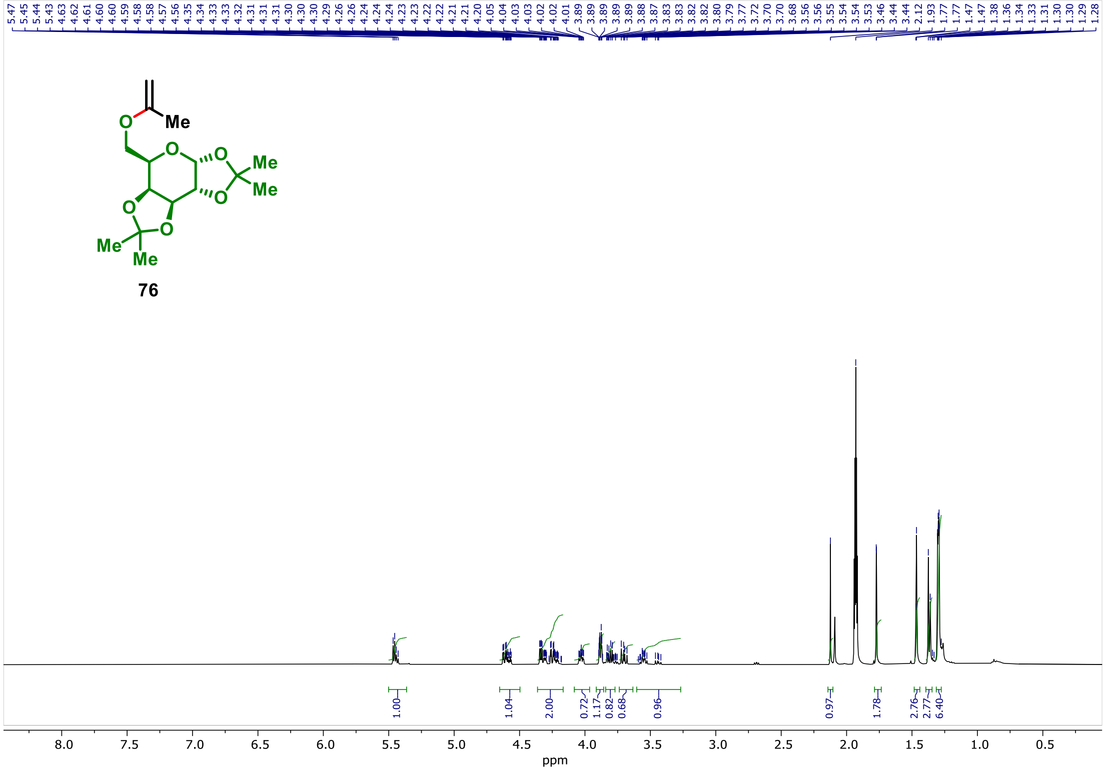


$^{13}$C NMR (101 MHz, $CD_3CN$) spectra of compound **76**

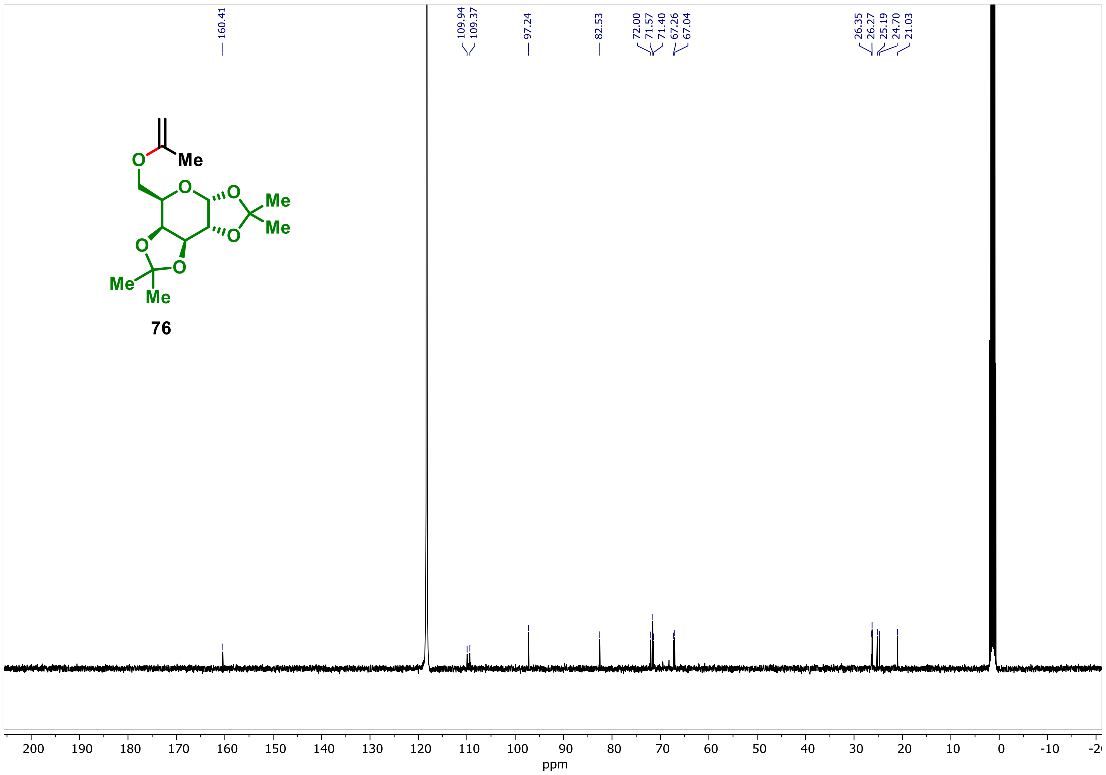

$^{1}H$ NMR (400 MHz, $CD_3CN$) spectra of compound **77**

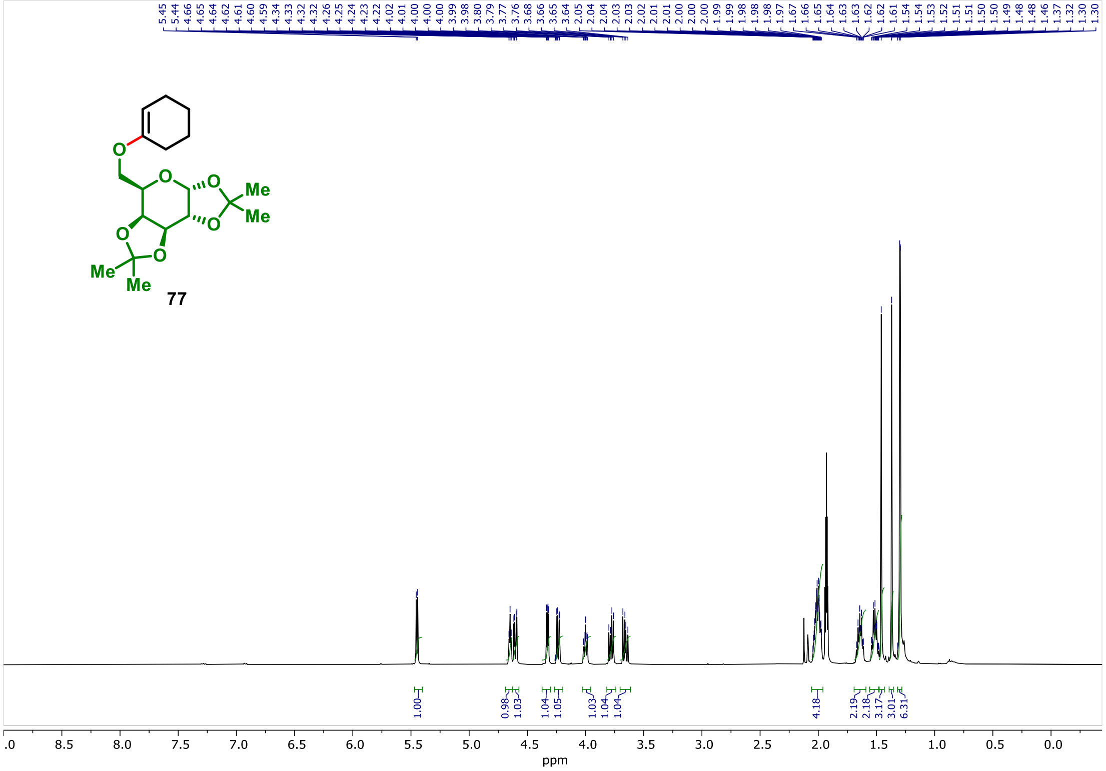


$^{13}C$ NMR (101 MHz, $CD_3CN$) spectra of compound **77**

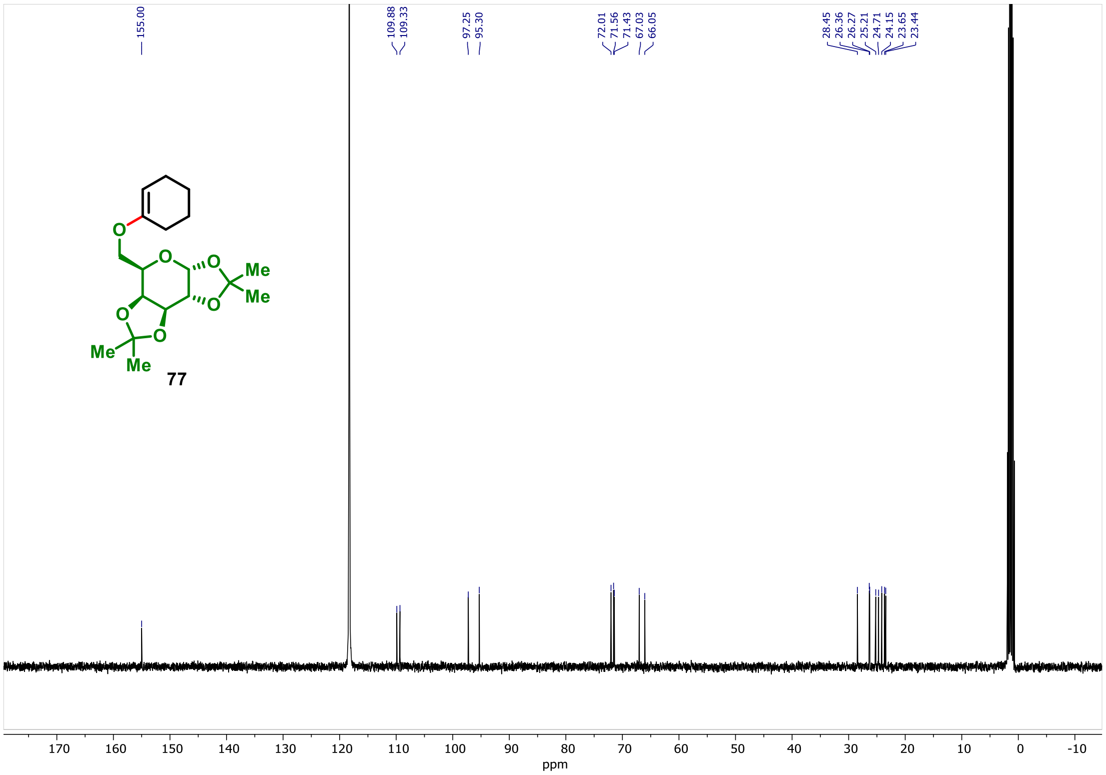

$^{1}$H NMR (400 MHz, $CD_3CN$) spectra of compound **78**

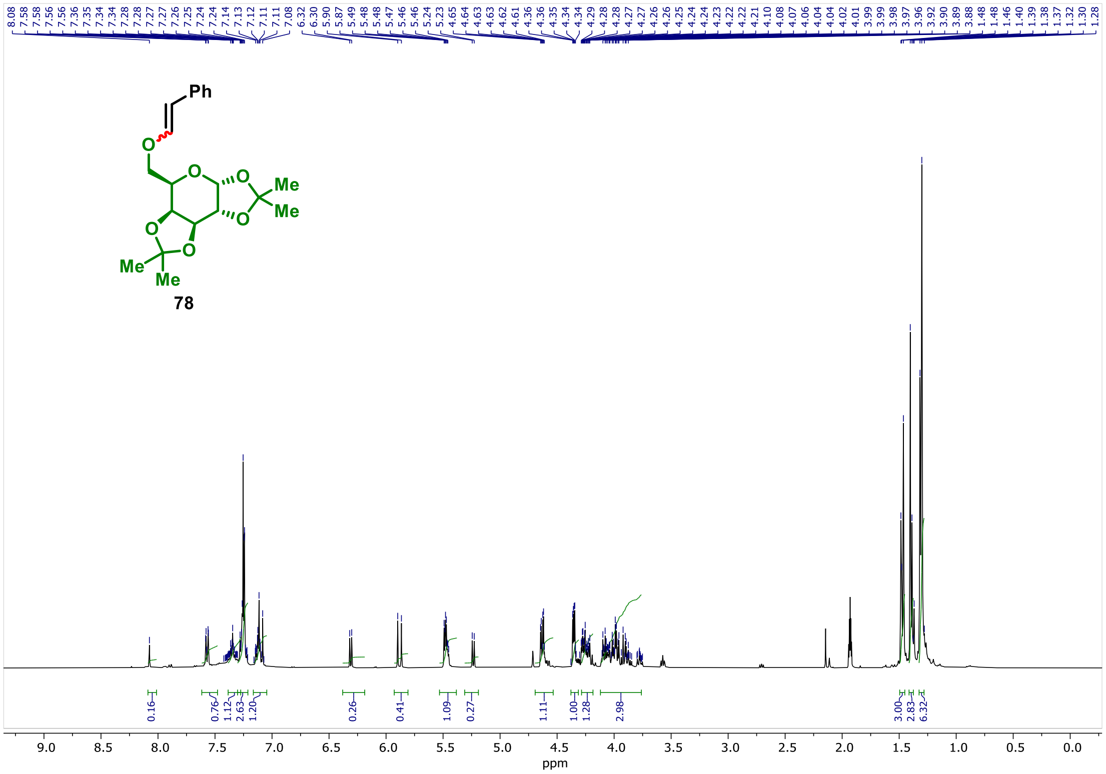

$^{13}$C NMR (101 MHz, $CD_3CN$) spectra of compound **78**

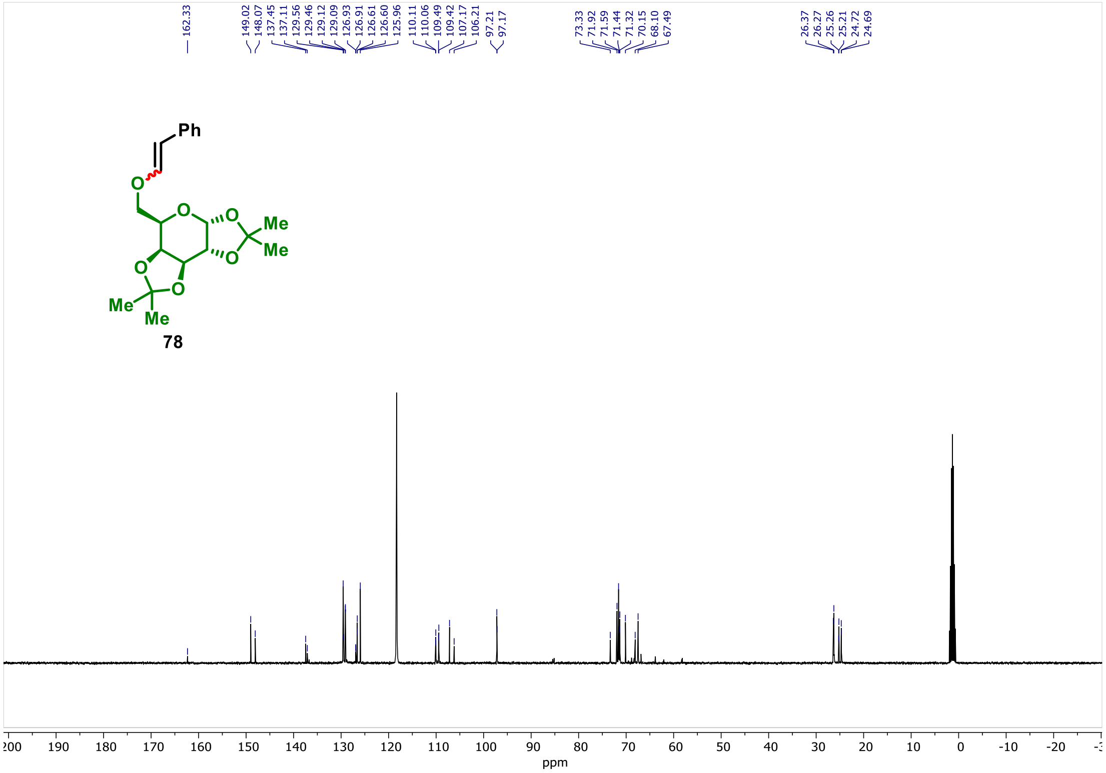

$^{1}$H NMR (400 MHz, $CD_3CN$) spectra of compound 7**9**

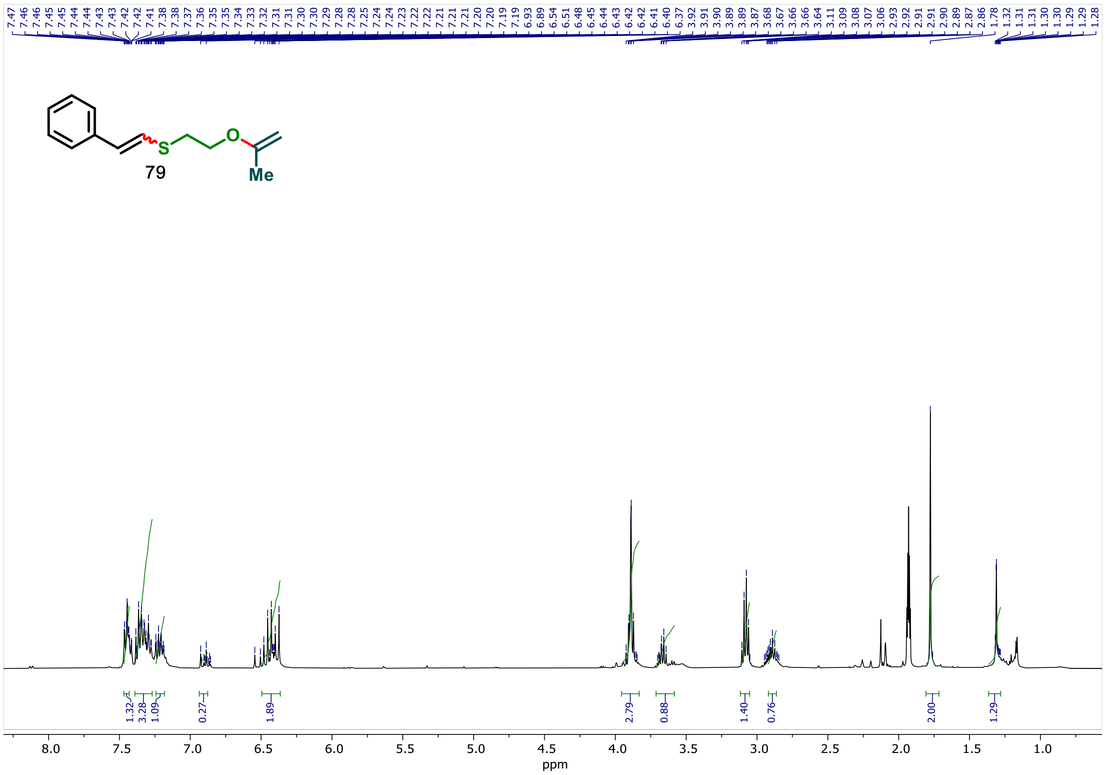


$^{13}$C NMR (101 MHz, $CD_3CN$) spectra of compound **79**

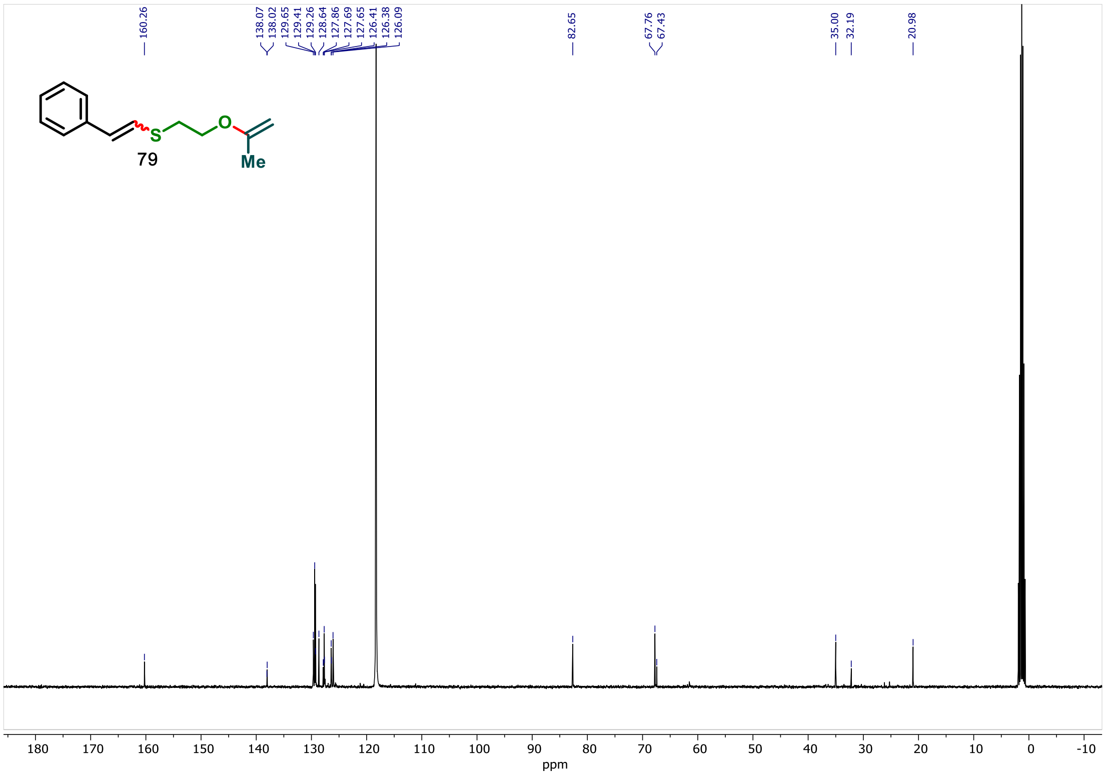

$^{1}$H NMR (400 MHz, $CD_3CN$) spectra of compound **80**

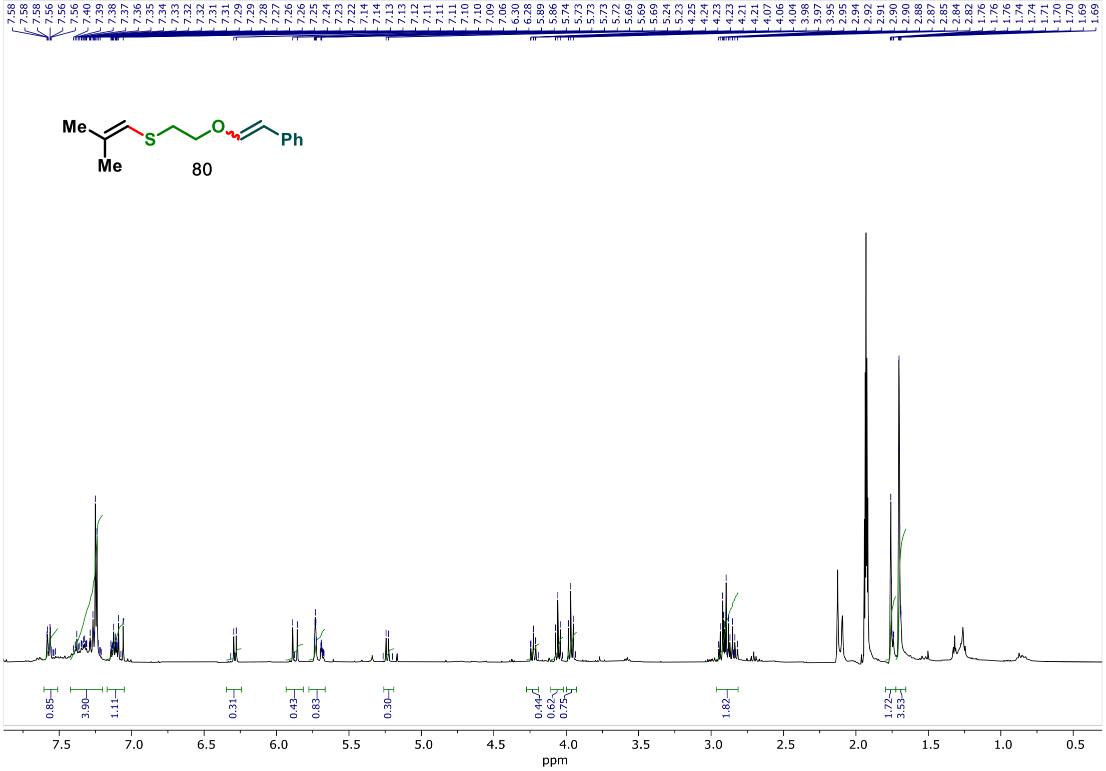


$^{13}$C NMR (101 MHz, $CD_3CN$) spectra of compound **80**

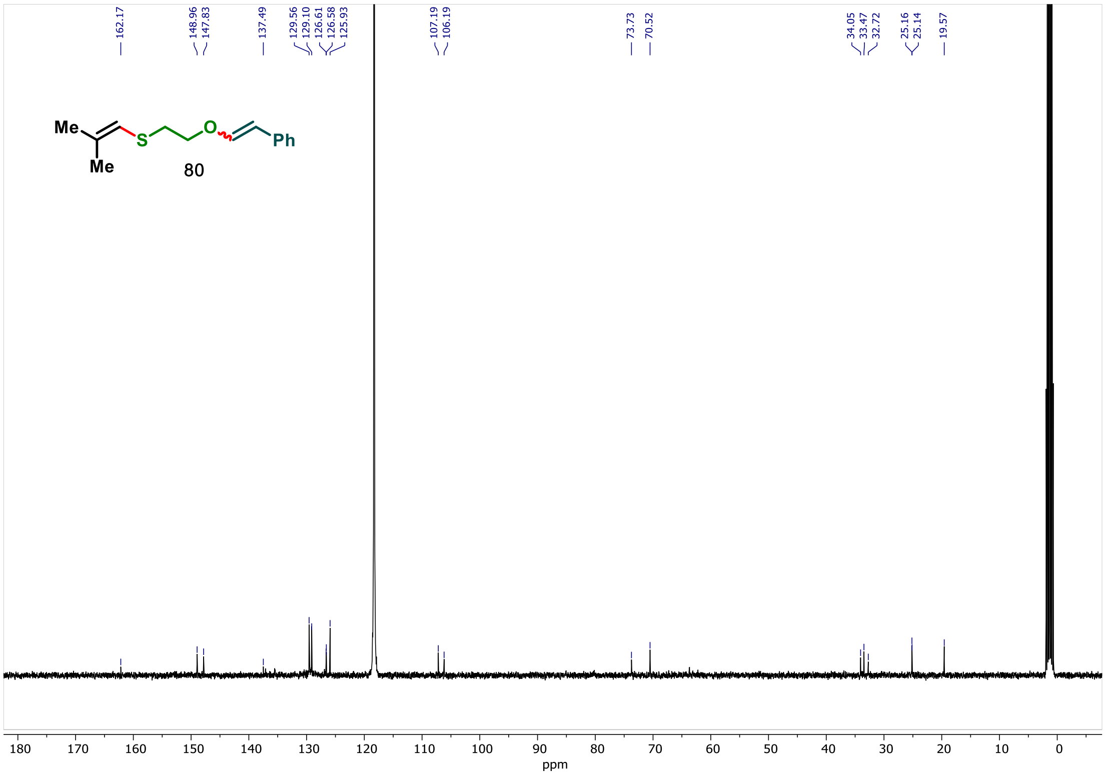

$^{1}$H NMR (400 MHz, $CDCl_3$) spectra of compound **81**

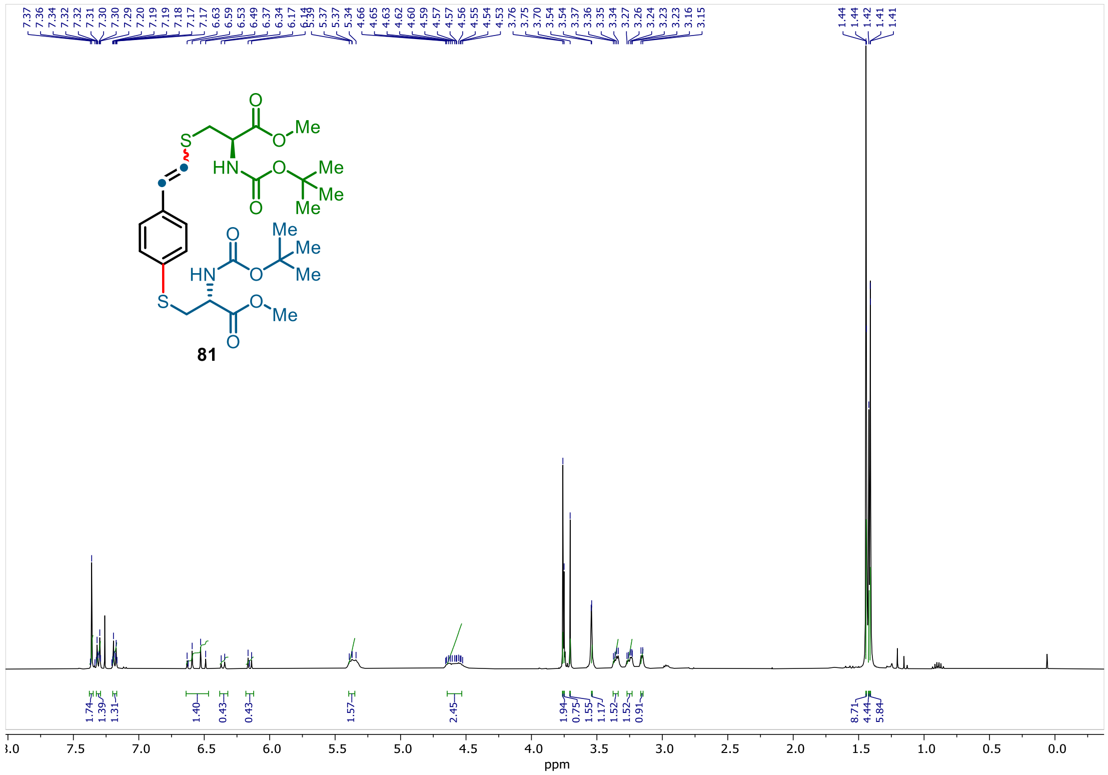

$^{13}$C NMR (101 MHz, $CDCl_3$) spectra of compound **81**

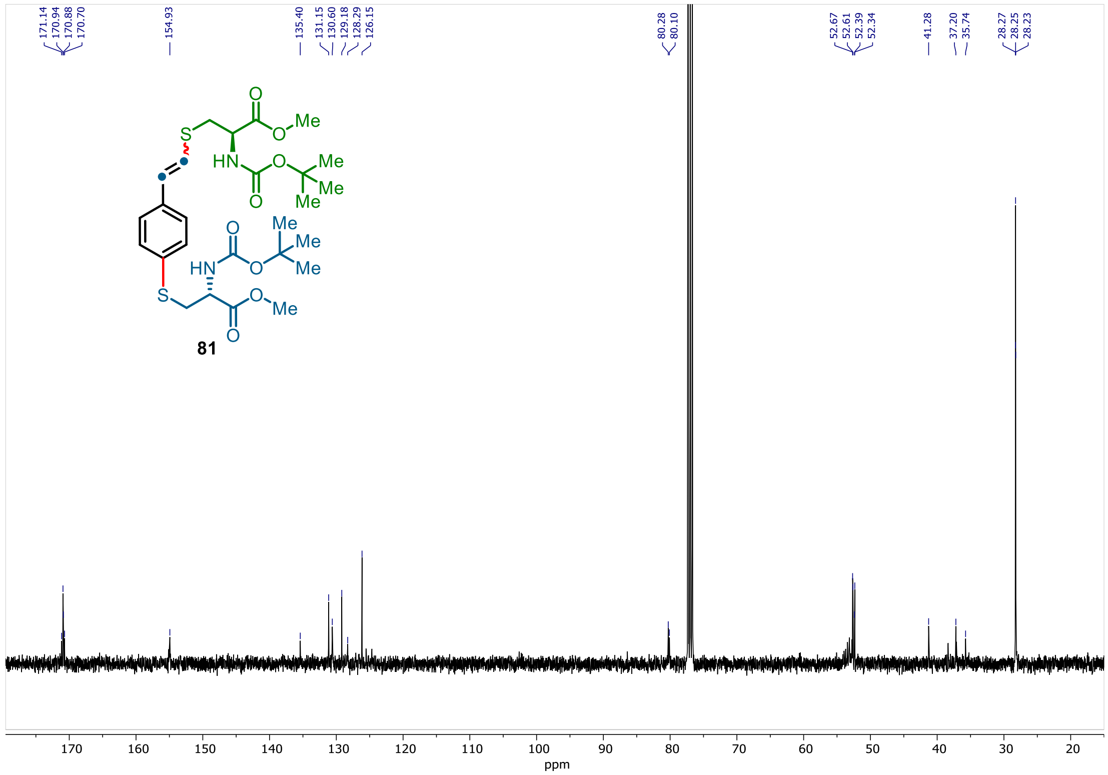

$^{1}$H NMR (400 MHz, $CD_3CN$) spectra of compound **82**

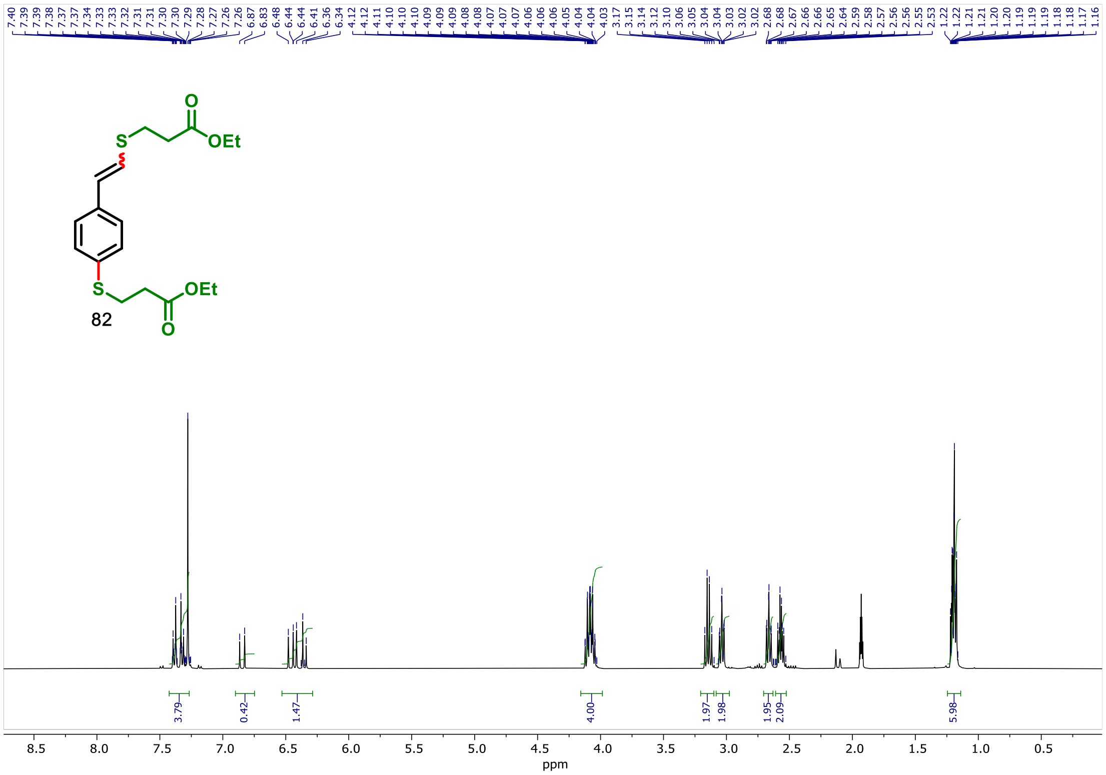

$^{13}$C NMR (101 MHz, $CD_3CN$) spectra of compound **82**

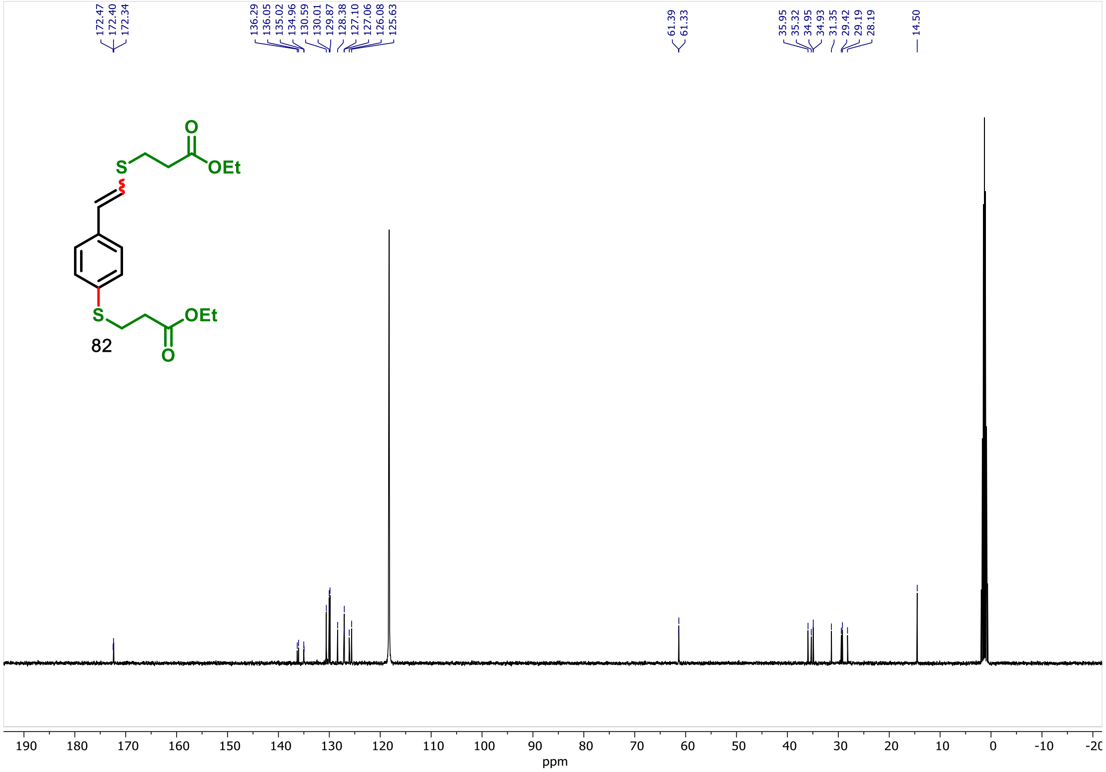

$^{1}H$ NMR (400 MHz, $CD_3CN$) spectra of compound **83**

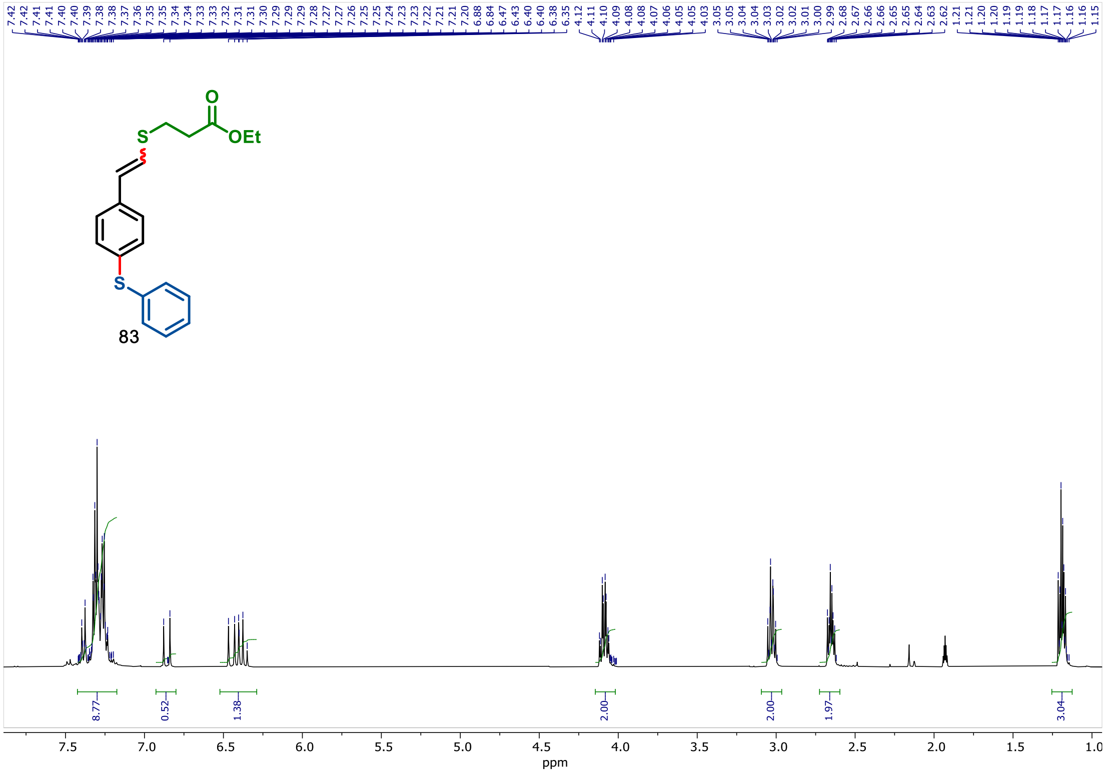


$^{13}C$ NMR (101 MHz, $CD_3CN$) spectra of compound **83**

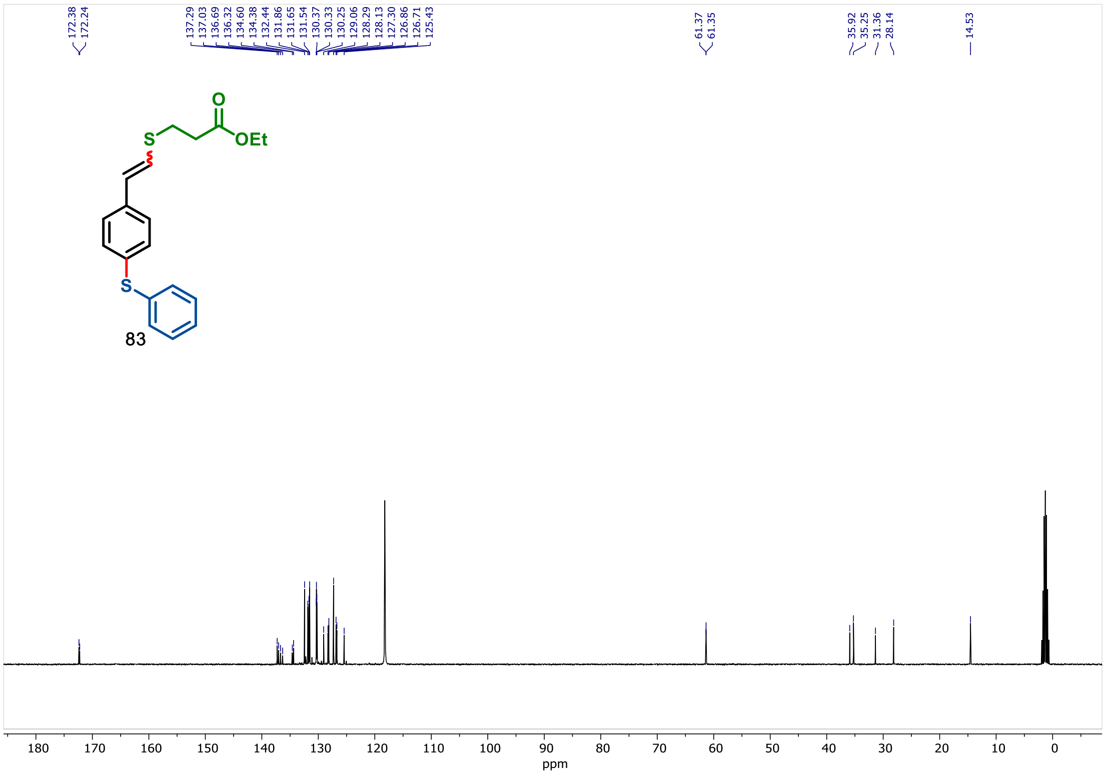

$^{1}$H NMR (400 MHz, $CD_3CN$) spectra of compound **84**

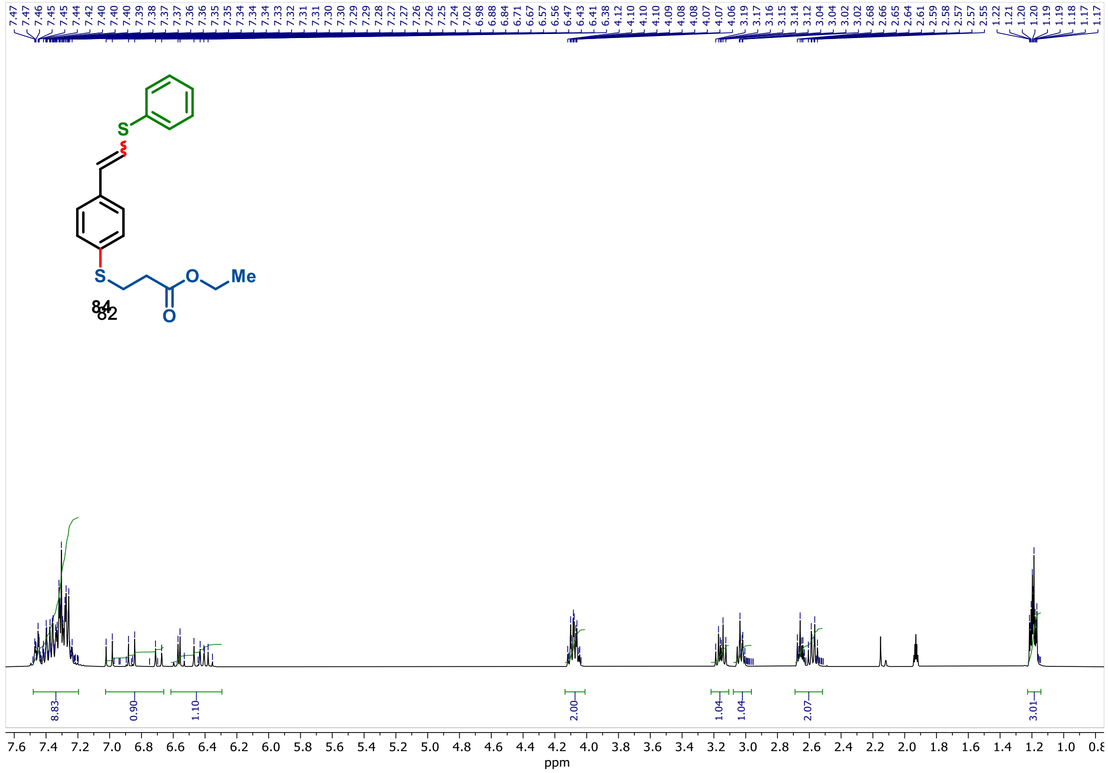

$^{13}$C NMR (101 MHz, $CD_3CN$) spectra of compound **84**

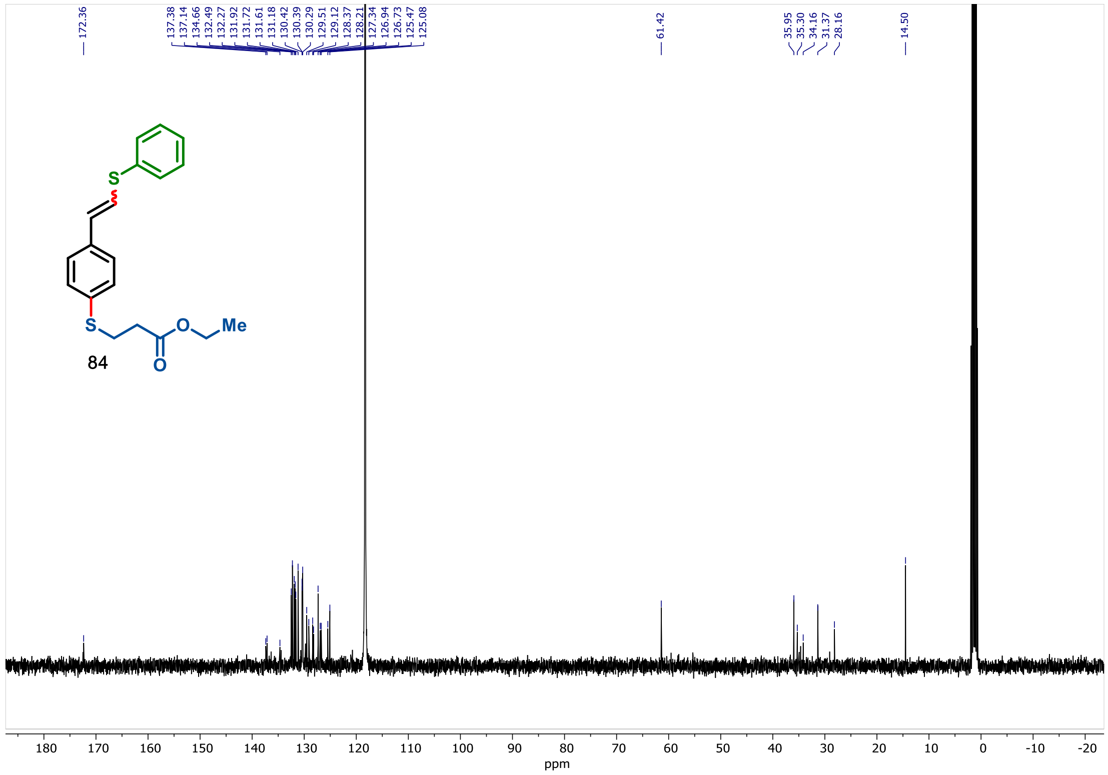

$^{1}$H NMR (400 MHz, $CD_3CN$) spectra of compound **85**

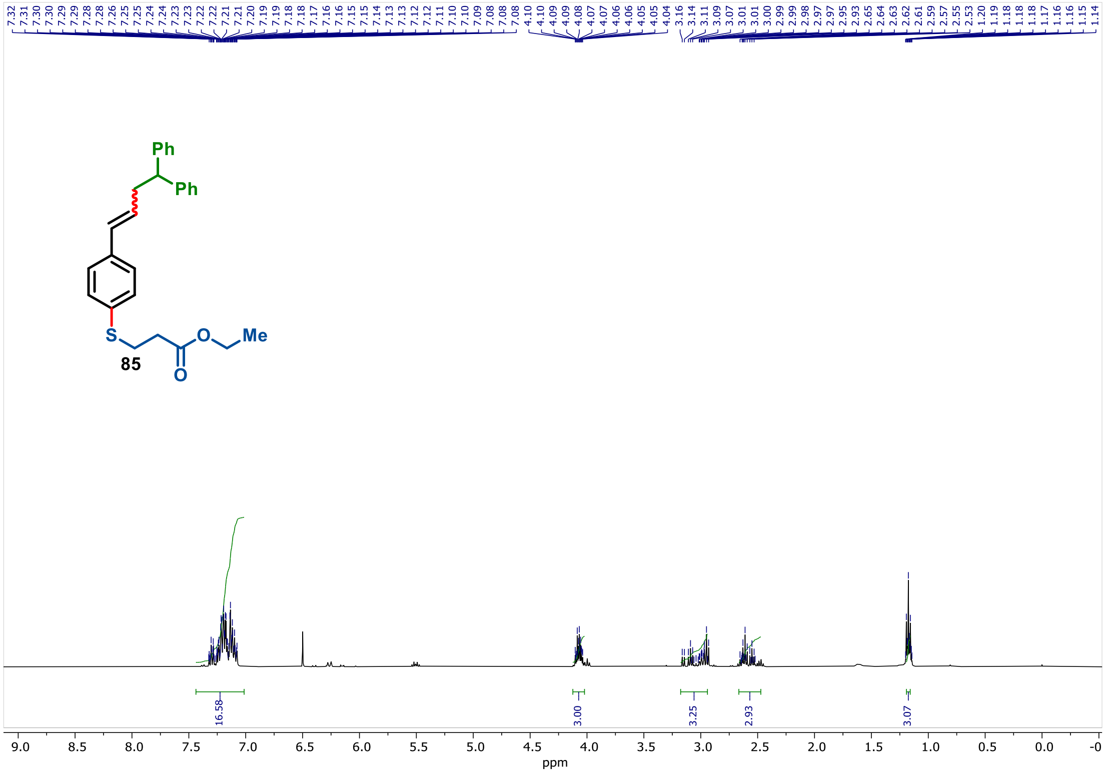

$^{13}$C NMR (101 MHz, $CD_3CN$) spectra of compound **85**

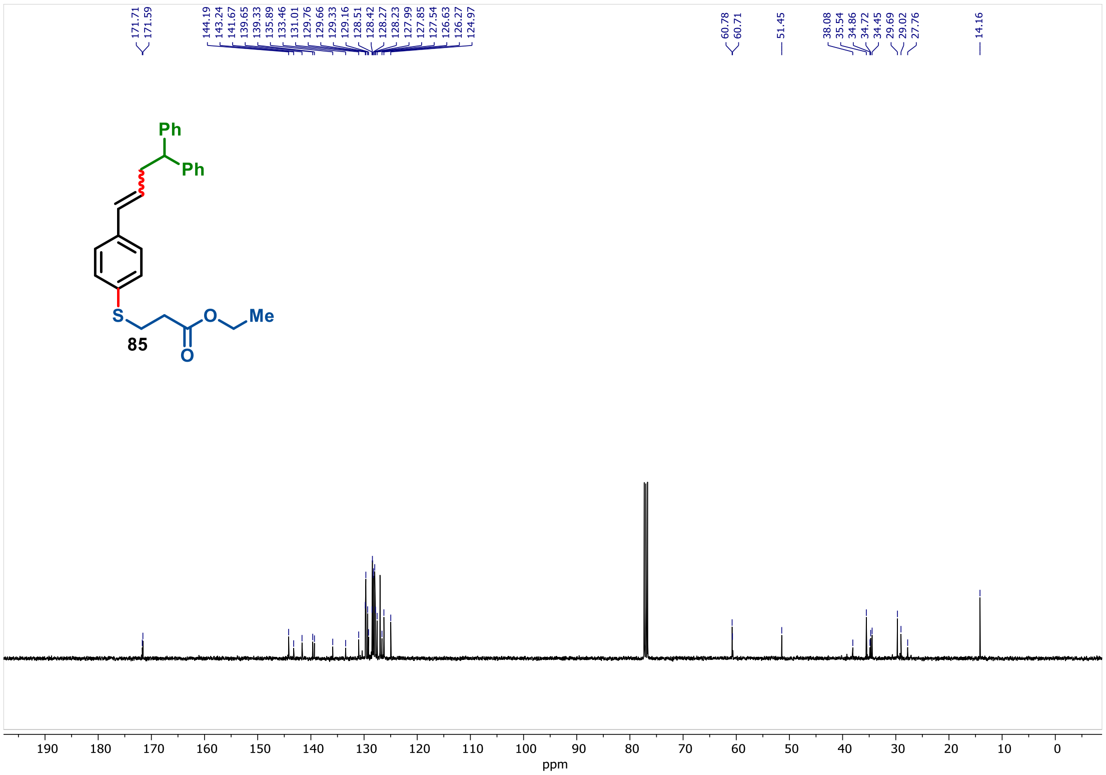

$^{1}H$ NMR (400 MHz, $CD_3CN$) spectra of compound **86**

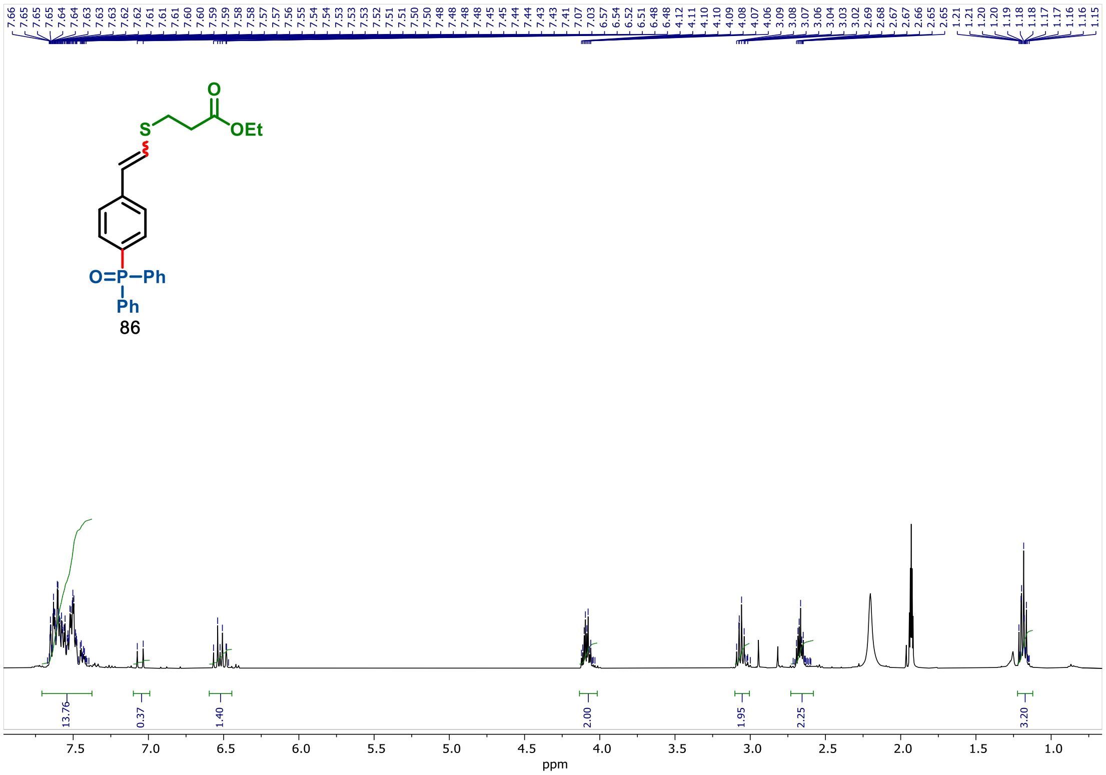


$^{13}C$ NMR (101 MHz, $CD_3CN$) spectra of compound **86**

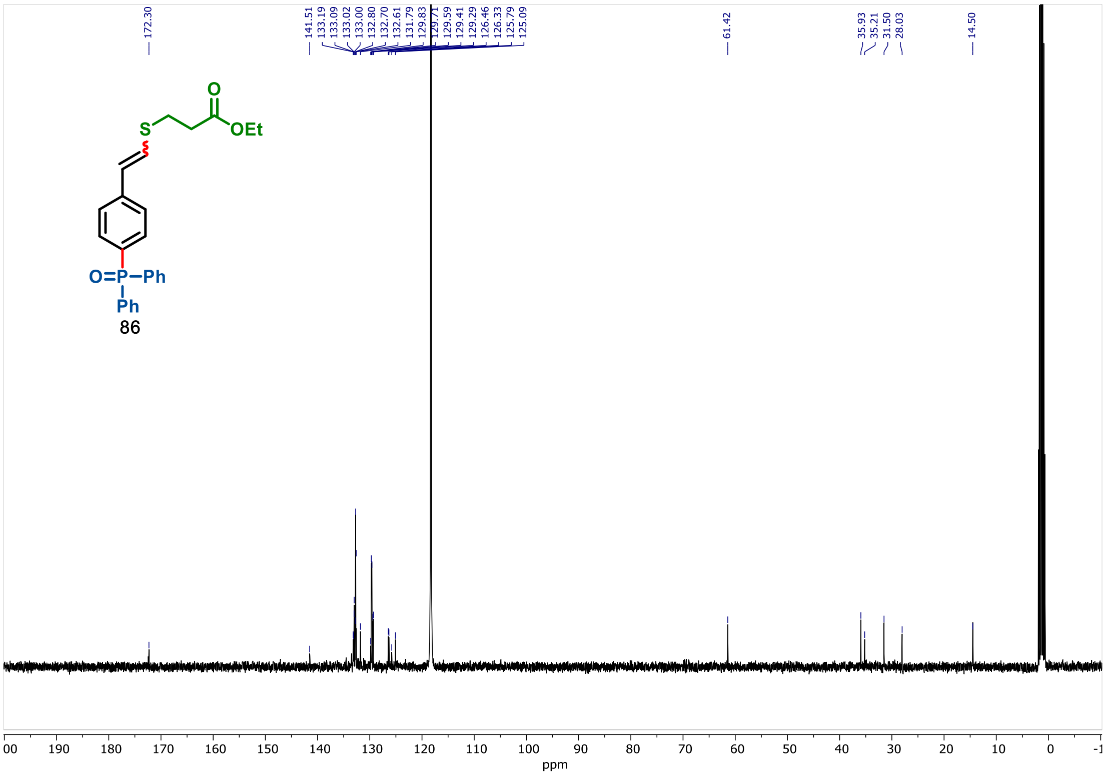

$^{31}$P NMR (162 MHz, $CD_3CN$) spectra of compound **86**

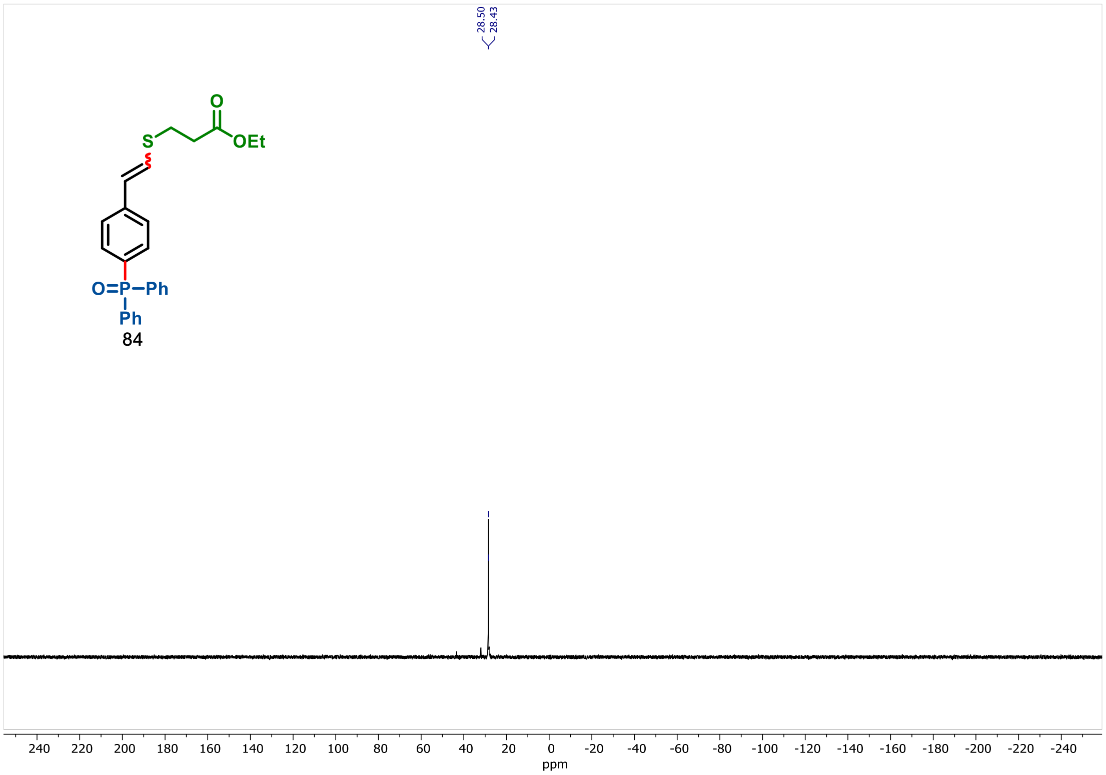

$^{1}H$ NMR (400 MHz, $CD_3CN$) spectra of compound **87**

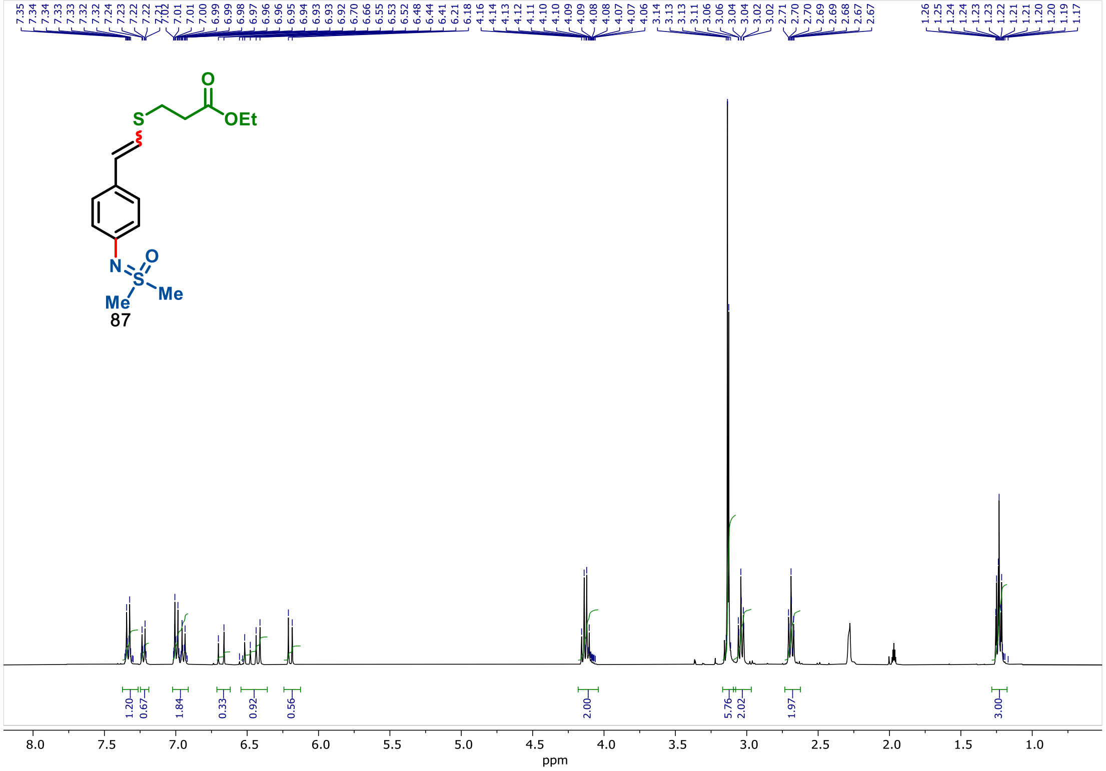


$^{13}C$ NMR (101 MHz, $CD_3CN$) spectra of compound **87**

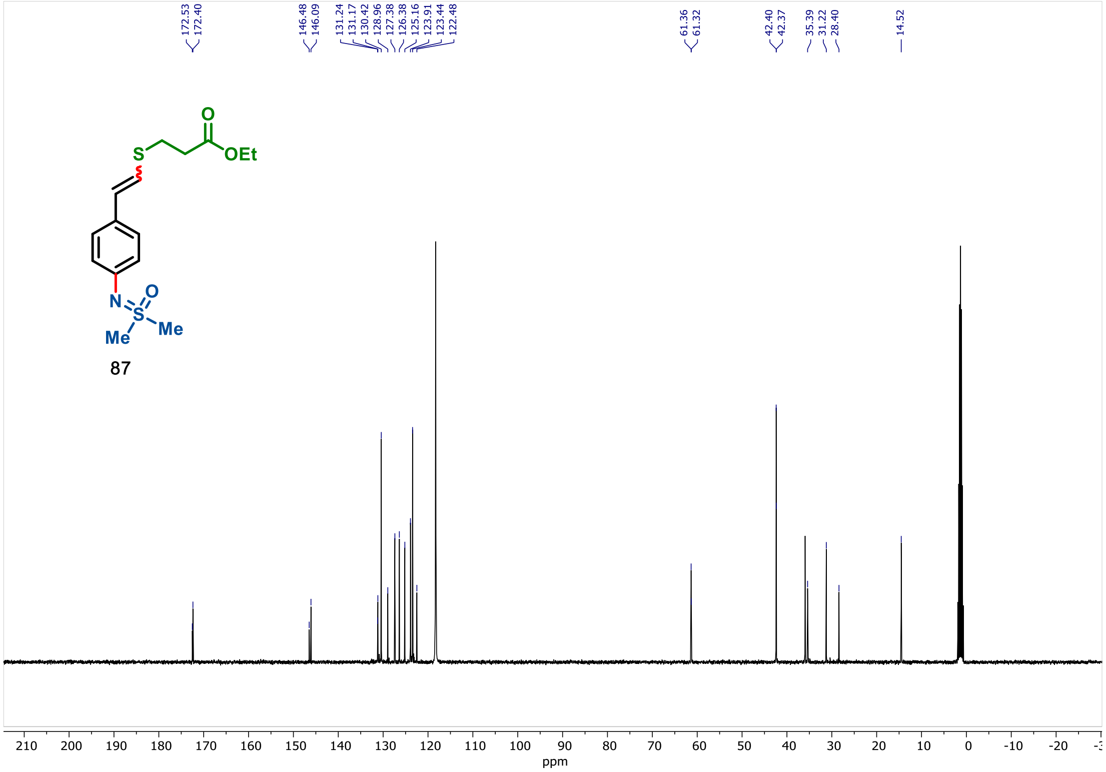

$^{1}$H NMR (400 MHz, $CDCl_3$) spectra of compound **88**

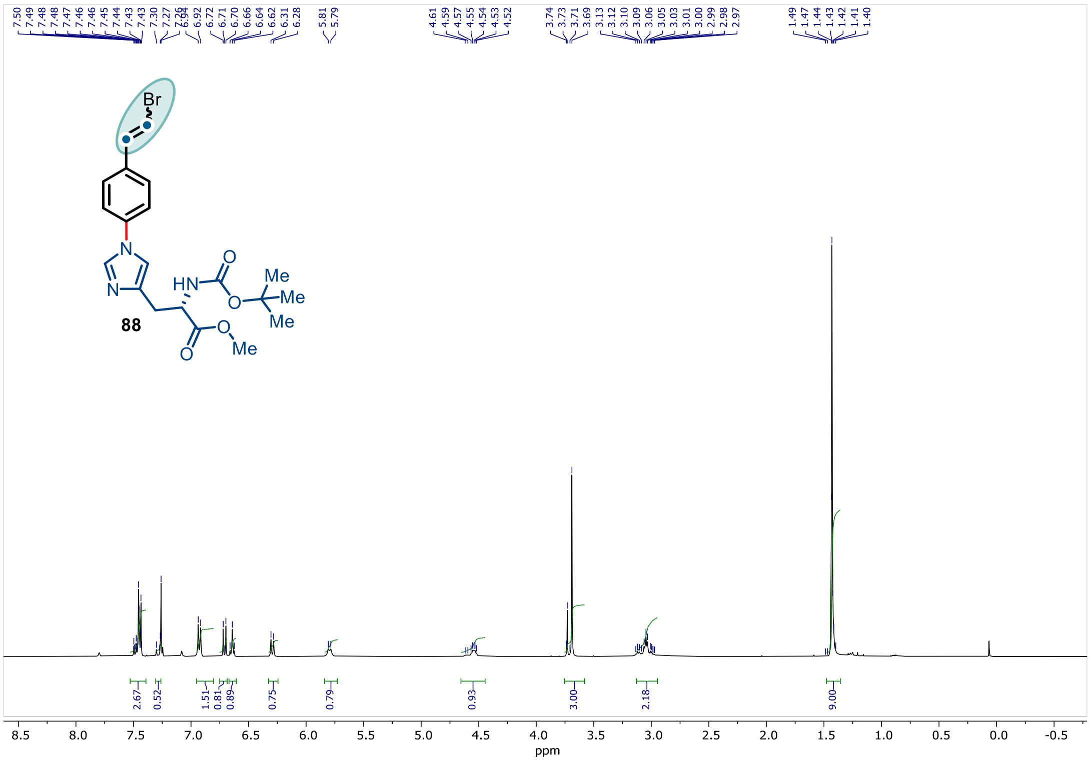


$^{13}$C NMR (101 MHz, $CDCl_3$) spectra of compound **88**

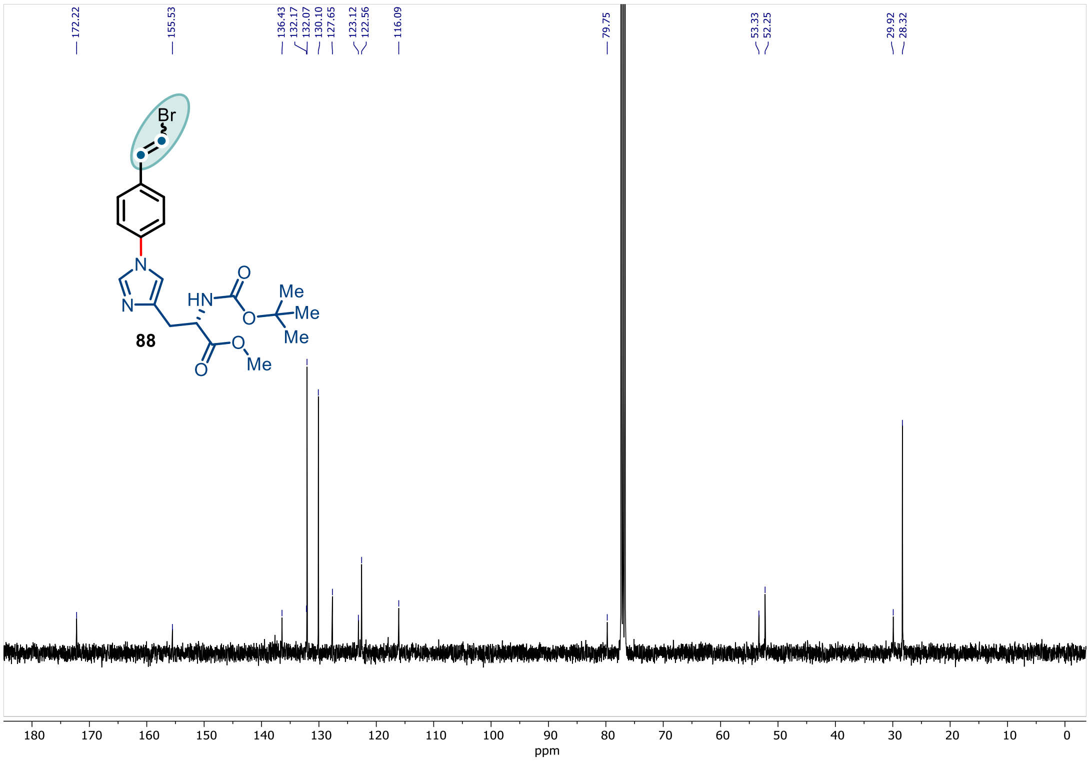

$^{1}H$ NMR (400 MHz, $CD_3CN$) spectra of compound **89**

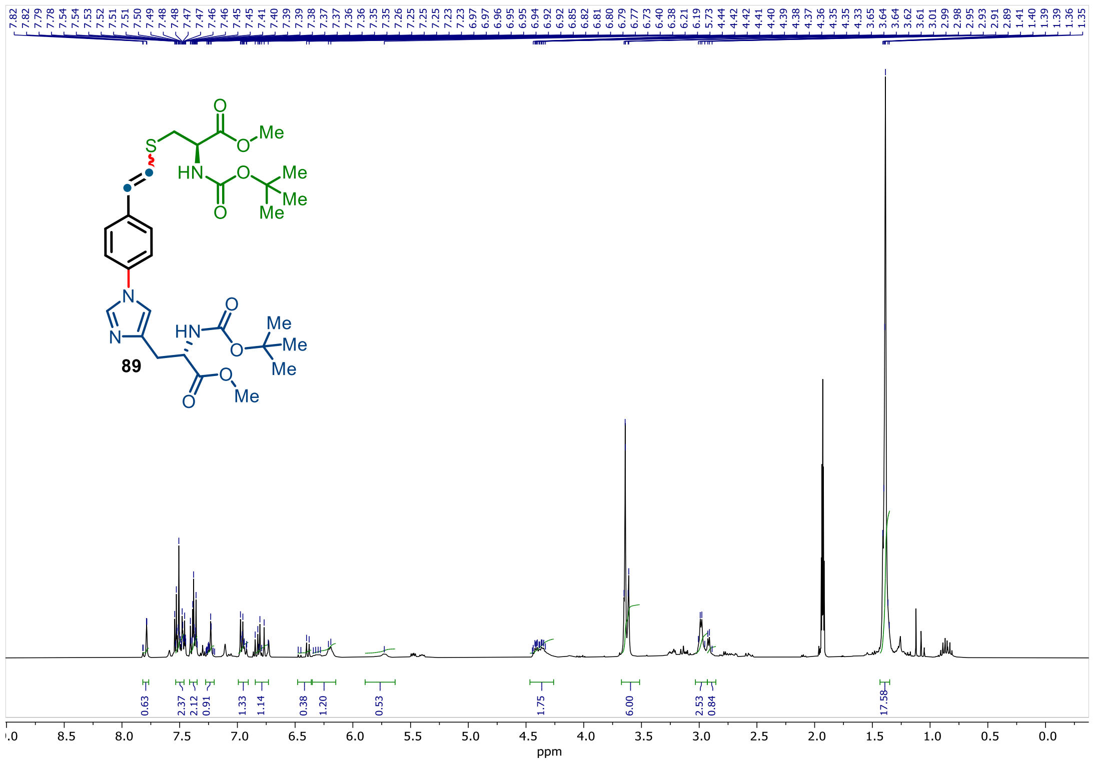

$^{13}C$ NMR (101 MHz, $CD_3CN$) spectra of compound **89**

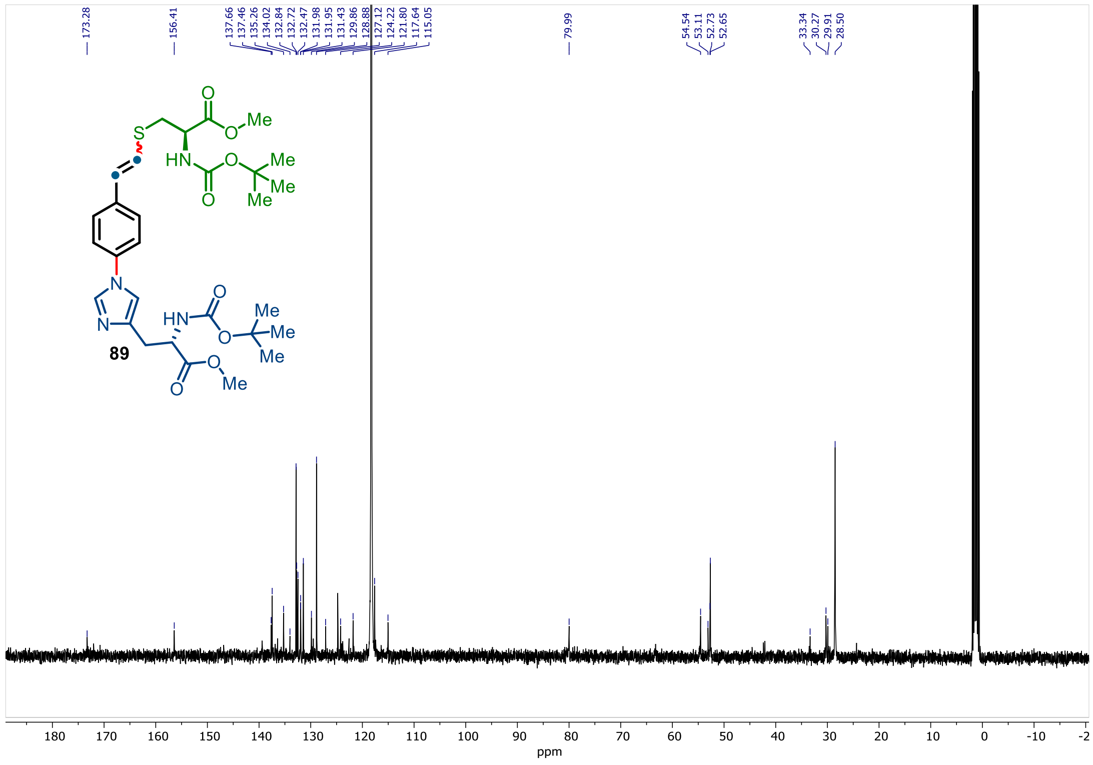

$^{1}$H NMR (400 MHz, $CDCl_3$) spectra of compound **90**

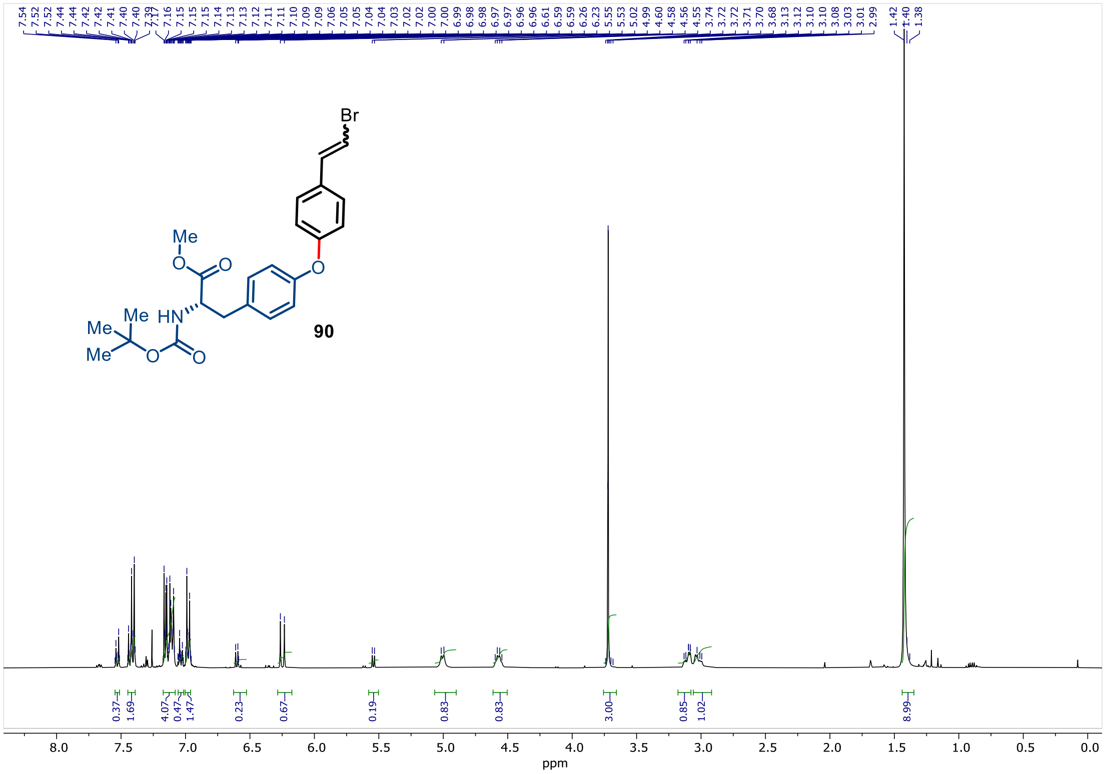

$^{13}$C NMR (101 MHz, $CDCl_3$) spectra of compound **90**

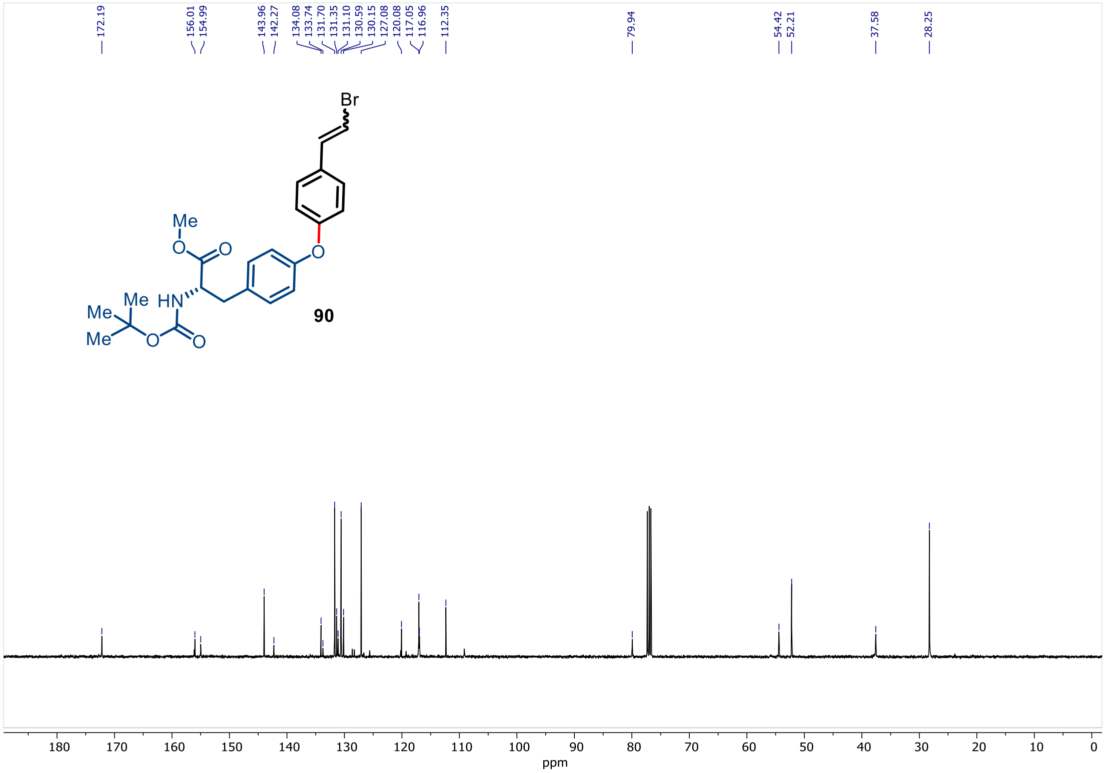

$^{1}$H NMR (400 MHz, $CDCl_3$) spectra of compound **91**

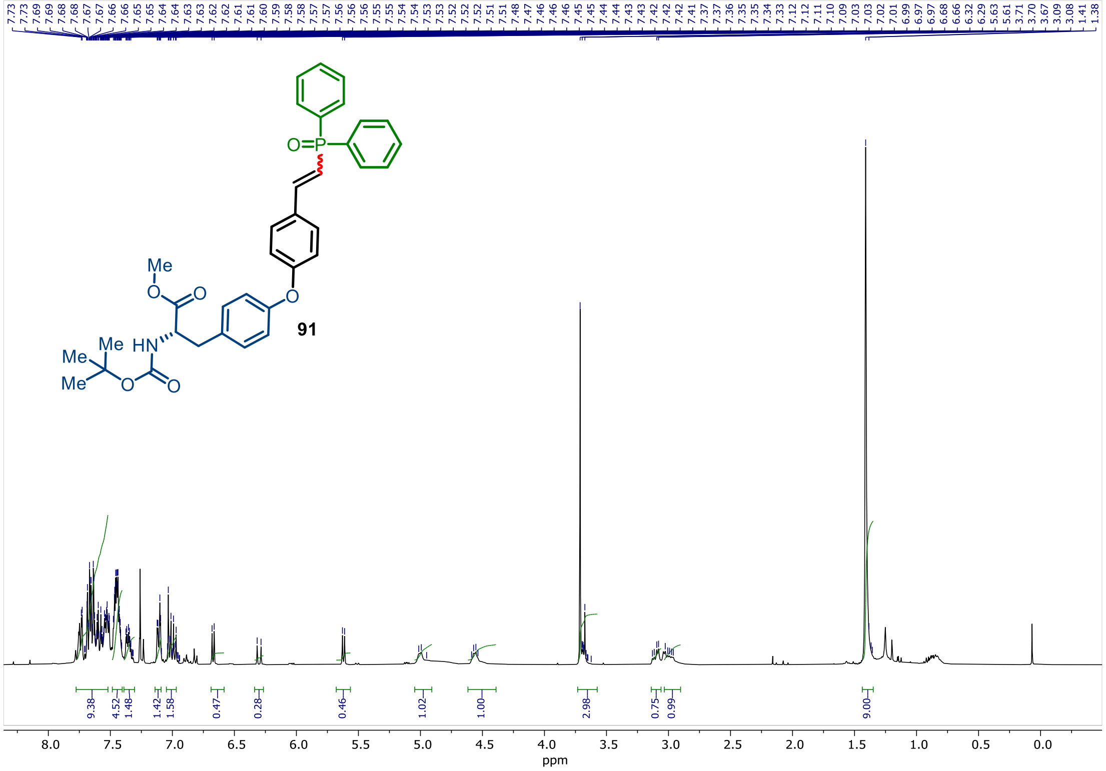


$^{13}$C NMR (101 MHz, $CDCl_3$) spectra of compound **91**

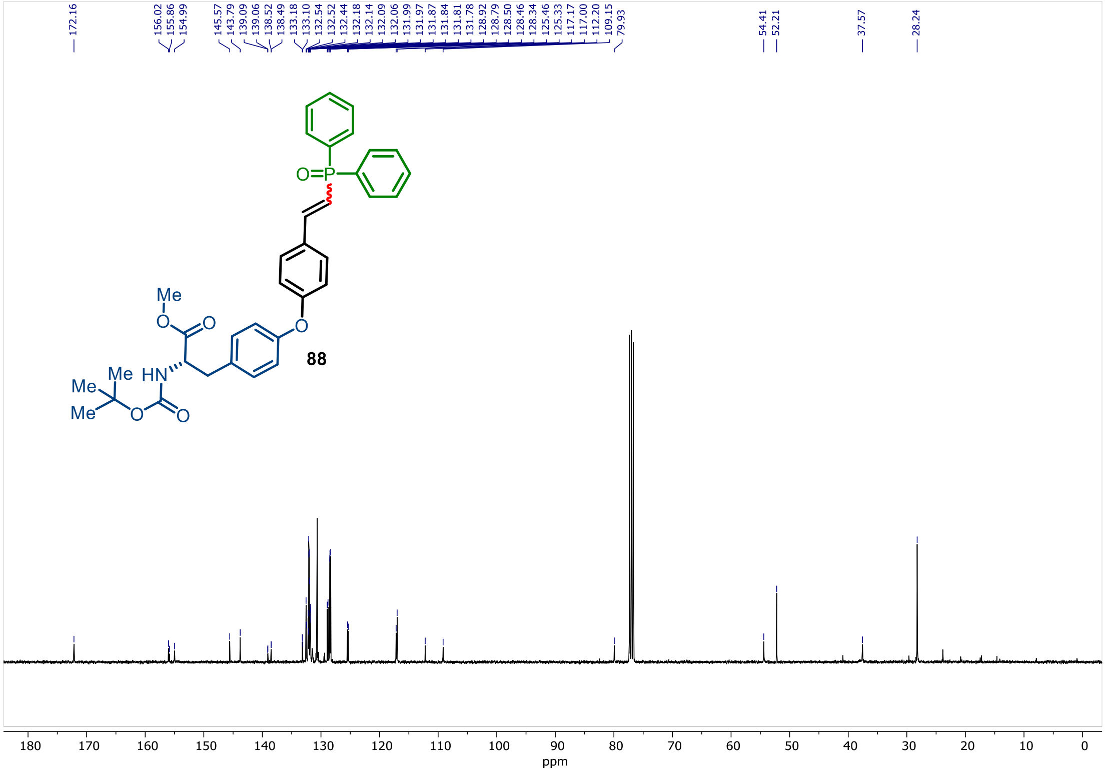

$^{31}$P NMR (162 MHz, $CDCl_3$) spectra of compound **91**

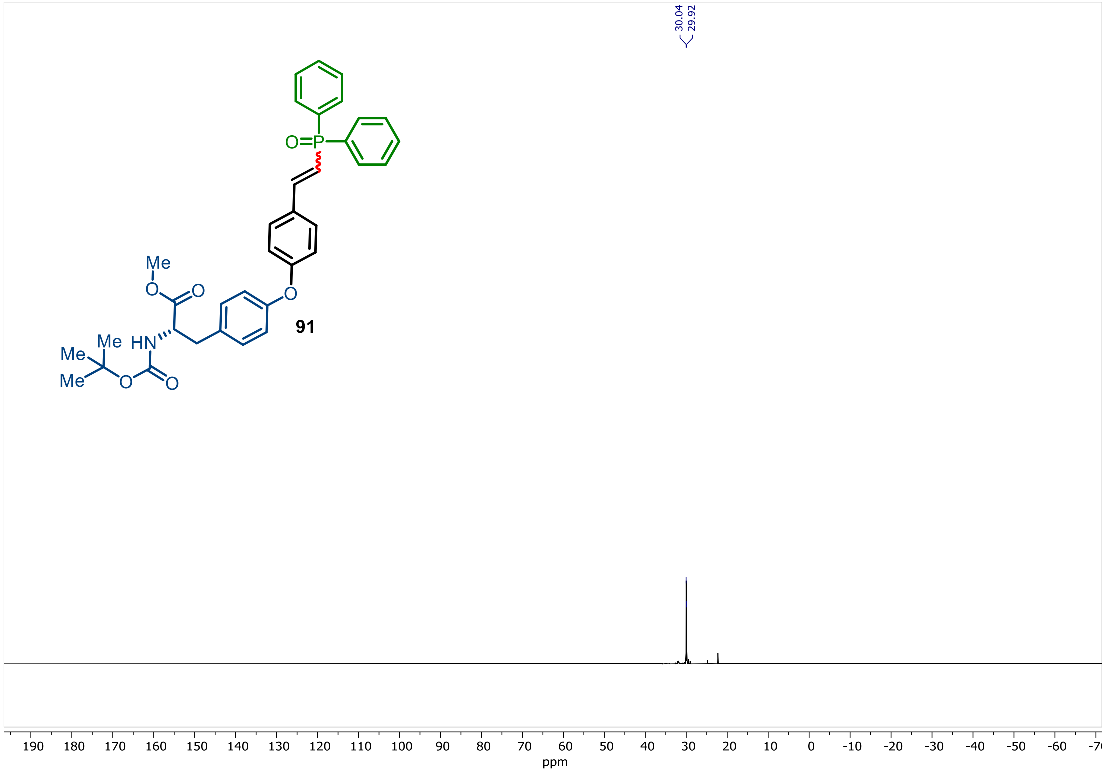

Supplement: Supplementary file 1 — Supporting Information [file ANIE-64-e202510715-s001.pdf]
